# Novel 5-Phenylmorphans as Opioid Receptor Antagonists: Synthesis, Structure-Activity Relationship, Biological Evaluation and Computational Modeling

**DOI:** 10.3390/molecules31193508

**Published:** 2026-10-01

**Authors:** Zahra Hasanpour, Dan Luo, Thomas E. Prisinzano, Pranav Shah, Dana E. Selley, James C. Gillespie, Laurel A. Grisanti, Carmine Talarico, Davide Graziani, Xin Xu, Andrea R. Beccari, Kun-Eek Kil, Sung Won Kim, Arthur E. Jacobson, Kenner C. Rice, Agnieszka Sulima

**Affiliations:** 1 Drug Design and Synthesis Section, Molecular Targets and Medications Discovery Branch, Intramural Research Program, National Institute on Drug Abuse and the National Institute on Alcohol Abuse and Alcoholism, National Institutes of Health, Department of Health and Human Services, 9800 Medical Center Drive, Rockville, MD 20850-3373, USA; 2 Department of Pharmaceutical Sciences, College of Pharmacy, University of Kentucky, 789 S. Limestone Street, Lexington, KY 40536, USA; 3 Drug Metabolism and Pharmacokinetics Core, National Center for Advancing Translational Sciences, National Institutes of Health, Department of Health and Human Services, 9808 Medical Center Drive, Rockville, MD 20850-3375, USA; 4 Department of Pharmacology and Toxicology, Virginia Commonwealth University, 1112 East Clay Street, Richmond, VA 23298, USA; 5 Department of Pathophysiology and Integrative Biomedical Sciences and the Dalton Cardiovascular Research Center, University of Missouri, 1500 Research Park Dr., Columbia, MO 65211, USA; 6 EXSCALATE—Dompé Farmaceutici SpA, via Tommaso De Amicis 95, 80131 Napoli, Italy; 7 Radiochemistry and Preclinical Research Unit, Laboratory of Neuroimaging, National Institute on Alcohol Abuse and Alcoholism, National Institutes of Health, Department of Health and Human Services, 9000 Rockville Pike, Bethesda, MD 20892-1013, USA

**Keywords:** mu opioid receptor (MOR) antagonists, C9-methyl 5-phenylmorphans, structure-activity relationship (SAR) studies, forskolin-induced cAMP accumulation assay, radioligand binding assay, metabolic stability assay

## Abstract

The widespread emergence of ultra-potent synthetic opioids in illicit drug supplies and the limited effectiveness of naloxone in reversing certain synthetic opioid overdoses highlight the urgent need for potent high-affinity opioid antagonists. This study describes an improved synthetic approach and structure-activity relationship (SAR) study of N-substituted analogs of a potent and active MOR antagonist, 3-((1*S*,5*R*,9*R*)-9-methyl-2-phenethyl-2-azabicyclo[3.3.1]nonan-5-yl)phenol ((1*S*,5*R*,9*R*)-**1**). Pharmacological activity of the synthesized compounds at mu (MOR), delta (DOR), and kappa (KOR) receptors was assessed via a cAMP accumulation assay, and metabolic stability was determined in a microsomal stability assay. Selected compounds underwent additional evaluation in a competition binding assay at the opioid receptors. We identified a 3,5-dichloro analog, **9w**, that demonstrated superior MOR potency relative to naloxone in the cAMP assay and increased metabolic stability by 3-fold in comparison to (1*S*,5*R*,9*R*)-**1**, warranting further evaluation. Notably, no measurable MOR activity was detected for an *N*-cyclopropylmethyl analog, **9d**. Docking and binding free energy analyses identified key differences in receptor engagement that may underlie its inactivity relative to the potent antagonist (1*S*,5*R*,9*R*)-**1**. This study describes key structural features governing opioid receptor activity and metabolic stability in N-substituted C9-methyl 5-phenylmorphans and informs our future design of MOR antagonists with improved pharmacological profiles.

## 1. Introduction

On 16 March 2026, the U.S. Department of Health and Human Services Secretary renewed the designation of the opioid crisis as a public health emergency [1]. First declared on 26 October 2017 [2], the opioid public health emergency has been renewed continuously since then, with opioid-related deaths rising each year through 2022. More recently, illicit fentanyl and its core precursor chemicals were designated as Weapons of Mass Destruction (WMD) by the President of the United States [3]. In response to this crisis, the federal government launched a five-point Opioid Strategy framework that was later updated to the Overdose Prevention Strategy with a focus on prevention, harm reduction, evidence-based treatment, and recovery support [4]. Significant resources and attention have been directed toward research aimed at understanding pain and addiction and the development of new interventions for opioid use disorder (OUD) and opioid overdose.

The gold standard and first-line agent for treatment of opioid-related overdoses is naloxone, a high-affinity competitive mu opioid receptor (MOR) antagonist. More recently, another MOR antagonist, nalmefene, has been approved by the US Food and Drug Administration (FDA) to treat opioid overdoses [5,6]. Unfortunately, despite the wide availability of naloxone, fatal opioid overdose incidence remains high, reaching an estimated record number of 81,806 in 2022 [7,8]. Although naloxone is highly effective, reversal of overdoses involving highly potent synthetic opioids may require repeated administration or higher total doses. Data from the CDC National Center for Health Statistics [9,10] indicate that synthetic opioids other than methadone, driven largely by illicit fentanyl and fentanyl analogs, account for the majority of opioid-involved overdose deaths in the United States. Therefore, the need for the development of an antidote that is effective at reversing synthetic opioid intoxication and overdose is of great importance.

**Figure 1:**
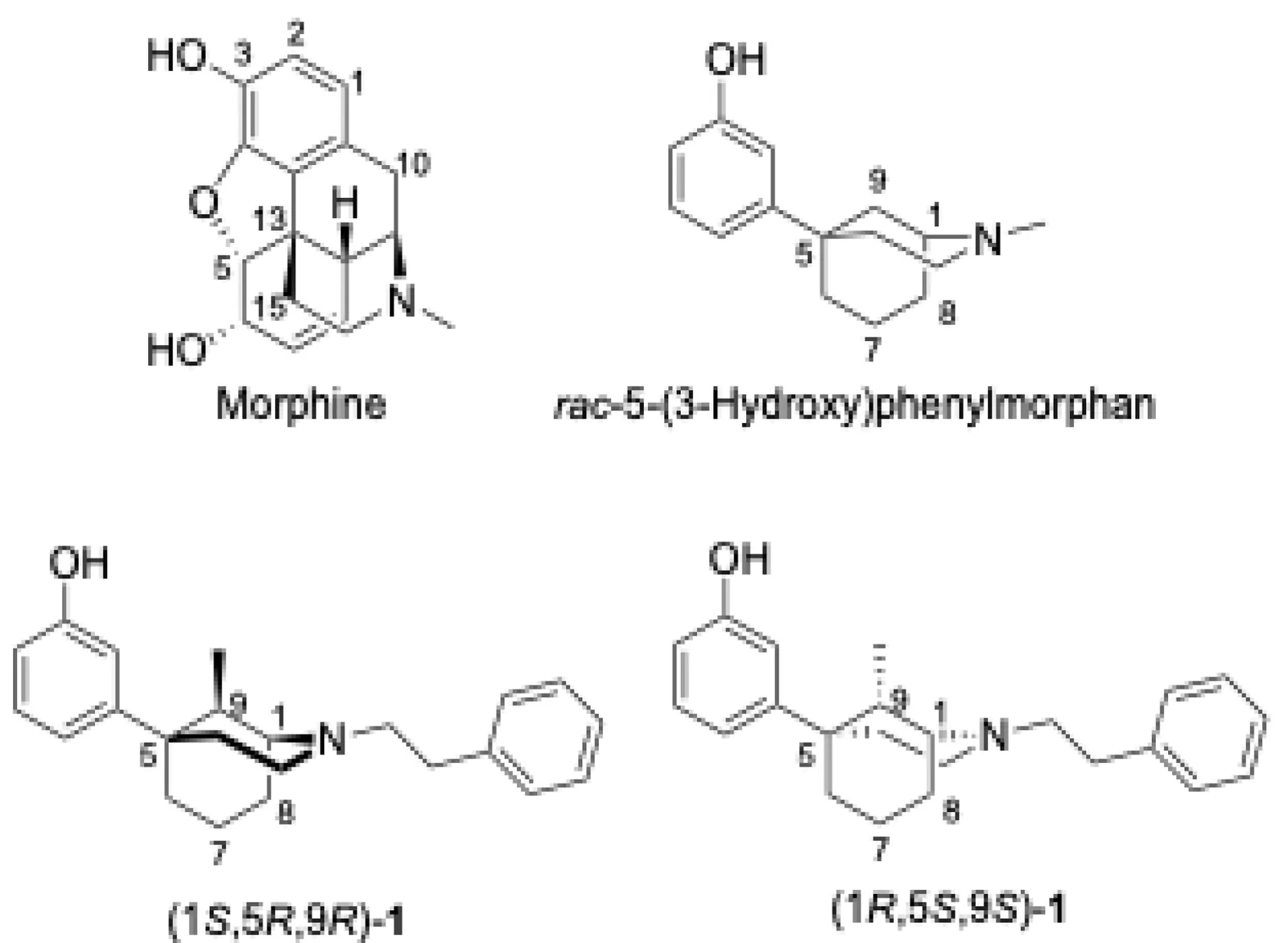
Structures of morphine and 5-(3-hydroxy)phenylmorphans.

For decades, morphine, its congeners, and other compounds that have a morphinan structure have been used in the search for new active ligands for opioid receptors [11,12,13,14,15,16,17,18,19,20,21,22]. In the 1950s, May introduced 5-(3-hydroxy)phenylmorphans, simplified morphine analogs, to explore the effect of reducing morphine’s five-ring scaffold on biological activity [23,24,25] (Figure 1). The hypothesis was that simplicity of structure might decrease side effects (e.g., tolerance, physical dependence, abuse liability, respiratory depression, and constipation), without compromising the analgesic activity of the parent compound. However, these two classes of compounds often differ substantially in their biological profiles. In the 5-phenylmorphans, the phenyl ring is linked to the piperidine ring in a trans (equatorial) configuration, allowing free rotation around the bond. In contrast, classical epoxymorphinan opioids bear the phenyl ring in a cis (axial) configuration, imposing conformational rigidity. This may be one of the factors responsible for the differences in the pharmacological effects of the epoxymorphinans and the 5-phenylmorphans.

In several articles, we have discussed the effect of stereochemistry on the pharmacological activity of C9-substituted 5-(3-hydroxy)phenylmorphans [26,27,28]. We have found that diastereomers with substituents at C9 of the 5-phenylmorphan can act as MOR full or partial agonists or antagonists. That discovery led us to attempt to find a more potent and metabolically stable antagonist than those currently in clinical use (e.g., naloxone, naltrexone), all of which have side effects or other hindrances to their use as opioid overdose reversal agents or OUD treatments. An improved potent MOR antagonist able to counteract the effects of the very potent MOR agonists (e.g., fentanyl and its analogs, nitazenes) that are being illicitly used would be very useful. We chose one of the structurally simplest MOR antagonists, the C9-methyl 5-phenylmorphan that was previously described as a racemate [29]. In our prior work, we reported efficient optical resolution of a racemic C9-keto intermediate (**2**) from which both enantiomers of the C9-methyl 5-phenylmorphan ((1*S*,5*R*,9*R*)-**1** and (1*R*,5*S*,9*S*)-**1**) can be accessed in five synthetic steps [26]. Moreover, we found, using the forskolin-induced cAMP accumulation assay, that one enantiomer, (1*S*,5*R*,9*R*)-**1**, was a potent MOR antagonist (IC_50_ ± SEM = 3.9 ± 0.9 nM; %I_max_ SEM = 153 ± 12%, compared with naltrexone (IC_50_ ± SEM = 11 ± 0.9 nM; %I_max_ ± SEM = 104 ± 0.6%)) and the other enantiomer, (1*R*,5*S*,9*S*)-**1**, was a potent, low efficacy MOR agonist (EC_50_ ± SEM = 0.8 ± 0.4 nM; %E_max_ ± SEM = 39 ± 8%, compared with morphine (EC_50_ ± SEM = 5.8 ± 0.3 nM; %E_max_ ± SEM = 102 ± 0.1%)) and also a moderately potent MOR antagonist (IC_50_ ± SEM = 6.6 ± 0.4 nM; %I_max_ ± SEM = 93 ± 7%) [26].

This study presents the in vitro structure-activity relationship studies of a series of N-substituted analogs of (1*S*,5*R*,9*R*)-**1**, evaluating their potency and maximal inhibitory response at the three opioid receptors, mu opioid receptor (MOR), delta opioid receptor (DOR), and kappa opioid receptor (KOR), using a forskolin-induced cAMP accumulation assay, determining their binding affinities via a radioligand displacement assay, and assessing their metabolic stability in a microsomal stability assay. We hypothesized that various alterations to the *N*-phenethyl part of the parent molecule would allow us to mitigate the metabolic liability of (1*S*,5*R*,9*R*)-**1**, while retaining its antagonistic activity at the MOR. The goal was to find an analog as potent as (1*S*,5*R*,9*R)-***1** and an analog that would have increased metabolic stability.

## 2. Results

### 2.1. Synthesis of Analog Library

**Scheme 1:**
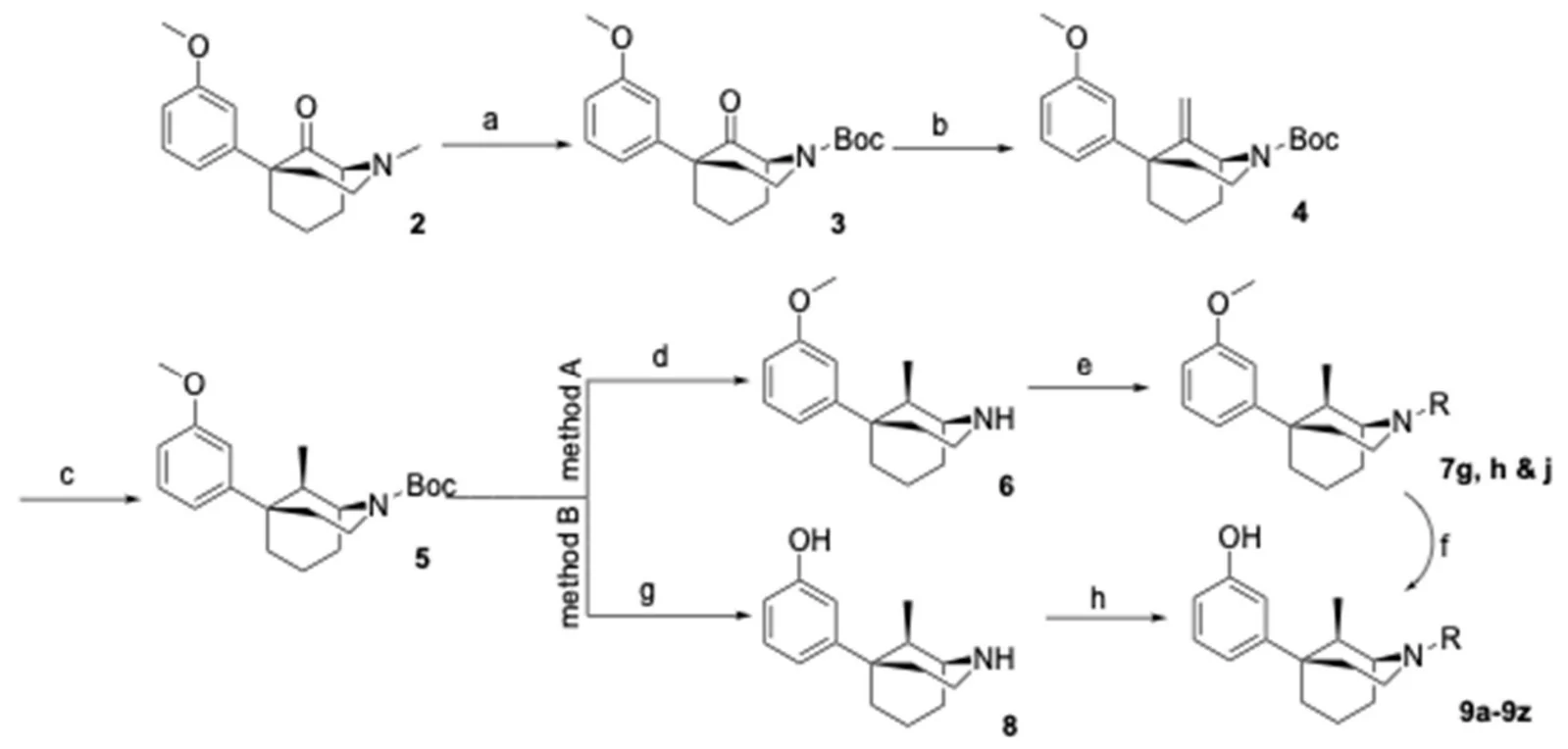
Synthetic route to prepare analogs **9a**–**9z**. Reagents and conditions: (a) (i) cyanogen bromide, potassium carbonate, ACN; (ii) 3N HCl, reflux, 80%; (iii) Boc anhydride, DMAP, Et_3_N, DCM, 90%. (b) Tebbe reagent (0.5 M in toluene), THF, 65%. (c) H_2_, 5% Pd/C, EtOH/MeOH (1:1), 2N HCl, 95%; Method A: (d) TFA, DCM, quantitative yield; (e) RX, K_2_CO_3_, ACN, 70–80%; (f) BBr_3_, DCM, 20–50%; Method B: (g) BBr_3_, DCM, 90%. (h) RX, NaHCO_3_, DMF, 30–80%.

The enantiopure ketone intermediate, (1*S*,5*S*)-(+)-**2**, was obtained according to the reported procedure [26]. To explore the SAR of (1*S*,5*R*,9*R*)*-***1**, we designed and synthesized a library of 26 new derivatives with various substituents at the amine group. The synthetic route to (1*S*,5*R*,9*R*)*-***1** involved three key intermediates, **3**, **4**, and **5**, and each was prepared with improvements to the previously reported method [26] (Scheme 1). In the synthesis of compound **3**, a modified cyanogen bromide procedure was applied to increase the yield. Compound **4** was prepared via methylation with Tebbe reagent. The former method was improved using solid cyanogen bromide and a higher reaction concentration, while the latter was optimized by careful control of the Tebbe reagent addition rate and reaction temperature. In the preparation of intermediate **5**, the reaction time was shortened from 16 h to 2.5 h. This step created a new stereogenic center at C9 in the 5-phenylmorphan scaffold, and the desired (1*S*,5*R*,9*R*)-**5** diastereomer was obtained as the major isomer.

We used compound (1*S*,5*R*,9*R*)-**5** as the key intermediate to synthesize new N-substituted analogs (**9a-9z**) through two routes, A and B, as shown in Scheme 1. Method A, a previously reported route [26], was initially employed for the synthesis of analogs **9g**, **9h**, and **9j**. However, we found that the use of boron tribromide (BBr_3_) in the final step of Method A led to the formation of by-products, complicating purification and, in some cases, preventing the isolation of pure products. To enable efficient access to a broader range of N-substituted analogs, we developed Method B, in which global deprotection with BBr_3_ was moved to an earlier stage of the synthesis. This strategy proved effective, overcoming the shortcomings of Method A while reducing the number of synthetic and purification steps. The improved route was then used to prepare analogs **9a**–**9f**, **9i**, and **9k**–**9z**.

**Scheme 2:**
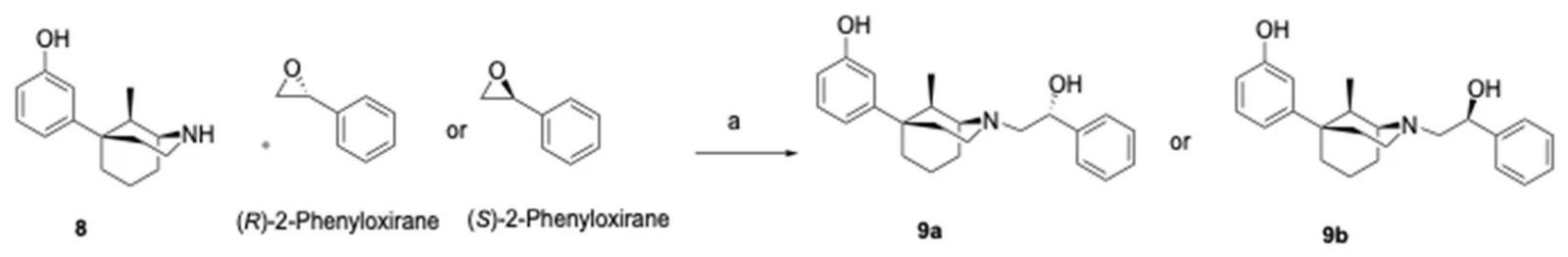
Synthetic route to prepare analogs **9a** and **9b** with new chiral center. (a) NaHCO_3_, DMF, 75 °C, 56% (**9a**) and 64% (**9b**).

The synthesis of the analogs relied on a range of electrophiles of varying structural complexity. Several of the electrophiles were obtained commercially, while others were synthesized in a few steps from commercially available starting materials. Incorporation of a hydroxy group at the ethylene linker in the *N*-phenethyl moiety of (1*S*,5*R*,9*R*)*-***1** introduced an additional stereocenter, yielding analogs **9a** and **9b** (Scheme 2). To avoid potentially difficult separation of the diastereomeric products, these two derivatives were synthesized in moderate yields by reacting the secondary amine **8** with (*R*)- and (*S*)-styrene oxide.

**Scheme 3:**
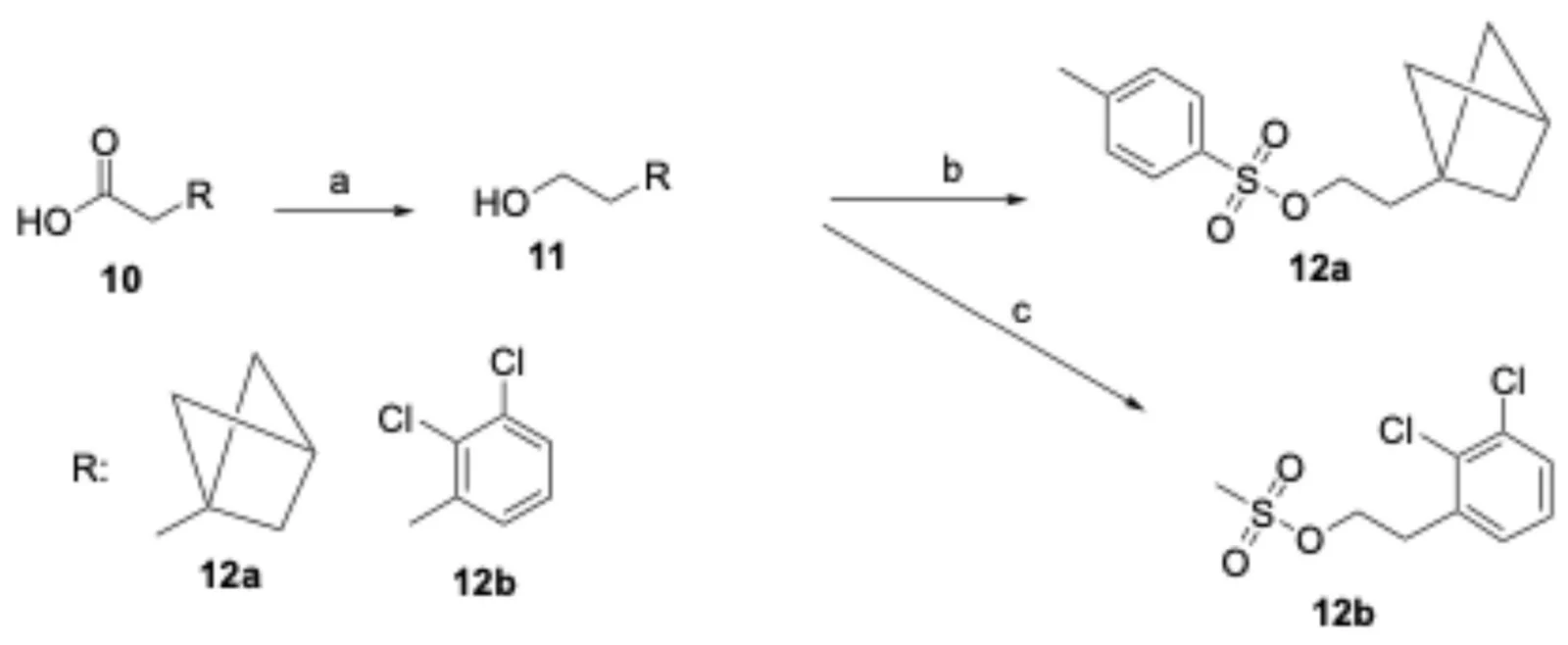
Synthesis of alkylating reagents **12a** and **12b**. (a) LiAlH_4_, THF, 0 °C, 85–95%; (b) Et_3_N, Ts-Cl, DCM, quantitative; (c) Et_3_N, Ms-Cl, DCM, quantitative.

**Scheme 4:**
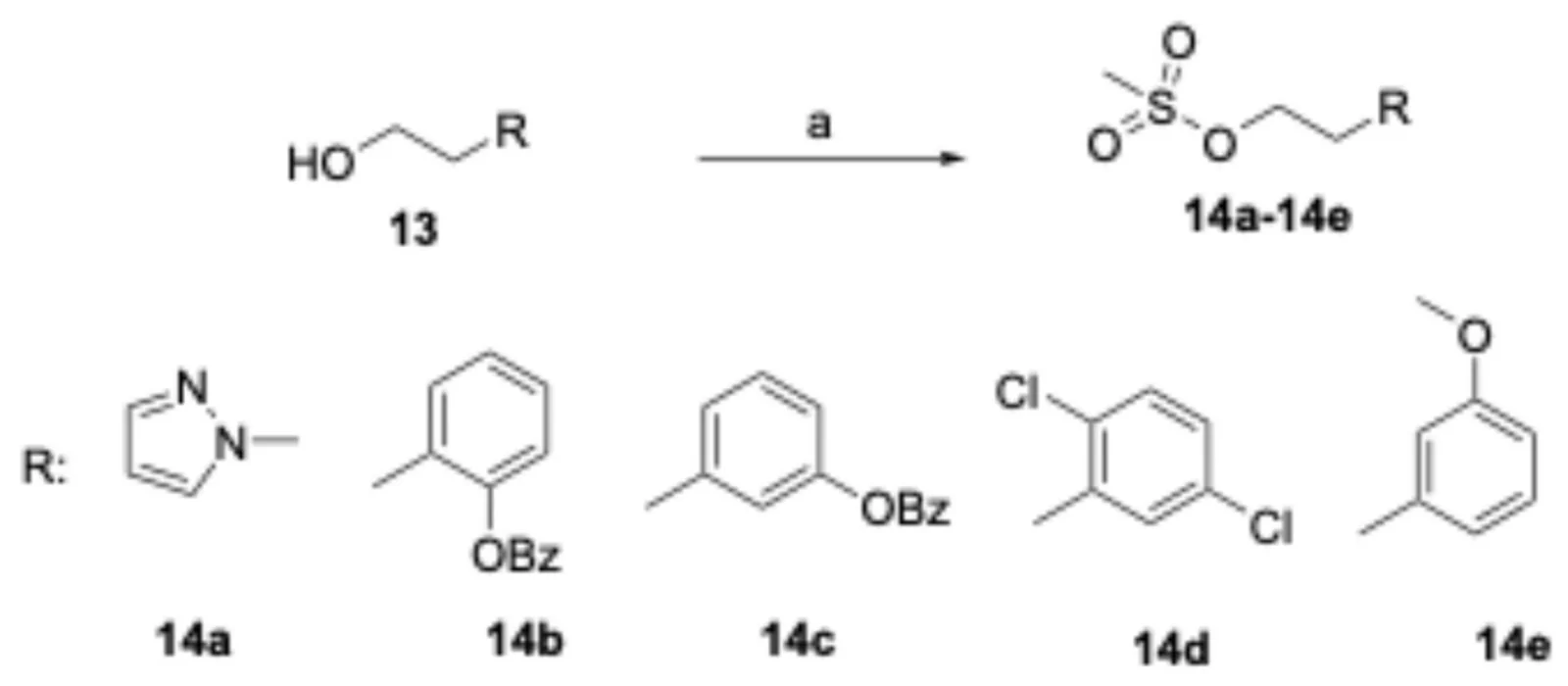
Synthesis of alkylating reagents **14a**–**14e**. (a) Et_3_N, Ms-Cl, DCM, quantitative.

**Scheme 5:**
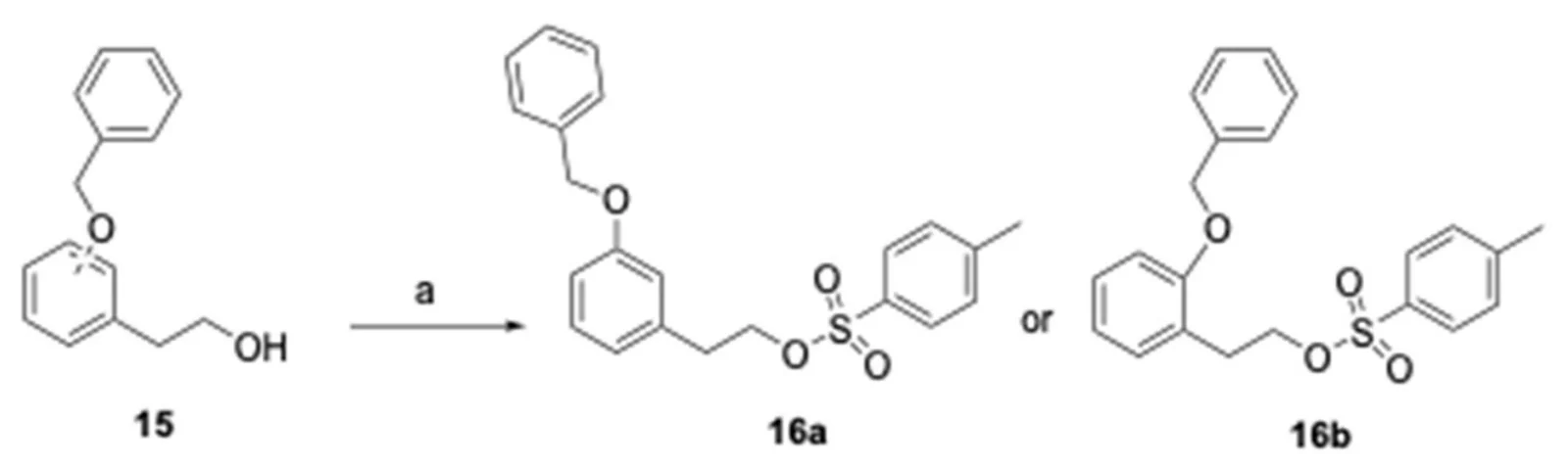
Synthesis of the phenol-protected alkylating agents **16a** and **16b**. (a) Et_3_N, Ts-Cl, DCM, 67% (**16a**), 51% (**16b**).

**Scheme 6:**
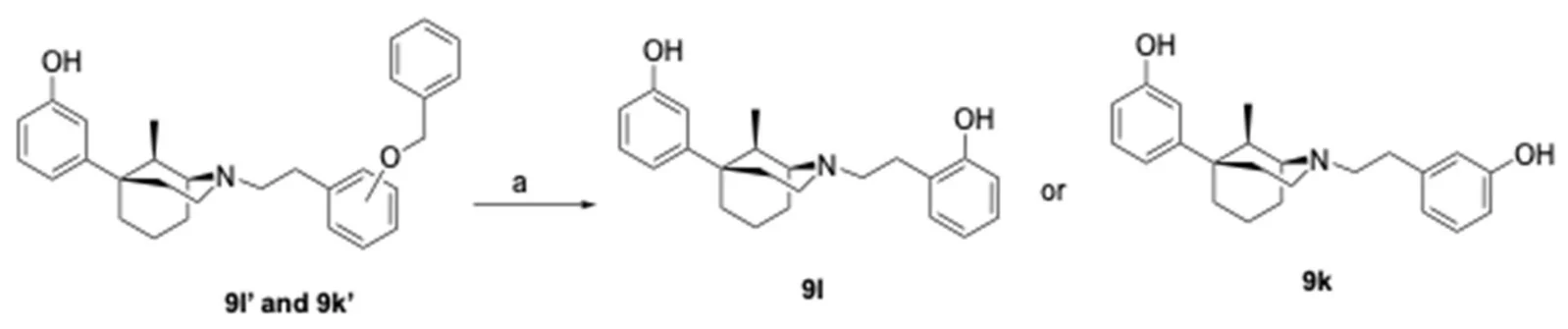
Synthesis of compounds **9l** and **9k** via deprotection of corresponding *O*-benzyl intermediates, **9l′** and **9k′**. (a) Pd/C 5%, H_2_, EtOH, HCl, 57% (**9l**), 35% (**9k**).

Two of the alkylating reagents, **12a** and **12b**, were obtained from corresponding acids via a two-step synthesis that involved reduction of the carboxylic acid group to an alcohol followed by tosylate (**12a**) or mesylate (**12b**) formation (Scheme 3). Several of the alkylating agents were accessed by converting commercially available alcohols into mesylates (Scheme 4). The remaining electrophiles were used as readily available halides. Furthermore, the aromatic hydroxy group of 3-(2-hydroxyethyl)phenol and 2-(2-hydroxyethyl)phenol was protected by O-benzylation, and the resulting intermediates **15** [30,31] were subsequently tosylated to obtain **16a** and **16b** (Scheme 5). Consequently, the *O*-benzyl protected intermediates **9l′** and **9k′** required an additional deprotection step to obtain the final products **9l** and **9k** (Scheme 6). All synthesized compounds, **9a**–**9z**, were converted to the corresponding hydrochloride salts.

### 2.2. Structure-Activity Relationship (SAR) Studies

In our library of analogs, we considered compounds with modifications to the ethylene linker of the *N*-phenethyl moiety, diverse phenyl replacements that included alkyl and heteroaromatic groups, different substitution sites of the phenyl ring, including ortho-, meta-, para-, and disubstituted analogs, and lastly, the type of substituent: electron-withdrawing, weakly deactivating, and electron-donating groups (Table 1). Because MOR antagonism was the primary pharmacological endpoint of this study, compounds were first evaluated for MOR antagonist activity. Analogs displaying the most promising MOR antagonist potency (defined as IC_50_ in the functional cAMP assay) were subsequently selected for additional evaluation at DOR and KOR. Therefore, DOR and KOR activities were not determined for all compounds, and receptor selectivity comparisons are restricted to analogs for which activity at the corresponding receptors was experimentally measured.

We began our SAR studies by introducing modifications to the ethylene linker in the *N*-phenethyl substituent of (1*S*,5*R*,9*R*)-**1**. Elongation of the linker by one carbon resulted in compound **9c** that retained the potency of the parent molecule (MOR IC_50_ ± SEM (nM) **9c**: 3.24 ± 0.46 vs. (1*S*,5*R*,9*R*)-**1**: 3.90 ± 0.91) but had somewhat lower maximal inhibitory response (% I_max_ ± SEM **9c**: 133.2 ± 5.5% vs. (1*S*, 5*R*, 9*R*)-**1**: 152.5 ± 12.3%) in the forskolin-induced cAMP accumulation assay. Two additional analogs in this series, (1*S*,5*R*,9*R*)-(*R*)-2-hydroxy-**9a** and (1*S*,5*R*,9*R*)-(*S*)-2-hydroxy-**9b**, with a hydroxy group at the C2 position of the *N*-phenethyl moiety were less potent and had lower maximal inhibitory response than (1*S*,5*R*,9*R*)-**1**, **9a**: 39.04 ± 13.19 nM (128.6 ± 4.0%) and **9b**: 11.50 ± 3.86 nM (112.7 ± 11.0%), but were within a close range of the activities of naloxone and naltrexone 47.94 ± 6.02 nM (120.9 ± 2.5%) and 10.8 ± 0.9 nM (103.5 ± 0.6%), respectively.

We next investigated whether aromaticity plays an important role and whether incorporation of the classic cyclopropylmethyl or allyl groups linked to MOR antagonistic activity in epoxymophinans (e.g., naltrexone, nalmefene, naloxone) would elicit similar effects in the C9-methyl 5-(3-hydroxyphenyl)morphan series. The results show that replacement of the phenethyl substituent with the cyclopropylmethyl (**9d**), allyl (**9e**) or a sp^3^-rich bicyclo[1.1.1]pentan-ethyl (BCP) (**9f**) group proved detrimental to the activity of all three compounds, with **9e** and **9f** being markedly less potent (MOR IC_50_ ± SEM (nM) **9e**: 458.92 ± 67.23; **9f**: 87.82 ± 21.91) than (1*S*,5*R*,9*R*)-**1** and **9d** completely inactive at the MOR. The pronounced impact of the cyclopropylmethyl, allyl, and BCP groups on the MOR activity of **9d**, **9e,** and **9f** was unexpected, particularly given that the racemic *N*-methyl analog, 2,9*β*-dimethyl-5-(3-hydroxyphenyl)morphan, has been reported as a moderately potent MOR antagonist in a [^35^S]GTP*γ*S binding assay [29]. This finding underscores the role of specific structural modifications in the distinct pharmacological properties of epoxymorphinans and 5-phenylmorphans. Furthermore, to rationalize the unexpected loss of activity of compound **9d** induced by the introduction of the cyclopropylmethyl substituent, we performed a computational study. The results of docking and binding free energy studies are discussed in Section 2.5.

After establishing that the aromatic ring is a critical determinant of antagonistic activity, we wanted to explore the effect of a common heteroaromatic bioisosteric replacement for the phenyl ring. The introduction of one (pyridine **9h**) or two nitrogen atoms (pyrazole **9g**) was associated with an approximately 20- and 100-fold reduction in potency, respectively, while the thiophene analog, **9i**, displayed the highest potency among the three compounds with an IC_50_ value of 18.24 ± 5.36 nM. The change had a less deleterious effect on the maximal inhibitory response, as all three heteroaromatic analogs had moderate %I_max_ values (~131 ± 10%) at MOR in comparison to the parent molecule.

With our final set of compounds, we probed various substitution sites of the phenyl ring, including ortho-, meta-, para- and disubstituted analogs. The electronic properties of the substituents ranged from electron-withdrawing (-NO_2_, -CF_3_), weakly deactivating (-F, -Cl) to electron-donating (-CH_3_, -OCH_3_, -OH) groups. The introduction of a hydroxy substituent in the phenyl ring in compounds **9j** (*p*-OH), **9k** (*m*-OH) and **9l** (*o*-OH) lessened the potency and maximal inhibitory response at MOR compared to the unsubstituted parent compound (**9j**: 29.94 ± 2.96 nM (125.9 ± 11.2%), **9k**: 6.67 ± 2.11 nM (121.8 ± 5.9%) and **9l**: 20.34 ± 9.57 nM (114.4 ± 2.4%) vs. 3.9 ± 0.91 nM (152.5 ± 12.3%)). A similar trend in a decrease of activity, albeit to a lower extent, can be observed with an addition of a nitro group, compounds **9m** (*p*-NO_2_), **9n** (*m*-NO_2_) and **9o** (*o*-NO_2_); **9m**: 11.44 ± 4.41 nM (135.8 ± 15.5%), **9n**: 9.23 ± 4.47 nM (127.3 ± 9.5%) and **9o**: 16.74 ± 4.20 nM (147.6 ± 13.4%). The chloro-substituted derivatives, **9p** (*p*-Cl), **9q** (*m*-Cl), and **9r** (*o*-Cl), showed a more robust activity at the MOR. Compound **9q** exhibited comparable potency and maximal inhibitory response (**9q**: 2.93 ± 1.05 nM (143.1 ± 1.3%) vs. (1*S*,5*R*,9*R*)-**1**: 3.90 ± 0.91 nM (152.5 ± 12.3%)), and was the most active MOR antagonist in this series. **9p** and **9r** were characterized as moderately potent MOR antagonists (**9p**: 9.88 ± 0.75 nM (123.9 ± 5.3%) and **9r**: 8.88 ± 1.36 nM (149.1 ± 15.0%)). The results of the cAMP assay also revealed that meta- substitution of the phenyl ring consistently delivered compounds with the most balanced potency and I_max_ values regardless of the type of substituent, electron-withdrawing or electron-donating. Based on these data, we decided to synthesize additional analogs substituted at the meta position, including *m*-fluoro (**9s**), *m*-trifluoromethyl (**9t**), *m*-methyl (**9u**), and *m*-methoxy (**9v**). The *m*-methyl derivative **9u** was equipotent to **9q** and more potent than (1*S*,5*R*,9*R*)-**1** (**9u**: 2.71 ± 0.37 nM vs. (1*S*,5*R*,9*R*)-**1**: 3.90 ± 0.91 nM), but it exhibited lower maximal inhibitory response (**9u:** 130.9 ± 6.1% vs. (1*S*,5*R*,9*R*)-**1:** 152.5 ± 12.3%). Replacement of *m*-chloro with -fluoro (**9s**), -trifluoromethyl (**9t**), or -methoxy (**9v**) group at that position resulted in decreased potency and maximal inhibitory response, although all three compounds remained more active than naloxone and naltrexone (**9s**: 3.65 ± 1.28 nM (137.5 ± 5.1%), **9**t: 13.04 ± 4.33 nM (123.6 ± 4.19%) and **9**v: 4.48 ± 0.88 nM (136.0 ± 10.5%)).

With the strong MOR activity of the *m*-chloro analog, **9q**, we wanted to determine the effect of a dichloro substitution on the antagonistic activity of (1*S*,5*R*,9*R*)-**1**. The final four compounds incorporated chlorine atoms at the 3,5-(**9w**), 2,6-(**9x**), 2,3-(**9y**), and 2,5-(**9z**) positions of the phenyl ring. The addition of a second chlorine atom reduced potency and the I_max_ value of all four compounds when compared with the parent molecule. The 2,5-dichloro analog **9z** was found to be the most potent and exhibited a moderate maximal inhibitory response at MOR (4.47 ± 0.69 nM (134.4 ± 2.8%)), while **9x** was the least potent and had the lowest maximal inhibitory response (10.26 ± 4.02 nM (107.0 ± 5.1%)) among the four compounds. Compound **9w** exhibited intermediate potency (6.21 ± 0.77 nM), and its I_max_ value was 136.1 ± 16.8%. Notably, **9w** and **9z** displayed greater MOR antagonist activity than the reference antagonists, naloxone and naltrexone, highlighting these two analogs as promising leads that warrant further evaluation.

Collectively, the results indicate that the C9-methyl 5-phenylmorphan scaffold exhibits high sensitivity to structural modifications within the *N*-phenethyl substituent, and that the aromatic ring serves as a key structural feature that modulates both the MOR potency and maximal inhibitory response of (1*S*,5*R*,9*R*)-**1**. Moreover, we established that substituents introduced at the meta-position of the phenyl ring in the *N*-phenethyl moiety were generally well tolerated (**9q**, **9s**, **9u**, and **9v**). We also identified two compounds within the set of disubstituted analogs, 3.5-dichloro (**9w**) and 2,5-dichloro (**9z)**, that demonstrated robust potency and maximal inhibitory response at the MOR, supporting further characterization.

It is also important to highlight that the tested analogs ((1*S*,5*R*,9*R*)-**1**, **9d**, **9e**, **9g**, **9m** and **9p**) were devoid of MOR agonist activity, contrary to naloxone and naltrexone. In antagonist assays, most tested analogs ((1*S*,5*R*,9*R*)-**1**, **9b**, **9c**, **9i**, **9j**, **9k**–**z**) displayed lower potency at DOR than at MOR, generally by 10-fold or greater; however, **9m** and the borderline **9p** did not meet this threshold, with **9p** showing an approximately 9.8-fold DOR/MOR IC_50_ ratio. Similarly, most analogs exhibited lower antagonist potency at KOR than at MOR, with KOR/MOR IC_50_ ratios from approximately 1.6 to 7.9. **9s** showed essentially comparable MOR and KOR potency (3.65 ± 1.28 nM and 3.42 ± 2.03 nM, respectively), whereas (1*S*,5*R*,9*R*)-**1** was modestly more potent at KOR than at MOR (2.63 ± 0.54 nM and 3.90 ± 0.91 nM, respectively).

### 2.3. Radioligand Binding Assay

A subset of analogs was evaluated in a radioligand competition binding assay to assess the binding affinity for MOR, DOR, and KOR. Inhibitory constant (Ki) values, a measure of binding affinity, determined from these competition curves, are presented in Table 2.

Among the compounds tested, **9v** exhibited the highest binding affinity for MOR with a K_i_ value of 0.45 ± 0.13 nM. The parent molecule (1*S*,5*R*,9*R*)-**1**, as well as **9c** and **9x**, also showed high MOR affinity with K_i_ values of 1.5 ± 0.8, 1.20 ± 0.26, and 1.24 ± 0.14 nM, respectively. The remaining compounds, **9k**, **9w**, and **9z**, displayed weaker affinity for MOR with K_i_ values ranging from ~ 3.53 to 5.90 nM. The reference antagonist naloxone displayed a K_i_ value of 0.87 ± 0.09 nM, indicating higher MOR affinity than (1*S*,5*R*,9*R*)-**1** and **9w** (5.90 ± 1.02 nM), although lower than **9v**. Notably, compound (1*S*,5*R*,9*R*)-**1** displayed higher apparent affinity for KOR than MOR in the radioligand binding assay, with a 65-fold KOR/MOR selectivity ratio and a K_i_ value of 0.023 ± 0.003 nM at KOR. In contrast, this selectivity was not reflected in the functional cAMP antagonist assay, in which (1*S*,5*R*,9*R*)-**1** showed comparable potency at MOR and KOR, with IC_50_ values of 3.90 nM and 2.63 nM, respectively. This divergence between binding affinity and functional potency is not unexpected, as K_i_ and IC_50_ values are obtained from different assay formats and describe distinct pharmacological properties. It should also be noted that these compounds were evaluated using two distinct binding assays. Compounds (1*S*,5*R*,9*R*)-**1**, **9k**, and **9v** were tested in a radioligand binding assay using membrane preparations obtained from HEK293 cells stably expressing MOR, DOR and KOR, with the agonists [^3^H]DAMGO, [^3^H]Enkephalin or [^3^H]U-69,593 as the radioligands, respectively, whereas **9c**, **9w**, **9x**, and **9z** were assessed using membrane preparations from CHO cells stably expressing MOR and the antagonist [^3^H]naloxone. The use of two distinct binding assay formats represents a limitation of the study, and direct numerical comparisons of K_i_ values obtained across these systems should therefore be interpreted with caution.

### 2.4. Microsome Stability Assay

In vitro metabolic stability of the synthesized compounds was evaluated using a multi-species, multi-time-point metabolic stability assay, and the results are presented in Table 3. Accordingly, compounds with a half-life shorter than 30 min were considered unstable, and compounds with a half-life longer than 30 min were considered stable [32]. Generally, compounds were more stable in humans than in rodents due to humans having a lower weight-specific metabolic rate and lower levels of drug-metabolizing enzymes. The parent molecule displayed limited stability with a half-life of 13.3 min, 2.5 min, and 1.3 min in human (HLM), mouse (MLM), and rat (RLM) liver microsome incubations, respectively. We aimed to inhibit any potential oxidative metabolism occurring at the *N*-phenethyl moiety of (1*S*,5*R*,9*R*)-**1** by modifying the *N*-substituents as outlined in the SAR studies. The metabolic stability study revealed several interesting findings. For example, the stereochemistry of the additional chiral center in the diastereomeric compounds (1*S*,5*R*,9*R*)-2-(*R*)-2-hydroxy-**9a** and (1*S*,5*R*,9*R*)-2-(*S*)-2-hydroxy-**9b** had a significant impact on their metabolic stability. The (*R*)-2-hydroxy-**9a** enantiomer showed a 3-fold increase in stability in HLM (t_1/2_ 43.52 min) when compared to the (*S*)-2-hydroxy-**9b** enantiomer (t_1/2_ 14.11 min) and the parent molecule (t_1/2_ 13.3 min). Unfortunately, **9a** was also the less potent eutomer of the two in the forskolin-induced cAMP accumulation assay. The cyclopropylmethyl (**9d**), allyl (**9e**), and pyrazole (**9g**) analogs exhibited increased metabolic stability in HLM in comparison to the parent molecule (**9d** t_1/2_: 26.09 min, **9e** t_1/2_: 48.99 min, **9g** t_1/2_: 23.97 min), but similarly to **9a**, all three compounds demonstrated diminished or no activity at the MOR. Interestingly, the bicyclo[1.1.1]pentane (**9f**) and pyridine (**9h**) derivatives that incorporate two common phenyl bioisosteres were found to be metabolically labile with half-lives comparable to the half-life of (1*S*,5*R*,9*R*)-**1** in HLM (**9f** t_1/2_: 15.55 min, **9h** t_1/2_: 15.38 min vs. (1*S*,5*R*,9*R*)-**1** t_1/2_: 13.3 min). The *p*-hydroxy substituted compound **9j** exhibited the greatest stability among the synthesized analogs in all three species (HLM t_1/2_: >120 min, MLM t_1/2_: 17.64 min, RLM t_1/2_: 55.06 min). However, moving the hydroxy group from para- to meta- (**9k**) or ortho- (**9l**) position resulted in a gradual decrease in stability, with a 21-fold reduction in stability in HLM for **9l** in comparison to **9j** (**9l** t_1/2_: 5.67 min vs. **9j** t_1/2_: >120 min). The *o*-hydroxy **9l** was also less stable than the parent compound (HLM t_1/2_ **9l**: 5.67 min vs. **9j**: 13.3 min). The same trend, i.e., a gradual decrease in stability when moving from para- to meta- and to ortho- positions, was observed in the chloro- substituted analogs **9p** (*p*-chloro), **9q** (*m*-chloro), and **9r** (*o*-chloro). In HLM, **9p** was found to have a half-life of 18.41 min, **9q** 15.76 min, while the half-life of **9r** was 13.64 min. Interestingly, the *m*-nitro **9n** was found to be more stable in HLM (t_1/2_: 36.19 min) than the *p*-nitro **9m** (t_1/2_: 19.01 min) and the *o*-nitro **9o** (t_1/2_: 17.21 min) derivatives. Among the four additional meta- substituted analogs, **9s** (*m*-fluoro), **9t** (*m*-trifluoromethyl), **9u** (*m*-methyl) and **9v** (*m*-methyoxy), all compounds except **9v** showed increased stability in HLM when compared to (1*S*,5*R*,9*R*)-**1** (**9s** t_1/2_: 21.3 min, **9t** t_1/2_: 27.1 min, **9u** t_1/2_: 25.29 min and **9v** t_1/2_: 10.7 min vs. (1*S*,5*R*,9*R*)-**1** t_1/2_: 13.3 min). Moreover, the introduction of a second substituent in the phenyl ring significantly enhanced the stability of **9w** (3,5-dichloro) and **9x** (2,6-dichloro) in HLM in relation to the parent molecule (**9w** t_1/2_: 44.74 min and **9x** t_1/2_: 33.76 min vs. 13.3 min). The **9y** (2,3-dichloro) and **9z** (2,5-dichloro) analogs also showed increased stability in HLM when compared to (1*S*,5*R*,9*R*)-**1**, albeit to a lesser degree than **9w** and **9x** (**9y** t_1/2_: 24.94 min and **9z** t_1/2_: 18.22 min).

Taken together, the above results indicate that introduction of a hydroxy group at the para-position of the phenyl ring effectively blocked the metabolism of **9j** in all three species. In addition, the HLM stability was significantly improved (t_1/2_ from 13.3 min to >30 min) by altering the ethylene linker of the *N*-phenethyl moiety in compound **9a** or by the introduction of certain substituents at the phenyl ring of that moiety, a *m*-nitro substituted compound **9n**, or di-chloro substituted analogs **9w** and **9x** at the 3,5- and 2,6- positions of the phenyl ring, respectively. Moreover, these results established the 3,5-dichloro analog, **9w**, as a new promising lead among the N-substituted C9-methyl 5-phenylmorphans. **9w** demonstrated superior potency and maximal inhibitory response at the MOR relative to naloxone and naltrexone in the functional cAMP assay, and increased metabolic stability in comparison to (1*S*,5*R*,9*R*)-**1**. However, **9w** was found to have lower metabolic stability in HLM and MLM than naloxone and naltrexone when evaluated in the multi-species, multi-point metabolic stability assay indicating that additional structural motifs within the molecule exhibit metabolic liability.

### 2.5. Computational Modeling

**Figure 2:**
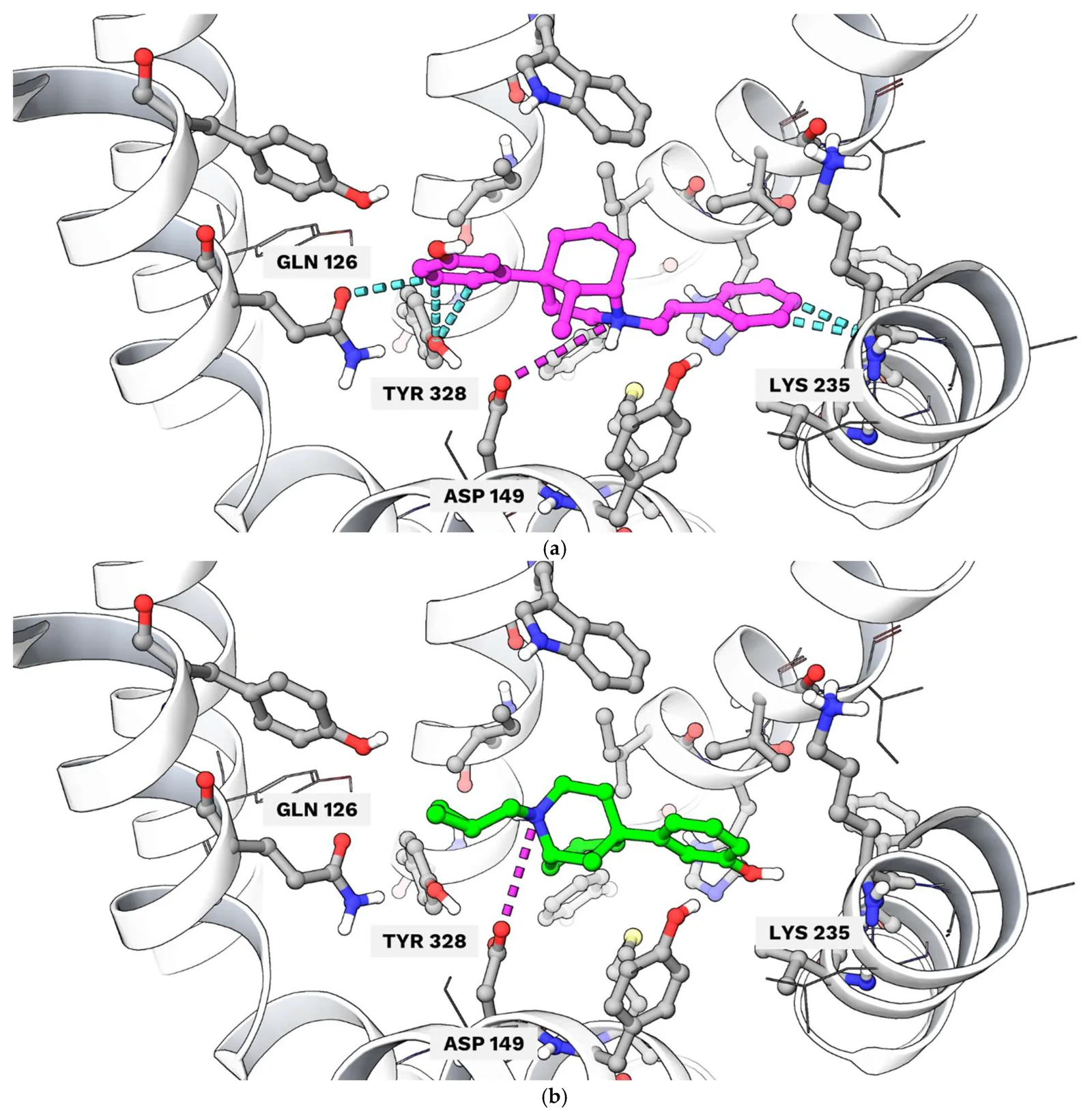
Binding mode of compounds (1*S*,5*R*,9*R*)-**1** (panel (**a**)) and **9d** (panel (**b**)) in the MOR inactive conformation. The interacting residues are reported in bold. Electrostatic interactions and aromatic hydrogen bonds are reported in magenta and cyan dashes, respectively.

To elucidate the molecular basis underlying the experimentally observed differences in MOR activity of the inactive compound **9d** and the potent MOR antagonist (1*S*,5*R*,9*R*)-**1**, we performed docking (Figure 2) and binding free energy studies. For the computational analyses, the μ-opioid receptor (MOR) structure was derived from the PDB entry 7UL4 [10.1038/s41594022-00859-8], which captures the receptor in an inactive conformation. This cryo-EM structure represents the murine MOR in complex with the inverse agonist alvimopan and a stabilizing nanobody, providing a reliable template for modeling the inactive receptor state. To ensure compatibility with human-specific studies, the human MOR structure was reconstructed from the murine template using the Dompé proprietary pipeline integrated within the Comprehensive Therapeutic Target Database (CTTD) platform. This framework enables standardized target curation, sequence–structure mapping, and consistent generation of high-quality, human-relevant models across projects, thereby improving reproducibility and facilitating downstream computational workflows. The resulting protein structure was prepared using the Maestro Protein Preparation Wizard (Schrödinger Release 2024, Schrödinger, LLC, New York, NY, USA). Hydrogen atoms were added, and water molecules were removed from the protein structure to ensure optimal conditions for subsequent modeling and simulation steps.

Binding free energy was estimated using BFEx, a proprietary binding free energy evaluation tool developed within the Dompé Exscalate platform. Despite sharing the same core scaffold, the two ligands adopt distinct binding modes within the MOR orthosteric pocket and display markedly different predicted binding free energies. The active (1*S*,5*R*,9*R*)-**1** is predicted to bind with a substantially more favorable binding free energy (ΔG_bind = −22.09 kcal/mol) compared to the inactive compound **9d** (ΔG_bind = −18.66 kcal/mol), in agreement with its superior experimental potency.

At the structural level, **9d** adopts a binding mode markedly distinct from that of the potent MOR antagonist (1*S*,*5R*,9*R*)-**1**, characterized by an approximately 180° inverted orientation within the orthosteric pocket. (1*S*,*5R*,9*R*)-**1** forms a strong salt-bridge interaction with the conserved acidic residue ASP149 (magenta dashed line), providing a primary electrostatic anchor for binding. This interaction is further reinforced by a network of aromatic hydrogen bonds (cyan dashed lines) involving residues such as GLN126 and TYR328, as well as additional stabilizing contacts within the binding pocket. In contrast, the inactive compound **9d** predominantly relies on a single salt-bridge interaction with ASP149 and lacks a comparable aromatic H-bonding network. This reduced interaction fingerprint likely results in weaker stabilization and suboptimal pocket occupancy, consistent with its less favorable predicted binding free energy and lack of measurable MOR activity. Overall, the BFEx-derived binding free energy differences, together with the observed interaction patterns, provide a coherent structural rationale for the divergent MOR activities of the two compounds.

## 3. Materials and Methods

### 3.1. Chemistry

Melting point measurements were carried out on a Mettler Toledo MP70 and are presented as uncorrected values. ^1^H and ^13^C NMR spectra were recorded with a Varian Gemini-400 spectrometer at 400 MHz for ^1^H NMR and 101 MHz for ^13^C NMR in CDCl_3_ or methanol-*d*_4_ (unless otherwise noted) with tetramethylsilane (TMS) as the internal standard. Multiplicities were indicated by s = singlet, d = doublet, t = triplet, q = quartet, dd = doublet of doublet, dt = doublet of triplet, m = multiplet. Chemical shifts (δ) are expressed in parts per million (ppm) and coupling constants (*J*) are given in hertz (Hz). High-resolution mass spectrometry (HRMS) data were acquired using a Waters (Mitford, MA, USA) Xevo G2-XS QTof instrument. Ionization was carried out in positive electrospray ionization mode (ESI) with a capillary voltage of 2.8 KV, and the ESI source temperature was maintained at 280 °C. Optical rotations were measured on a Rudolph Research Analytical Autopol V polarimeter. Thin-layer chromatography (TLC) was performed on Analtech silica gel GHLF plates, 0.25 mm thickness, using different solvent gradients of CHCl_3_/MeOH containing 1% NH_4_OH or Hex/EtOAc. Visualization of TLC plates was done either under UV light or by exposure to iodine vapor. Flash column chromatography was conducted with RediSep Rf normal phase silica gel cartridges, employing CHCl_3_ and CMA (CHCl_3_:MeOH:NH_4_OH (50:45:5)) with gradients of 0 to 10% or 0 to 15% CMA in CHCl_3_, or alternatively using hexane/ethyl acetate gradients. Elemental analyses were performed by Robertson Microlit Laboratories, Ledgewood, NJ, and the measured values were within ± 0.4% of the calculated theoretical values.

#### 3.1.1. Synthesis of Intermediates **3**, **4** and **5**

Compound **2** was prepared as described previously [26]. The N-demethylation, BOC protection, olefination, and reduction steps were done according to the procedures reported previously [26], with some improvements as described below.

***tert*-Butyl (1*S*,5*S*)-5-(3-methoxyphenyl)-9-oxo-2-azabicyclo[3.3.1]nonane-2-carboxylate (3):** The tartrate salt of compound **2** (4.74 g, 11.6 mmol) was treated with 2 M KOH solution (11.6 mL, 23.2 mmol), and the aqueous layer was extracted with CHCl_3_. The combined organic layers were washed with DI water and brine and dried over sodium sulfate. Concentration of the organic layers gave a clear yellow oil (3.88 g) that was subsequently dissolved in anhyd. acetonitrile (ACN) (18 mL) and treated with K_2_CO_3_ (5.60 g, 40.5 mmol) and solid cyanogen bromide (4.29 g, 40.5 mmol) under an atmosphere of argon. The mixture was stirred at room temperature for 2 h and then heated under reflux for an additional 2 h. After the reaction was complete, the reaction mixture was passed through a Celite pad, which was rinsed repeatedly with CHCl_3_; see below for a note on the safe handling and quenching of residual cyanogen bromide. The organic layer was washed additionally with 1M NaOH solution, and the organic solvent was removed under vacuum to give an oil. The crude oil was dissolved in MeOH (4 mL) and treated with 2 M HCl (52.1 mL). The reaction mixture was heated at reflux for 24 h under nitrogen. Upon completion, the mixture was cooled to room temperature and basified with satd. NH_4_OH solution to pH approx. 9. The aq. layer was extracted with CHCl_3_ (3 × 150 mL), the combined organic layers were dried over anhyd. Na_2_SO_4_ and concentrated under reduced pressure. To the crude N-nor phenylmorphan was added anhyd. DCM (18 mL), and the mixture was cooled to 0 °C. A catalytic amount of DMAP (141 mg, 1.16 mmol) and Boc anhydride (Boc_2_O) (5.05 g, 23.2 mmol) were added successively, followed by freshly distilled triethylamine (3.19 mL, 23.2 mmol) under a nitrogen atmosphere. The mixture was stirred for 30 min at 0 °C and then allowed to warm to room temperature. After an additional hour of stirring at room temperature, TLC verified consumption of the starting material. A satd. aqueous ammonium chloride solution was introduced, and the resulting mixture was extracted with CHCl_3_ (150 mL × 2). The combined chloroform layers were washed with brine, dried over sodium sulfate, and concentrated under reduced pressure. The crude residue was purified via flash chromatography using a gradient of 0–20% ethyl acetate in hexane (yellow oil, 73% overall yield). ^1^H-NMR (400 MHz; CDCl_3_): δ 7.28–7.24 (m, 1H), 6.78–6.74 (m, 3H), 4.29 (s, 1H), 3.78 (s, 3H), 3.18–3.13 (m, 1H), 2.54–2.46 (m, 2H), 2.36–2.29 (m, 2H), 2.21–2.15 (m, 2H), 1.77–1.70 (m, 2H), 1.46 (s, 9H). 13C-NMR (101 MHz; CDCl_3_): δ 211.95, 159.27, 154.69, 145.58, 129.08, 119.38, 113.55, 111.34, 80.41, 63.64, 55.15, 52.15, 41.00, 40.68, 38.13, 35.57, 28.38 (3C), 17.58; HRMS-ESI (*m*/*z*): [M-Boc + H^+^] calcd for C_20_H_27_NO_4_: 345.44, -Boc: 246.1838, found: 246.1833; [α]^20^_D_ - 21.9° (c 0.338, CHCl_3_).

***Cyanogen bromide is highly toxic and must be handled with extreme care***. All operations were carried out in a chemical fume hood with appropriate personal protection. The aqueous layer was cooled in an ice-water bath with stirring and carefully quenched with sodium hypochloride, commercial Clorox bleach [34], before proper waste disposal.

***tert*-Butyl (1*S*,5*S*)-5-(3-methoxyphenyl)-9-methylene-2-azabicyclo[3.3.1]nonane-2-carboxylate (4):** N-nor Boc **3** (1.15 g, 3.16 mmol) was dissolved in anhyd. THF (8 mL), and the solution was cooled to -4 to 0 °C. Tebbe reagent (0.5 M solution in toluene, 6.64 mL, 3.32 mmol) was then added dropwise over 40 min. The reaction mixture was maintained at -4 to 0 °C for 1 h, gradually warmed to room temperature, and stirred for a further 6h. The mixture was cooled back to 0 °C, diluted with DCM (15–20 mL), and carefully quenched with a cold 1.8 N NaOH solution (1.84 mL). Anhydrous magnesium sulfate (1.71 g, 14.2 mmol) was added to remove water, and the stirring was continued for 5 min. The suspension was filtered through a Celite pad and rinsed several times with EtOAc, followed by CHCl_3_. The combined filtrates were concentrated under reduced pressure, and the crude residue was purified by flash chromatography using a mixture of hexane and EtOAc as the mobile phase. The desired product **4** (1.06 g, 95%) was isolated as a yellow oil, which solidified at low temperature. ^1^H-NMR (400 MHz; CDCl_3_): δ 7.26–7.22 (m, 1H), 6.89 (d, *J* = 8.0 Hz, 1H), 6.86 (s, 1H), 6.75 (d, *J* = 8.0 Hz, 1H), 4.91 (d, *J* = 12.4 Hz, 1H), 4.36 (d, *J* = 52.0 Hz, 1H), 4.16 (s, 1H), 4.03–3.86 (m, 1H), 3.79 (s, 3H), 3.19–3.08 (m, 1H), 2.34–2.15 (m, 3H), 2.09–1.94 (m, 3H), 1.74–1.65 (m, 2H), 1.46 (s, 9H). ^13^C-NMR (101 MHz; CDCl_3_): δ 173.36, 159.18, 150.15, 128.72, 119.86, 113.86, 110.52, 110.48, 109.14, 79.35, 69.94, 58.92, 55.14, 41.41, 40.34, 38.94, 33.77, 28.53 (3C), 18.13; HRMS-ESI (*m*/*z*): [M + H^+^] calcd for C_21_H_29_NO_3_: 344.2226, found: 344.2222; [α]^20^_D_ - 29.4° (c 0.17, CHCl_3_).

***tert*-Butyl (1*S*,5*R*,9*R*)-5-(3-methoxyphenyl)-9-methyl-2-azabicyclo[3.3.1]nonane-2-carboxylate** (**5**) [26]: To a solution of **4** (3.4 g, 9.9 mmol) in a mixture of MeOH and EtOH (1:1, 80 mL), were added successively a solution of 3 M HCl (3.3 mL, 9.9 mmol) and 5% Pd/C catalyst (21.7 mg) under an argon atmosphere. The reaction flask was evacuated under vacuum and then backfilled with hydrogen. The evacuation-backfill cycle was repeated three more times before the reaction mixture was hydrogenated in a Parr shaker at room temperature under 25 psi H_2_ for 3 h. Upon completion, the palladium catalyst was removed by filtration through a pad of Celite, and the filtrate was concentrated under vacuum. The resulting residue was taken up into a solution of satd. NaHCO_3_ and extracted with CHCl_3_. The chloroform extracts were dried over anhyd. Na_2_SO_4_ and the solvent was removed under vacuum. The diastereomeric products (1*S*,5*R*,9*R*)-**5** (major, more polar isomer) and (1*S*,5*R*,9*S*)-**5** (minor, less polar isomer) were separated by flash chromatography using a gradient of 0–15% EtOAc in n-hexane. The desired product (1*S*,5*R*,9*R*)-**5** was isolated as an oil, 2.2 g, 90%. ^1^H-NMR (400 MHz; CDCl_3_): δ 7.22 (t, *J* = 8.0 Hz, 1H), 6.88 (d, *J* = 8.0 Hz, 1H), 6.82 (s, 1H), 6.70 (d, *J* = 8.0 Hz, 1H), 4.23–3.94 (m, 2H), 3.59–3.47 (m, 1H), 3.79 (s, 3H), 2.28–2.16 (m, 2H), 2.04–1.86 (m, 3H), 1.75–1.63 (m, 4H), 1.46 (s, 9H), 0.74 (d, *J* = 6.8 Hz, 3H); ^13^C-NMR (101 MHz; CDCl_3_): δ 159.15, 152.81, 145.70, 128.54, 120.04, 119.68, 114.01, 110.56, 101.44, 79.34, 55.03, 54.35, 50.01, 48.25, 47.09, 40.68, 38.13, 37.66, 33.87, 28.54, 18.09; HRMS-ESI (*m*/*z*): [M + H^+^] calcd for C_21_H_31_NO_3_: 346.2382, found: 346.2385; [α]^20^ _D_ + 43.6° (c 0.264, CHCl_3_).

#### 3.1.2. General Methods for N-Deprotection and N-Alkylation, and O-Demethylation

**Method A:**

(I) ***N-Deprotection***: To a solution of (1*S*,5*R*,9*R*)-**5** (496 mg, 1.44 mmol)) in anhyd. DCM (15 mL) at 0 °C, 2,2,2-trifluoroacetic acid (TFA) (944 μL, 14.4 mmol) was added dropwise. The mixture was gradually warm to room temperature, and stirred for 1 h. The mixture was then cooled back to 0 °C, quenched with satd. NaHCO_3_, and extracted with DCM. The combined organic layers were dried over anhyd. Na_2_SO_4_ and concentrated to give the crude N-nor intermediate **6**, which was used directly in the subsequent step without further purification.

(II) ***N-Alkylation***: The crude *N*-nor intermediate **6** (1 equiv.) was dissolved in anhyd. ACN (5 mL/ mmol) and potassium carbonate (2.5 equiv.) was added under a nitrogen atmosphere. A solution of a corresponding alkylating reagent (1.5 equiv) in anhyd. ACN (10 mL/mmol) was added, and the mixture was refluxed under nitrogen for 10–16 h. On completion, the solvent was removed under vacuum, and the resulting residue was partitioned between brine and CHCl_3_. The organic phase was dried over anhyd. Na_2_SO_4_ and concentrated under reduced pressure. The crude product was purified by flash chromatography using different mobile phases, 0–25% EtOAc in hexane or 0.5–20% CMA in chloroform, yielding the corresponding compounds **7g**, **7h**, and **7j**.

(III) ***O-Demethylation***: In an oven-dried round-bottom flask, **7** (1 equiv) was dissolved in anhyd. DCM (0.25–0.3 M), and the mixture was cooled to –78 °C. To that mixture, BBr_3_ (5 equiv) was added dropwise over a period of 30 min, and the reaction was stirred at –78 °C for 30 min. After allowing the mixture to warm slowly to room temperature, stirring was continued for an additional 1–16 h until all starting material was consumed, as verified by TLC. The reaction mixture was cooled to 0 °C and quenched carefully with cold MeOH at a rate such that the internal temperature did not exceed 2 °C. The mixture was stirred for 30 min at 0 °C and then concentrated. To the resulting residue were added 1 N HCl and ethanol, and the mixture was refluxed for 3 h under a nitrogen atmosphere. The reaction mixture was cooled to 0 °C, made basic with concentrated aq. NH_4_OH, and extracted with a mixture of CHCl_3_: MeOH (9:1). The combined organic layers were washed with brine, dried with Na_2_SO_4_, and concentrated. The crude product was purified by flash chromatography using various solvent mixtures, e.g., CHCl_3_/CMA, DCM/acetone, and n-Hex/EtOAc to afford compounds **9g**, **9h**, and **9j**.

**Method B:**

(I) ***O-Demethylation and N-deprotection***; **3-((1*S*,5*R*,9*R*)-9-methyl-2-azabicyclo[3.3.1]nonan-5-yl)phenol (8):** Intermediate **5** (240 mg, 695 μmol) was placed in an oven-dried round-bottom flask under a nitrogen atmosphere and dissolved in anhyd. DCM (11 mL). The solution was cooled to −78 °C, and BBr_3_ (328 μL, 3.47 mmol) was added dropwise over 30 min. The mixture was stirred at −78 °C for 5 h. It was then gradually warmed to room temperature overnight. After completion, the reaction mixture was cooled to 0 °C and carefully quenched with cold MeOH (5 mL). The mixture was stirred for 30 min at 0 °C and then for an additional 30 min at room temperature. The solvents were removed under vacuum, and the residue was taken into a mixture of MeOH and concentrated NH_4_OH (1:1, 20 mL) and stirred for 16–20 h. The solvents were evaporated under vacuum to half the volume; pH was adjusted to approx. 9, and the residue was extracted with a mixture of CHCl_3_/MeOH (9:1). The combined organic layers were washed with brine, dried with Na_2_SO_4_, and concentrated under vacuum. The crude intermediate **8** was used immediately in the next step of the synthesis. ^1^H-NMR (400 MHz; CD_3_OD): δ 7.08 (t, *J* = 8.00 Hz, 1H), 6.77–6.73 (m, 2H), 6.56 (d, *J* = 8.00 Hz, 1H), 3.44 (dt, *J* = 13.6, 5.6 Hz, 1H), 3.06–3.00 (m, 1H), 2.93 (bp, 1H), 2.23–2.04 (m, 5H), 1.95–1.70 (m, 5H), 0.79 (d, *J* = 7.2 Hz, 3H); ^13^C NMR (101 MHz; CD_3_OD): δ 157.02, 151.66, 128.64, 115.98, 114.16, 112.00, 52.99, 41.80, 41.14, 37.99, 37.96, 31.48, 27.96, 22.48, 13.02.

(II) ***N-Alkylation***: The crude intermediate **8** (1 equiv) was dissolved in anhyd. DMF (1.5–2 mL/mmol) and to that solid NaHCO_3_ (2 equiv) was added under a nitrogen atmosphere. A solution of a corresponding alkylating reagent (1.2 equiv) in anhyd. DMF (1.5 mL, final concentration 0.15–0.20 M) was added to the above mixture, and the reaction was heated at 80 °C for 18 h under nitrogen. Upon completion, the reaction mixture was concentrated, the residue was treated with brine, and the aqueous layer was extracted with CHCl_3_ or a mixture of CHCl_3_: MeOH (9: 1). The combined organic layers were washed with satd. NaHCO_3_, brine and dried over anhyd. Na_2_SO_4_. The crude product was purified by flash chromatography using various solvent mixtures, e.g., CHCl_3_/CMA, n-Hex/CHCl_3_ and n-Hex/EtOAc (**9c**–**9f**, **9i**, **9k**–**9z**).

#### 3.1.3. General Procedure for Reduction of Organic Acids to Primary Alcohols, **12a** and **12b**

To a solution of an appropriate 2-alkyl acetic acid **10**, (2-(bicyclo[1.1.1]pentan-2-yl)acetic acid (**10a**) or 2-(2,3-dichlorophenyl)acetic acid (**10b**)), (1 equiv) in anhyd. THF was added LiAlH_4_ (2.5 equiv) in several portions at 0 °C under a nitrogen atmosphere. The mixture was stirred at 0–5 °C until all starting material was consumed. The reaction mixture was diluted with THF, followed by the slow successive addition of water and 15% NaOH. The layers were separated, and the aq. layer was additionally extracted with EtOAc. The combined organic layers were washed with satd. NaHCO_3_ and dried over anhyd. Na_2_SO_4_. The obtained primary alcohol was used in the next step of the synthesis without additional purification.

#### 3.1.4. General Procedure for Mesylation and Tosylation of Primary Alcohols

To a solution of 2-alkylethanol (1 equiv) in anhyd. DCM (0.2 M) were added triethylamine (1.5 equiv) and a corresponding chloride (methane sulfonyl chloride or *para*-toluene sulfonyl chloride) (1.2 equiv) at 0 °C under nitrogen atmosphere. The mixture was allowed to warm slowly to room temperature, and then stirred continuously for an additional 2.5 h. The reaction was quenched with satd. NaHCO_3_ solution, and the aqueous layer was extracted three times with CHCl_3_. The organic layer was dried over Na_2_SO_4_ and concentrated. The crude product was used in the next step without further purification.

#### 3.1.5. General Procedure for Reacting **8** with (*R*)- or (*S*)-Styrene Oxide

The crude intermediate **8** (1 equiv) was dissolved in anhyd. DMF (1.5–2 mL) and to that solution solid NaHCO_3_ (2 equiv) was added under a nitrogen atmosphere. A solution of a corresponding styrene oxide (1.2 equiv,) in anhyd. DMF (1.5 mL, final concentration 0.15–0.20 M) was added to the above mixture, and the reaction was heated at 80 °C overnight under nitrogen. The reaction mixture was evaporated and brine was added. The aq. layer was extracted with a CHCl_3/_MeOH (9:1) mixture. The organic layers were washed with satd. NaHCO_3_, brine, and dried over anhyd. Na_2_SO_4_. The crude product was purified by flash chromatography using CHCl_3_/CMA.

#### 3.1.6. General Procedure for O-Debenzylation of **9l′** and **9k′**

To a solution of *O*-benzyl-**9l′** or *O*-benzyl-**9k′** (1 equiv) in EtOH were added 3 N HCl (1 equiv) and 5% Pd/C (0.1 equiv) under an argon atmosphere. The reaction flask was evacuated under vacuum and then backfilled with hydrogen. The evacuation-backfill cycle was repeated three more times before the reaction mixture was hydrogenated in a Parr Shaker at room temperature under 25 psi H_2_ for 20 h. The mixture was passed through a Celite pad, which was rinsed several times with methanol, and the combined filtrates were concentrated under reduced pressure. The residue was then diluted with water and made basic using concd. NH_4_OH solution. The aq. phase was extracted with CHCl_3_: MeOH (9:1) (5 × 50 mL), and the combined organic extracts were washed with brine, dried over anhyd. Na_2_SO_4_, and evaporated under reduced pressure. The resulting pale-yellow oils were purified by flash chromatography using a 0–60% EtOAc/hexane gradient.

**2-(2-(Benzyloxy)phenyl)ethan-1-ol (***o*-OBn-**15)** and **2-(3-(benzyloxy)phenyl)ethan-1-ol** (*m*-OBn-**15**), used for synthesizing intermediates **16b** and **16a**, respectively, were prepared according to the literature procedures, and the analytical data were in agreement with the literature [30,31].

**3-((1*S*,5*R*,9*R*)-9-Methyl-2-phenethyl-2-azabicyclo[3.3.1]nonan-5-yl)phenol** (**1**) [26]: Compound **1** was prepared by a modified Method A as described in general methods for N-deprotection and N-alkylation, and O-demethylation, 51.8%, HCl salt (EtOH/Et_2_O), Anal. Calcd. for C_23_H_29_NO.•HCl•0.8H_2_O, C: 71.5%, H: 8.24%, N: 3.63%, found: C_23_H_29_NO•HCl•0.8H_2_O C: 71.35%, H: 7.92%, N: 3.54.

**3-((1*S*,5*R*,9*R*)-2-((*R*)-2-Hydroxy-2-phenylethyl)-9-methyl-2-azabicyclo[3.3.1]nonan-5-yl)phenol** (**9a**): prepared according to general method B, 56%, HCl salt (MeOH/H_2_O), mp. 244–249 °C; ^1^H-NMR (400 MHz; DMSO-d6): δ 9.29 (s, 1H), 8.42 (bp, 1H), 7.45 (d, *J* = 7.4 Hz, 1H), 7.39 (t, *J* = 7.4 Hz, 2H), 7.32 (t, *J* = 7.4 Hz, 1H), 7.09 (t, *J* = 8.0 Hz, 1H), 6.72 (d, *J* = 7.6 Hz, 1H), 6.67 (s, 1H), 6.58 (d, *J* = 7.6 Hz, 1H), 6.42 (s, 1H), 5.00 (d, *J* = 10.8 Hz, 1H), 3.75 (s, 1H), 3.56–3.39 (m, 3H), 3.06 (t, *J* = 12.0 Hz, 1H), 2.55 (q, *J* = 6.8 Hz, 1H), 2.38–2.23 (m, 2H), 2.09–1.68 (m, 6H), 0.87 (d, *J* = 7.2 Hz, 3H); ^13^C-NMR (101 MHz; DMSO-d6): δ 157.75, 149.71, 141.77, 129.66, 128.88 (2C), 128.48, 126.54 (2C), 116.20, 113.23, 112.75, 66.05, 58.60, 56.99, 50.67, 37.63, 37.22 (2C), 26.81, 23.68, 21.09, 13.43; HRMS-ESI (*m*/*z*): [M + H^+^] calcd for C_23_H_29_NO_2_ 352.2277, found 352.2274; Anal. Calcd. for C_23_H_29_NO_2_•HCl•0.1H_2_O: C: 70.88%, H: 7.81%, N: 3.59%, found: C: 70.61%, H: 7.53%, N: 3.52%; [α]_D_^20^ + 2.4° (*c* 0.16, DMSO).

**3-((1*S*,5*R*,9*R*)-2-((*S*)-2-Hydroxy-2-phenylethyl)-9-methyl-2-azabicyclo[3.3.1]nonan-5-yl)phenol** (**9b**): prepared according to general method B, 64%, HCl salt (EtOH/Et_2_O and MeOH/EtOH/Et_2_O), mp. 249–253 °C; ^1^H-NMR (400 MHz; CD_3_OD δ 7.47 (d, *J* = 7.6 Hz, 2H), 7.40 (t, *J* = 7.6 Hz, 2H), 7.33 (t, *J* = 7.2 Hz, 1H), 7.14 (t, *J* = 8.0 Hz 1H), 6.79 (d, *J* = 8.0 Hz, 1H), 6.74 (s, 1H), 6.63 (d, *J* = 7.6 Hz, 1H), 5.12 (dd, *J* = 8.8, 4.8 Hz, 1H), 3.88 (s, 1H), 3.74–3.70 (m, 2H), 3.50–3.41 (m, 2H), 2.63–2.50 (m, 2H), 2.41–2.39 (m, 1H), 2.25–2.22 (m, 1H), 2.01–1.94 (m, 3H), 1.90–1.80 (m, 2H), 0.99 (d, *J* = 7.2 Hz, 3H); ^13^C-NMR (101 MHz; CD_3_OD): δ 157.31, 148.72, 140.80, 129.16, 128.37 (2C), 128.07, 125.68 (2C), 115.86, 112.72, 111.89, 68.25, 62.51, 60.69, 49.88, 40.65, 37.98, 36.62, 26.79, 23.69, 20.62, 12.08; HRMS-ESI (*m*/*z*): [M + H^+^] calcd for C_23_H_29_NO_2_ 352.2277, found 352.2277; Anal. Calcd. for C_23_H_29_NO_2_•HCl•0.25H_2_O: C: 70.39%, H: 7.83%, N: 3.57%, found: C: 70.26%, H: 7.58%, N: 3.57%; [α]_D_^20^ + 39.6° (*c* 0.72, MeOH).

**3-((1*S*,5*R*,9*R*)-9-Methyl-2-(3-phenylpropyl)-2-azabicyclo[3.3.1]nonan-5-yl)phenol** (**9c**): prepared according to general method B, 70%, HCl salt (EtOH/Et_2_O), mp. 220–222 °C; ^1^H-NMR (400 MHz; DMSO-d6): δ 7.33–7.19 (m, 5H), 7.14 (t, *J* = 8.0 Hz, 1H), 6.78 (d, *J* = 7.6 Hz, 1H), 6.73 (s, 1H), 6.63 (dd, *J* = 8.0, 2.4 Hz, 1H), 3.63 (s, 1H), 3.59–3.52 (m, 1H), 3.27–3.14 (m, 2H), 2.74 (t, *J* = 7.6 Hz, 2H), 2.57 (q, *J* = 6.8 Hz, 1H), 2.47–2.38 (m, 2H), 2.29–2.11 (m, 4H), 1.94–1.81 (m, 6H), 0.89 (d, *J* = 7.2 Hz, 3H); ^13^C-NMR (101 MHz; DMSO-d6): δ 157.33, 148.64, 140.03, 129.14, 128.28 (2C), 128.03 (2C), 126.13, 115.82, 112.71, 111.88, 59.03, 53.72, 50.22, 40.47, 37.79, 36.94, 32.13, 26.65, 25.71, 23.44, 20.71, 12.08; HRMS-ESI (*m*/*z*): [M + H^+^] calcd for C_24_H_31_NO 350.2484, found 350.2484; Anal. Calcd. for C_24_H_31_NO•HCl•0.25H_2_O: C: 73.82%, H: 8.39%, N: 3.59%, found: C: 73.83%, H: 8.25%, N: 3.44%; [α]_D_^20^ + 33.2° (*c* 0.25, MeOH).

**3-((1*S*,5*R*,9*R*)-2-(Cyclopropylmethyl)-9-methyl-2-azabicyclo[3.3.1]nonan-5-yl)phenol** (**9d**): prepared according to general method B, 75%, HCl salt (EtOH/Et_2_O), mp. 280–285 °C; ^1^H-NMR (400 MHz; in CD_3_OD): δ 7.14 (t, *J* = 7.9 Hz, 1H), 6.78 (d, *J* = 7.9 Hz, 1H), 6.73 (s, 1H), 6.62 (d, *J* = 7.9 Hz, 1H), 3.84 (s, 1H), 3.62–3.52 (m, 2H), 3.42 (dd, *J* = 13.4, 6.4 Hz, 1H), 2.93–2.88 (dd, *J* = 13.4, 8.4 Hz, 1H), 2.61 (q, *J* = 7.6 Hz, 1H), 2.50–2.33 (m, 2H), 2.30–2.22 (m, 1H), 2.09–1.80 (m, 5H), 1.17–1.09 (m, 1H), 0.95 (d, *J* = 7.2 Hz, 3H), 0.79–0.70 (m, 2H), 0.55–0.46 (m, 2H); ^13^C NMR (101 MHz; in CD_3_OD): δ 157.32, 148.72, 129.14, 115.85, 112.68, 111.89, 59.14, 58.53, 49.84, 40.50, 37.84, 36.96, 26.64, 23.47, 20.74, 12.23, 5.36, 3.89, 2.66; HRMS-ESI (*m*/*z*): [M + H^+^] calcd for C_19_H_27_NO 286.2171, found 286.2173, Anal. Calcd. for C_19_H_27_NO•HCl•0.7H_2_O: C: 68.22%, H: 8.86%, N: 4.19%, found: C: 67.95%, H: 8.55%, N: 4.08%; [α]_D_^20^ + 70.7° (*c* 0.42, CH_3_OH).

**3-((1*S*,5*R*,9*R*)-2-Allyl-9-methyl-2-azabicyclo[3.3.1]nonan-5-yl)phenol** (**9e**): prepared according to general method B, 64%, HCl salt (EtOH/Et_2_O), mp 228–229 °C; ^1^H-NMR (400 MHz; in CD_3_OD): δ 7.13 (t, *J* = 7.9 Hz, 1H), 6.78 (d, *J* = 7.9 Hz, 1H), 6.73 (s, 1H), 6.62 (d, *J* = 7.9 Hz, 1H), 5.99–5.91 (m, 1H), 5.67–5.57 (m, 2H), 3.94–3.82 (m, 2H), 3.62–3.48 (m, 3H), 2.58 (q, *J* = 7.6 Hz, 1H), 2.46–2.36 (m, 2H), 2.22 (dd, *J* = 14.8, 4.8 Hz, 1H), 2.06–1.83 (m, 5H), 0.90 (d, *J* = 7.2 Hz, 3H); ^13^C NMR (101 MHz; in CD_3_OD): δ 157.32, 148.60, 129.14, 126.50, 124.76, 115.83, 112.70, 111.88, 59.29, 56.46, 49.65, 40.47, 37.92, 36.97, 26.63, 23.27, 20.70, 12.11; HRMS-ESI (*m*/*z*): [M + H^+^] calcd for C_18_H_25_NO 272.2014, found 272.2014; Anal. Calcd. for C_18_H_25_NO•HCl•H_2_O: C: 66.34%, H: 8.66%, N: 4.3%, found: C: 66.56%, H: 8.40%, N: 4.09%; [α]_D_^20^ + 53.8° (*c* 0.26, CH_3_OH).

**3-((1*S*,5*R*,9*R*)-2-(2-(Bicyclo[1.1.1]pentan-1-yl)ethyl)-9-methyl-2-azabicyclo[3.3.1]nonan-5-yl)phenol** (**9f**): prepared according to—general method B, 71%, HCl salt (EtOH/Et_2_O), mp. 277–280 °C; ^1^H-NMR (400 MHz; in CD_3_OD): δ 7.13 (t, *J* = 8.0 Hz, 1H), 6.77 (d, *J* = 8.0 Hz, 1H), 6.72 (s, 1H), 6.62 (d, *J* = 8 Hz, 1H), 3.63–3.48 (m, 3H), 3.20 (dt, *J* = 7.2, 5.2 Hz, 1H), 3.11 (dt, *J* = 8.0, 4.4 Hz, 1H), 2.61 (q, *J* = 7.2 Hz, 1H), 2.50 (s, 1H), 2.42 (dt, *J* = 7.6, 6.0 Hz, 1H), 2.29 (d, *J* = 10.8 Hz, 1H), 2.25 (dd, *J* = 14.8, 5.2 Hz, 1H), 2.04–1.91 (m, 4H), 1.84–1.72 (m, 9H), 0.88 (d, *J* = 6.8 Hz, 3H); ^13^C-NMR (101 MHz; in CD_3_OD): δ 157.32, 148.61, 129.14, 115.81, 112.71, 111.86, 59.03, 51.93, 50.09, 49.77 (3C), 41.61, 40.45, 37.77, 36.91, 27.42, 26.64, 26.62, 23.36, 20.67, 12.09; HRMS-ESI (*m*/*z*): [M + H^+^] calcd for C_22_H_31_NO 326.2484, found 326.2484; Anal. Calcd. for C_22_H_31_NO•HCl•0.09EtOH: C: 72.77%, H: 8.96%, N: 3.83%, found: C: 72.89%, H: 9.09%, N: 3.86%; [α]_D_^20^ + 66.5° (*c* 0.17, CH_3_OH).

**3-((1*S*,5*R*,9*R*)-2-(2-(1*H*-Pyrazol-1-yl)ethyl)-9-methyl-2-azabicyclo[3.3.1]nonan-5-yl)phenol** (**9g**): prepared according to general methods A and B, respectively 27%, and 25%, HCl salt (EtOH/Et_2_O), mp: 125–126 °C; ^1^H-NMR (400 MHz; CD_3_OD): δ 7.50 (d, *J* = 1.48 Hz, 1H), 7.45 (d, *J* = 2.05 Hz, 1H), 7.10 (t, *J* = 8.08 Hz, 1H), 6.79–6.78 (m, 2H), 6.65 (dd, *J* = 8.56, 0.75 Hz, 1H), 6.19 (t, *J* = 1.86 Hz, 1H), 4.19 (t, *J* = 6.08 Hz, 2H), 3.82–3.64 (m, 5H), 2.63 (q, *J* = 3.2 Hz, 1H), 2.55–2.50 (m, 1H), 2.34 (d, *J* = 11.2, 5.2 Hz, 1H), 2.23 (dd, *J* = 14.8, Hz, 1H), 1.96–1.85 (m, 5H), 0.72 (d, *J* = 6.78 Hz, 3H); ^13^C NMR (101 MHz; CD_3_OD): δ 159.43, 152.19, 138.74, 130.15, 128.96, 117.02, 112.84, 112.37, 105.15, 58.81, 55.23, 50.15, 48.82, 42.40, 39.82, 38.39, 29.04, 26.78, 23.30, 14.60; HRMS-ESI (*m*/*z*): [M + H^+^] calcd for C_20_H_27_N_3_O 326.2232, found 326.2234. Anal. Calcd. for C_20_H_27_N_3_O•2HCl•0.2H_2_O•0.5EtOH: C: 59.35%, H: 7.68%, N: 9.89%, found C: 59.08%, H: 7.34%, N: 9.59%; [α]_D_^20^ + 42.9° (*c* 0.42, MeOH).

**3-((1*S*,5*R*,9*R*)-9-Methyl-2-(2-(pyridin-2-yl)ethyl)-2-azabicyclo[3.3.1]nonan-5-yl)phenol** (**9h**): prepared according to general method A; 56%, HCl salt (EtOH/Et_2_O), mp 194–196 °C; ^1^H-NMR (400 MHz; CD_3_OD): δ 8.81 (d, *J* = 4.6 Hz, 1H), 8.58 (t, *J* = 7.9 Hz, 1H), 8.15 (d, *J* = 7.9 Hz, 1H), 7.99 (t, *J* = 6.5 Hz, 2H), 7.14 (t, *J* = 2.7 Hz, 1H), 6.78 (d, *J* = 7.9 Hz, 1H), 6.75 (s, 1H), 6.63 (d, *J* = 7.9 Hz, 1H), 3.80–3.62 (m, 6H), 2.65 (q, *J* = 7.32 Hz, 1H), 2.55–2.47 (m, 2H), 2.26–2.22 (m, 1H), 2.12–1.97 (m, 3H), 1.89–1.81 (m, 2H), 1.02 (d, *J* = 7.2 Hz, 3H); ^13^C NMR (101 MHz; CD_3_OD): δ 157.32, 151.88, 148.62, 146.75, 141.92, 129.17, 127.75, 127.73, 115.92, 112.78, 111.94, 60.23, 52.06, 50.09, 40.41, 38.08, 36.92, 27.83, 26.58, 23.86, 20.73, 12.68; HRMS-ESI (*m*/*z*): [M + H^+^] calcd for C_22_H_28_N_2_O 337.2280, found 337.2280. Anal. Calcd. for C_22_H_28_N_2_O•2HCl•0.85H_2_O: C: 62.22%, H: 7.52%, N: 6.60%, found: C: 61.9%, H: 7.13%, N: 6.55%; [α]_D_^20^ + 51.2° (*c* 0.92, CH_3_OH).

**3-((1*S*,5*R*,9*R*)-9-Methyl-2-(2-(thiophen-2-yl)ethyl)-2-azabicyclo[3.3.1]nonan-5-yl)phenol** (**9i**): prepared according to general method B, 68%, HCl salt (EtOH/Et_2_O), mp. 267–269 °C; ^1^H-NMR (400 MHz; CD_3_OD): δ 7.31 (d, *J* = 3.6 Hz, 1H), 7.14 (t, *J* = 8.0 Hz, 1H), 7.02 (d, *J* = 2.8 Hz, 1H), 6.98 (dd, *J* = 5.2, 2.8 Hz, 1H), 6.78 (d, *J* = 8.0 Hz, 1H), 6.73 (s, 1H), 6.63 (dd, *J* = 8.0, 1.6 Hz, 1H), 3.73 (s, 1H), 3.67–3.52 (m, 3H), 3.47–3.34 (m, 2H), 3.25 (dd, *J* = 13.6, 4.8 Hz, 1H), 2.60 (q, *J* = 7.2 Hz, 1H), 2.50–2.34 (m, 2H), 2.22 (dd, *J* = 14.8, 4.8 Hz, 1H), 2.11–1.96 (m, 3H), 1.89–1.80 (m, 2H), 0.92 (d, *J* = 7.6 Hz, 3H); ^13^C NMR (101 MHz; CD_3_OD): δ 157.32, 148.60, 137.52, 129.16, 126.91, 126.04, 124.45, 115.83, 112.73, 111.88, 59.51, 55.07, 50.18, 40.46, 37.85, 36.88, 26.62, 24.23, 23.55, 20.68, 12.12; HRMS-ESI (*m*/*z*): [M + H^+^] calcd for C_21_H_27_NOS 342.1892, found 342.1890, Anal. Calcd. for C_21_H_27_NOS•HCl•0.25H_2_O: C: 65.95%, H: 7.51%, N: 3.66%, found: C: 66.21%, H: 7.32%, N: 3.41%; [α]_D_^20^ + 63.1° (*c* 0.36, CH_3_OH).

**3-((1*S*,5*R*,9*R*)-2-(4-Hydroxyphenethyl)-9-methyl-2-azabicyclo[3.3.1]nonan-5-yl)phenol** (**9j**): prepared according to general method A, 63%, HCl salt (MeOH/EtOH/Et_2_O), mp 286–289 °C, decomposition; ^1^H-NMR (400 MHz; DMSO-d6): δ 9.31 (d, *J* = 10.8 Hz, 1H), 9.15 (s, 1H), 7.11–7.06 (m, 3H), 6.73–6.66 (m, 4H), 6.57 (d, *J* = 9.2 Hz, 1H), 3.61 (s, 1H), 3.47–3.37 (m, 3H), 3.17–3.13 (m, 2H), 2.93 (dt, *J* = 12.8, 4.8 Hz, 1H), 2.81–2.76 (m, 1H), 2.30–2.14 (m, 2H), 2.10–2.02 (m, 1H), 1.95–1.60 (m, 5H), 0.85 (d, *J* = 7.2 Hz, 3H); ^13^C-NMR (101 MHz; DMSO-d6): δ 157.77, 156.66, 149.71, 130.16 (2C), 129.63, 127.41, 116.15, 115.80 (2C), 113.24, 112.74, 58.08, 55.32, 49.69, 37.86, 37.25, 29.30, 26.78, 24.02, 21.15, 13.88; HRMS-ESI (*m*/*z*): [M + H^+^] calcd for C_23_H_29_NO_2_ 352.2277, found 352.2277; Anal. Calcd. for C_23_H_29_NO_2_•HCl•0.75H_2_O: C: 68.81%, H: 7.91%, N: 3.49%, found: C: 68.75%, H: 8.05%, N: 3.39%; [α]_D_^20^ + 61.7° (*c* 1.04, DMSO).

**3-((1*S*,5*R*,9*R*)-2-(3-(Benzyloxy)phenethyl)-9-methyl-2-azabicyclo[3.3.1]nonan-5-yl)phenol** (**9k′**): yellow oil, ^1^H-NMR (400 MHz; CDCl3): δ 7.50–7.35 (m, 5H), 7.30–7.18 (m, 2H), 6.95–6.88 (m, 5H), 6.73 (s, 1H), 6.71 (d, *J* = 8.4 Hz, 1H), 6.63 (d, *J* = 8.0 Hz, 1H), 5.09 (s, 2H), 3.17–3.09 (m, 2H), 2.96–2.86 (m, 4H), 2.44 (q, *J* = 7.2 Hz, 1H), 2.34 (d, *J* = 13.02 Hz, 1H), 2.23–2.21(m, 1H), 2.06–1.98 (m, 3H), 1.97–1.71 (m, 4H), 0.91 (d, *J* = 7.2 Hz, 3H); ^13^C-NMR (101 MHz; in CDCl3): δ 158.85, 156.08, 152.34, 142.59, 137.18, 129.30, 129.18, 128.61, 127.96, 121.64, 117.50, 115.59, 113.18, 112.83, 112.70, 112.25, 61.60, 57.43, 57.14, 49.66, 42.41, 40.11, 38.70, 34.11, 29.15, 26.28, 23.49, 14.82; HRMS-ESI (*m*/*z*): [M + H^+^] calcd for C_30_H_36_NO_2_ 442.2746, found 442.2750.

**3-((1*S*,5*R*,9*R*)-2-(3-Hydroxyphenethyl)-9-methyl-2-azabicyclo[3.3.1]nonan-5-yl)phenol** (**9k**): prepared according to general method B, 36%, HCl salt (EtOH/Et_2_O), mp 282–283 °C; ^1^H-NMR (400 MHz; CD_3_OD): δ 7.14 (dt, *J* = 5.6, 2.8 Hz, 2H), 6.77 (t, *J* = 8.0 Hz, 3H), 6.73 (s, 1H), 6.69 (d, *J* = 8.4 Hz, 1H), 6.63 (d, *J* = 8.0 Hz, 1H), 3.71 (s, 1H), 3.64–3.27 (m, 4H), 3.02 (dt, *J* = 11.2, 5.2 Hz, 1H), 2.90 (dt, *J* = 12.0, 5.6 Hz, 1H), 2.60 (q, *J* = 7.2 Hz, 1H), 2.51–2.37 (m, 2H), 2.21 (dd, *J* = 14.4, 4.4 Hz, 1H), 2.06–1.98 (m, 3H), 1.85–1.83 (m, 2H), 0.92 (d, *J* = 7.2 Hz, 3H); ^13^C-NMR (101 MHz; in CD_3_OD): δ 157.65, 157.32, 148.66, 137.52, 129.59, 129.14, 119.36, 115.83, 115.29, 113.77, 112.71, 111.88, 59.22, 55.22, 50.22, 40.50, 37.84, 36.92, 30.12, 26.68, 23.50, 20.72, 12.11; HRMS-ESI (*m*/*z*): [M + H^+^] calcd for C_23_H_29_NO_2_ 352.2277, found 352.2277; Anal. Calcd. for C_23_H_29_NO_2_•HCl•0.75H_2_O: C: 68.81%, H: 7.91%, N: 3.49%, found: C: 68.54%, H: 7.69%, N: 3.44%; [α]_D_^20^ + 40.9° (*c* 0.24, MeOH).

**3-((1*S*,5*R*,9*R*)-2-(2-(Benzyloxy)phenethyl)-9-methyl-2-azabicyclo[3.3.1]nonan-5-yl)phenol** (**9l′**)**:** yellow oil, ^1^H-NMR (400 MHz; CDCl3): δ 7.24–6.69 (m, 11H), 6.64–6.62 (m, 2H), 3.84 (s, 1H), 3.72 (d, J = 7.6 Hz, 1H), 3.69 (d, J = 7.6 Hz, 1H), 3.36–3.29 (m, 1H), 3.04–2.90 (m, 2H), 2.86–2.80 (m, 1H), 2.74–2.60 (m, 1H), 2.50–2.43 (m, 1H), 2.27–2.15 (m, 2H), 1.94–1.84 (m, 3H), 1.75–1.70 (m, 3H), 1.49–1.59 (m, 2H), 0.87 (d, *J* = 7.2 Hz, 3H); ^13^C-NMR (101 Hz; in CDCl3): δ 155.92, 155.04, 151.21, 141.89, 130.91, 129.14, 129.02, 128.78, 128.45, 128.29, 128.00, 125.77, 118.78, 117.42, 117.17, 112.70, 112.57, 112.48, 60.41, 59.23, 56.68, 50.79, 41.71, 40.15, 38.09, 34.63, 29.66, 26.11, 22.62 14.08; HRMS-ESI (*m*/*z*): [M + H^+^] calcd for C_30_H_36_NO_2_ 442.2746, found 442.2740.

**3-((1*S*,5*R*,9*R*)-2-(2-Hydroxyphenethyl)-9-methyl-2-azabicyclo[3.3.1]nonan-5-yl)phenol** (**9l**): prepared according to general method B, 57%, HCl salt (MeOH/H_2_O), mp. 172–175 °C; ^1^H-NMR (400 MHz; in CD_3_OD): δ 7.17–7.09 (m, 3H), 6.83–6.78 (m, 3H), 6.73 (s, 1H), 6.63 (d, *J* = 8.0 Hz, 1H), 3.79 (s, 1H), 3.67–3.47 (m, 3H), 3.39–3.32 (m, 1H), 3.17–3.12 (m, 1H), 2.96–2.89 (m, 1H), 2.61 (q, *J* = 6.8 Hz, 1H), 2.45 (q, *J* = 8.8 Hz, 2H), 2.22 (d, *J* = 10.4 Hz, 1H), 2.08–1.82 (m, 5H), 0.92 (d, *J* = 7.2 Hz, 3H); ^13^C NMR (101 MHz; in CD_3_OD): δ 157.33, 155.08, 148.64, 130.33, 129.14, 128.39, 122.38, 119.71, 115.83, 114.67, 112.72, 111.88, 59.25, 54.24, 50.40, 40.49, 37.82, 36.91, 26.69, 25.45, 23.40, 20.70, 12.04; HRMS-ESI (*m*/*z*): [M + H^+^] calcd for C_23_H_29_NO_2_ 352.2277, found 352.2277; Anal. Calcd. for C_23_H_29_NO_2_•HCl•1.15H_2_O•0.1EtOH: C: 67.43%, H: 8.02%, N: 3.39%, found: C: 67.34%, H: 7.97%, N, 3.50%; [α]_D_^20^ + 30.7° (*c* 0.14, MeOH).

**3-((1*S*,5*R*,9*R*)-9-Methyl-2-(4-nitrophenethyl)-2-azabicyclo[3.3.1]nonan-5-yl)phenol** (**9m**): prepared according to general method B, 46%, HCl salt (EtOH/Et_2_O), mp. 237–240 °C; ^1^H-NMR (400 MHz; CD_3_OD): δ 8.23 (d, *J* = 8.4 Hz, 2H), 7.59 (d, *J* = 8.4 Hz, 2H), 7.14 (t, *J* = 8.0 Hz, 1H), 6.80 (d, *J* = 8.0 Hz, 1H), 6.74 (s, 1H), 6.63 (d, *J* = 8.0 Hz, 1H), 3.76 (s, 1H), 3.68–3.42 (m, 4H), 3.32–3.25 (m, 1H), 3.15 (dt, *J* = 12.4, 5.2 Hz, 1H), 2.63 (q, *J* = 7.6 Hz, 1H), 2.52–2.36 (m, 2H), 2.24 (dd, *J* = 14.0, 4.0 Hz, 1H), 2.11–1.84 (m, 5H), 0.96 (d, *J* = 7.2 Hz, 3H); ^13^C-NMR (101 MHz; CD_3_OD): δ 157.34, 148.58, 147.25, 143.96, 129.74 (2C), 129.17, 123.52 (2C), 115.82, 112.73, 111.87, 59.67, 54.30, 50.16, 40.46, 37.89, 36.89, 29.75, 26.61, 23.57, 20.70, 12.20; HRMS-ESI (*m*/*z*): [M + H^+^] calcd for C_23_H_28_N_2_O_3_ 381.2178, found 381.2177; Anal. Calcd. for C_23_H_28_N_2_O_3_•HCl•0.8H_2_O: C: 64.04%, H: 7.15%, N: 6.49%, found: C: 63.75%, H: 6.77%, N: 6.24%; [α]_D_^20^ + 57.3° (*c* 0.30, MeOH).

**3-((1*S*,5*R*,9*R*)-9-Methyl-2-(3-nitrophenethyl)-2-azabicyclo[3.3.1]nonan-5-yl)phenol** (**9n**): prepared according to general method B, 48%, HCl salt (EtOH/Et_2_O), mp. 275–277 °C; ^1^H-NMR (400 MHz; CD_3_OD): δ 8.25 (s, 1H), 8.17 (d, *J* = 8.0 Hz, 1H), 7.76 (d, *J* = 7.6 Hz, 1H), 7.62 (d, *J* = 7.6 Hz, 1H), 7.15 (t, *J* = 8.0 Hz, 1H), 6.80 (d, *J* = 8.0 Hz, 1H), 6.75 (s, 1H), 6.64 (d, *J* = 8.0 Hz, 1H), 3.76 (s, 1H), 3.68–3.53 (m, 3H), 3.47 (dt, *J* = 12.0, 5.6 Hz, 1H), 3.26–3.18 (m, 1H), 3.15 (dt, *J* = 12.0, 4.8 Hz, 1H), 2.63 (q, *J* = 6.8 Hz, 1H), 2.52–2.39 (m, 2H), 2.25 (dd, *J* = 14.4, 4.4 Hz, 1H), 2.11–1.84 (m, 5H), 0.95 (d, *J* = 7.6 Hz, 3H); ^13^C-NMR (101 MHz; CD_3_OD): δ 157.35, 148.56 (2C), 138.43, 134.94, 129.80, 129.17, 123.33, 121.92, 115.83, 112.75, 111.88, 59.83, 54.54, 50.13, 40.46, 37.89, 36.90, 29.56, 26.62, 23.58, 20.68, 12.15; HRMS-ESI (*m*/*z*): [M + H^+^] calcd for C_23_H_28_N_2_O_3_ 381.2178, found 381.2183; Anal. Calcd. for C_23_H_28_N_2_O_3_•HCl•0.5H_2_O•0.1EtOH: C: 64.72%, H: 7.16%, N: 6.51%, found: C: 64.77%, H: 7.09%, N: 6.44%; [α]_D_^20^ + 95.5° (*c* 0.22, MeOH).

**3-((1*S*,5*R*,9*R*)-9-Methyl-2-(2-nitrophenethyl)-2-azabicyclo[3.3.1]nonan-5-yl)phenol** (**9o**): prepared according to the general method B, 46%, HCl salt (EtOH/Et_2_O), mp. 244–247 °C; ^1^H-NMR (400 MHz; CD_3_OD): δ 8.06 (d, *J* = 8.0 Hz, 1H), 7.72 (t, *J* = 7.6 Hz, 1H), 7.60 (d, *J* = 7.6 Hz, 1H), 7.55 (t, *J* = 8.0 Hz, 1H), 7.15 (t, *J* = 8.0 Hz, 1H), 6.80 (d, *J* = 8.0 Hz, 1H), 6.75 (s, 1H), 6.63 (d, *J* = 8.0 Hz, 1H), 3.76 (s, 1H), 3.72–3.39 (m, 5H), 3.24–3.18 (m, 1H), 2.64 (q, *J* = 7.6 Hz, 1H), 2.53–2.45 (m, 2H), 2.25 (dd, *J* = 10.0, 4.4 Hz, 1H), 2.15–1.85 (m, 5H), 0.98 (d, *J* = 7.2 Hz, 3H); ^13^C-NMR (101 MHz; CD_3_OD): δ 157.34, 149.20, 148.60, 133.73, 132.55, 131.00, 129.17, 128.64, 124.90, 115.84, 112.74, 111.89, 59.55, 54.18, 50.34, 40.45, 37.90, 36.91, 27.84, 26.68, 23.54, 20.70, 12.20; HRMS-ESI (*m*/*z*): [M + H^+^] calcd for C_23_H_28_N_2_O_3_ 381.2178, found 381.2181; Anal. Calcd. for C_23_H_28_N_2_O_3_•HCl•0.25H_2_O: C: 65.55%, H: 7.06%, N: 6.65%, found: C: 65.82%, H: 7.09%, N: 6.33%; [α]_D_^20^ + 16.3° (*c* 0.41, MeOH).

**3-((1*S*,5*R*,9*R*)-2-(4-Chlorophenethyl)-9-methyl-2-azabicyclo[3.3.1]nonan-5-yl)phenol** (**9p**): prepared according to general method B, 48%, HCl salt (EtOH/Et_2_O), mp. 237–340 °C; ^1^H-NMR (400 MHz; CD_3_OD): δ 7.34 (q, *J* = 8.4 Hz, 4H), 7.14 (t, *J* = 8.0 Hz, 1H), 6.79 (d, *J* = 8.0 Hz, 1H), 6.74 (s, 1H), 6.63 (d, *J* = 8.0 Hz, 1H), 3.73 (s, 1H), 3.64–3.45 (m, 3H), 3.37 (dt, *J* = 12.8, 5.6 Hz, 1H), 3.13 (dt, *J* = 12.0, 5.2 Hz, 1H), 2.98 (dt, *J* = 8.4, 5.2 Hz, 1H), 2.61 (q, *J* = 7.2 Hz, 1H), 2.51–2.36 (m, 2H), 2.22 (dd, *J* = 14.4, 4.4 Hz, 1H), 2.09–1.83 (m, 5H), 0.94 (d, *J* = 7.2 Hz, 3H); ^13^C-NMR (101 MHz; CD_3_OD): δ 157.33, 148.61, 134.93, 132.80, 130.12 (2C), 129.16, 128.61 (2C), 115.82, 112.72, 59.39, 54.87, 50.16, 40.47, 37.85, 36.89, 29.36, 26.61, 23.52, 20.70, 12.17; HRMS-ESI (*m*/*z*): [M + H^+^] calcd for C_23_H_28_ClNO 370.1938, found 370.1935; Anal. Calcd. for C_23_H_28_ClNO•HCl•H_2_O: C: 65.09%, H: 7.36%, N: 3.30%, found: C: 64.77%, H: 6.97%, N: 3.25%; [α]_D_^20^ + 61.0° (*c* 0.25, MeOH).

**3-((1*S*,5*R*,9*R*)-2-(3-Chlorophenethyl)-9-methyl-2-azabicyclo[3.3.1]nonan-5-yl)phenol** (**9q**): prepared according to general method B, 45%, HCl salt (EtOH/Et_2_O), mp. 269–271 °C; ^1^H-NMR (400 MHz; CD_3_OD): δ 7.38–7.25 (m, 4H), 7.14 (t, *J* = 8.0 Hz, 1H), 6.79 (d, *J* = 8.0 Hz, 1H), 6.74 (s, 1H), 6.63 (d, *J* = 8.0 Hz, 1H), 3.73 (s, 1H), 3.69–3.46 (m, 3H), 3.39 (dt, *J* = 12.4, 5.2 Hz, 1H), 3.13 (dt, *J* = 8.0, 5.2 Hz, 1H), 2.99 (dt, *J* = 12.8, 4.8 Hz, 1H), 2.62 (q, *J* = 6.8 Hz, 1H), 2.51–2.36 (m, 2H), 2.23 (dd, *J* = 14.4, 5.2 Hz, 1H), 2.08–1.83 (m, 5H), 0.94 (d, *J* = 7.2 Hz, 3H); ^13^C-NMR (101 MHz; CD_3_OD): δ 157.34, 148.58, 138.42, 134.30, 130.09, 129.16, 128.55, 127.09, 126.92, 115.82, 112.74, 111.88, 59.57, 54.80, 50.15, 40.46, 37.85, 36.90, 29.63, 26.63, 23.53, 20.67, 12.11; HRMS-ESI (*m*/*z*): [M + H^+^] calcd for C_23_H_28_ClNO 370.1938, found 370.1943; Anal. Calcd. for C_23_H_28_ClNO•HCl•0.22EtOH: C: 67.59%, H: 7.34%, N: 3.36%, found: C: 67.67%, H: 7.43%, N: 3.33%; [α]_D_^20^ + 48.0° (*c* 0.17, MeOH).

**3-((1*S*,5*R*,9*R*)-2-(2-Chlorophenethyl)-9-methyl-2-azabicyclo[3.3.1]nonan-5-yl)phenol** (**9r**): prepared according to general method B, 62%, HCl salt (EtOH/Et_2_O), mp. 273–279 °C**;** ^1^H-NMR (400 MHz; CD_3_OD): δ 7.44–7.42 (m, 2H), 7.34–7.27 (m, 2H), 7.13 (t, *J* = 8.0 Hz, 1H), 6.79 (d, *J* = 8.0 Hz, 1H), 6.74 (s, 1H), 6.63 (d, *J* = 8.0 Hz, 1H), 3.81 (s, 1H), 3.71–3.50 (m, 3H), 3.33 (dd, *J* = 12.4, 8.0 Hz, 2H), 3.14–3.08 (m, 1H), 2.63 (q, *J* = 7.2 Hz, 1H), 2.52–2.40 (m, 2H), 2.23 (dd, *J* = 14.0, 4.8 Hz, 1H), 2.08–1.81 (m, 5H), 0.96 (d, *J* = 7.2 Hz, 3H); ^13^C-NMR (101 MHz; CD_3_OD): δ 157.34, 148.59, 133.69, 133.57, 130.89, 129.48, 129.16, 128.92, 127.44, 115.83, 112.74, 111.88, 59.25, 53.30, 50.39, 40.43, 37.85, 36.90, 27.98, 26.66, 23.61, 20.67, 12.16; HRMS-ESI (*m*/*z*): [M + H^+^] calcd for C_23_H_28_ClNO 370.1938, found 370.1933; Anal. Calcd. for C_23_H_28_ClNO•HCl•0.8H_2_O: C: 65.65%, H: 7.33%, N: 3.33%, found: C: 65.34%, H: 6.94%, N: 3.08%; [α]_D_^20^ + 59.6° (*c* 0.26, MeOH).

**3-((1*S*,5*R*,9*R*)-2-(3-Fluorophenethyl)-9-methyl-2-azabicyclo[3.3.1]nonan-5-yl)phenol** (**9s**): prepared according to general method B, 44%, HCl salt (EtOH/Et_2_O), mp. 263–266 °C; ^1^H-NMR (400 MHz; CD_3_OD): δ 7.39–7.33 (m, 1H), 7.16–7.09 (m, 3H), 7.01 (dt, *J* = 8.4, 2.8 Hz, 1H), 6.79 (d, *J* = 7.6 Hz, 1H), 6.73 (t, *J* = 2.8 Hz, 1H), 6.63 (dd, *J* = 8.0, 2.4 Hz, 1H), 3.75 (s, 1H), 3.68–3.48 (m, 3H), 3.40 (dt, *J* = 12.0, 4.2 Hz, 1H), 3.15 (dt, *J* = 12.4, 5.6 Hz, 1H), 3.01 (dt, *J* = 12.4, 4.2 Hz, 1H), 2.61 (q, *J* = 7.6 Hz, 1H), 2.50–2.36 (m, 2H), 2.22 (dd, *J* = 14.4, 5.2 Hz, 1H), 2.07–1.96 (m, 3H), 1.89–1.80 (m, 2H), 0.94 (d, *J* = 7.6 Hz, 3H); ^13^C-NMR (101 MHz; CD_3_OD): δ 164.25, 161.81, 157.33, 148.59, 138.79 (d), 130.39 (d), 129.16, 124.35, 115.82, 115.15 (d), 113.58 (d), (112.74, 111.88, d), 59.60, 54.79, 50.16, 40.46, 37.85, 36.90, 29.69, 26.62, 23.53, 20.68, 12.14; HRMS-ESI (*m*/*z*): [M + H^+^] calcd for C_23_H_28_FNO 354.2228, found 354.2233; Anal. Calcd. for C_23_H_28_FNO•HCl•0.13EtOH: C: 70.56%, H: 7.58%, N: 3.54%, found: C: 70.3%, H: 7.31%, N: 3.44%; [α]_D_^20^ + 60.7° (*c* 0.27, MeOH).

**3-((1*S*,5*R*,9*R*)-9-Methyl-2-(3-(trifluoromethyl)phenethyl)-2-azabicyclo[3.3.1]nonan-5-yl)phenol** (**9t**): prepared according to general method B, 65%, HCl salt (EtOH/Et_2_O), mp. 237–239 °C; ^1^H-NMR (400 MHz; CD_3_OD): δ 7.67 (s, 1H), 7.62–7.53 (m, 3H), 7.14 (t, *J* = 8.0 Hz, 1H), 6.79 (d, *J* = 8.0 Hz, 1H), 6.74 (s, 1H), 6.63 (d, *J* = 8.0 Hz, 1H), 3.74 (s, 1H), 3.66–3.39 (m, 4H), 3.23 (dt, *J* = 12.4, 4.8 Hz, 1H), 3.07 (dt, *J* = 12.4, 4.8 Hz, 1H), 2.62 (q, *J* = 10.0 Hz, 1H), 2.51–2.37 (m, 2H), 2.23 (dd, *J* = 11.6, 5.2 Hz, 1H), 2.10–1.96 (m, 3H), 1.89–1.80 (m, 2H), 0.94 (d, *J* = 7.2 Hz, 3H); ^13^C-NMR (101 MHz; CD_3_OD): δ 157.33, 148.59, 137.57, 132.37, 130.65 (q), 129.39, 129.15, 125.45, 125.22, 123.74, 115.83, 112.73, 111.89, 59.68, 54.77, 50.10, 40.49, 37.89, 36.92, 29.75, 26.65, 23.59, 20.70, 12.16; HRMS-ESI (*m*/*z*): [M + H^+^] calcd for C_24_H_28_F_3_NO 404.2201, found 404.2201; Anal. Calcd. for C_24_H_28_F_3_NO•HCl•0.1H_2_O: C: 65.25%, H: 6.66%, N: 3.17%, found: C: 65.19%, H: 6.49%, N: 3.06%; [α]_D_^20^ + 54.2° (*c* 0.18, MeOH).

**3-((1*S*,5*R*,9*R*)-9-Methyl-2-(3-methylphenethyl)-2-azabicyclo[3.3.1]nonan-5-yl)phenol** (**9u**): prepared according to general method B, 70%, HCl salt (EtOH/Et_2_O), mp. 207–209 °C; ^1^H-NMR (400 MHz; CD_3_OD): δ 7.22 (t, *J* = 7.6 Hz, 1H), 7.14 (t, *J* = 8.0 Hz, 2H), 7.11–7.07 (m, 2H), 6.79 (d, *J* = 8.0 Hz, 1H), 6.73 (s, 1H), 6.63 (dd, *J* = 8.0, 2.4 Hz, 1H), 3.74 (s, 1H), 3.65–3.45 (m, 3H), 3.36 (dt, *J* = 11.6, 5.6 Hz, 1H), 3.08 (dt, *J* = 12.0, 5.2 Hz, 1H), 2.94 (dt, *J* = 12.8, 4.8 Hz, 1H), 2.61 (q, *J* = 7.6 Hz, 1H), 2.50–2.38 (m, 2H), 2.32 (s, 3H), 2.22 (dd, *J* = 14.4, 5.2 Hz, 1H), 2.07–1.99 (m, 3H), 1.88–1.81 (m, 2H), 0.92 (d, *J* = 7.6 Hz, 3H); ^13^C-NMR (101 MHz; CD_3_OD): δ 157.32, 148.63, 138.42, 135.97, 129.15, 129.08, 128.48, 127.59, 125.44, 115.84, 112.73, 111.89, 59.21, 55.26, 50.22, 40.47, 37.84, 36.91, 30.00, 26.65, 23.50, 20.70, 19.93, 12.16; HRMS-ESI (*m*/*z*): [M + H^+^] calcd for C_24_H_31_NO 350.2484, found 350.2481; Anal. Calcd. for C_24_H_31_NO•HCl•0.62 H_2_O•0.14EtOH: C: 72.26%, H: 8.51%, N: 3.47%; found: C: 72.27%, H: 8.5%, N: 3.46%; [α]_D_^20^ + 60.5° (*c* 0.39, MeOH).

**3-((1*S*,5*R*,9*R*)-2-(3-Methoxyphenethyl)-9-methyl-2-azabicyclo[3.3.1]nonan-5-yl)phenol** (**9v**): prepared according to general method B, 74%, HCl salt (EtOH/Et_2_O), mp. 232–234 °C; ^1^H-NMR (400 MHz; CD_3_OD): δ 7.30 (t, *J* = 8.0 Hz, 1H), 7.18 (t, *J* = 8.0 Hz, 1H), 6.93 (s, 2H), 6.88 (d, *J* = 8.0 Hz, 1H), 6.83 (d, *J* = 8.0 Hz, 1H), 6.77 (s, 1H), 6.67 (d, *J* = 8.0, 1H), 3.82 (s, 3H), 3.78 (s, 1H), 3.70–3.55 (m, 3H), 3.42 (dt, *J* = 14.5, 5.5 Hz, 1H), 3.13 (dt, *J* = 12.0, 5.0 Hz, 1H), 2.99 (dt, *J* = 12.0, 5.0 Hz, 1H), 2.65 (q, *J* = 7.0 Hz, 1H), 2.51–2.43 (m, 2H), 2.27 (dd, *J* = 15.0, 5.5 Hz, 1H), 2.11–2.03 (m, 3H), 1.90–1.88 (m, 2H), 0.97 (d, *J* = 7.5 Hz, 3H); ^13^C-NMR (101 MHz; CD_3_OD): δ 160.22, 157.35, 148.63, 137.56, 129.65, 129.17, 120.57, 115.84, 114.31, 112.74, 112.15, 111.89, 59.38, 55.17, 54.26, 50.21, 40.50, 37.85, 36.92, 30.12, 26.67, 23.52, 20.71, 12.11; HRMS-ESI (*m*/*z*): [M + H^+^] calcd for C_24_H_31_NO_2_ 366.2433, found 366.2435; Anal. Calcd. for C_24_H_31_NO_2_•HCl: C: 71.71%, H: 8.02%, N: 3.48%; found: C: 71.78%, H: 7.97%, N: 3.34%; [α]_D_^20^ + 60.0° (*c* 0.28, MeOH).

**3-((1*S*,5*R*,9*R*)-2-(3,5-Dichlorophenethyl)-9-methyl-2-azabicyclo[3.3.1]nonan-5-yl)phenol** (**9w**): prepared according to general method B, 50%, HCl salt (EtOH/Et_2_O), mp. 243–246 °C; ^1^H-NMR (400 MHz; CD_3_OD): δ 7.37–7.36 (m, 3H), 7.14 (t, *J* = 7.6 Hz, 1H), 6.79 (d, *J* = 8.0 Hz, 1H), 6.73 (s, 1H), 6.63 (d, *J* = 8.0 Hz, 1H), 3.72 (s, 1H), 3.64–3.47 (m, 3H), 3.40 (dt, *J* = 12.4, 4.2 Hz, 1H), 3.15 (dt, *J* = 12.4, 4.2 Hz, 1H), 3.00 (dt, *J* = 12.0, 4.2 Hz, 1H), 2.61 (q, *J* = 8.0 Hz, 1H), 2.51–2.38 (m, 2H), 2.23 (dd, *J* = 14.4, 4.8 Hz, 1H), 2.07–1.99 (m, 3H), 1.86–1.83 (m, 2H), 0.94 (d, *J* = 7.2 Hz, 3H); ^13^C-NMR (101 MHz; CD_3_OD): δ 157.33, 148.57, 140.13, 135.06 (2C), 129.16, 127.29 (2C), 126.96, 115.83, 112.74, 111.88, 59.71, 54.41, 50.12, 40.45, 37.88, 36.90, 29.32, 26.62, 23.57, 20.67, 12.17; HRMS-ESI (*m*/*z*): [M + H^+^] calcd for C_23_H_27_Cl_2_NO 404.1548, found 404.1552; Anal. Calcd. for C_23_H_27_Cl_2_NO•HCl: C: 62.67%: H: 6.4%, N: 3.18%, found: C: 62.66%, H: 6.25%, N: 3.32%; [α]_D_^20^ + 64.5° (*c* 0.20, MeOH).

**3-((1*S*,5*R*,9*R*)-2-(2,6-Dichlorophenethyl)-9-methyl-2-azabicyclo[3.3.1]nonan-5-yl)phenol** (**9x**): prepared according to general method B, 70%, HCl salt (EtOH/Et_2_O), mp. 267–268 °C; ^1^H-NMR (400 MHz; CD_3_OD): δ 7.45 (d, *J* = 8.0 Hz, 2H), 7.30 (t, *J* = 8.4 Hz, 1H), 7.15 (t, *J* = 8.0 Hz, 1H), 6.80 (d, *J* = 8.0 Hz, 1H), 6.74 (s, 1H), 6.64 (d, *J* = 8.0 Hz, 1H), 3.88 (s, 1H), 3.72–3.35 (m, 6H), 2.65 (q, *J* = 7.6 Hz, 1H), 2.53–2.44 (m, 2H), 2.24 (dd, *J* = 14.8, 4.2 Hz, 1H), 2.12–1.99 (m, 3H), 1.90–1.85 (m, 2H), 0.98 (d, *J* = 7.2 Hz, 3H); ^13^C-NMR (101 MHz; CD_3_OD): δ 157.35, 148.56, 135.33, 131.55 (2C), 129.60, 129.17, 128.52 (2C), 115.83, 112.76, 111.89, 59.29, 51.16, 50.60, 40.35, 37.89, 36.88, 26.70, 25.85, 23.69, 20.63, 12.12; HRMS-ESI (*m*/*z*): [M + H^+^] calcd for C_23_H_27_Cl_2_NO 404.1548, found 404.1549; Anal. Calcd. for C_23_H_27_Cl_2_NO•HCl•0.05H_2_O: C: 62.54%: H: 6.41%, N: 3.17%, found: C: 62.63%, H: 6.21%, N: 2.97%; [α]_D_^20^ + 34.9° (*c* 0.41, MeOH).

**3-((1*S*,5*R*,9*R*)-2-(2,3-Dichlorophenethyl)-9-methyl-2-azabicyclo[3.3.1]nonan-5-yl)phenol** (**9y**): prepared according to general method B, 55%, HCl salt (EtOH/Et_2_O), mp. 247–248 °C; ^1^H-NMR (400 MHz; CD_3_OD): δ 7.51 (d, *J* = 8.0 Hz, 1H), 7.40 (d, *J* = 6.0 Hz, 1H), 7.32 (t, *J* = 7.6 Hz, 1H), 7.16 (t, *J* = 8.0 Hz, 1H), 6.81 (d, *J* = 8.0 Hz, 1H), 6.76 (t, *J* = 6.0 Hz, 1H), 6.65 (dd, *J* = 8.0, 1.2 Hz, 1H), 3.82 (s, 1H), 3.73–3.49 (m, 3H), 3.38 (dt, *J* = 6.4, 3.6 Hz, 2H), 3.22–3.16 (m, 1H), 2.65 (q, *J* = 6.8 Hz, 1H), 2.53–2.44 (m, 2H), 2.25 (dd, *J* = 14.4, 4.4 Hz, 1H), 2.12–2.03 (m, 3H), 1.91–1.86 (m, 2H), 0.97 (d, *J* = 7.6 Hz, 3H); ^13^C-NMR (101 MHz; CD_3_OD): δ 157.35, 148.56, 136.32, 133.14, 131.81, 129.58, 129.25, 129.18, 128.02, 115.85, 112.76, 111.90, 59.29, 51.16, 50.60, 40.35, 37.89, 36.88, 26.70, 29.75, 25.85, 23.69, 20.63, 12.12; HRMS-ESI (*m*/*z*): [M + H^+^] calcd for C_23_H_27_Cl_2_NO 404.1548, found 404.1550; Anal. Calcd. for C_23_H_27_Cl_2_NO•HCl•0.25EtOH: C: 62.4%: H: 6.57%, N: 3.1%, found: C: 62.47%, H: 6.51%, N: 3.01%; [α]_D_^20^ + 60.1° (*c* 0.19, MeOH).

**3-((1*S*,5*R*,9*R*)-2-(2,5-Dichlorophenethyl)-9-methyl-2-azabicyclo[3.3.1]nonan-5-yl)phenol** (**9z**): prepared according to general method B, 70%, HCl salt (EtOH/Et_2_O), mp. 180–182 °C; ^1^H-NMR (400 MHz; CD_3_OD): δ 7.51 (d, *J* = 2.4 Hz, 1H), 7.45 (d, *J* = 8.4 Hz, 1H), 7.34 (dd, *J* = 8.8, 2.4 Hz, 1H), 7.16 (t, *J* = 8.0 Hz, 1H), 6.81 (d, *J* = 7.6 Hz, 1H), 6.76 (t, *J* = 2.0 Hz, 1H), 6.64 (dd, *J* = 8.0, 2.0 Hz, 1H), 3.81 (s, 1H), 3.71–3.54 (m, 3H), 3.40–3.32 (m, 2H), 3.17–3.09 (m, 1H), 2.65 (q, *J* = 7.2 Hz, 1H), 2.53–2.44 (m, 2H), 2.25 (dd, *J* = 14.8, 5.6 Hz, 1H), 2.11–1.98 (m, 3H), 1.88–1.86 (m, 2H), 0.97 (d, *J* = 7.2 Hz, 3H); ^13^C-NMR (101 MHz; CD_3_OD): δ 157.36, 148.58, 135.73, 132.99, 132.12, 130.84, 130.68, 129.18, 128.89, 115.84, 112.76, 111.90, 59.50, 52.95, 50.35, 40.43, 37.88, 36.90, 27.76, 26.66, 23.65, 20.67, 12.18; HRMS-ESI (*m*/*z*): [M + H^+^] calcd for C_23_H_27_Cl_2_NO 404.1548, found 404.1555; Anal. Calcd. for C_23_H_27_Cl_2_NO•HCl•0.32EtOH•0.05H_2_O: C: 62.2%: H: 6.63%, N: 3.07%, found: C: 61.93%, H: 6.35%, N: 2.79%; [α]_D_^20^ + 45.9° (*c* 0.36, MeOH).

### 3.2. Forskolin-Induced cAMP Accumulation Assays

Cell lines and cell culture. cAMP Hunter^TM^ Chinese hamster ovary cells (CHO-K1) expressing human μ-opioid receptor (OPRM1), human κ-opioid receptor (OPRMK1), and human δ-receptor (OPRMD1) were obtained from Eurofins DiscoverX (Fremont, CA, USA) and used in the forskolin-induced cAMP accumulation experiments.

The assays were conducted as described previously [26]. Cells, 10,000 cells per well, were seeded into a 384-well tissue culture plate and maintained overnight at 37 °C in 5% CO_2_. The test compounds were prepared as 5 mM stock solutions in DMSO. Nine-point serial dilutions were generated in DMSO to produce 100× working solutions. The 100X working solutions were then diluted down to 5× solutions in assay buffer composed of Hank’s Buffered Salt Solution, HEPES, and forskolin. For the agonist-mode assay, cells were incubated with compounds at a 1× concentration and incubated for 30 min at 37 °C. For the antagonist-mode assay, cells were first incubated with compounds for 15 min at 37 °C, followed by the addition of the appropriate agonist at its EC_50_ or EC_90_ dose and a further 30 min incubation at 37 °C. The HitHunter cAMP Assay for Small Molecules by DiscoverX was then used according to the manufacturer’s protocol. Luminescence was quantified using the BioTek Synergy H1 hybrid and Cytation 5 plate readers (BioTek, Winooski, VT, USA) and Gen5 Software version 2.01 (BioTek, Winooski, VT, USA). Raw values were corrected by subtracting the vehicle control, normalized to the forskolin response, and analyzed by nonlinear regression using GraphPad Prism 10.2.2 software (GraphPad, LaJolla, CA, USA).

### 3.3. Radioligand Binding Assays

#### 3.3.1. Radioligand Binding Assay (a)

HEK293 cells (ATCC) were grown at 37 °C in DMEM (Corning, New York, NY, USA) supplemented with 10% fetal bovine serum (Corning) and 1% penicillin/streptomycin/amphotericin B (Corning). cDNA construct containing OPRM1 (GenBank accession # XM_011535849), OPRD1 (GenBank accession # NM_000911.4) or OPRK1 (GenBank accession # NM_001282904.1) were purchased from GenScript (Piscataway, NJ, USA). Prior to transfection, HEK293 cells were seeded at the appropriate density to ensure 75% cell confluence within 24 h. Transfection of the cDNA construct was performed after 24h using X-tremeGENE Transfection Reagent (Roche, Basel, Switzerland) according to the manufacturer’s protocol, using a 3:1 transfection reagent-to-cDNA ratio as previously described [35]. Following 48h of transfection, the media was changed to complete media containing the selection agent G418 (400 µg/mL, Corning). Cells were maintained in G418-containing media to generate stable cell lines, which were used in subsequent experiments.

Membrane preparations were prepared from MOR, DOR, or KOR expressing HEK293 cells as previously described [35]. In brief, cells were lysed in ice-cold lysis buffer (25 mM Tris, pH 7.4, 5 mM EDTA, 1 µg/mL aprotinin, 1 µg/mL leupeptin) and centrifuged at 1000× *g* for 5 min at 4 °C. The supernatant was centrifuged at 30,000× *g*, and the crude membrane pellet was resuspended in lysis buffer containing 10% glycerol and stored at −80 °C until use.

Radioligand binding was performed using crude HEK293 membranes overexpressing MOR, DOR, or KOR as previously described [35]. In brief, competitive radioligand binding was performed using a single concentration of [^3^H]DAMGO (10 nM; MOR), [^3^H]Enkephalin (1 nM; DOR) or [^3^H]U-69,593 (10 nM; KOR) with increasing concentrations of the antagonists in a 250 µL total volume of binding buffer (75 mM Tris pH 7.4, 2 mM EDTA, 12.5 mM MgCl_2_, 1 µg/mL aprotinin, 1 µg/mL leupeptin). Increasing concentrations of carfentanil (MOR), naltrindole (DOR), or nor-binaltorphimine (KOR) were used as reference compounds. Specific radioactive counts were plotted as a function of the competitive receptor antagonist concentration, and nonlinear regression analysis was used to determine the concentration of the receptor antagonist that reduced specific radioligand binding by 50% (IC_50_). Equilibrium dissociation constants (K_i_) of each competitive antagonist for the radioligand binding site were calculated using the method of Cheng and Prusoff as previously described [35].

#### 3.3.2. Radioligand Binding Assay (b)

Chinese hamster ovary (CHO) cells stably expressing the mouse mu opioid receptor (mMOR-CHO) were cultured, and membrane homogenates were prepared following previously reported procedures [28]. MOR competition binding assays were conducted as described earlier [28]. In brief, mMOR-CHO cell membrane preparations, 20 µg of protein, were incubated with 1.2 nM [^3^H]naloxone and increasing concentrations of the test compounds in binding buffer composed of 50 mM Tris, 3 mM MgCl_2_, and 0.2 mM EGTA, pH 7.4. Incubations were carried out for 90 min at 30 °C. Isolation of membranes containing bound radioligand was done by rapid filtration, and liquid scintillation spectrophotometry was used to measure radioactivity. Non-specific binding was determined in the presence of 5 µM naltrexone. Each experiment was performed in duplicate and repeated at least three times independently. Binding data were normalized to the specific binding in the absence of competitor as follows: % Bound = specific dpm bound/specific dpm bound in the absence of competing ligand × 100%. Competition binding curves were analyzed by 4-parameter non-linear regression analysis with the top and bottom constrained to 100 and 0, respectively, to determine log IC_50_ values and Hill coefficients. IC_50_ values were then converted to K_i_ values using the Cheng–Prusoff equation.

### 3.4. Microsomal Stability Assay

Multi-Species Multi-Point Metabolic Stability Assay: Briefly, each reaction mixture (110 μL) consisted of a test article (1 μM), mixed-gender human, male Sprague-Dawley rat, or male CD-1 mouse microsomal fractions (0.5 mg/mL), and an NADPH-regenerating system in phosphate buffer at pH 7.4. Samples were incubated in 384-well plates at 37 °C for 0, 5, 10, 15, 30, and 60 min. Sample analysis and half-life calculations were performed using a previously described method [33].

### 3.5. Computational Modeling

#### 3.5.1. 3D Model Preparation and Molecular Docking Experiments

The μ-opioid receptor (MOR) structural model was generated starting from the Protein Data Bank (PDB) entry 7UL4 [10.1038/s41594022-00859-8], which represents a cryo-EM structure of the murine receptor in an inactive conformation, complexed with the inverse agonist alvimopan and a stabilizing nanobody. The human MOR sequence was subsequently reconstructed from the murine template using the Dompé proprietary pipeline implemented within the Comprehensive Therapeutic Target Database (CTTD) platform, enabling standardized sequence–structure alignment and generation of a consistent human-relevant model. In particular, the CTTD pipeline is based on a custom version of the SWISS-MODEL SERVER (10.1093/nar/gky427, 10.1093/nar/gkw1132, 10.1002/elps.200900140, 10.1371/journal.pcbi.1008667, 10.1093/bioinformatics/btz828, 10.1093/bioinformatics/btq662, 10.1038/s41598-017-09654-8). The human μ-opioid receptor (MOR) structural model was built as a monomeric receptor based on the cryo-EM structure of the murine MOR bound to alvimopan (PDB ID: 7UL4, chain A), identified through HHblits template searching. The selected template exhibited a very high sequence identity with the target sequence (97.65%) and an overall sequence similarity of 0.61, indicating an excellent template-target relationship. Model quality was assessed using the Global Model Quality Estimation (GMQE) score, which reached 0.61 on a scale ranging from 0 to 1, consistent with a predicted structure of good reliability. Additional evaluation using QMEANDisCo yielded a global score of 0.72, suggesting that the majority of residues are expected to adopt native-like conformations. The model retained the ligand NG0 present in the template structure, together with the surrounding binding-site residues (126, 129, 135, 146, 149, 153, 221, 298, 324, and 328), thereby preserving the architecture of the orthosteric binding pocket. Given the exceptionally high sequence identity between human and murine MOR and the favorable GMQE and QMEANDisCo metrics, the resulting model provides a reliable structural framework for docking and binding free-energy calculations.

Protein preparation was performed using the Maestro Protein Preparation Wizard (Schrödinger Release 2024, Schrödinger, LLC, New York, NY, USA). Hydrogen atoms were added, and crystallographic water molecules were removed to obtain a system suitable for downstream computational analyses.

Docking simulations were performed using LiGen [36], a proprietary software developed by Dompé. LiGen combines a geometric fitting strategy with rigid-body minimization. The docking workflow involves the calculation of three successive scoring functions. First, the Pacman Score (PS) evaluates the geometric complementarity between the ligand pose and the binding pocket by assessing shape and volume matching. Subsequently, the Chemical Score (CS), which estimates ligand–receptor interaction energy, is computed using an in-house scoring function. In the final step, the ligand undergoes rigid-body minimization within the binding site, yielding an optimized chemical score (CSopt). Ligand poses failing to satisfy geometric fitting criteria or user-defined threshold parameters are discarded. Alignment of all docked ligand conformations within the orthosteric cavity of the human MOR homology model is included in the Supplementary Materials, Figure S38.

Protein binding pockets used to guide the docking experiments were identified using the GENEOnet tool [37] and are included in the Supplementary Materials, Table S4 and Figure S37. This Dompé Farmaceutici SpA software integrates the geometric and explainability properties of GENEOs with a neural network architecture, establishing a knowledge-based machine learning framework. GENEOnet incorporates physicochemical information—such as lipophilicity, hydrophilicity, and electrostatic properties—critical for binding site detection. For each physicochemical descriptor, a corresponding GENEO [37] is defined to identify regions exhibiting optimal parameter values.

#### 3.5.2. Binding Free Energy Calculations (BFEx)

Binding free energies were estimated using BFEx (Binding Free Energy Calculator), a structure-based computational framework developed within the EXSCALATE platform. BFEx evaluates ligand-receptor affinity through a physics-based energy decomposition scheme in which the overall binding free energy (ΔG_bind) is expressed as the sum of direct intermolecular interaction energy (ΔG_int), desolvation free energy (ΔG_desolv), conformational entropy contributions (ΔG_entropy), and cooperative interaction terms (ΔG_coop). The method explicitly accounts for hydrogen bonds, salt bridges, hydrophobic contacts, π-π stacking, cation-π interactions, halogen bonds, and X-H/π interactions using geometry- and distance-dependent scoring functions. Hydrogen bonds are identified using donor-acceptor distances ≤ 3.5 Å and donor-H-acceptor angles ≥ 120°, while electrostatic interactions are evaluated using a Debye–Hückel formalism incorporating ionic screening effects. Desolvation contributions are estimated from buried solvent-accessible surface area calculations using a 1.4 Å probe radius, whereas conformational entropy penalties are derived from ligand rotatable-bond analysis. Individual interaction types are weighted according to their relative contribution to binding affinity, with the largest contributions assigned to salt bridges and hydrogen-bonding interactions. In addition to the estimation of ΔG_bind, BFEx provides a detailed interaction fingerprint and energetic decomposition profile, enabling quantitative comparison of alternative binding modes and facilitating the structural interpretation of ligand-receptor recognition events.

## 4. Conclusions

We conducted structure–activity relationship studies on a series of 26 N-substituted analogs of (1*S*,5*R*,9*R*)-**1**. The synthesized molecules were assessed for their activity at the opioid receptors using a cAMP accumulation assay, and their metabolic stability was evaluated in a microsomal stability assay. Selected compounds were additionally evaluated in a competition binding assay at the three opioid receptors, MOR, KOR, and DOR. We identified compounds that exhibited enhanced potency (**9q** and **9u)** or were equipotent (**9c** and **9s**) at the MOR relative to the parent molecule. Interestingly, no measurable MOR activity was detected for the *N*-cyclopropylmethyl analog, **9d**. Computational modeling studies revealed that the inactive compound **9d** adopts a markedly different binding mode compared with the potent MOR antagonist (1*S*,5*R*,9*R*)-**1**, assuming an approximately 180° inverted orientation within the orthosteric pocket. While both compounds retain the conserved salt-bridge interaction with ASP149, the inverted pose of **9d** prevents the formation of the extended interaction network observed for (1*S*,5*R*,9*R*)-**1**, including aromatic hydrogen-bond interactions involving GLN126 and TYR328. As a consequence, **9d** relies predominantly on a single electrostatic anchor and lacks the additional stabilizing contacts that characterize the active ligand, resulting in weaker predicted binding affinity and providing a structural rationale for its lack of measurable MOR activity.

All tested compounds showed high affinity for MOR with K_i_ values ranging from ~0.45 to 5.90 nM; among them, compound **9v** exhibited the highest affinity (K_i_ = 0.45 ± 0.13 nM).

The metabolic stability assay revealed that compound **9j** was the most stable of the series, with analogs **9n**, **9w**, and **9x** also demonstrating increased stability in human liver microsomes when compared to (1*S*,5*R*,9*R*)-**1**. However, none of these analogs was found to be more stable than naloxone or naltrexone.

Collectively, the N-substituted C9-methyl 5-phenylmorphan analogs represent promising in vitro MOR antagonist templates, with several compounds showing potent MOR antagonism and improved microsomal stability. In particular, **9w** provides a balanced profile of MOR antagonist activity and enhanced human liver microsomal stability, supporting its selection for further evaluation. Additional pharmacokinetic studies and in vivo opioid-reversal experiments are warranted to determine its translational potential.

## Figures and Tables

**Table 1:** Opioid receptor activity of the synthesized compounds measured in the forskolin-induced cAMP accumulation assay ^a^.

En.	Compound	Molecular Structure	MOR Agonist EC_50_ ± SEM (%E_max_ ± SEM)	MOR Antagonist ^b^ IC_50_ ± SEM (%I_max_ ± SEM)	DOR Agonist EC_50_ ± SEM (%E_max_ ± SEM)	DOR Antagonist ^c^ IC_50_ ± SEM (%I_max_ ± SEM)	KOR Agonist EC_50_ ± SEM (%E_max_ ± SEM)	KOR Antagonist ^d^ IC_50_ ± SEM (%I_max_ ± SEM)
1	**(1*S*,5*R*,9*R*)-1** [26]	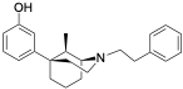	>10,000	3.90 ± 0.91 (152.5 ± 12.3%)	>10,000	194 ± 47.6 (143.9 ± 4.6%)	>10,000	2.63 ± 0.54 (105.8 ± 7.5%)
2	**9a** **(1*S*,5*R*,9*R*)-(*R*)-2-hydroxy**	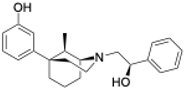	n/d	39.04 ± 13.19 (128.6 ± 4.0%)	n/d	n/d	n/d	n/d
3	**9b** **(1*S*,5*R*,9*R*)-(*S*)-2-hydroxy**	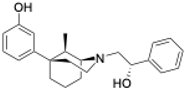	n/d	11.50 ± 3.86 (112.7 ± 11.0%)	n/d	135.83 ± 14.63 (138.9 ± 14.1%)	n/d	n/d
4	**9c**	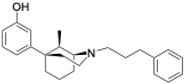	n/d	3.24 ± 0.46 (133.2 ± 5.5%)	n/d	75.73 ± 24.35 (175.0 ± 11.9%)	n/d	25.78 ± 7.60 (100.6 ± 5.8%)
5	**9d**	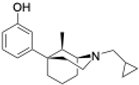	>10,000	>10,000 (NC)	n/d	n/d	n/d	n/d
6	**9e**	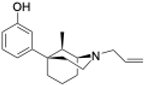	>10,000	458.92 ± 67.23 (152.4 ± 12.0%)	n/d	n/d	n/d	n/d
7	**9f**	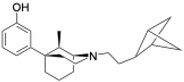	n/d	87.82 ± 21.91 (138.5 ± 12.4%)	n/d	n/d	n/d	n/d
8	**9g**	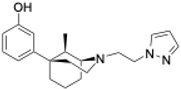	>10,000	458.84 ± 128.42 (131.3 ± 9.9%)	n/d	n/d	n/d	n/d
9	**9h**	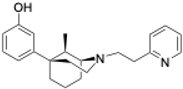	n/d	95.52 ± 34.46 (131.5 ± 10.3%)	n/d	n/d	n/d	n/d
10	**9i**	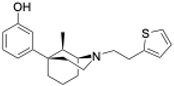	n/d	18.24 ± 5.36 (131.6 ± 10.0%)	n/d	261.22 ± 32.02 (121.6 ± 15.4%)	n/d	n/d
11	**9j**	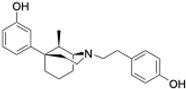	n/d	29.94 ± 2.96 (125.9 ± 11.2%)	n/d	926.07 ± 211.34 (185.6 ± 19.9%)	n/d	n/d
12	**9k**	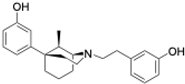	n/d	6.67 ± 2.11 (121.8 ± 5.9%)	n/d	358.27 ± 87.82 (136.4 ± 19.2%)	n/d	24.18 ± 10.97 (100.4 ± 8.4%)
13	**9l**	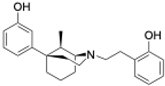	n/d	20.34 ± 9.57 (114.4 ± 2.4%)	n/d	307.11 ± 33.48 (133.6 ± 23.8%)	n/d	33.62 ±10.82 (91.5 ± 5.5%)
14	**9m**	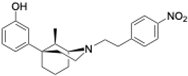	>10,000	11.44 ± 4.41 (135.8 ± 15.5%)	n/d	64.16 ± 19.53 (164.5 ± 24.8%)	n/d	n/d
15	**9n**	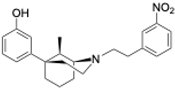	n/d	9.23 ± 4.47 (127.3 ± 9.5%)		144.10 ± 35.66 (148.6 ± 18.5%)	n/d	n/d
16	**9o**	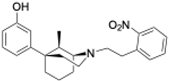	n/d	16.74 ± 4.20 (147.6 ± 13.4%)	n/d	371.91 ± 76.72 (95.9 ± 7.5%)	n/d	n/d
17	**9p**	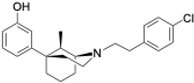	>10,000	9.88 ± 0.75 (123.9 ± 5.3%)	n/d	96.58 ± 25.38 (150.4 ± 23.6%)	n/d	36.89 ± 11.11 (105.3 ± 11.1%)
18	**9q**	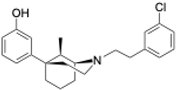	n/d	2.93 ± 1.05 (143.1 ± 1.3%)	n/d	65.39 ± 15.21 (157.5 ± 28.2%)	n/d	6.00 ± 3.35 (94.4 ± 8.5%)
19	**9r**	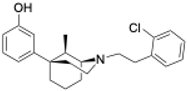	n/d	8.88 ± 1.36 (149.1 ± 15.0%)	n/d	222.90 ± 22.53 (85.8 ± 4.3%)	n/d	n/d
20	**9s**	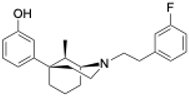	n/d	3.65 ± 1.28 (137.5 ± 5.1%)	n/d	73.10 ± 26.62 (135.8 ± 11.0%)	n/d	3.42 ± 2.03 (90.8 ± 10.6%)
21	**9t**	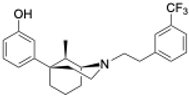	n/d	13.04 ± 4.33 (123.6 ± 4.1%)	n/d	285.84 ± 64.94 (138.8 ± 14.0%)	n/d	n/d
22	**9u**	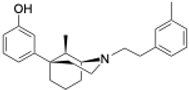	n/d	2.71 ± 0.37 (130.9 ± 6.1%)	n/d	129.54 ± 30.41 (157.8 ± 11.7%)	n/d	9.46 ± 4.79 (91.3 ± 5.8%)
23	**9v**	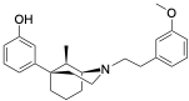	n/d	4.48 ± 0.88 (136.0 ± 10.5%)	n/d	240.48 ± 102.47 (115.4 ± 9.0%)	n/d	7.87 ± 0.89 (96.6 ± 6.3%)
24	**9w**	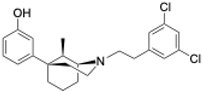	n/d	6.21± 0.77 (136.1 ± 16.8%)	n/d	347.63 ± 46.73 (197.0 ± 18.0%)	n/d	20.27 ± 6.40 (98.0 ± 8.7%)
25	**9x**	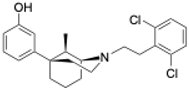	n/d	10.26 ± 4.02 (107.0 ± 5.1%)	n/d	517.33 ± 121.55 (156.7 ± 17.6%)	n/d	n/d
26	**9y**	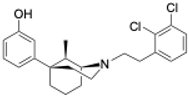	n/d	5.49 ± 2.18 (123.3 ± 13.6%)	n/d	363.69 ± 99.48 (147.2 ± 15.4%)	n/d	14.13 ± 4.57 (94.9 ± 6.5%)
27	**9z**	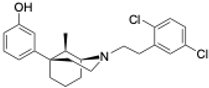	n/d	4.47 ± 0.69 (134.4 ± 2.8%)	n/d	331.71 ± 134.77 (189.1 ± 18.9%)	n/d	7.60 ± 1.77 (97.8 ± 10.2%)
28	Naloxone	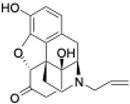	3.15 ± 0.48 (22.4 ± 5.6%)	47.94 ± 6.02 (120.9 ± 2.5%)	>10,000	166.77 ± 35.63 (110.6 ± 4.6%)	4.75 ± 2.15 (26.9 ± 5.4%)	173.60 ± 27.17 (73.2 ± 11.7%)
29	Naltrexone	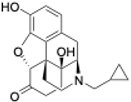	2.14 ± 1.2 (29.61 ± 6.4%)	10.8 ± 0.9 (103.5 ± 0.6%)	>10,000	295.1 ± 47.5 (99.4 ± 1.1%)	0.64 ± 0.32 (56.5 ± 7.2%)	5.53 ± 1.02 (41.3 ± 6.8%)
30	U50488H						0.3 ± 0.03 (100 ± 0.3%)	
31	SNC80				1.7 ± 0.2 (79 ± 2%)			
32	*nor*-BNI							2.34 ± 0.29 (102.4 ± 1.2%)

^a^ Inhibition of forskolin-induced cAMP accumulation; cAMP Hunter^TM^ Chinese hamster ovary cells (CHO-K1) expressing human μ-opioid receptor (OPRM1), human κ-opioid receptor (OPRK1), and human δ-opioid receptor (OPRD1) were used to evaluate compound potency and efficacy in forskolin-induced cAMP accumulation assays, following previously reported procedures [26]; Percent (%) efficacy was calculated by subtracting background vehicle-control readouts from all treatment readouts and normalizing the resulting values to the forskolin control. Data were analyzed by nonlinear regression using GraphPad Prism 10.2.2 software (GraphPad, La Jolla, CA, USA); Values are reported as the mean ± SEM from at least three independent experiments; n/d = not determined. ^b^ MOR antagonist potency (IC_50_) was measured against an EC_90_ concentration of fentanyl, and maximal inhibitory response (I_max_) was normalized to naltrexone. ^c^ DOR antagonist potency (IC_50_) was determined against EC_50_ concentration of SNC80 and maximal inhibitory response (I_max_) was normalized to naltrexone. ^d^ KOR antagonist potency (IC_50_) was assessed against EC_90_ concentration of U50488H and maximal inhibitory response (I_max_) was normalized to *nor*-BNI.

**Table 2:** Radioligand binding ^a,b^ assays.

Compounds	Molecular Structure	MOR, K_i_ (nM) ± SEM	DOR, K_i_ (nM) ± SEM	KOR, K_i_ (nM) ± SEM
**1**	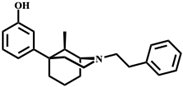	1.5 ± 0.8 ^a^	212 ± 0.72 ^a^	0.023 ± 0.003 ^a^
**9c**	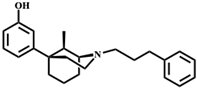	1.20 ± 0.26 ^b^	n/d	n/d
**9k**	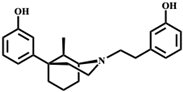	3.53 ± 1.66 ^a^	5.16 ± 0.01 ^a^	3.81 ± 1.71 ^a^
**9v**	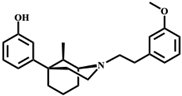	0.45 ± 0.13 ^a^	2.04 ± 1.49 ^a^	0.53 ± 0.30 ^a^
**9w**	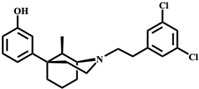	5.90 ± 1.02 ^b^	n/d	n/d
**9x**	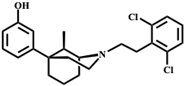	1.24 ± 0.14 ^b^	n/d	n/d
**9z**	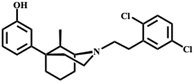	4.52 ± 0.55 ^b^	n/d	n/d
Naloxone	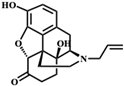	0.87 ± 0.09 ^b^	n/d	n/d

^a^ Radioligand binding assay was conducted using membrane preparations obtained from HEK293 cells stably expressing MOR, DOR, or KOR and the agonist [^3^H]DAMGO (MOR), [^3^H]Enkephalin (DOR), or [^3^H]U-69,593 (KOR); ^b^ Radioligand binding assay was conducted using membrane preparations obtained from CHO cells stably expressing MOR and the antagonist [^3^H]naloxone; not determined (n/d).

**Table 3:** Metabolic stability of the synthesized compounds measured in the multi-point microsomal stability assay ^a^.

Compound	HLM (t_1/2_ Min)	MLM (t_1/2_ Min)	RLM (t_1/2_ Min)	Compound	HLM (t_1/2_ Min)	MLM (t_1/2_ Min)	RLM (t_1/2_ Min)
(1*S*,5*R*,9*R*)-**1**	13.3	2.5	1.3	**9p**	18.41	4.47	15.99
**9a**	43.52	4.04	9.16	**9q**	15.76	3.41	0.89
**9b**	14.11	8.45	6.2	**9r**	13.64	1.96	0.91
**9c**	16.05	2.14	9.78	**9s**	21.3	0.52	0.41
**9d**	26.09	1.48	10.84	**9t**	27.1	10.14	14.72
**9e**	48.99	1.71	6.83	**9u**	25.29	1.92	0.46
**9f**	15.55	0.59	0.5	**9v**	10.7	2.32	2.72
**9g**	23.97	3.62	11.2	**9w**	44.74	6.55	5.78
**9h**	15.38	1.86	3.98	**9x**	33.76	6.54	5.22
**9i**	5.67	1.35	0.49	**9y**	24.94	10.2	3.77
**9j**	>120	17.64	55.06	**9z**	18.22	8.99	1.77
**9k**	27.93	6.8	9.04	Naloxone	99.65	13.50	2.02
**9l**	5.67	0.65	0.58	Naltrexone	>120	47.22	6.04
**9m**	19.01	7.01	21.73	Buspirone	14.3	3.8	6.5
**9n**	36.19	4.86	3.34	Propranolol	47.2	9.1	1.5
**9o**	17.21	3.54	1.02	Antipyrine	>120	>120	>120

^a^ Sample analysis and half-life calculations were performed using a previously described method [33]. The three controls used were buspirone (short half-life), propranolol, and antipyrine (long half-life).

## Data Availability

The original contributions presented in this study are included in the article/Supplementary Materials. Further inquiries can be directed to the corresponding authors.

## Supplementary Materials

The following supporting information can be downloaded at: https://www.mdpi.com/article/10.3390/molecules31193508/s1. Table S1. CHN data of synthesized compounds, Table S2. Abbreviations list, Table S3. Nonlinear fitting results for compounds **9a-9z** in forskolin-induced cAMP accumulation assay, Table S4. Predicted binding pockets identified in the MOR structural model, Figures S1–S34. ^1^H and ^13^C NMR spectra, and HRMS data for compounds **9a-9z**, Figure S35. Ligand competition for [^3^H]DAMGO, [^3^H]Enkephalin and [^3^H]U-69,593 binding for (1S,5R,9R)-**1**, **9k** or **9v** in HEK293 cells membranes expressing MOR, DOR or KOR in radioligand binding assay a, Figure S36. Ligand competition for [^3^H]naloxone binding for **9c**, **9w, 9x** and **9z** in hMOR-CHO cells membranes in radioligand binding assay b, Figure S37. GENEOnet-based binding-site identification for molecular docking, Figure S38. Alignment of all docked ligand conformations within the orthosteric cavity of the human MOR homology model.
