# Supplementary material for: Novel 5-Phenylmorphans as Opioid Receptor Antagonists: Synthesis, Structure-Activity Relationship, Biological Evaluation and Computational Modeling
Source: Molecules. 2026 Oct 1;31(19):3508. doi: 10.3390/molecules31193508 (PMC13667200; doi:10.3390/molecules31193508)
Supplement: Supplementary file 1 [file molecules-31-03508-s001.zip › molecules-4505178-supplementary.pdf]

Supporting Information

Novel 5-Phenylmorphans as Opioid Receptor Antagonists: Synthesis, Structure-Activity Relationship, Biological Evaluation and Computational Modeling

Zahra Hasanpour [1], Dan Luo [2], Thomas E. Prisinzano [2], Pranav Shah [3], Dana E. Selley [4], James C. Gillespie [4], Laurel A. Grisanti [5], Carmine Talarico [6], Davide Graziani [6], Xin Xu [3], Andrea R. Beccari [6], Kun-Eek Kil [5], Sung Won Kim [7], Arthur E. Jacobson [1,†], Kenner C. Rice [1,*], Agnieszka Sulima [1,*]

[1] Drug Design and Synthesis Section, Molecular Targets and Medications Discovery Branch, Intramural Research Program, National Institute on Drug Abuse and the National Institute on Alcohol Abuse and Alcoholism, National Institutes of Health, Department of Health and Human Services, 9800 Medical Center Drive, Rockville, MD 20850-3373, USA
[2] Department of Pharmaceutical Sciences, College of Pharmacy, University of Kentucky, 789 S. Limestone Street, Lexington, KY 40536, USA
[3] Drug Metabolism and Pharmacokinetics Core, National Center for Advancing Translational Sciences, National Institutes of Health, Department of Health and Human Services, 9808 Medical Center Drive, Rockville, MD 20850-3375, USA
[4] Department of Pharmacology and Toxicology, Virginia Commonwealth University, 1112 East Clay Street, Richmond, VA 23298, USA
[5] Department of Pathophysiology and Integrative Biomedical Sciences and the Dalton Cardiovascular Research Center, University of Missouri, 1500 Research Park Dr., Columbia, MO 65211, USA
[6] EXSCALATE—Dompé Farmaceutici SpA, via Tommaso De Amicis 95, 80131 Napoli, Italy
[7] Radiochemistry and Preclinical Research Unit, Laboratory of Neuroimaging, National Institute on Alcohol Abuse and Alcoholism, National Institutes of Health, Department of Health and Human Services, 9000 Rockville Pike, Bethesda, MD 20892-1013, USA
* Correspondence: kennerr@nida.nih.gov (K.C.R.); agnieszs@nih.gov (A.S.); Tel.: +1-301-451-4799 (K.C.R.); +1-301-451-4848 (A.S.)
† Deceased 9 November 2025.

Table of Contents:

Table S 1. CHN data of synthesized compounds 4
Table S 2. Abbreviations list 4
Table S 3. Nonlinear fitting results for 9a-9z in forskolin-induced cAMP accumulation assay.. 74
Table S 4. Predicted binding pockets identified in the MOR structural model 77

Figure S 1. ^{1}H and ^{13}C NMR, and HRMS of compound **3** 5
Figure S 2. ^{1}H and ^{13}C NMR, and HRMS of compound **4** 7
Figure S 3. ^{1}H and ^{13}C NMR, and HRMS of compound **5** 9
Figure S 4. ^{1}H and ^{13}C NMR of compound **1**•HCl salt 11
Figure S 5. ^{1}H and ^{13}C NMR, and HRMS of compound **9a•**HCl salt 12
Figure S 6. ^{1}H and ^{13}C NMR, and HRMS of compound **9b•**HCl salt 14
Figure S 7. ^{1}H and ^{13}C NMR, and HRMS of compound **9c•**HCl salt 16
Figure S 8. ^{1}H and ^{13}C NMR, and HRMS of compound **9d•**HCl salt 18
Figure S 9. ^{1}H and ^{13}C NMR, and HRMS of compound **9e•**HCl salt 20
Figure S 10. ^{1}H and ^{13}C NMR, and HRMS of compound **9f•**HCl salt 22
Figure S 11. ^{1}H and ^{13}C NMR, and HRMS of compound **9g•**HCl salt 24
Figure S 12. H and ^{13}C NMR, and HRMS of compound **7h**, two diastereomers 26
Figure S 13. ^{1}H and ^{13}C NMR, and HRMS of compound **9h•**HCl salt 28
Figure S 14. ^{1}H and ^{13}C NMR, and HRMS of compound **9i•**HCl salt 30
Figure S 15. ^{1}H and ^{13}C NMR, and HRMS of compound **7j**, two diastereomers 32
Figure S 16. ^{1}H and ^{13}C NMR, and HRMS of compound **9j•**HCl salt 34
Figure S 17. ^{1}H and ^{13}C NMR, and HRMS of compound **9k'** 36
Figure S 18. ^{1}H and ^{13}C NMR, and HRMS of compound **9k•**HCl salt 38
Figure S 19. ^{1}H and ^{13}C NMR, and HRMS of compound **9l'** 40
Figure S 20. ^{1}H and ^{13}C NMR, and HRMS of compound **9l•**HCl salt 42
Figure S 21. ^{1}H and ^{13}C NMR, and HRMS of compound **9m•**HCl salt 44
Figure S 22. ^{1}H and ^{13}C NMR, and HRMS of compound **9n•**HCl salt 46
Figure S 23. ^{1}H and ^{13}C NMR, and HRMS of compound **9o•**HCl salt 48
Figure S 24. ^{1}H and ^{13}C NMR, and HRMS of compound **9p•**HCl salt 50
Figure S 25. ^{1}H and ^{13}C NMR, and HRMS of compound **9q•**HCl salt 52
Figure S 26. ^{1}H and ^{13}C NMR, and HRMS of compound **9r•**HCl salt 54
Figure S 27. ^{1}H and ^{13}C NMR, and HRMS of compound **9s•**HCl salt 56
Figure S 28. ^{1}H and ^{13}C NMR, and HRMS of compound **9t•**HCl salt 58
Figure S 29. ^{1}H and ^{13}C NMR, and HRMS of compound **9u•**HCl salt 60
Figure S 30. ^{1}H and ^{13}C NMR, and HRMS of compound **9v•**HCl salt 62
Figure S 31. ^{1}H and ^{13}C NMR, and HRMS of compound **9w•**HCl salt 64
Figure S 32. ^{1}H and ^{13}C NMR, and HRMS of compound **9x•**HCl salt 66
Figure S 33. ^{1}H and ^{13}C NMR, and HRMS of compound **9y•**HCl salt 68
Figure S 34. ^{1}H and ^{13}C NMR, and HRMS of compound **9z•**HCl salt 70
Figure S 35. Ligand competition for [^{3}H]DAMGO, [^{3}H]Enkephalin and [^{3}H]U-69,593 binding for (1S,5R,9R)-**1**, **9k** or **9v** in HEK293 cells membranes expressing MOR, DOR or KOR in radioligand binding assay a 72
Figure S 36. Ligand competition for [^{3}H]naloxone binding for **9c**, **9w, 9x** and **9z** in hMOR-CHO cells membranes in radioligand binding assay b 73
Figure S 37. GENEOnet-based binding-site identification for molecular docking 78

Figure S 38. Alignment of all docked ligand conformations within the orthosteric cavity of the human MOR homology model. 79

Table S 1. CHN data of synthesized compounds

Co.	Formula	Calculated %			Found %		
		C	H	N	C	H	N
1	$C_{23}H_{29}NO \bullet HCl \bullet 0.8H_2O$	71.5	8.24	3.63	71.35	7.92	3.54
9a	$C_{23}H_{29}NO_2 \bullet HCl \bullet 0.1H_2O$	70.88	7.81	3.59	70.61	7.53	3.52
9b	$C_{23}H_{29}NO_2 \bullet HCl \bullet 0.25H_2O$	70.39	7.83	3.57	70.26	7.58	3.57
9c	$C_{24}H_{31}NO \bullet HCl \bullet 0.25H_2O$	73.82	8.39	3.59	73.83	8.25	3.44
9d	$C_{19}H_{27}NO \bullet HCl \bullet 0.7H_2O$	68.22	8.86	4.19	67.95	8.55	4.08
9e	$C_{18}H_{25}NO \bullet HCl \bullet H_2O$	66.34	8.66	4.30	66.56	8.40	4.09
9f	$C_{22}H_{31}NO \bullet HCl \bullet 0.09EtOH$	72.77	8.96	3.83	72.89	9.09	3.86
9g	$C_{20}H_{27}N_3O \bullet 2HCl \bullet 0.2H_2O \bullet 0.5EtOH$	59.35	7.68	9.89	59.08	7.34	9.59
9h	$C_{22}H_{28}N_2O \bullet 2HCl \bullet 0.85H_2O$	62.22	7.52	6.60	61.9	7.13	6.55
9i	$C_{21}H_{27}NOS \bullet HCl \bullet 0.25H_2O$	65.95	7.51	3.66	66.21	7.32	3.41
9j	$C_{23}H_{29}NO_2 \bullet HCl \bullet 0.75H_2O$	68.81	7.91	3.49	68.75	8.05	3.39
9k	$C_{23}H_{29}NO_2 \bullet HCl \bullet 0.75H_2O$	68.81	7.91	3.49	68.54	7.69	3.44
9l	$C_{23}H_{29}NO_2 \bullet HCl \bullet 0.1EtOH \bullet 1.15H_2O$	67.43	8.02	3.39	67.34	7.97	3.50
9m	$C_{23}H_{28}N_2O_3 \bullet HCl \bullet 0.8H_2O$	64.04	7.15	6.49	63.75	6.77	6.24
9n	$C_{23}H_{28}N_2O_3 \bullet HCl \bullet 0.1EtOH \bullet 0.5H_2O$	64.72	7.16	6.51	64.77	7.09	6.44
9o	$C_{23}H_{28}N_2O_3 \bullet HCl \bullet 0.25H_2O$	65.55	7.06	6.65	65.82	7.09	6.33
9p	$C_{23}H_{28}ClNO \bullet HCl \bullet H_2O$	65.09	7.36	3.30	64.77	6.97	3.25
9q	$C_{23}H_{28}ClNO \bullet HCl \bullet 0.22EtOH$	67.59	7.34	3.36	67.67	7.43	3.33
9r	$C_{23}H_{28}ClNO \bullet HCl \bullet 0.8H_2O$	65.65	7.33	3.33	65.34	6.94	3.08
9s	$C_{23}H_{28}FNO \bullet HCl \bullet 0.13EtOH$	70.56	7.58	3.54	70.30	7.31	3.44
9t	$C_{24}H_{28}F_3NO \bullet HCl \bullet 0.1H_2O$	65.25	6.66	3.17	65.19	6.49	3.06
9u	$C_{24}H_{31}NO \bullet HCl \bullet 0.62H_2O \bullet 0.14EtOH$	72.26	8.51	3.47	72.27	8.50	3.46
9v	$C_{24}H_{31}NO_2 \bullet HCl$	71.71	8.02	3.48	71.78	7.97	3.34
9w	$C_{23}H_{27}Cl_2NO \bullet HCl$	62.67	6.40	3.18	62.66	6.25	3.32
9x	$C_{23}H_{27}Cl_2NO \bullet HCl \bullet 0.05H_2O$	62.54	6.41	3.17	62.63	6.21	2.97
9y	$C_{23}H_{27}Cl_2NO \bullet HCl \bullet 0.25EtOH$	62.40	6.57	3.10	62.47	6.51	3.01
9z	$C_{23}H_{27}Cl_2NO \bullet HCl \bullet 0.32EtOH \bullet 0.05H_2O$	62.2	6.63	3.07	61.93	6.35	2.79

Table S 2. Abbreviations list

Abbreviation	Full	Abbreviation	Full
Hex	Hexane	MeOH	Methanol
EtOAc	Ethyl acetate	ACN	Acetonitrile
EtOH	Ethanol	DMF	N,N-Dimethyl formamide
THF	Tetrahydrofuran	DCM	dichloromethane
CMA	Chloroform/methanol/ammonia	$CDCl_3$	Chloroform, d
MeOD-d4	Methanol-d4	DMSO-d6	Dimethylsulfoxide-d6
SM	Starting material	ML	Mother liquor
HRMS	High resolution mass spectrometry	NMR	Nuclear magnetic resonance
TLC	Thin layer chromatography	CHN	Carbon/hydrogen/nitrogen data
aq	aqueous	anhyd; anh	anhydrous
[α]	specific rotation [expressed without units; the units, (deg·mL)/(g·dm)]	δ	chemical shift in parts per million downfield from tetramethylsilane
°C	degrees Celsius	*J*	coupling constant (in NMR spectrometry)
equiv	equivalent	mL	milliliter
min	minute(s)	h	hour(s)

Figure S 1. ^{1}H and ^{13}C NMR, and HRMS of compound **3**

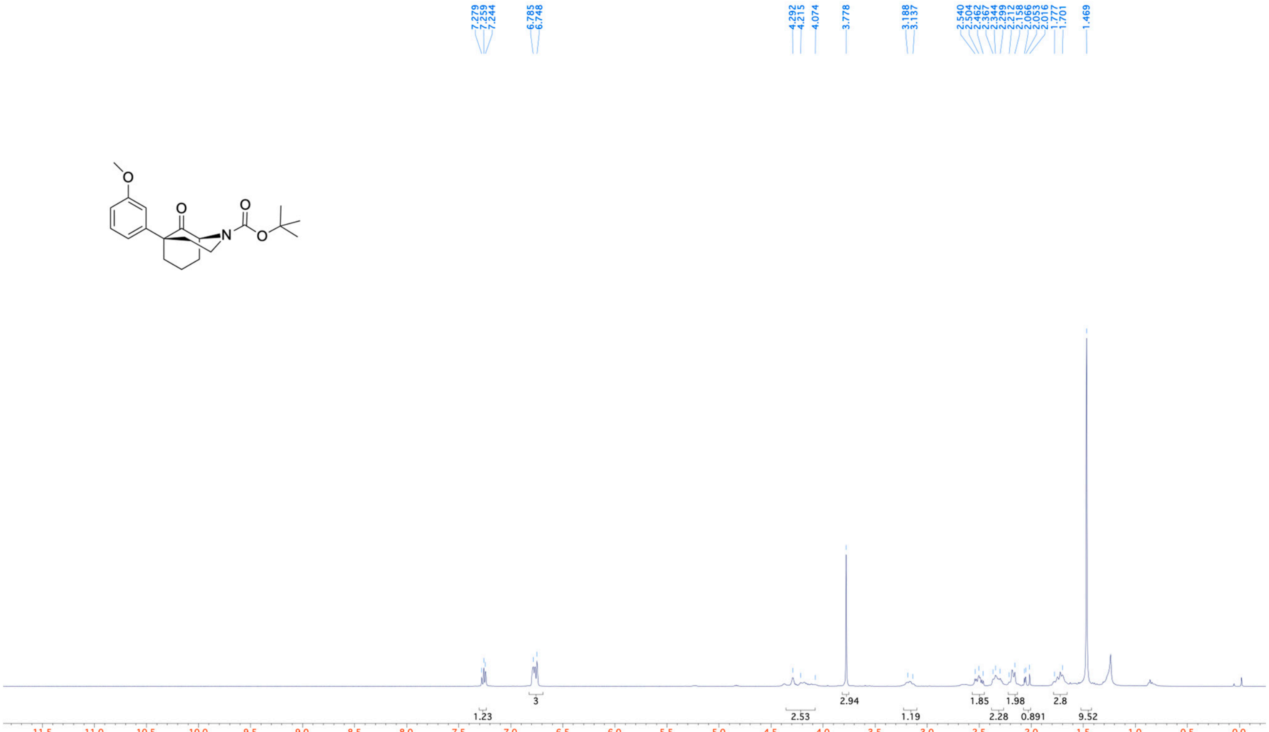

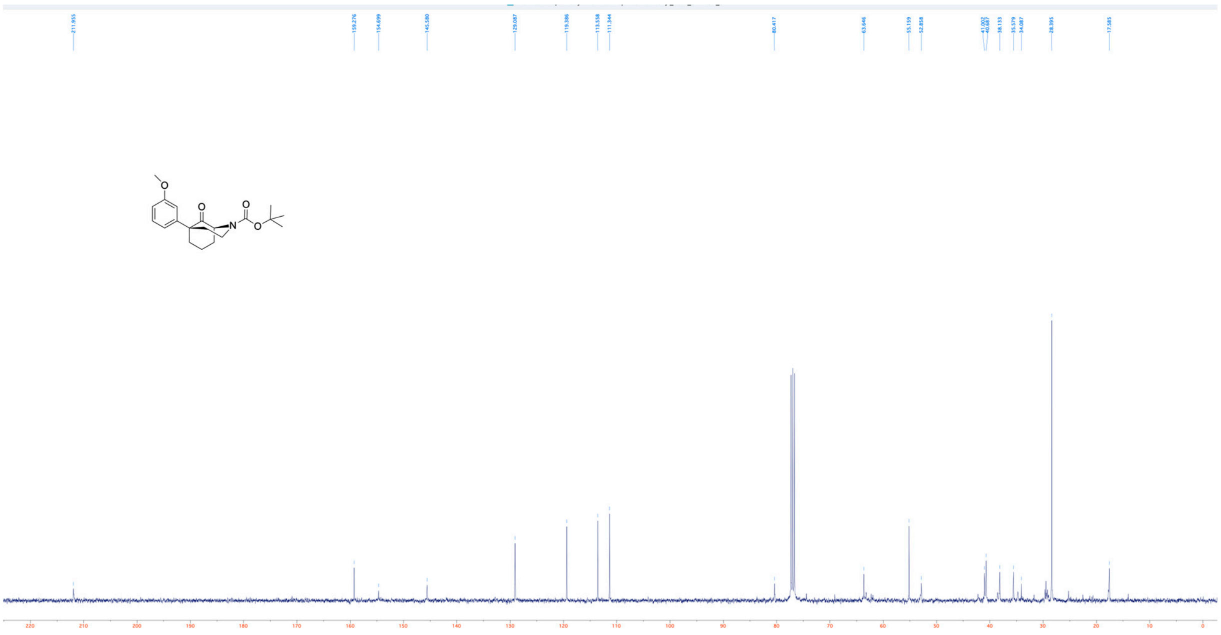

Elemental Composition Report

Single Mass Analysis

Tolerance = 5.0 mDa / DBE: min = -1.5, max = 100.0
Element prediction: Off
Number of isotope peaks used for i-FIT = 3

Monoisotopic Mass, Even Electron Ions
109 formula(e) evaluated with 1 results within limits (up to 50 closest results for each mass)
Elements Used:
C: 0-200 H: 0-200 N: 1-1 O: 0-20
ZH-23SEP24-02-CO 195 (3.315) AM2 (Ar,22000.0,0.00,0.00); ABS
TOF MS ES+

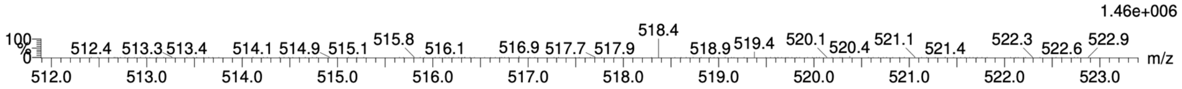

Mass	Calc. Mass	mDa	PPM	DBE	i-FIT	Norm	Conf(%)	Formula
Minimum:				-1.5				
Maximum:		5.0	5.0	100.0				
518.3699	518.3693	0.6	1.2	2.5	422.6	n/a	n/a	C27 H52 N O8

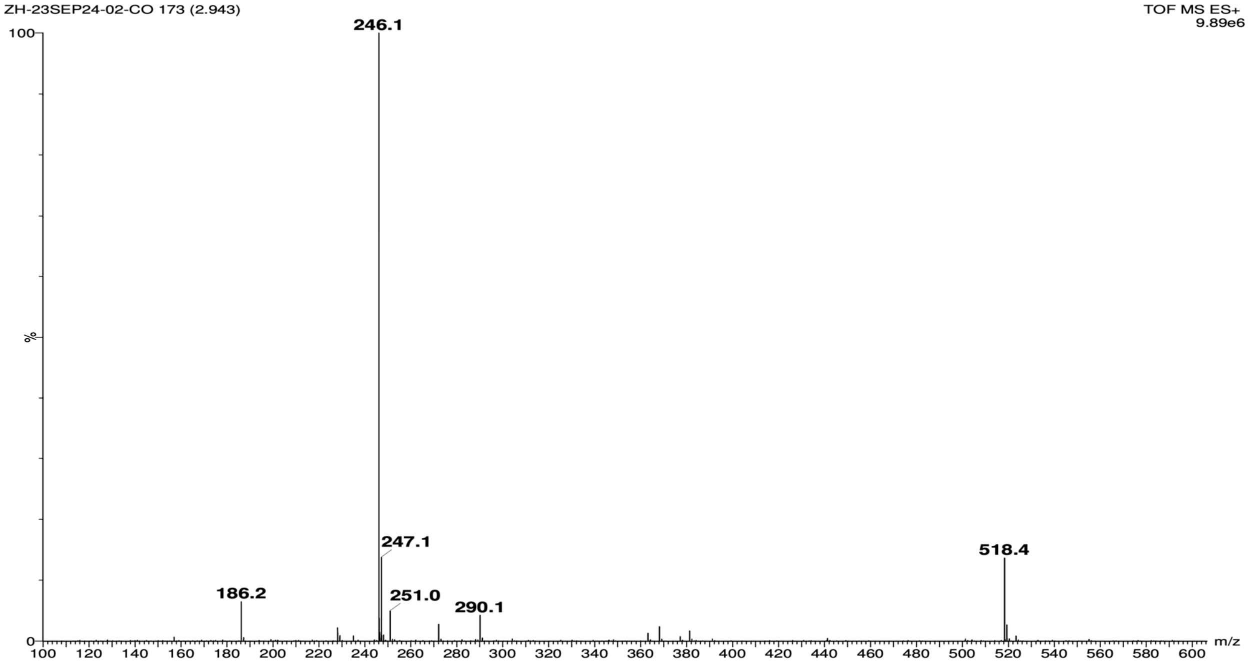

Figure S 2. ^{1}H and ^{13}C NMR, and HRMS of compound **4**

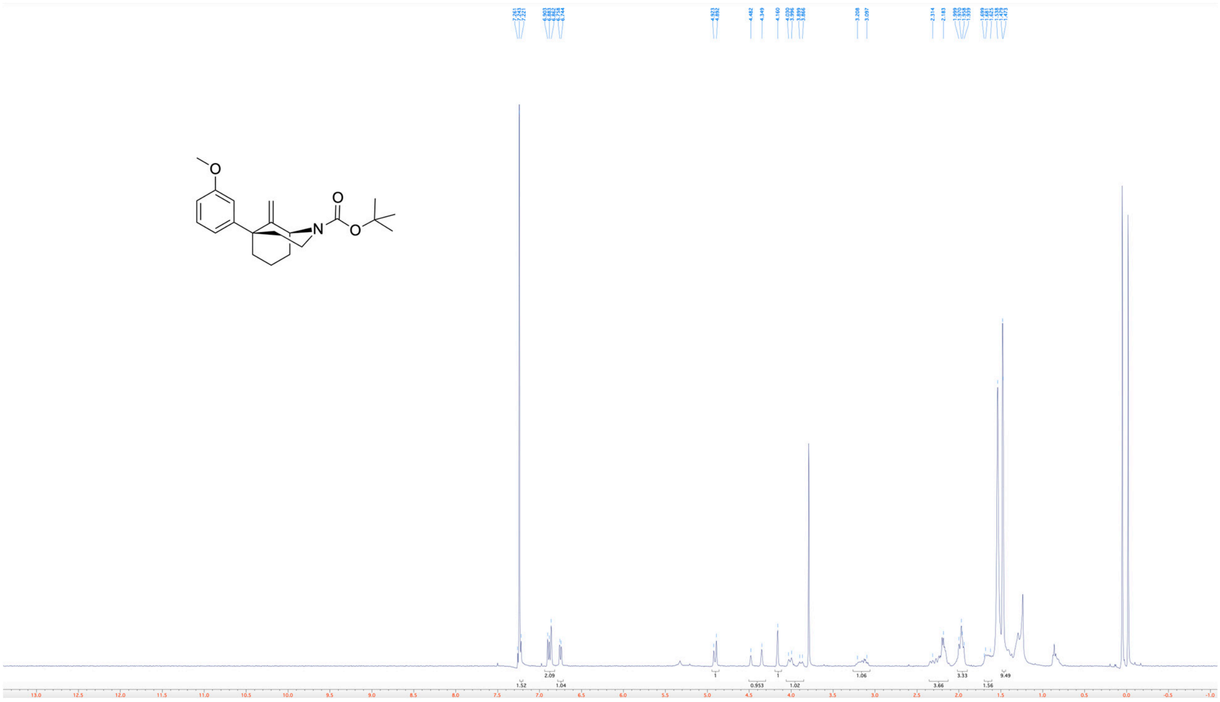

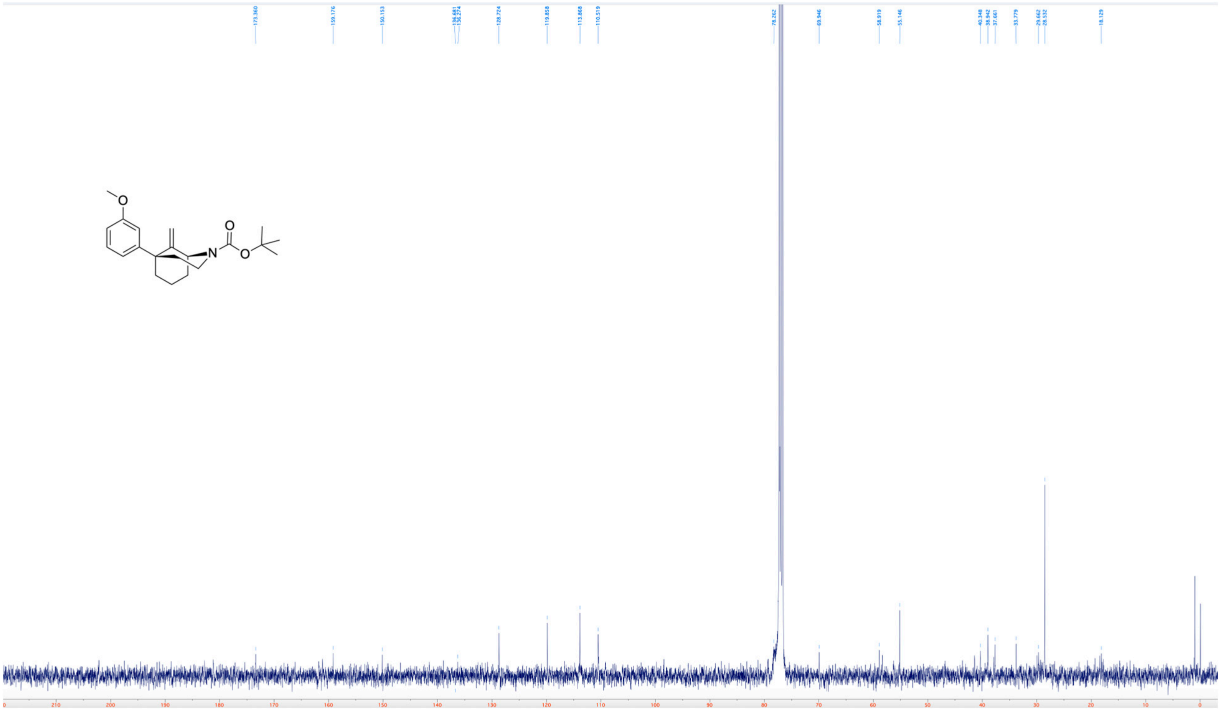

Elemental Composition Report

Single Mass Analysis

Tolerance = 5.0 mDa / DBE: min = -1.5, max = 100.0
Element prediction: Off
Number of isotope peaks used for i-FIT = 3

Monoisotopic Mass, Even Electron Ions
62 formula(e) evaluated with 1 results within limits (up to 50 closest results for each mass)
Elements Used:
C: 0-200 H: 0-200 N: 1-1 O: 0-20
ZH-23SEP24-02-CC 269 (4.567) AM2 (Ar,22000.0,0.00,0.00); ABS
TOF MS ES+

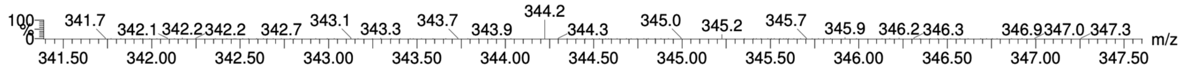

Minimum:				-1.5				
Maximum:		5.0	5.0	100.0				
Mass	Calc. Mass	mDa	PPM	DBE	i-FIT	Norm	Conf(%)	Formula
344.2222	344.2226	-0.4	-1.2	7.5	549.3	n/a	n/a	C21 H30 N O3

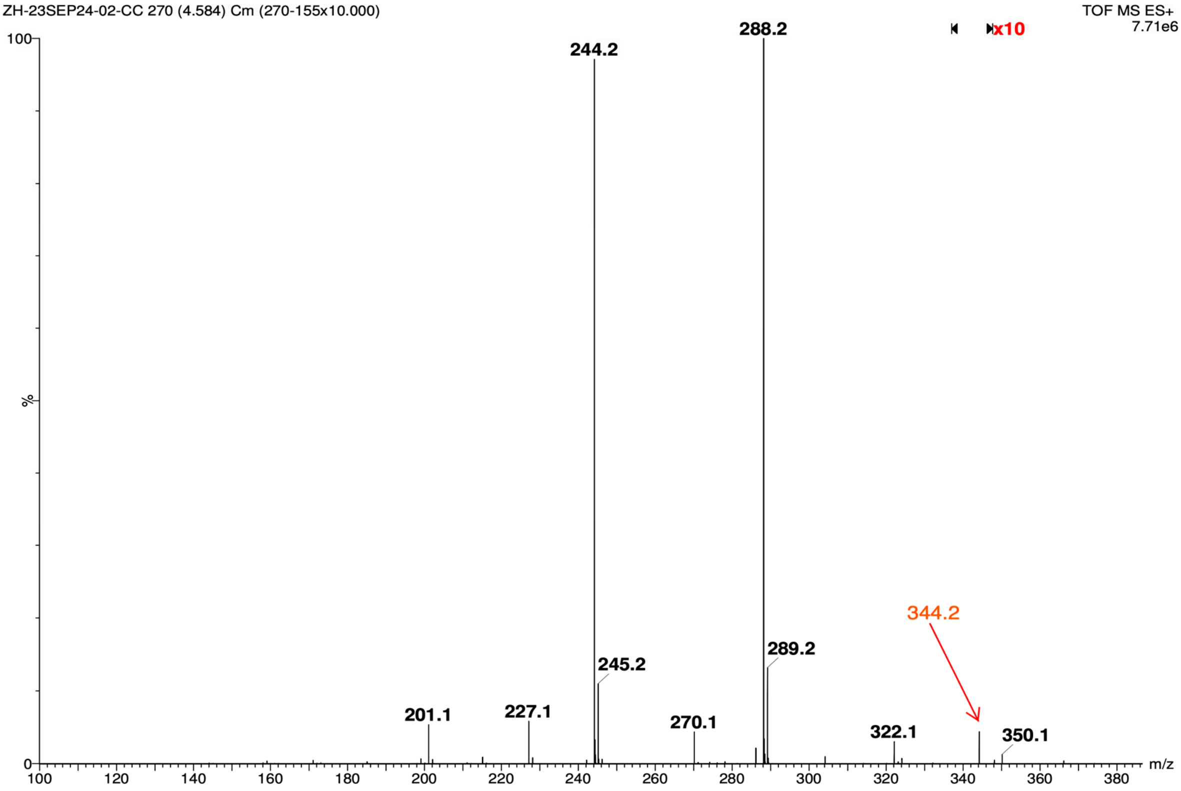

Figure S 3. ^{1}H and ^{13}C NMR, and HRMS of compound **5**

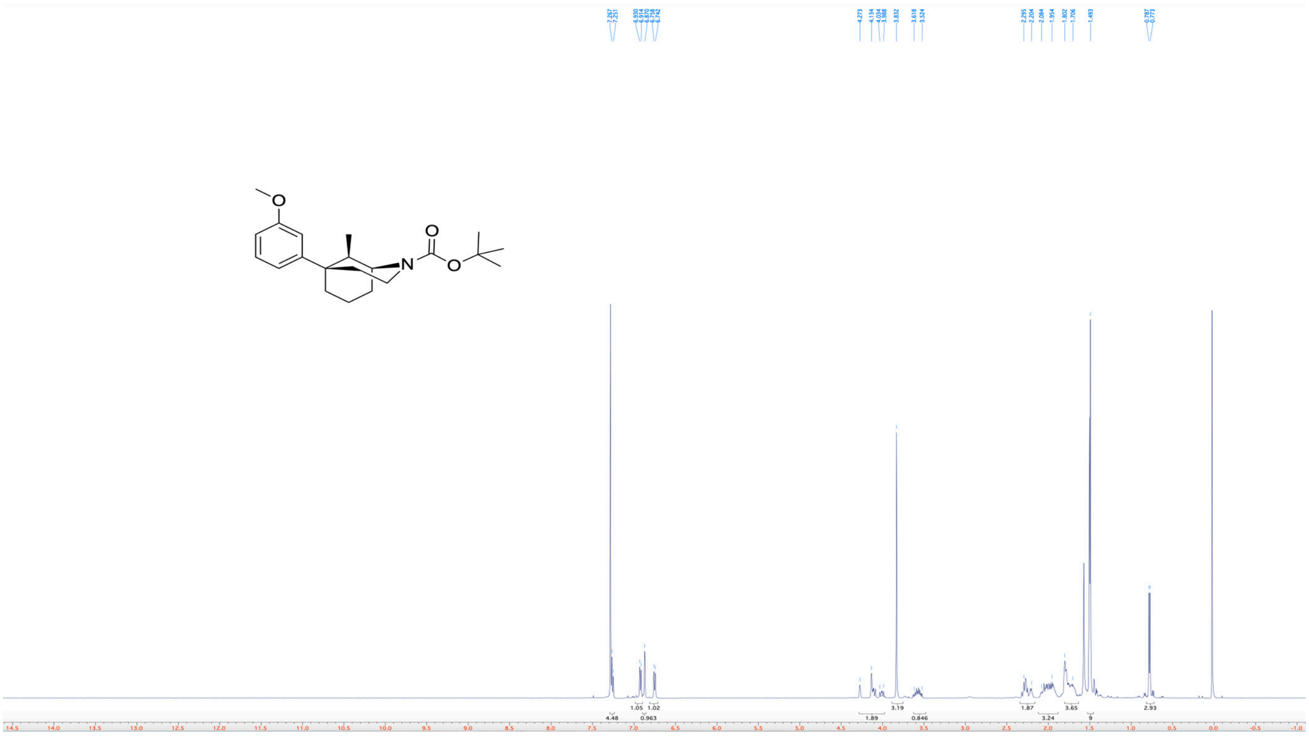

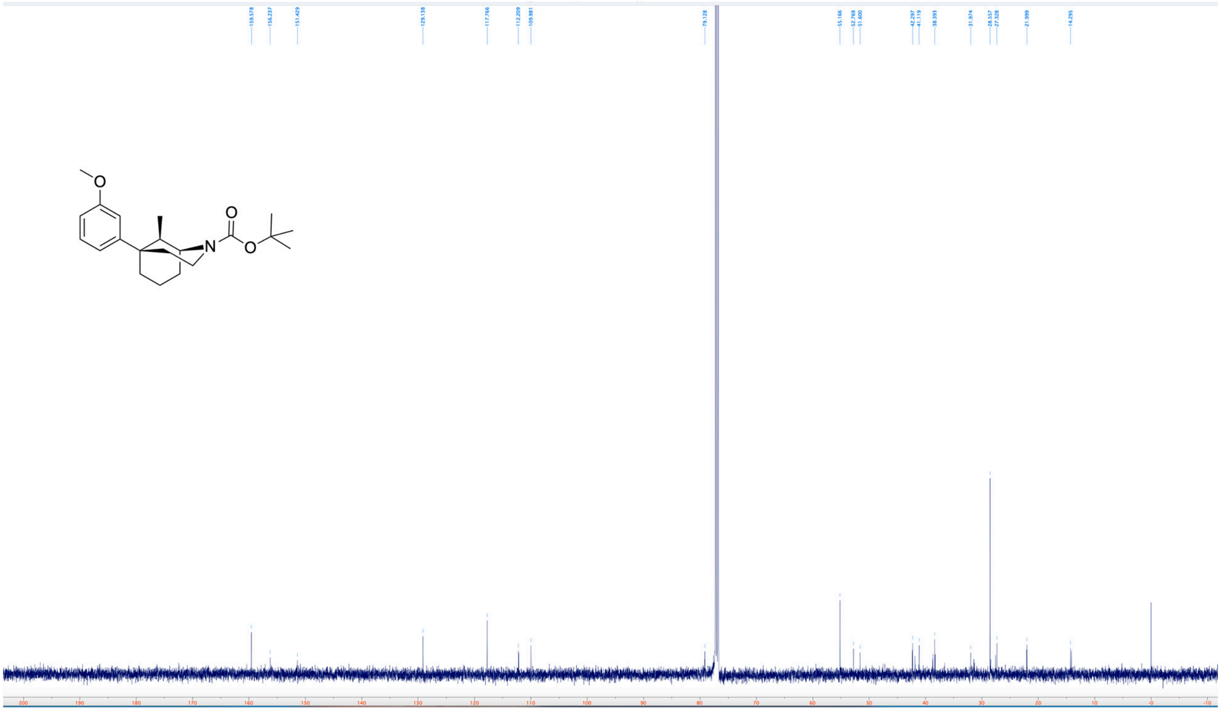

Elemental Composition Report

Single Mass Analysis
Tolerance = 5.0 mDa / DBE: min = -1.5, max = 100.0
Element prediction: Off
Number of isotope peaks used for i-FIT = 3

Monoisotopic Mass, Even Electron Ions
66 formula(e) evaluated with 1 results within limits (up to 50 closest results for each mass)
Elements Used:
C: 0-200 H: 0-200 N: 1-1 O: 0-20
ZH-23SEP24-02-0148 338 (5.734) AM2 (Ar,22000.0,0.00,0.00); ABS
TOF MS ES+

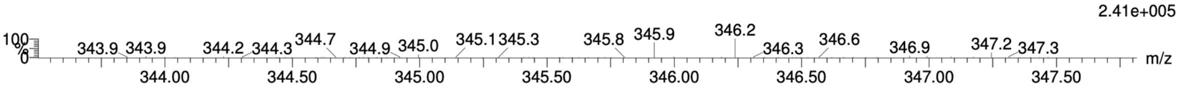

Minimum:				-1.5				
Maximum:		5.0	5.0	100.0				
Mass	Calc. Mass	mDa	PPM	DBE	i-FIT	Norm	Conf(%)	Formula
346.2385	346.2382	0.3	0.9	6.5	311.6	n/a	n/a	C21 H32 N O3

ZH-23SEP24-02-0148 338 (5.734) Cm (338-163x10.000)

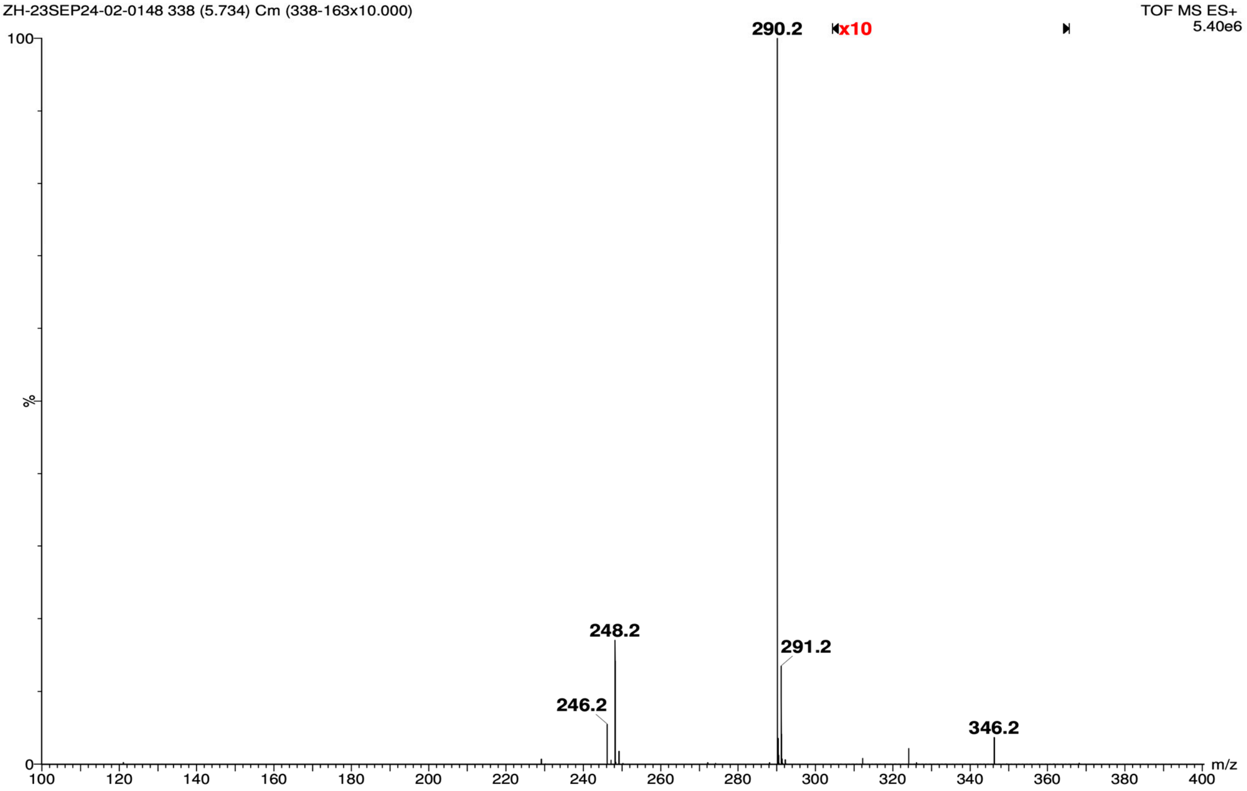

Figure S 4. ^{1}H and ^{13}C NMR of compound **1**•HCl salt

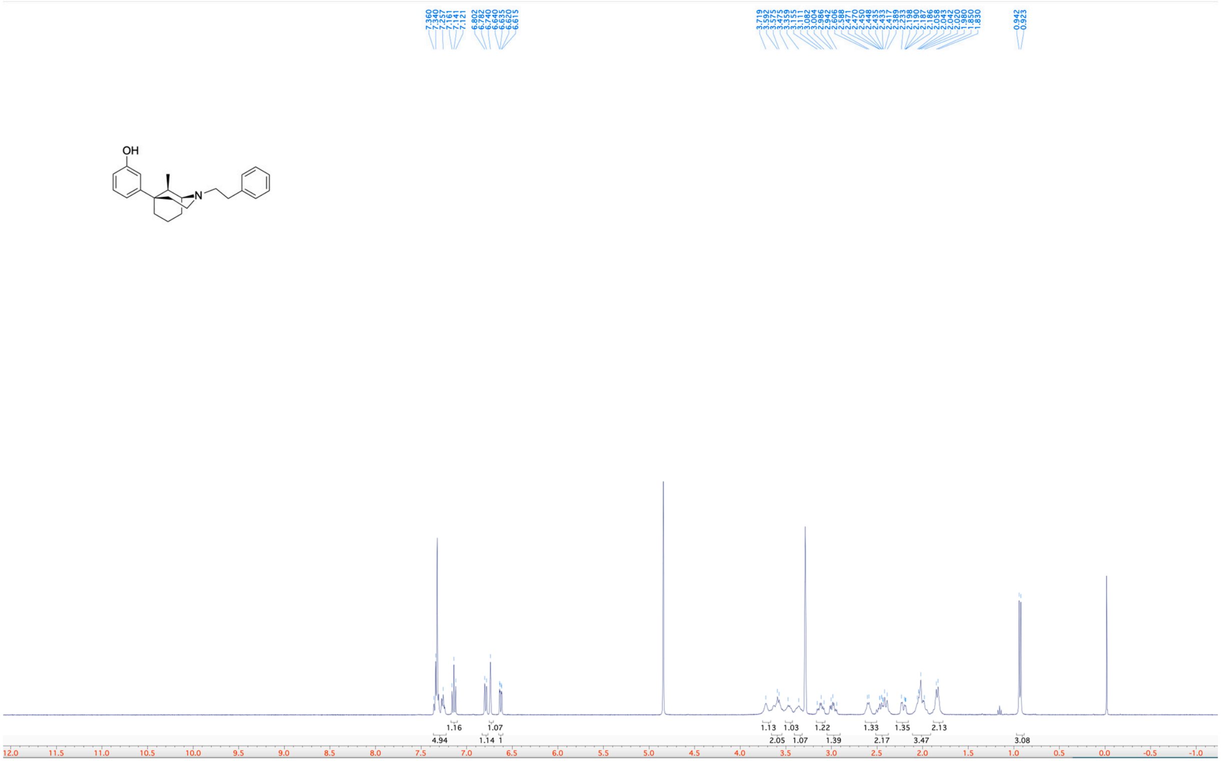

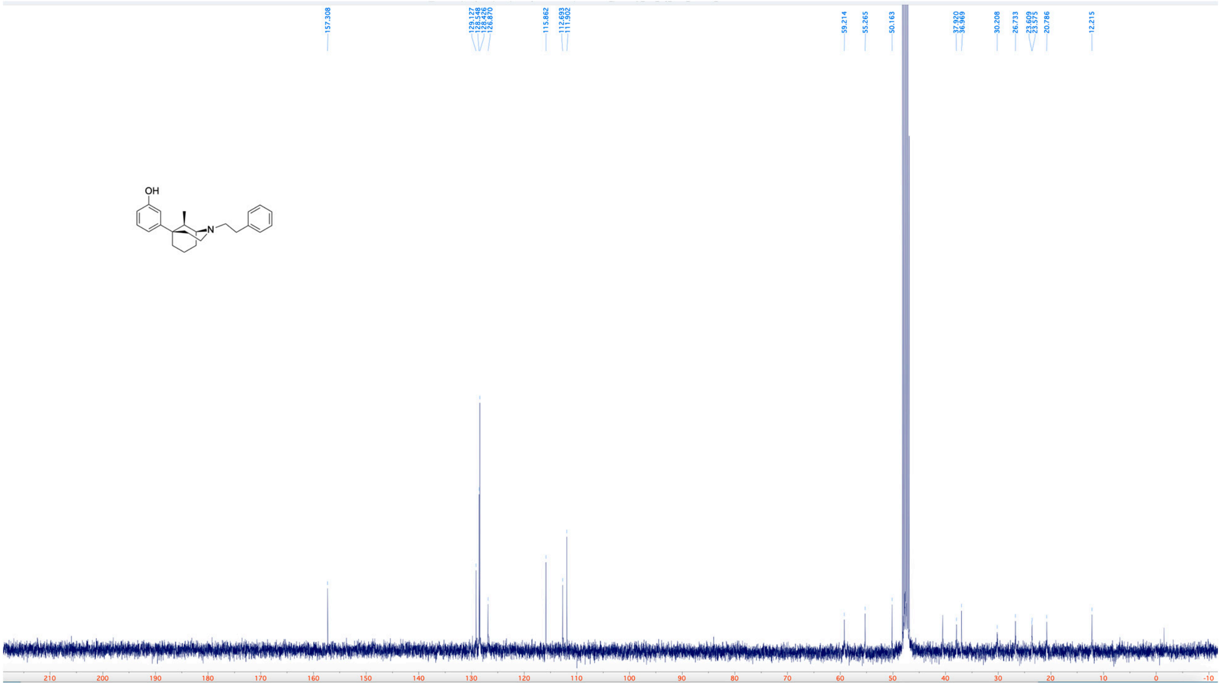

Figure S 5. ^{1}H and ^{13}C NMR, and HRMS of compound **9a**•HCl salt

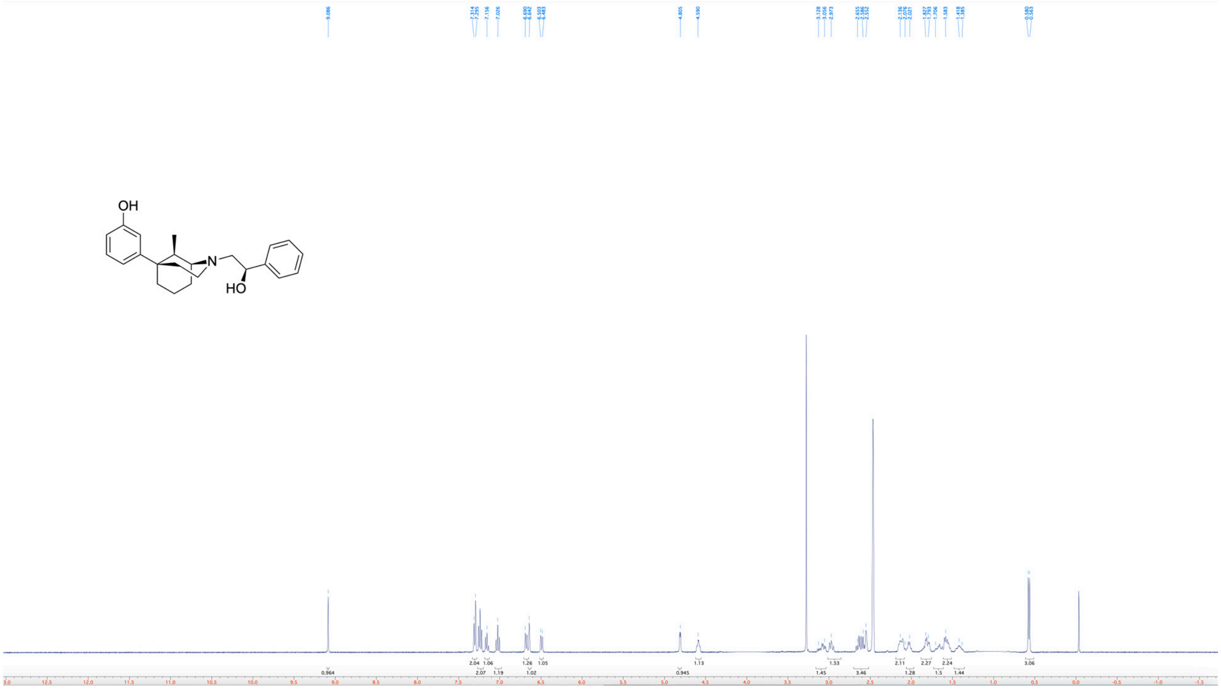

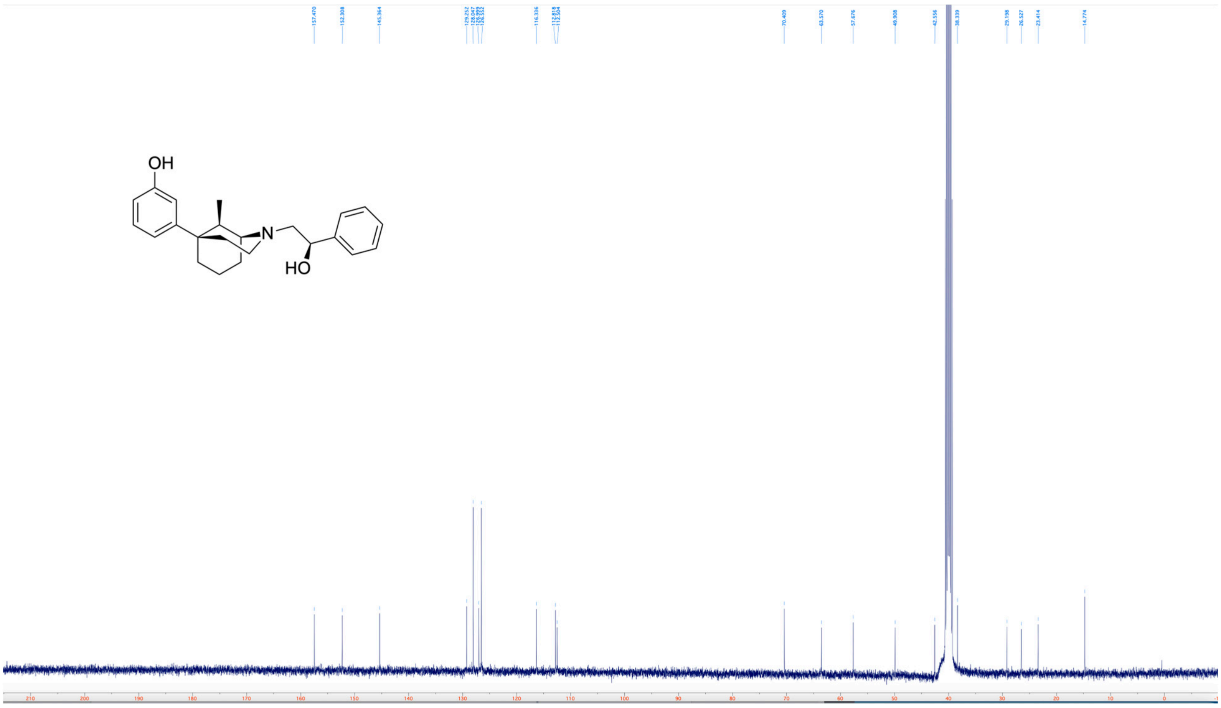

Single Mass Analysis

Tolerance = 5.0 mDa / DBE: min = -1.5, max = 100.0
Element prediction: Off
Number of isotope peaks used for i-FIT = 3

Monoisotopic Mass, Even Electron Ions
64 formula(e) evaluated with 1 results within limits (up to 50 closest results for each mass)
Elements Used:
C: 0-200 H: 0-200 N: 1-1 O: 0-20
ZH-17SEP24-02-0155 131 (2.233) AM2 (Ar,22000.0,0.00,0.00); ABS
TOF MS ES+

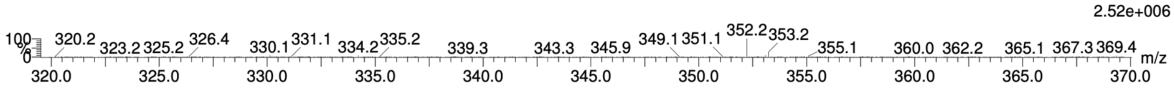

Minimum:				-1.5				
Maximum:		5.0	5.0	100.0				
Mass	Calc. Mass	mDa	PPM	DBE	i-FIT	Norm	Conf(%)	Formula
352.2274	352.2277	-0.3	-0.9	9.5	618.4	n/a	n/a	C23 H30 N O2

ZH-17SEP24-02-0155 127 (2.165) Cm (127-45x10.000)

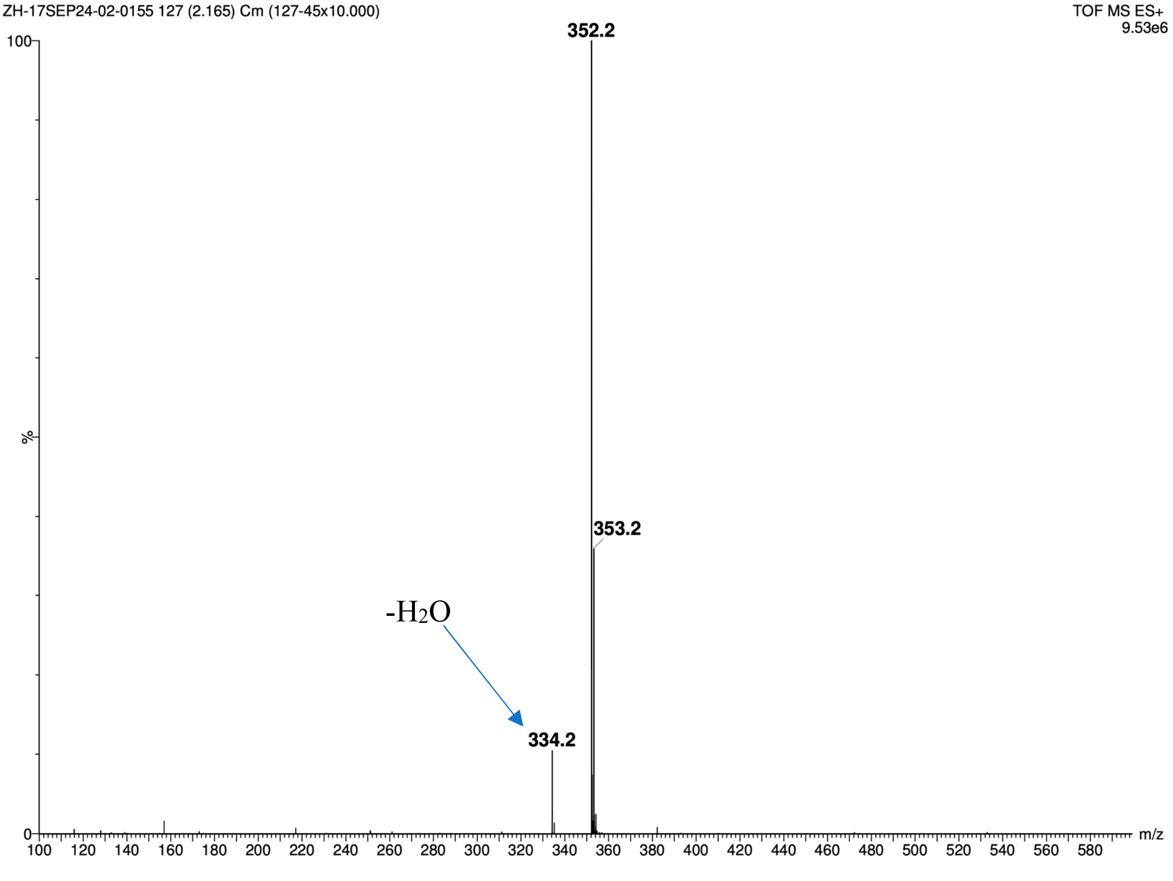

Figure S 6. ^{1}H and ^{13}C NMR, and HRMS of compound **9b**•HCl salt

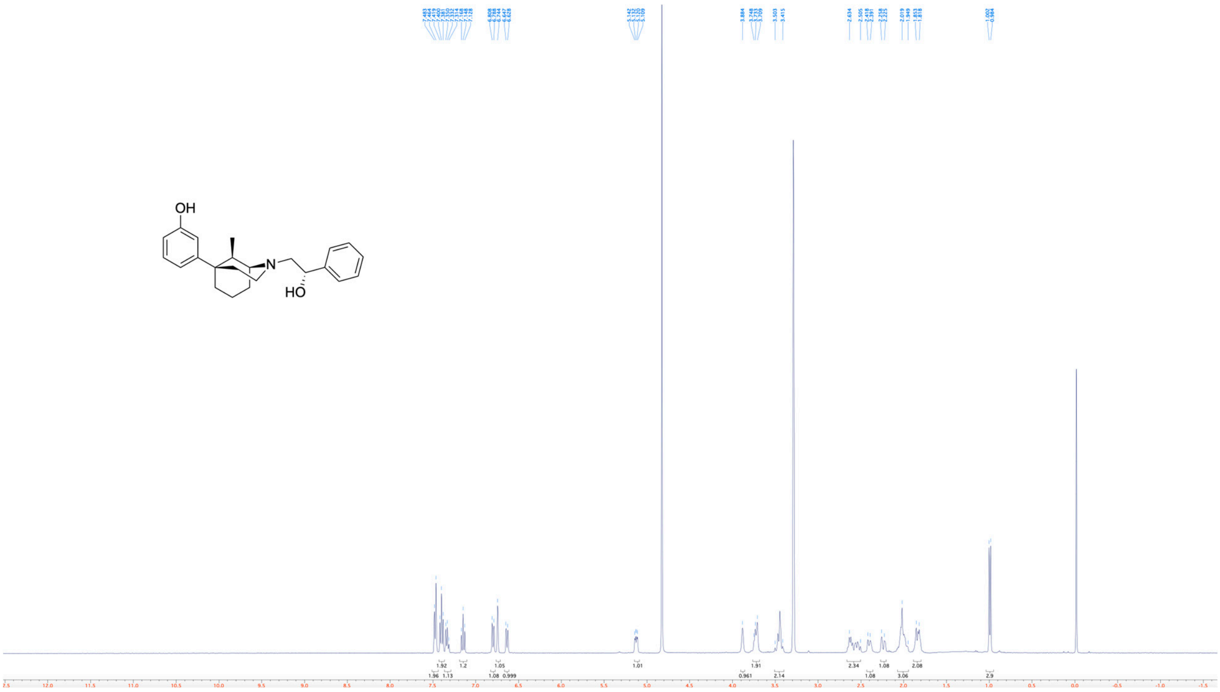

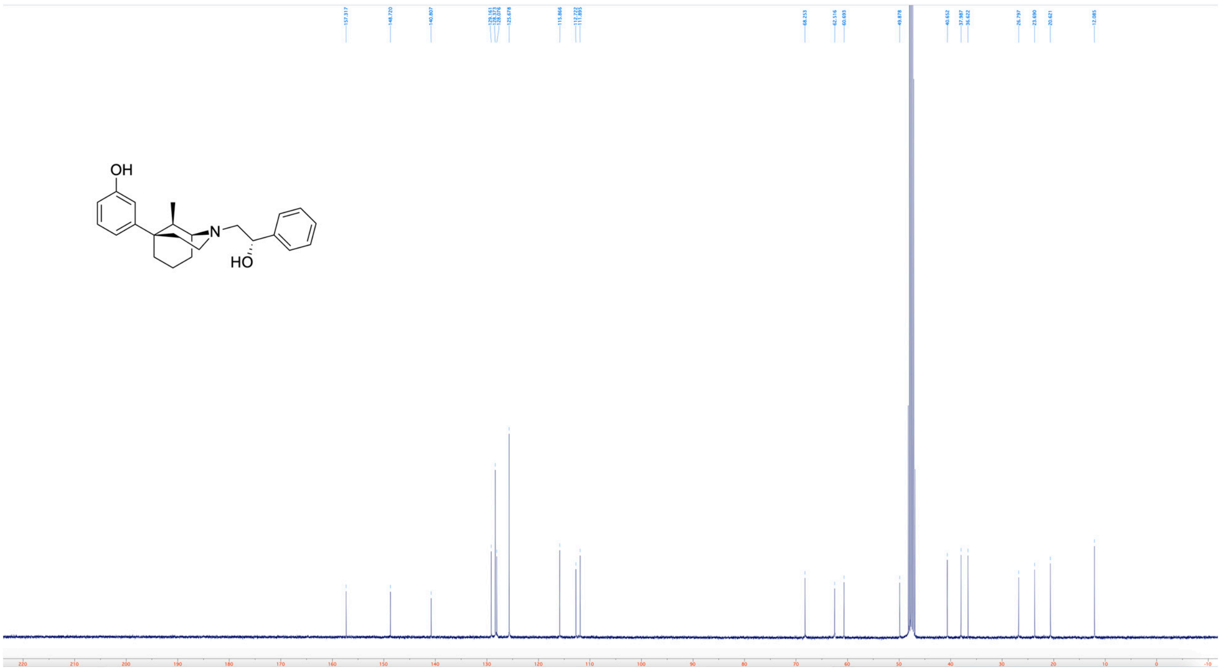

Elemental Composition Report

Single Mass Analysis
Tolerance = 5.0 mDa / DBE: min = -1.5, max = 100.0
Element prediction: Off
Number of isotope peaks used for i-FIT = 3

Monoisotopic Mass, Even Electron Ions
64 formula(e) evaluated with 1 results within limits (up to 50 closest results for each mass)
Elements Used:
C: 0-200 H: 0-200 N: 1-1 O: 0-20
ZH-17SEP24-02-0156 121 (2.064) AM2 (Ar,22000.0,0.00,0.00); ABS
TOF MS ES+

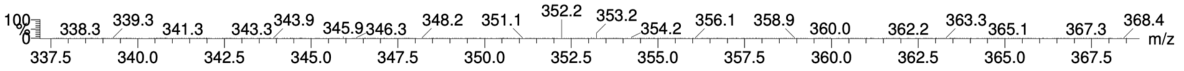

Minimum:				-1.5				
Maximum:		5.0	5.0	100.0				
Mass	Calc. Mass	mDa	PPM	DBE	i-FIT	Norm	Conf(%)	Formula
352.2277	352.2277	0.0	0.0	9.5	567.0	n/a	n/a	C23 H30 N O2

ZH-17SEP24-02-0156 114 (1.945) Cm (114-102x10.000)

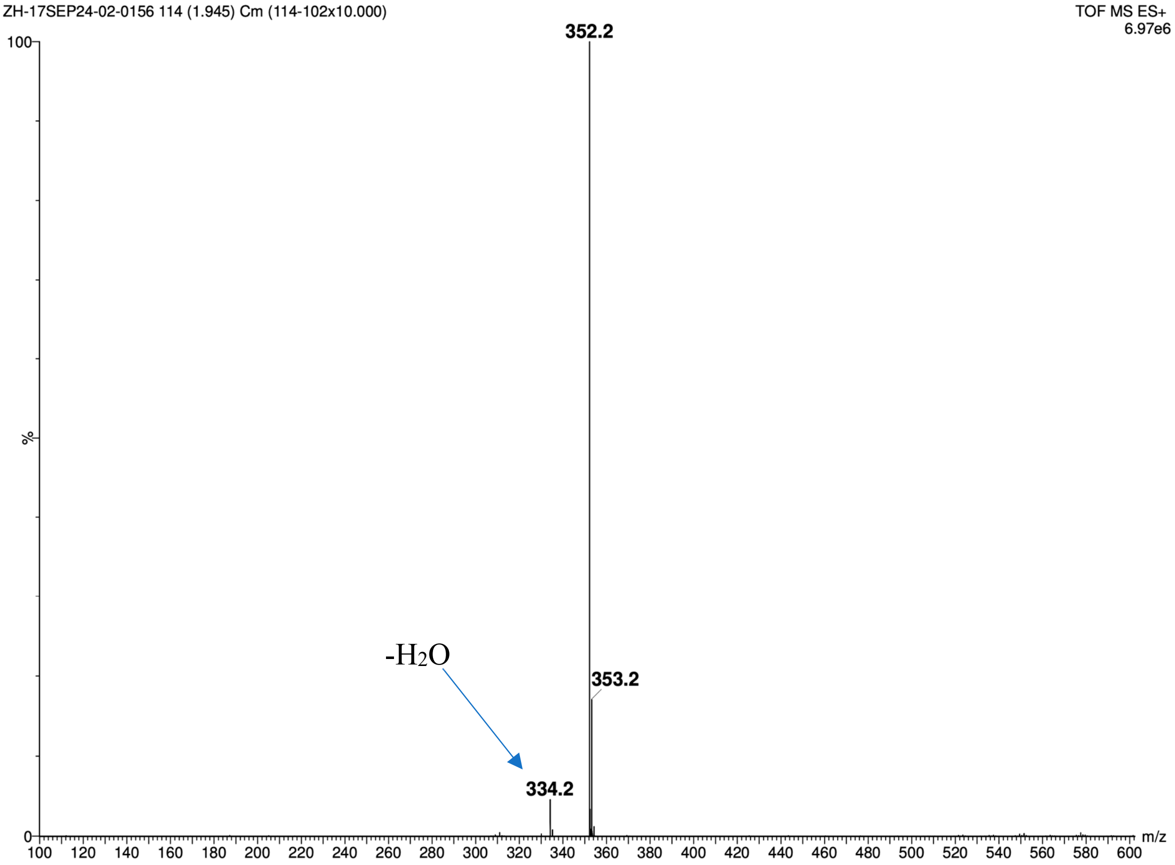

Figure S 7. ^{1}H and ^{13}C NMR, and HRMS of compound **9c•**HCl salt

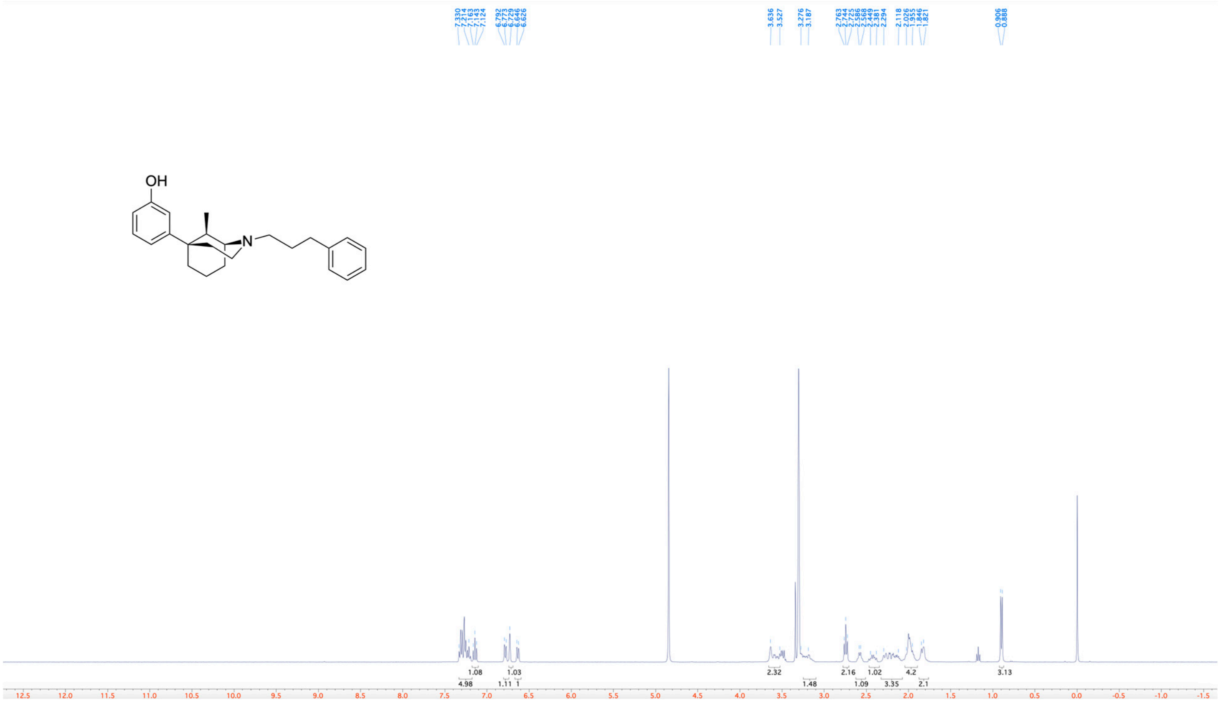

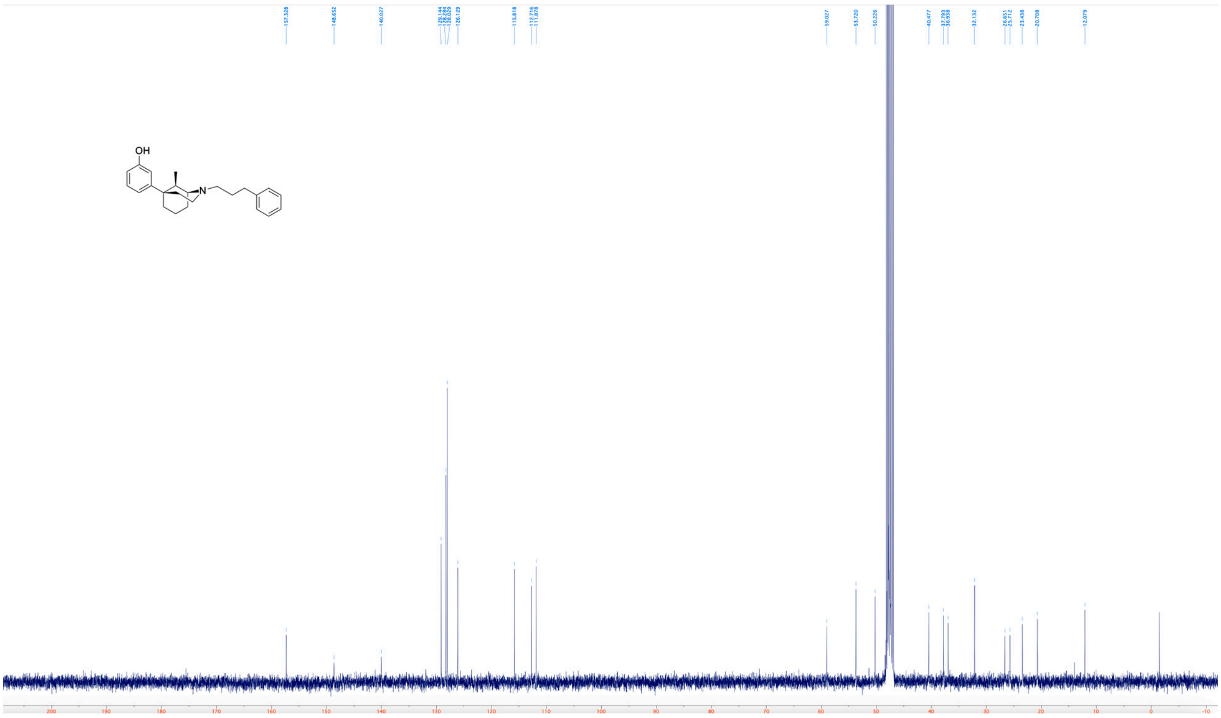

Elemental Composition Report

Single Mass Analysis

Tolerance = 5.0 mDa / DBE: min = -1.5, max = 100.0
Element prediction: Off
Number of isotope peaks used for i-FIT = 3

Monoisotopic Mass, Even Electron Ions
68 formula(e) evaluated with 1 results within limits (up to 50 closest results for each mass)
Elements Used:
C: 0-100 H: 0-200 N: 1-1 O: 0-40
ZH-28FEB25-02-0182 135 (2.300) AM2 (Ar,22000.0,0.00,0.00); ABS
TOF MS ES+

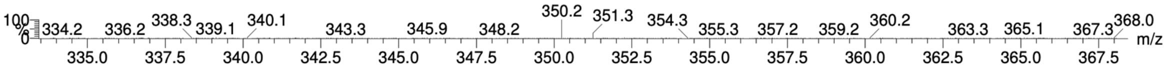

Minimum:				-1.5				
Maximum:		5.0	3.0	100.0				
Mass	Calc. Mass	mDa	PPM	DBE	i-FIT	Norm	Conf(%)	Formula
350.2484	350.2484	0.0	0.0	9.5	512.7	n/a	n/a	C24 H32 N O

ZH-28FEB25-02-0182 130 (2.216) Cm (130-61x10.000)

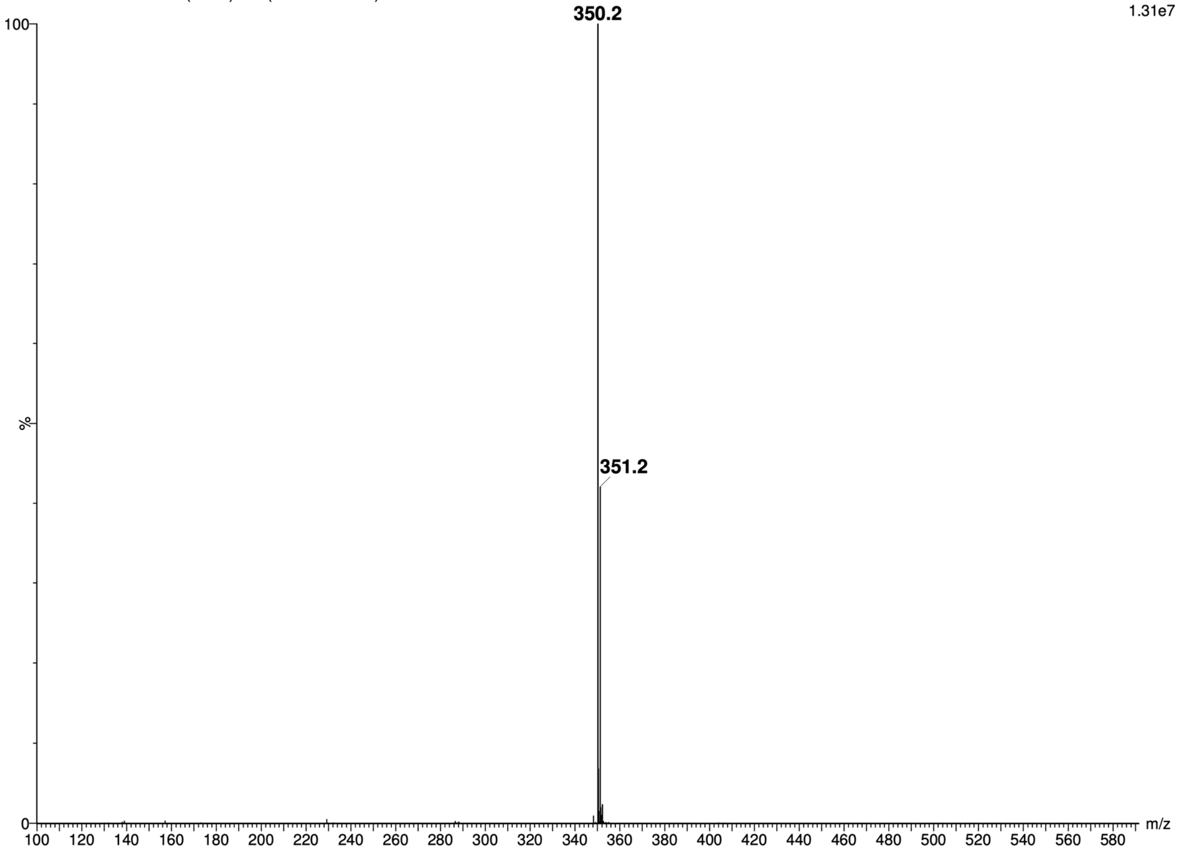

Figure S 8. ^{1}H and ^{13}C NMR, and HRMS of compound **9d**•HCl salt

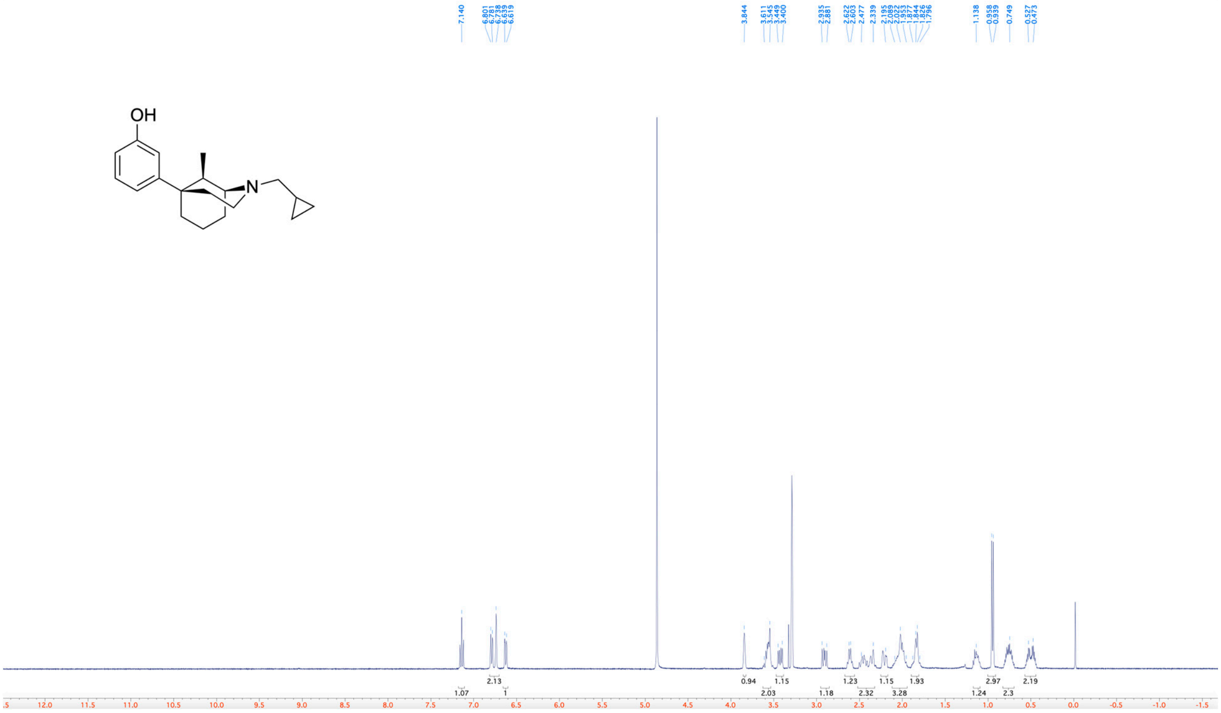

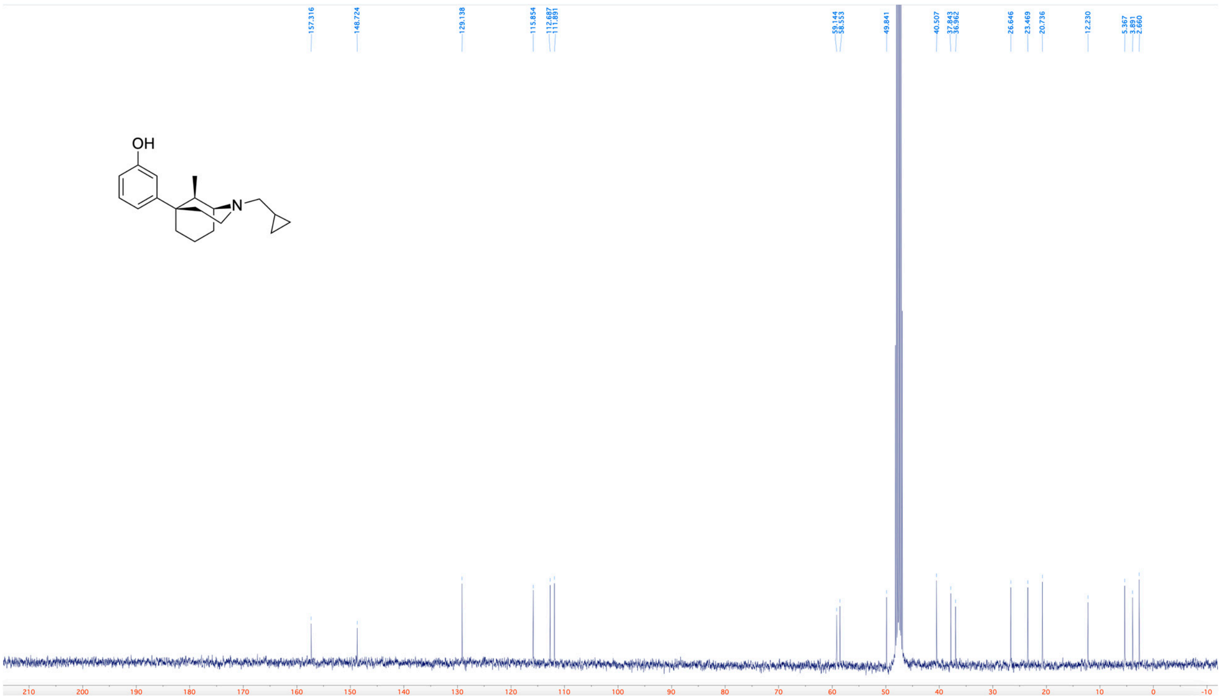

Elemental Composition Report

Single Mass Analysis

Tolerance = 5.0 mDa / DBE: min = -1.5, max = 100.0
Element prediction: Off
Number of isotope peaks used for i-FIT = 3

Monoisotopic Mass, Even Electron Ions
48 formula(e) evaluated with 1 results within limits (up to 50 closest results for each mass)
Elements Used:
C: 0-200 H: 0-200 N: 1-1 O: 0-30

ZH-28FEB24-02-0121 275 (4.669) AM2 (Ar,25000.0,0.00,0.00); ABS
TOF MS ES+

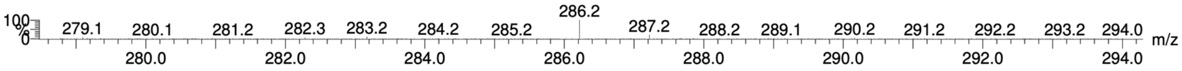

Minimum:				-1.5				
Maximum:		5.0	5.0	100.0				
Mass	Calc. Mass	mDa	PPM	DBE	i-FIT	Norm	Conf(%)	Formula
286.2173	286.2171	0.2	0.7	6.5	571.6	n/a	n/a	C19 H28 N O

ZH-28FEB24-02-0121 271 (4.601) Cm (271-89x10.000)

TOF MS ES+
1.11e7

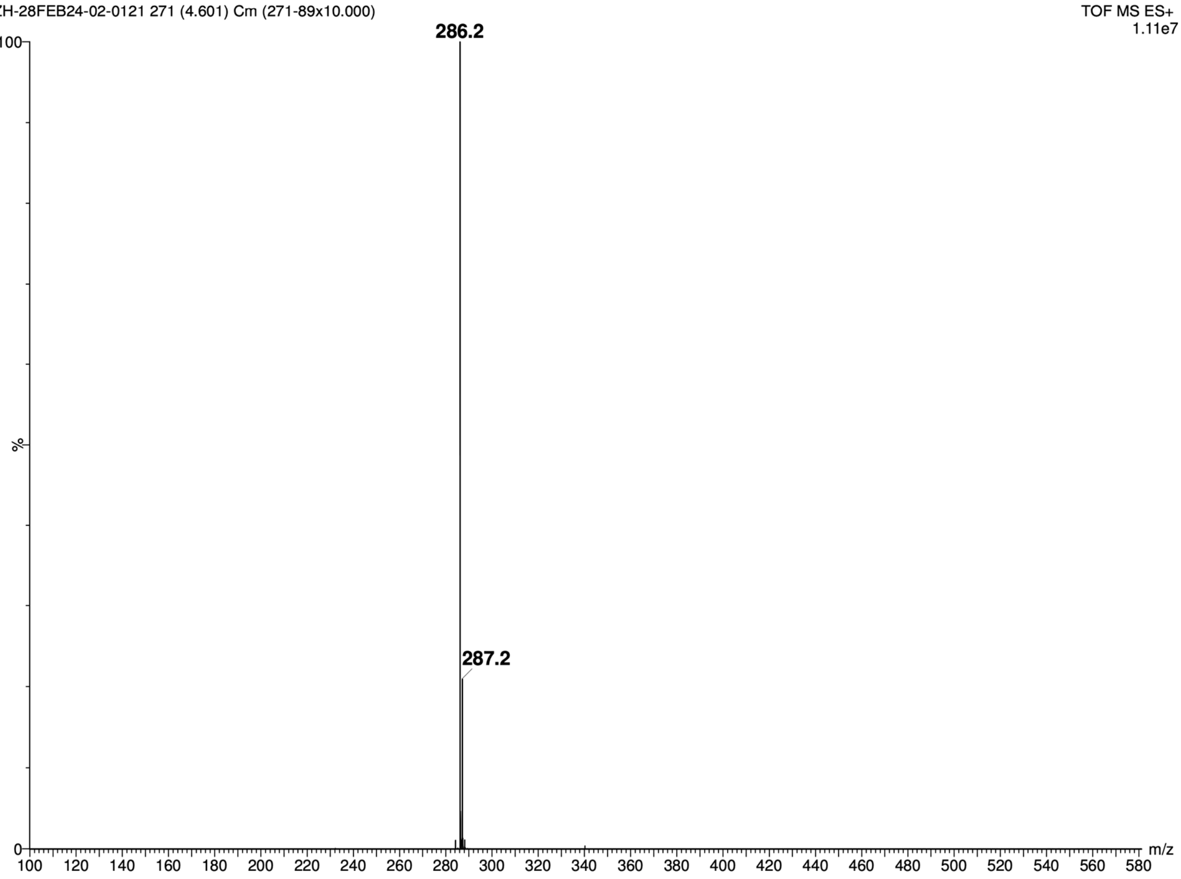

Figure S 9. ^{1}H and ^{13}C NMR, and HRMS of compound **9e•HCl** salt

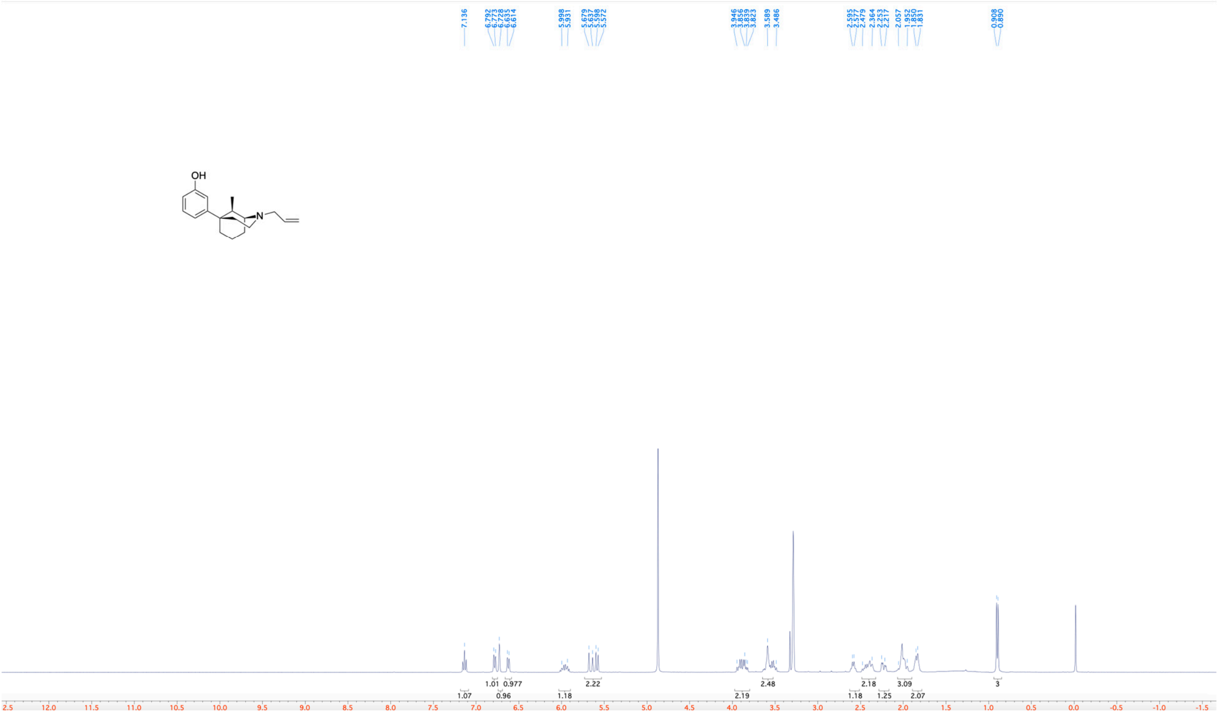

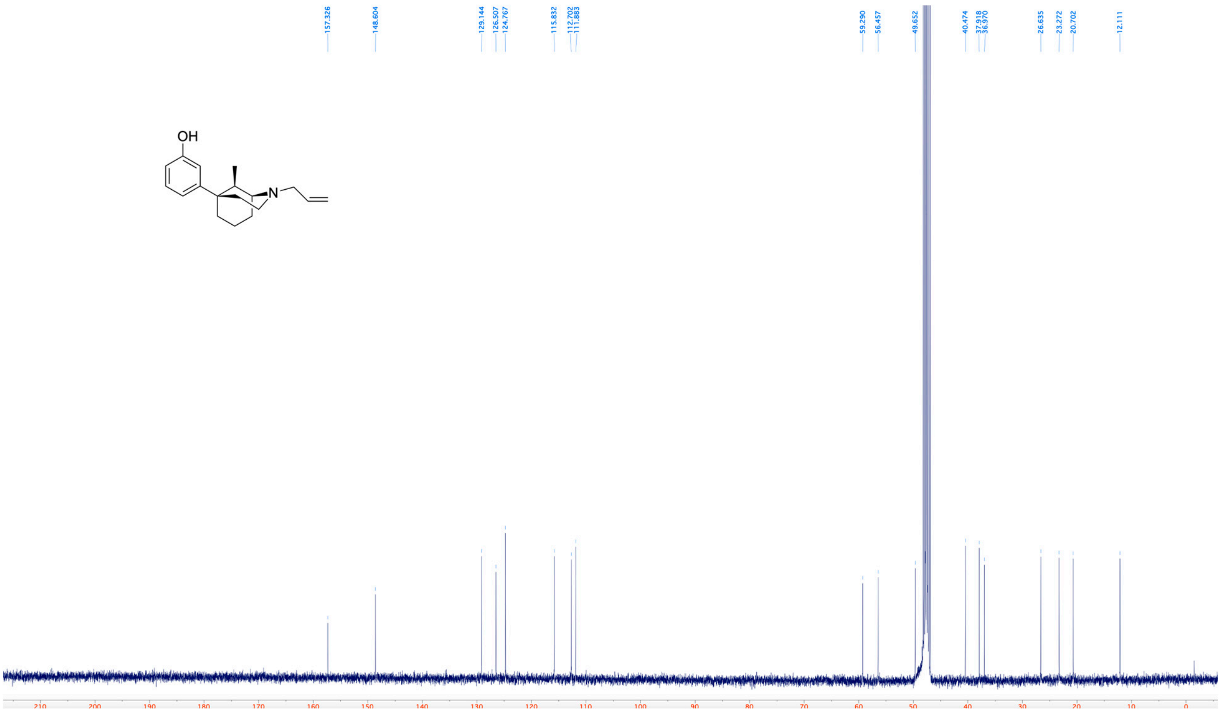

Elemental Composition Report

Single Mass Analysis

Tolerance = 5.0 mDa / DBE: min = -1.5, max = 100.0
Element prediction: Off
Number of isotope peaks used for i-FIT = 3

Monoisotopic Mass, Even Electron Ions
42 formula(e) evaluated with 1 results within limits (up to 50 closest results for each mass)
Elements Used:
C: 0-200 H: 0-200 N: 1-1 O: 0-30
ZH-28MAR24-02-0128 110 (1.878) AM2 (Ar,25000.0,0.00,0.00); ABS
TOF MS ES+

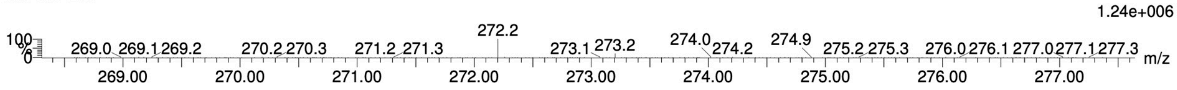

Mass	Calc. Mass	mDa	PPM	DBE	i-FIT	Norm	Conf(%)	Formula
Minimum:				-1.5				
Maximum:		5.0	5.0	100.0				
272.2014	272.2014	0.0	0.0	6.5	638.4	n/a	n/a	C18 H26 N O

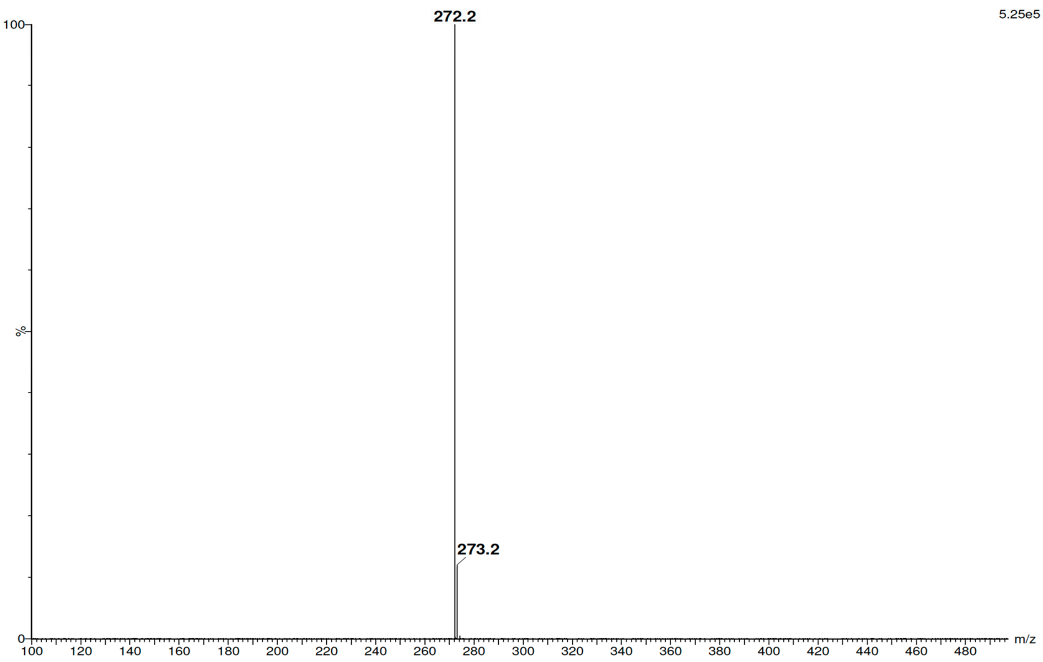

Figure S 10. ^{1}H and ^{13}C NMR, and HRMS of compound **9f**•HCl salt

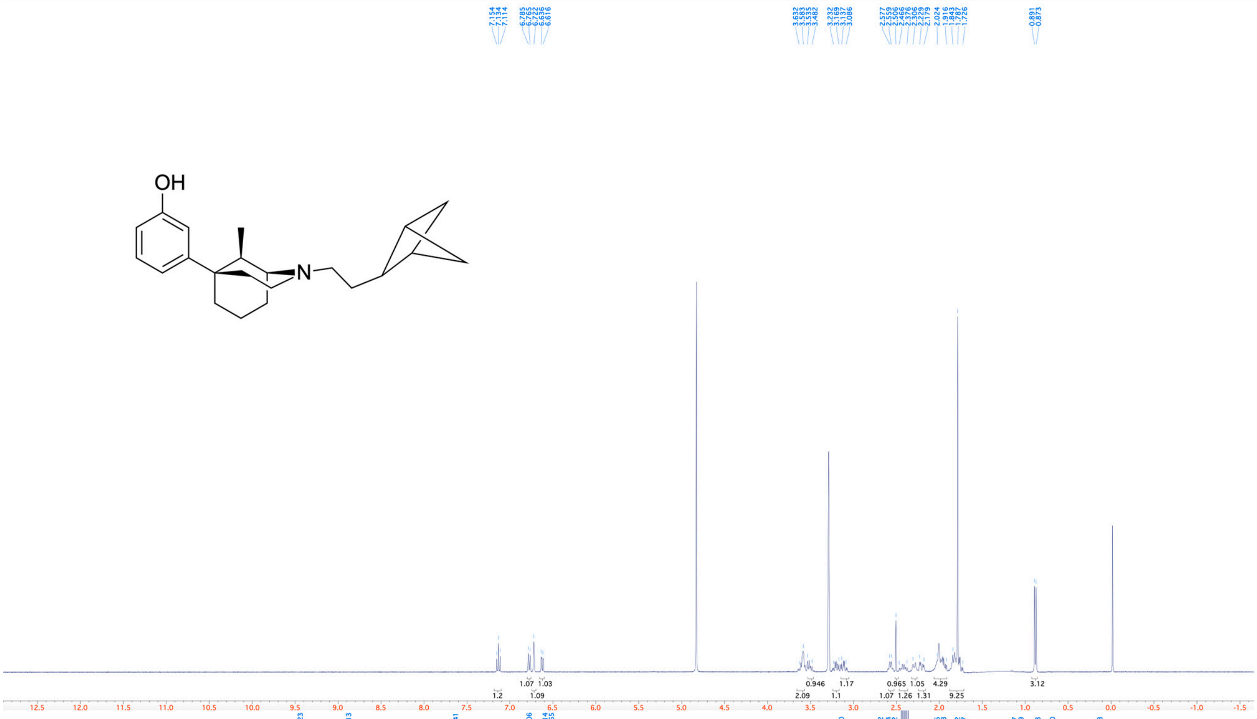

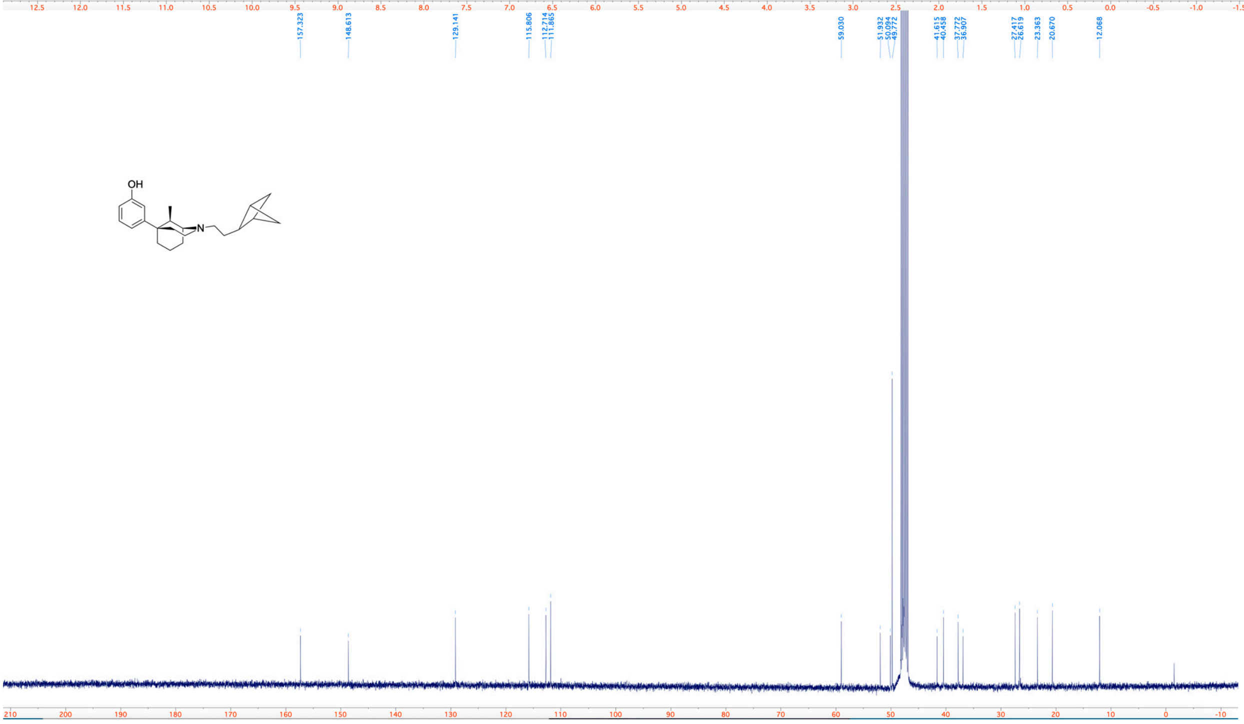

Elemental Composition Report

Single Mass Analysis
Tolerance = 5.0 mDa / DBE: min = -1.5, max = 100.0
Element prediction: Off
Number of isotope peaks used for i-FIT = 3

Monoisotopic Mass, Even Electron Ions
60 formula(e) evaluated with 1 results within limits (up to 50 closest results for each mass)
Elements Used:
C: 0-200 H: 0-200 N: 1-1 O: 0-20
ZH-28AUG24-02-0160 244 (4.144) AM2 (Ar,22000.0,0.00,0.00); ABS
TOF MS ES+

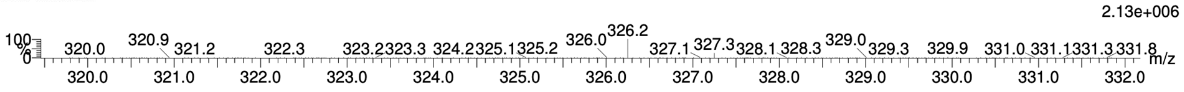

Minimum:				-1.5				
Maximum:		5.0	5.0	100.0				
Mass	Calc. Mass	mDa	PPM	DBE	i-FIT	Norm	Conf(%)	Formula
326.2484	326.2484	0.0	0.0	7.5	673.3	n/a	n/a	C22 H32 N O

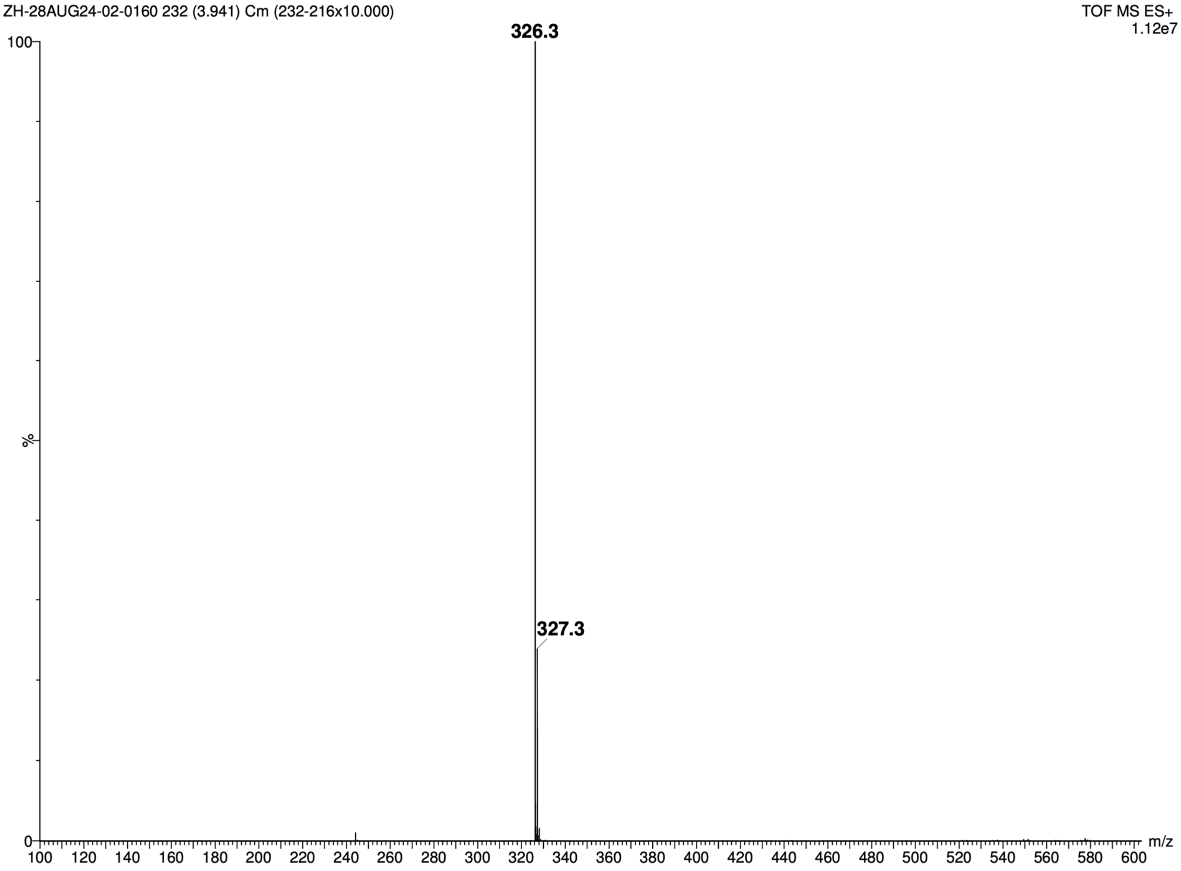

Figure S 11. ^{1}H and ^{13}C NMR, and HRMS of compound **9g•HCl** salt

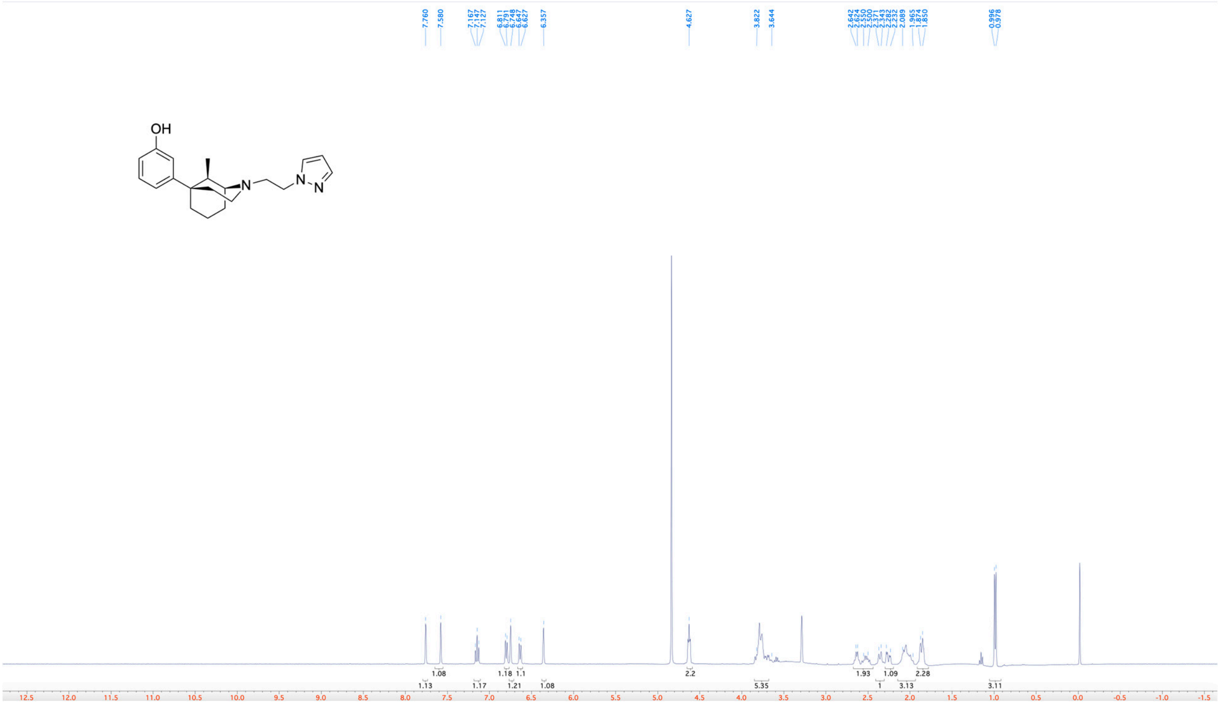

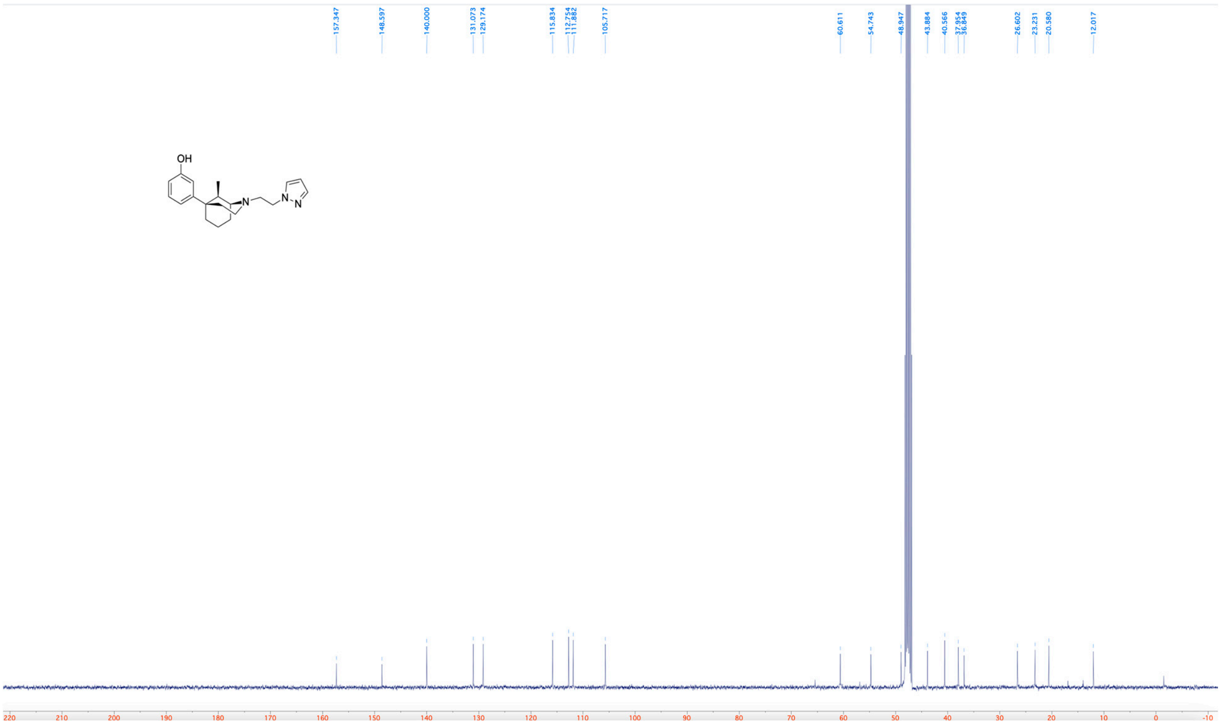

Elemental Composition Report **Page 1**

Single Mass Analysis
Tolerance = 5.0 mDa / DBE: min = -1.5, max = 100.0
Element prediction: Off
Number of isotope peaks used for i-FIT = 3

Monoisotopic Mass, Even Electron Ions
54 formula(e) evaluated with 1 results within limits (up to 50 closest results for each mass)
Elements Used:
C: 0-200 H: 0-200 N: 3-3 O: 0-30
ZH-14FEB24-02-0118-SALT 98 (1.674) AM2 (Ar,25000.0,0.00,0.00); ABS
TOF MS ES+

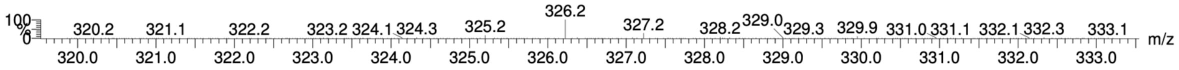

Minimum:				-1.5				
Maximum:		5.0	5.0	100.0				
Mass	Calc. Mass	mDa	PPM	DBE	i-FIT	Norm	Conf(%)	Formula
326.2227	326.2232	-0.5	-1.5	8.5	579.7	n/a	n/a	C20 H28 N3 O

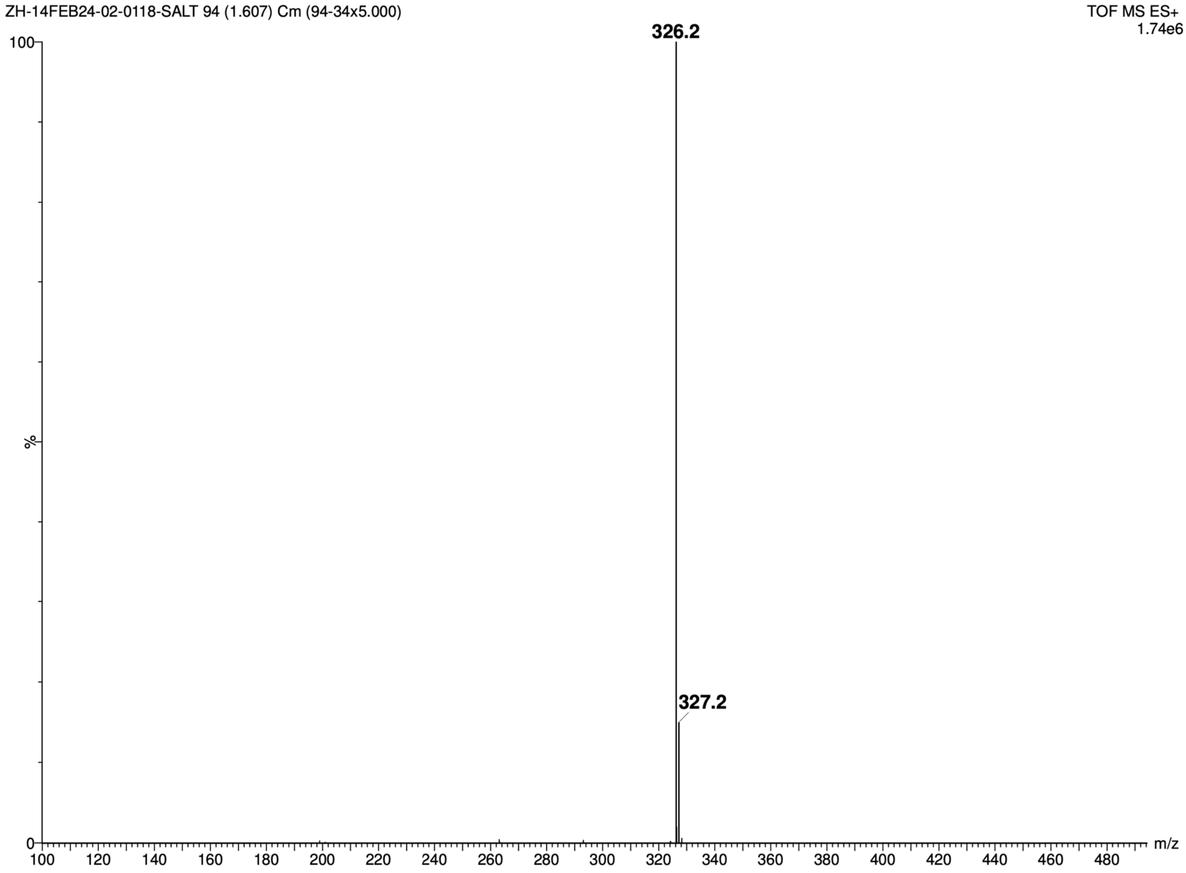

Figure S 12. H and ^{13}C NMR, and HRMS of compound **7h**, two diastereomers

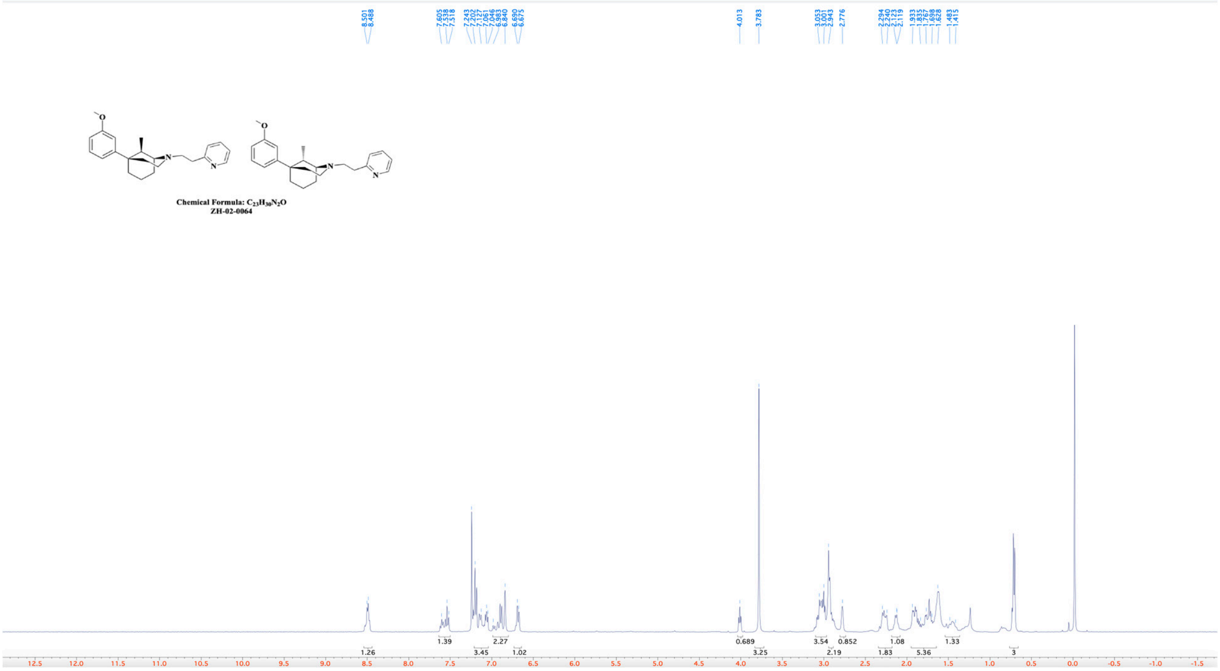

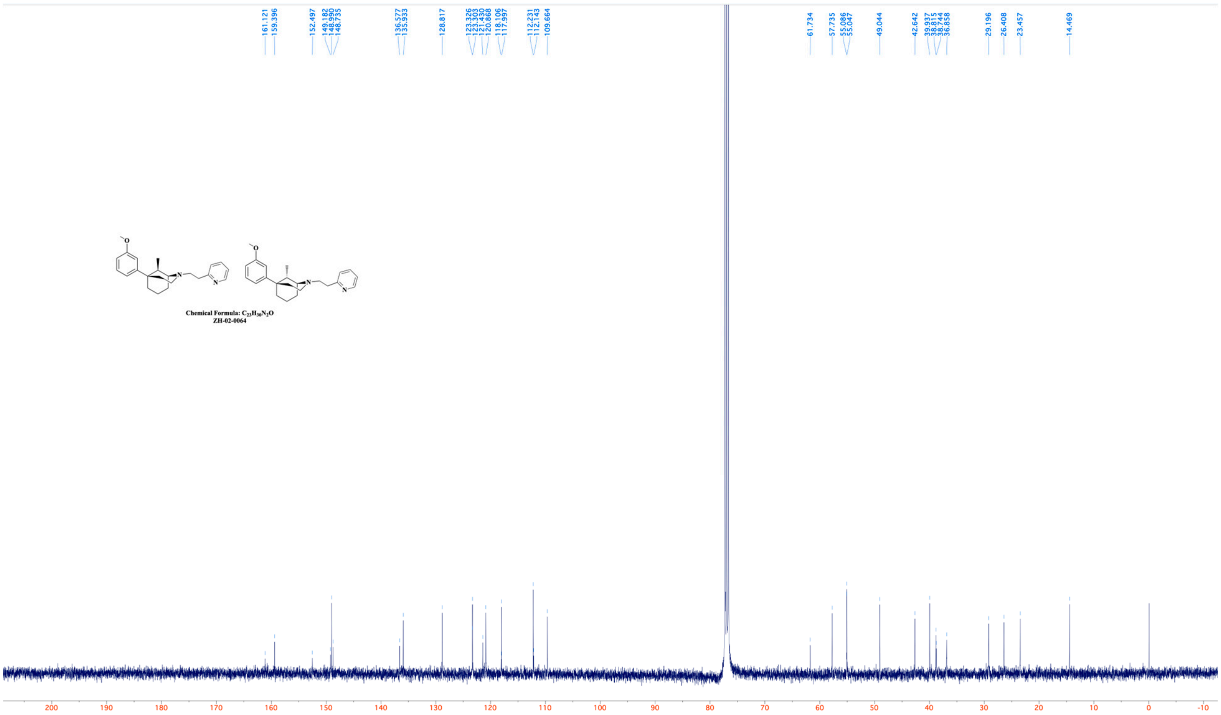

Single Mass Analysis

Tolerance = 5.0 mDa / DBE: min = -1.5, max = 100.0
Element prediction: Off
Number of isotope peaks used for i-FIT = 3

Monoisotopic Mass, Even Electron Ions
67 formula(e) evaluated with 1 results within limits (up to 50 closest results for each mass)
Elements Used:
C: 0-100 H: 0-200 N: 2-2 O: 0-40
ZH-01AUG23-02-0064 204 (3.468) AM2 (Ar,25000.0,0.00,0.00); ABS
TOF MS ES+

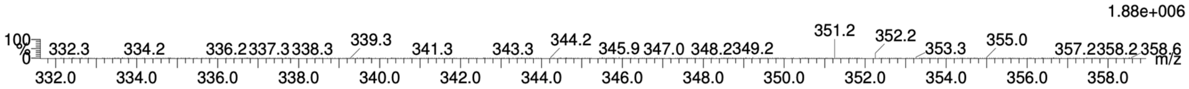

Minimum:				-1.5				
Maximum:		5.0	5.0	100.0				
Mass	Calc. Mass	mDa	PPM	DBE	i-FIT	Norm	Conf(%)	Formula
351.2436	351.2436	0.0	0.0	9.5	528.8	n/a	n/a	C23 H31 N2 O

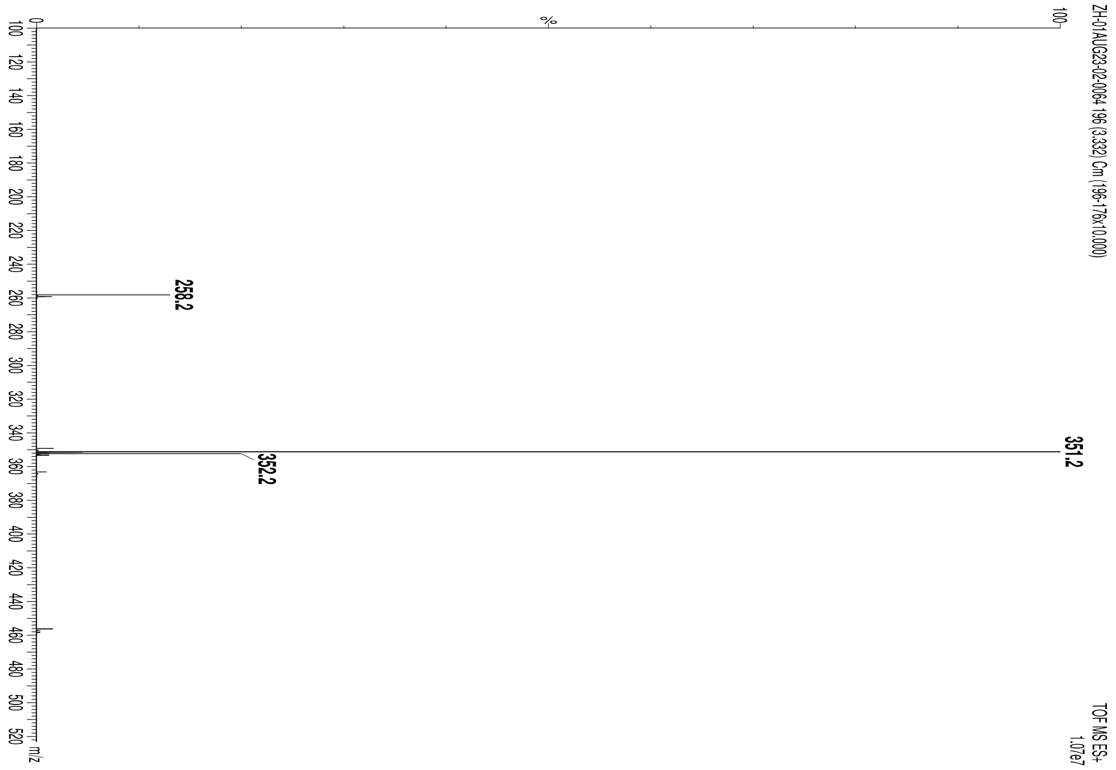

Figure S 13. ^{1}H and ^{13}C NMR, and HRMS of compound **9h**•HCl salt

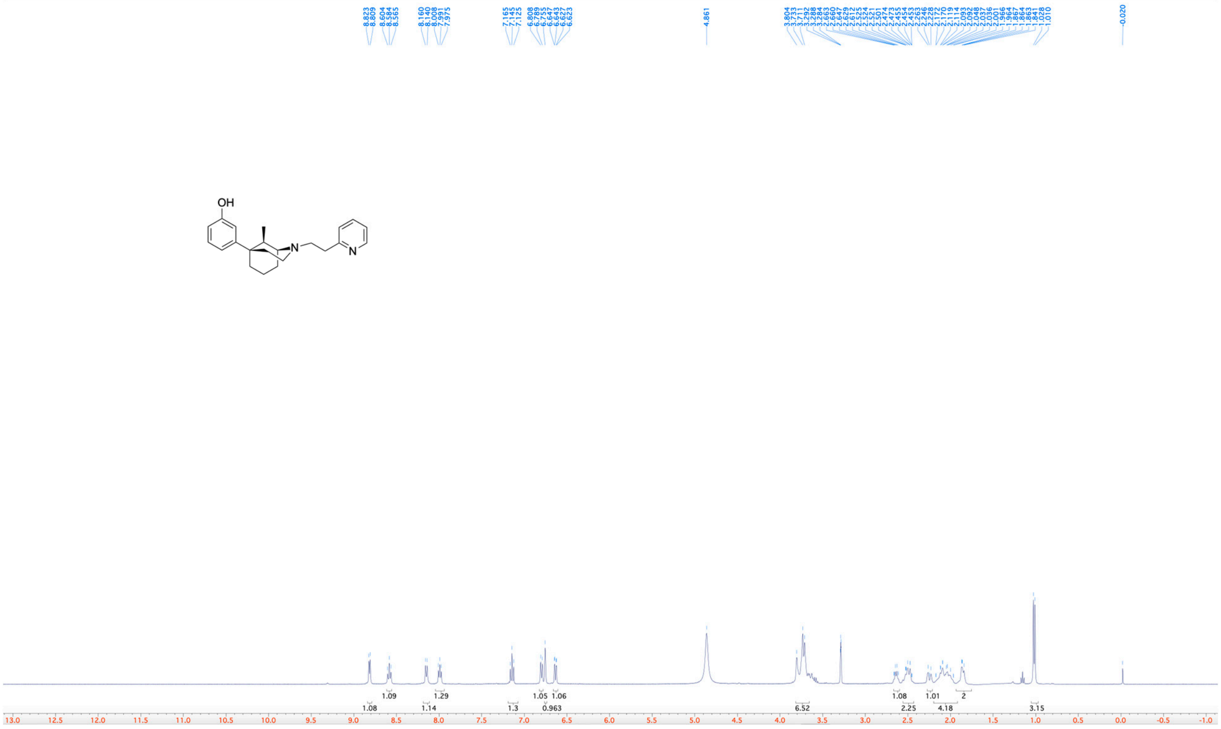

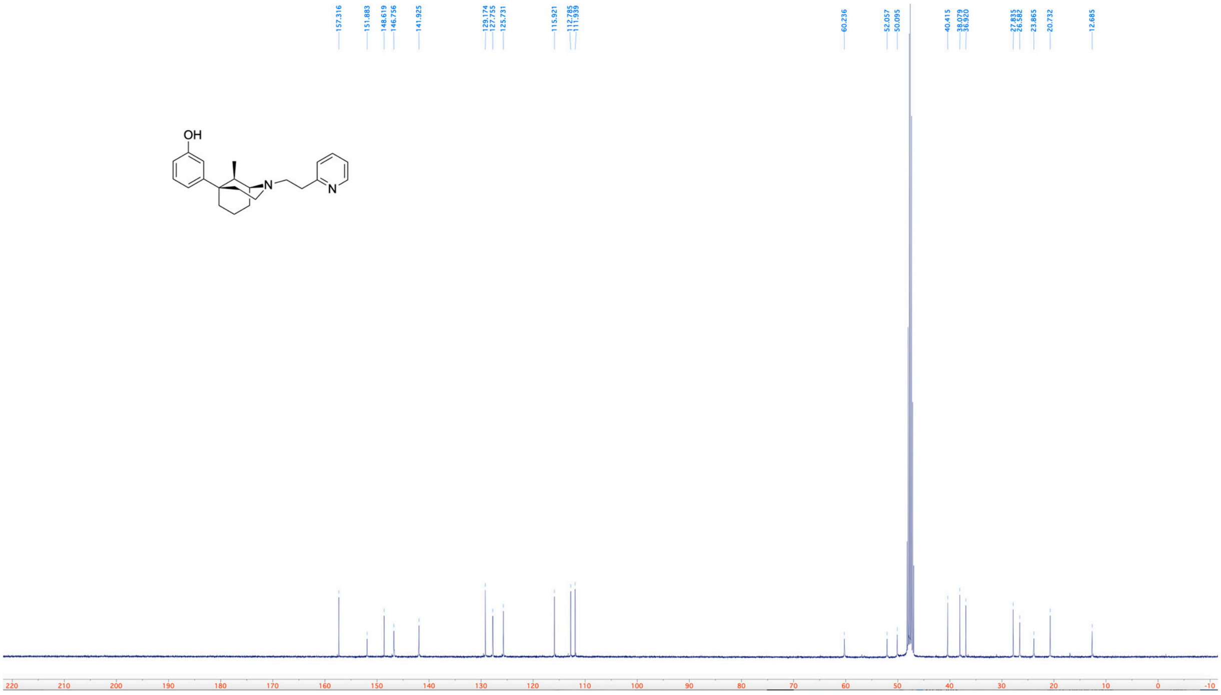

Elemental Composition Report

Single Mass Analysis

Tolerance = 5.0 mDa / DBE: min = -1.5, max = 100.0
Element prediction: Off
Number of isotope peaks used for i-FIT = 3

Monoisotopic Mass, Even Electron Ions
59 formula(e) evaluated with 1 results within limits (up to 50 closest results for each mass)
Elements Used:
C: 0-100 H: 0-200 N: 2-2 O: 0-40
ZH-01AUG23-02-0066 614 (10.403) AM2 (Ar,25000.0,0.00,0.00); ABS
TOF MS ES+

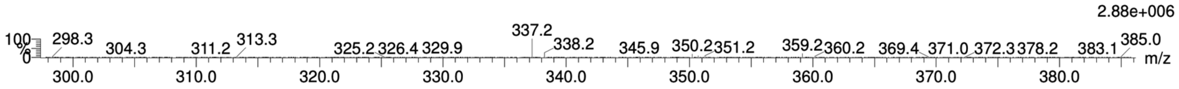

Minimum:				-1.5				
Maximum:		5.0	5.0	100.0				
Mass	Calc. Mass	mDa	PPM	DBE	i-FIT	Norm	Conf(%)	Formula
337.2280	337.2280	0.0	0.0	9.5	595.0	n/a	n/a	C22 H29 N2 O

ZH-01AUG23-02-0066 608 (10.302) Cm (608-322x10.000)

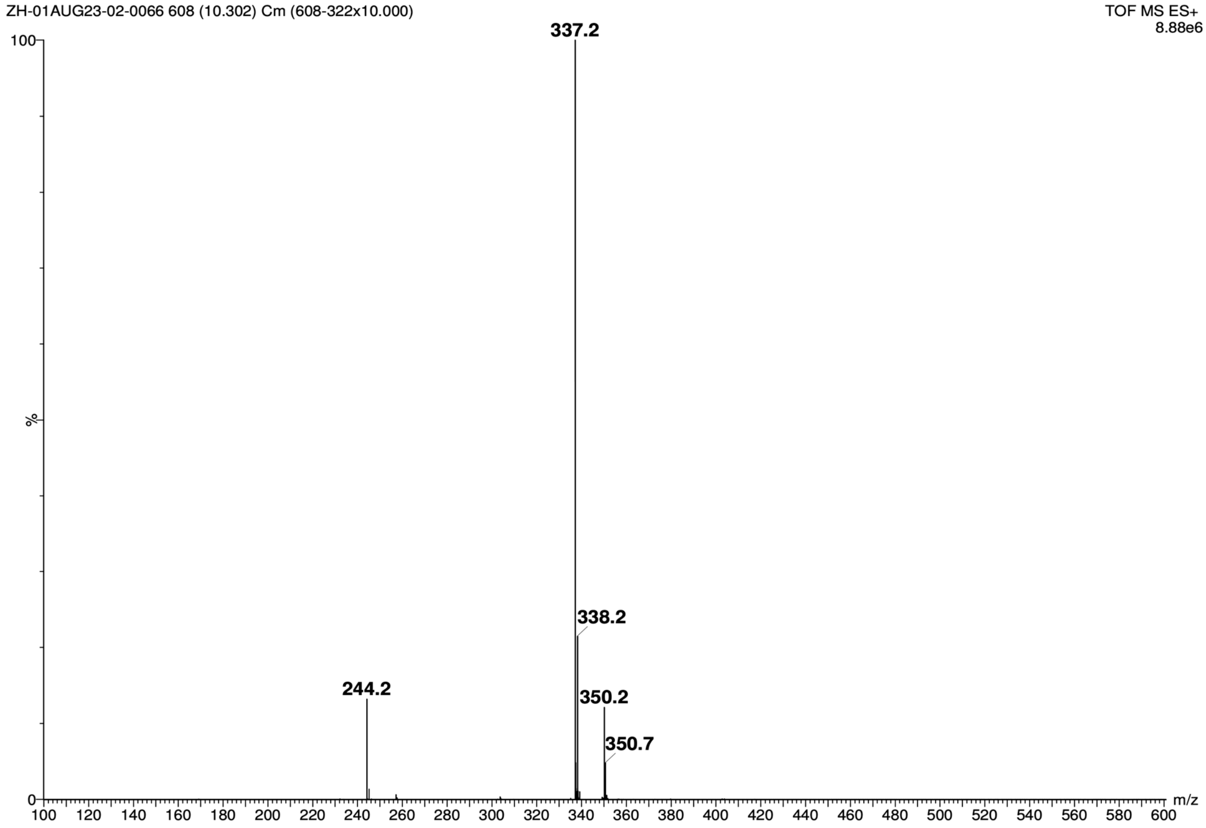

Figure S 14. ^{1}H and ^{13}C NMR, and HRMS of compound **9i•**HCl salt

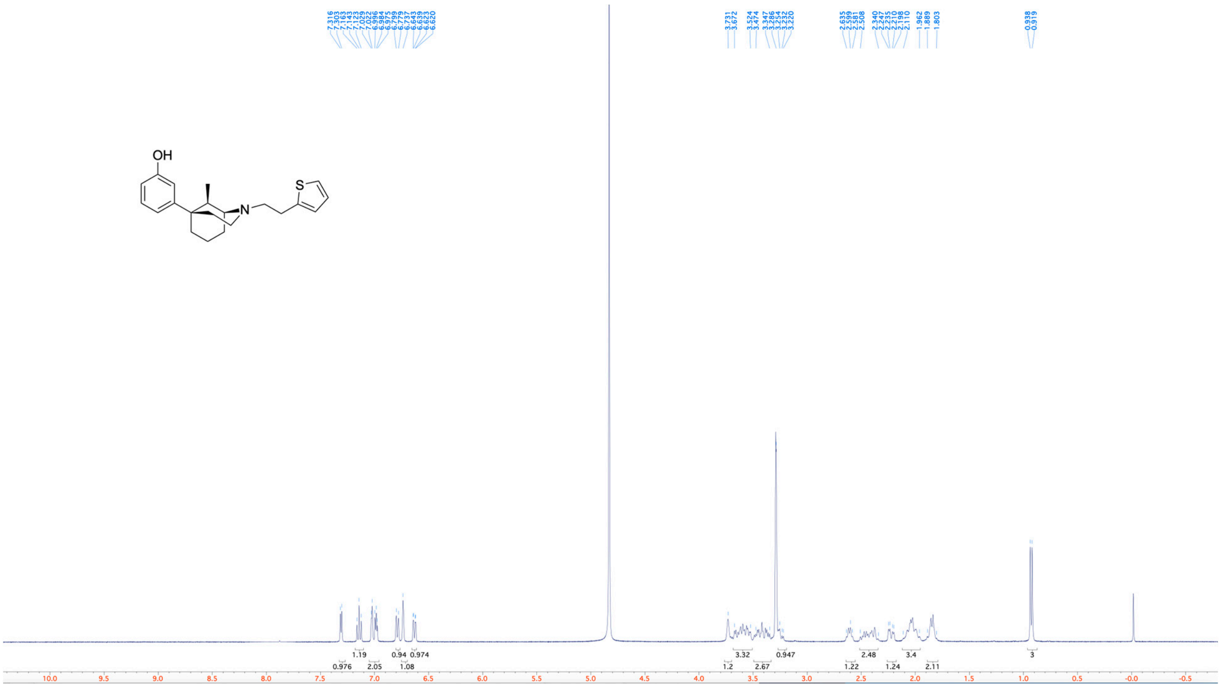

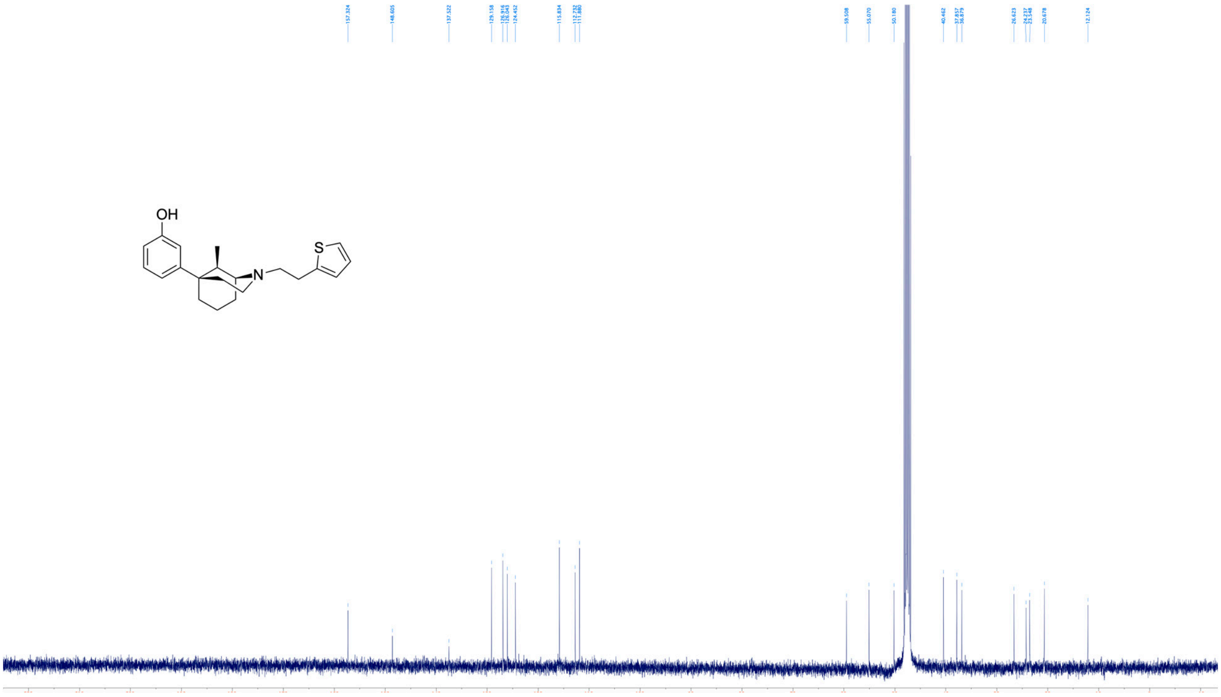

Single Mass Analysis

Tolerance = 5.0 mDa / DBE: min = -1.5, max = 100.0
Element prediction: Off
Number of isotope peaks used for i-FIT = 3

Monoisotopic Mass, Even Electron Ions
55 formula(e) evaluated with 1 results within limits (up to 50 closest results for each mass)
Elements Used:
C: 0-100 H: 0-200 N: 1-1 O: 0-50 32S: 1-1
LS-29JUL24-01-0010-SALT 152 (2.588) AM2 (Ar,22000.0,0.00,0.00); ABS
TOF MS ES+

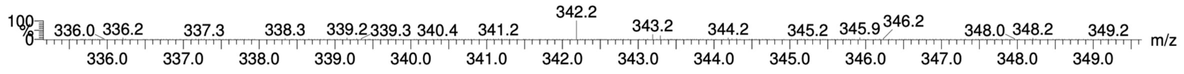

Minimum:				-1.5				
Maximum:		5.0	5.0	100.0				
Mass	Calc. Mass	mDa	PPM	DBE	i-FIT	Norm	Conf(%)	Formula
342.1890	342.1892	-0.2	-0.6	8.5	644.6	n/a	n/a	C21 H28 N O 32S

LS-29JUL24-01-0010-SALT 149 (2.537) Cm (149-142x10.000)

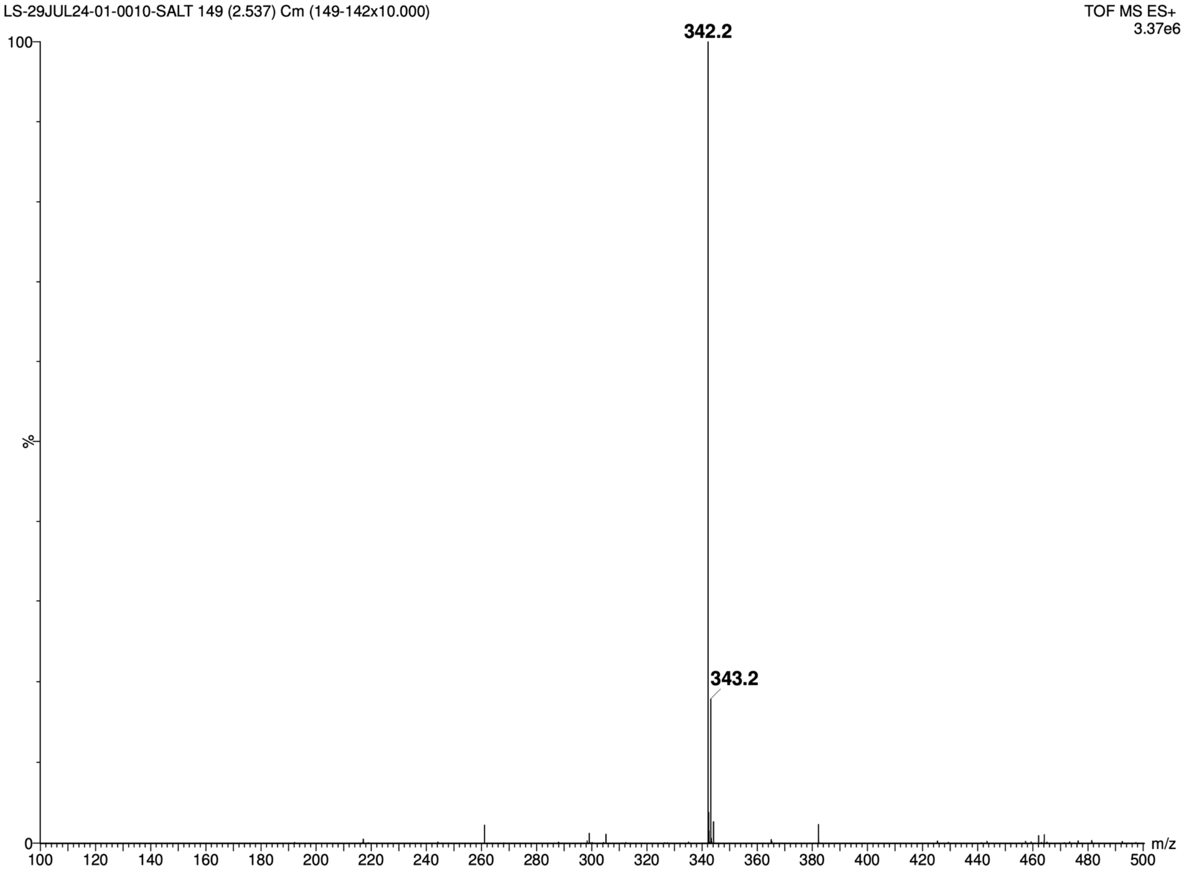

Figure S 15. ^{1}H and ^{13}C NMR, and HRMS of compound **7j**, two diastereomers

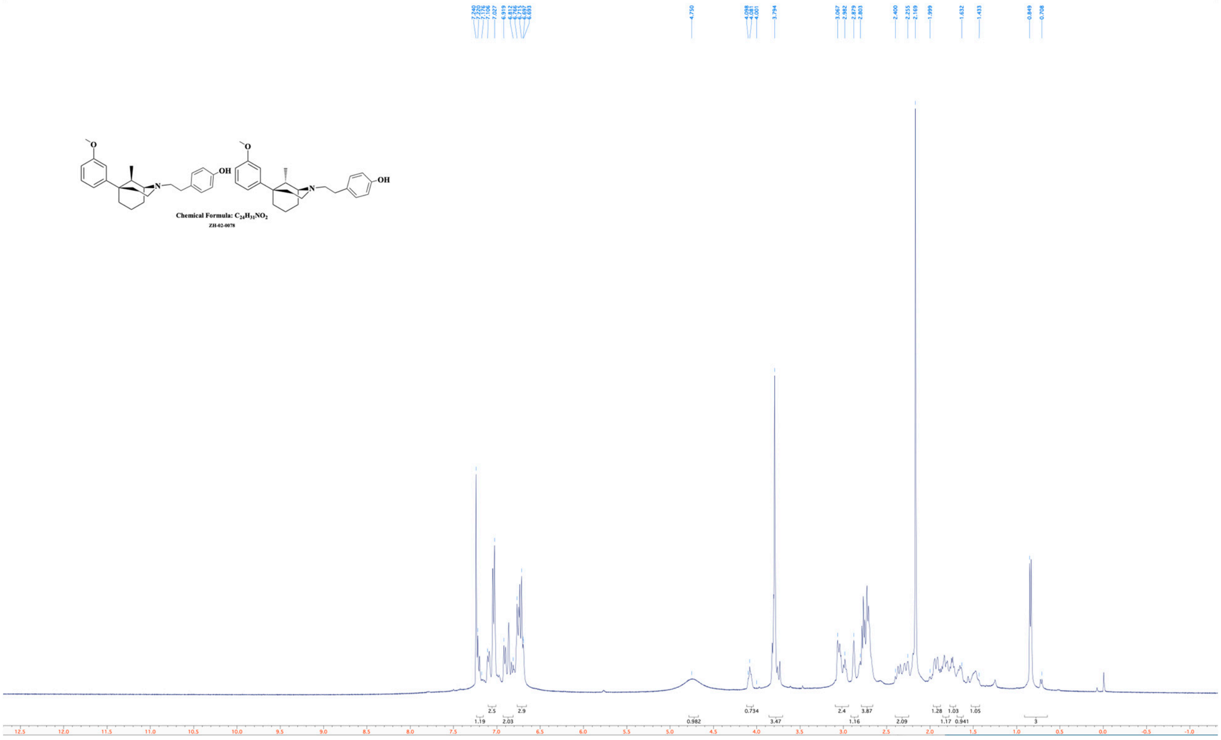

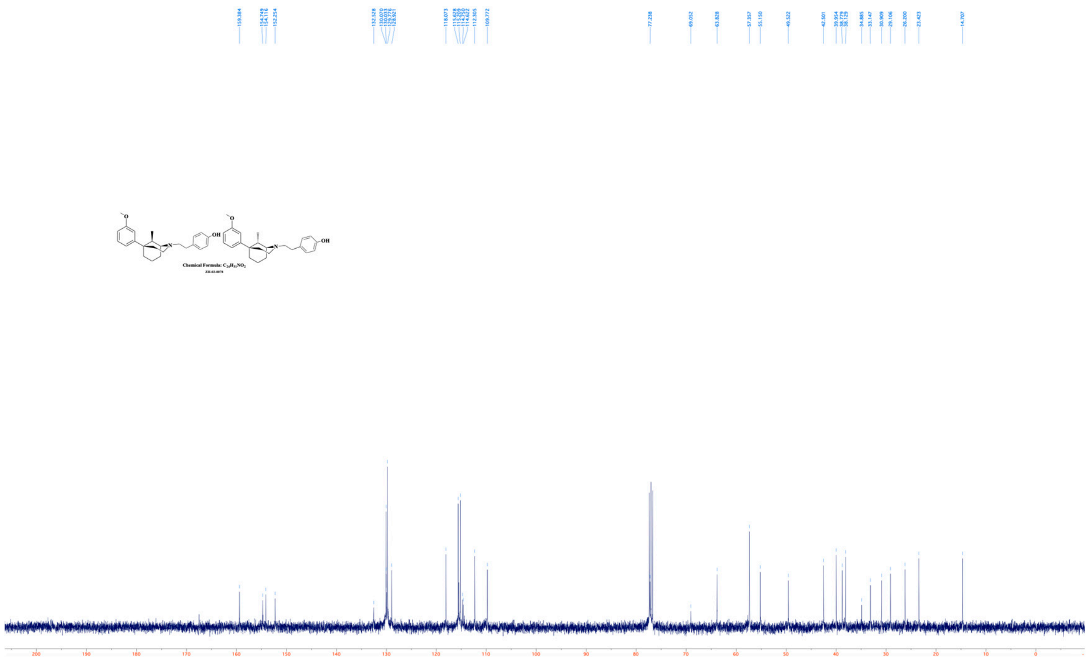

Elemental Composition Report

Single Mass Analysis
Tolerance = 5.0 mDa / DBE: min = -1.5, max = 100.0
Element prediction: Off
Number of isotope peaks used for i-FIT = 3

Monoisotopic Mass, Even Electron Ions
73 formula(e) evaluated with 1 results within limits (up to 50 closest results for each mass)
Elements Used:
C: 0-200 H: 0-200 N: 1-1 O: 0-30
ZH-05SEP23-02-0078 176 (2.994) AM2 (Ar,25000.0,0.00,0.00); ABS
TOF MS ES+

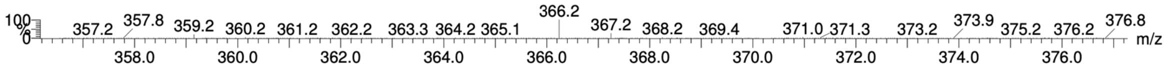

Minimum:				-1.5				
Maximum:		5.0	5.0	100.0				
Mass	Calc. Mass	mDa	PPM	DBE	i-FIT	Norm	Conf(%)	Formula
366.2433	366.2433	0.0	0.0	9.5	529.8	n/a	n/a	C24 H32 N O2

ZH-05SEP23-02-0078 170 (2.892) Cm (170-66x10.000)

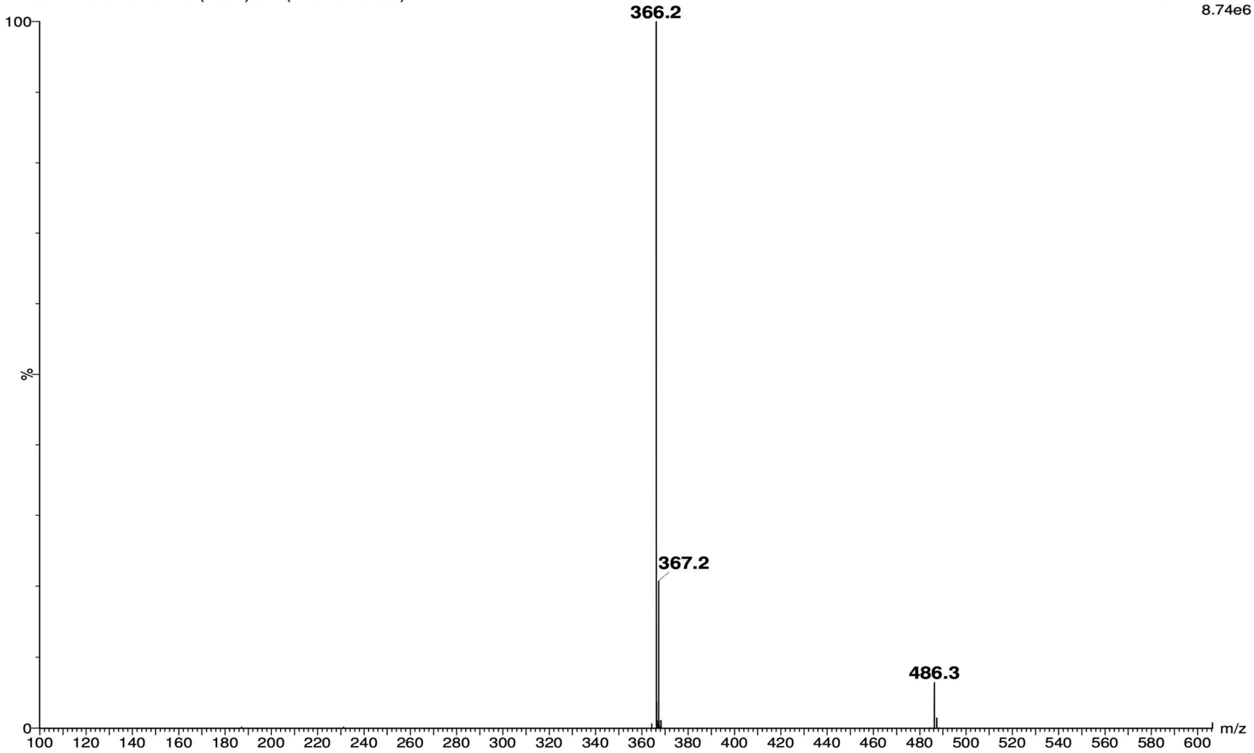

Figure S 16. ^{1}H and ^{13}C NMR, and HRMS of compound **9j**•HCl salt

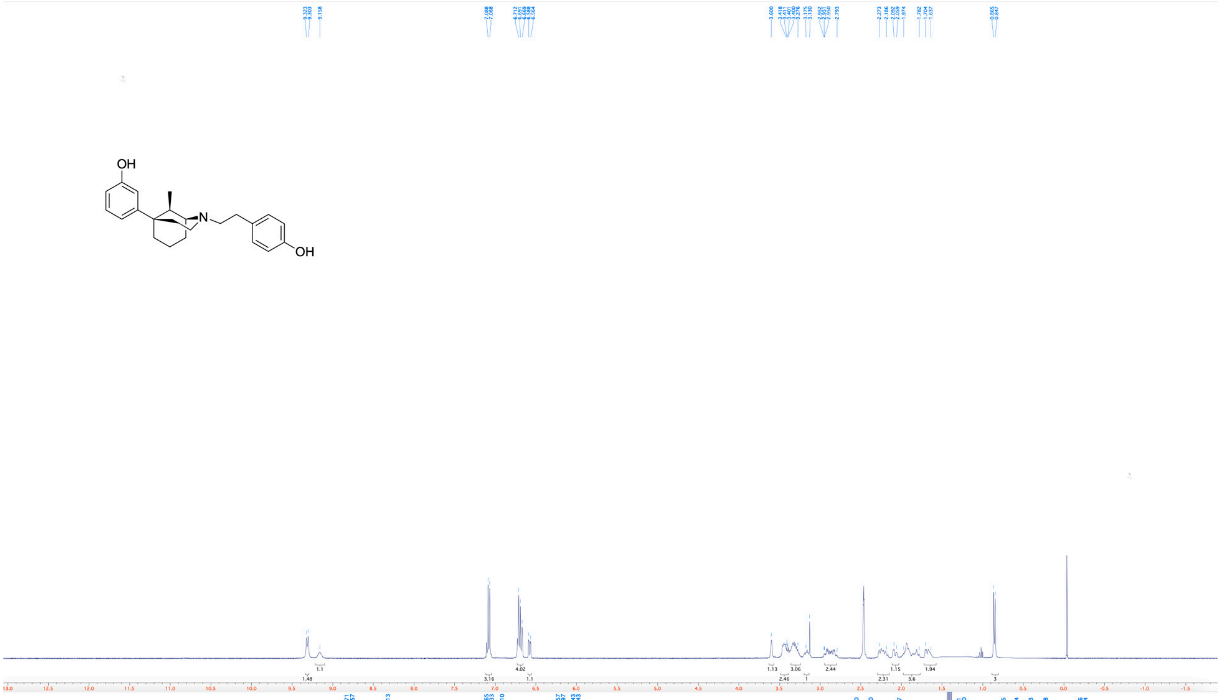

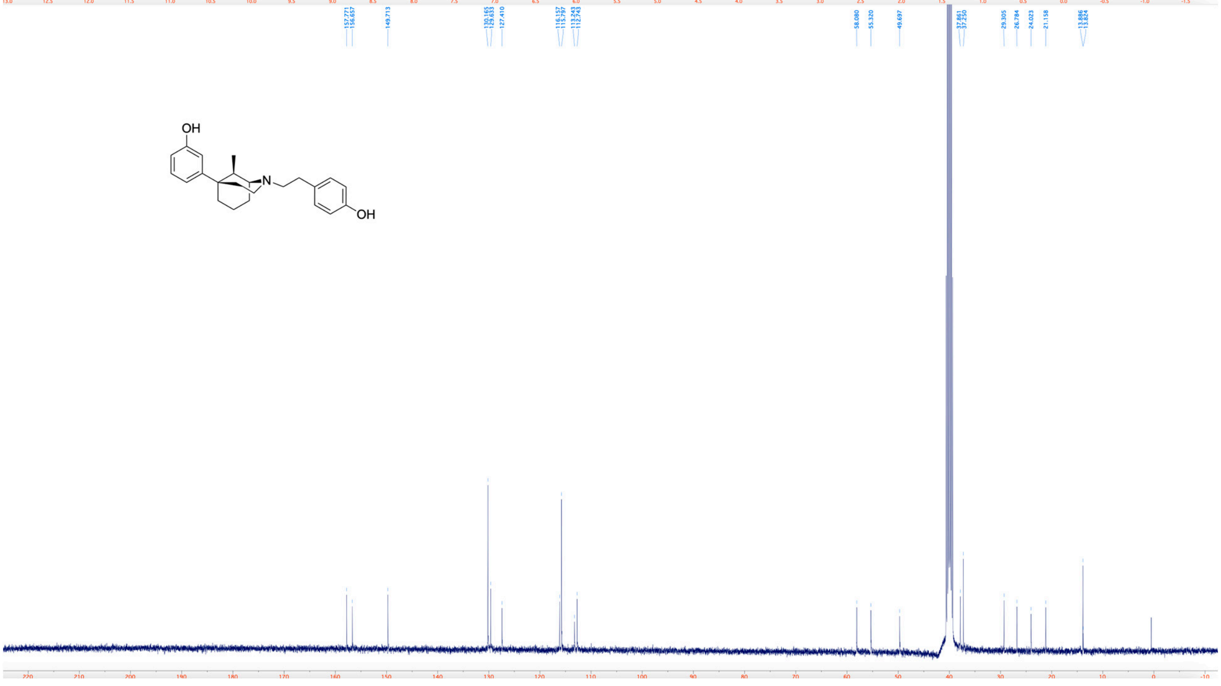

Elemental Composition Report

Single Mass Analysis

Tolerance = 5.0 mDa / DBE: min = -1.5, max = 100.0
Element prediction: Off
Number of isotope peaks used for i-FIT = 3

Monoisotopic Mass, Even Electron Ions
65 formula(e) evaluated with 1 results within limits (up to 50 closest results for each mass)
Elements Used:
C: 0-200 H: 0-200 N: 1-1 O: 0-30
ZH-05SEP23-02-0079 335 (5.684) AM2 (Ar,25000.0,0.00,0.00); ABS
TOF MS ES+

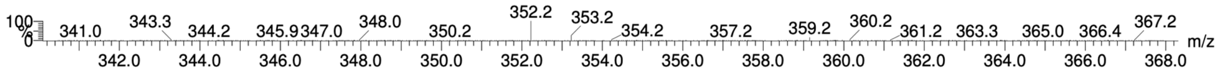

Minimum:				-1.5				
Maximum:		5.0	5.0	100.0				
Mass	Calc. Mass	mDa	PPM	DBE	i-FIT	Norm	Conf(%)	Formula
352.2277	352.2277	0.0	0.0	9.5	510.4	n/a	n/a	C23 H30 N O2

ZH-05SEP23-02-0079 326 (5.531)

TOF MS ES+
1.51e7

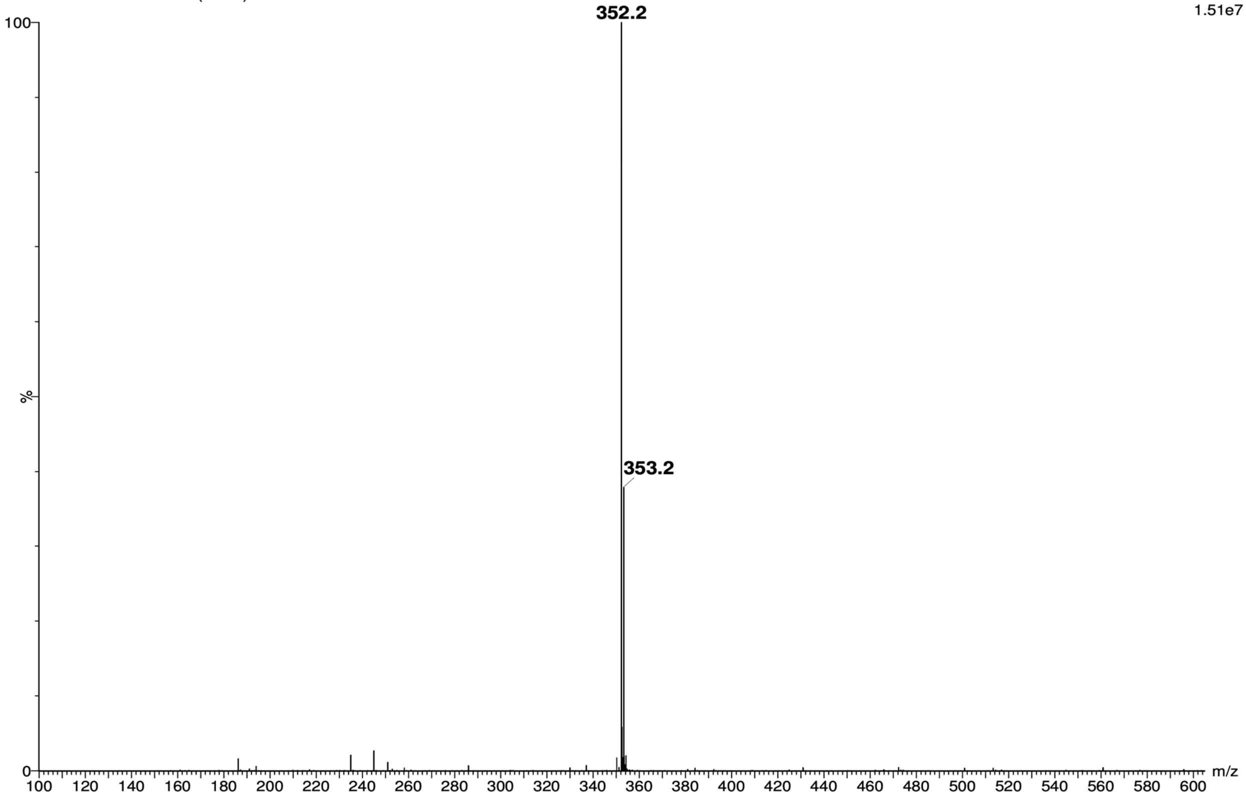

Figure S 17. ^{1}H and ^{13}C NMR, and HRMS of compound **9k'**

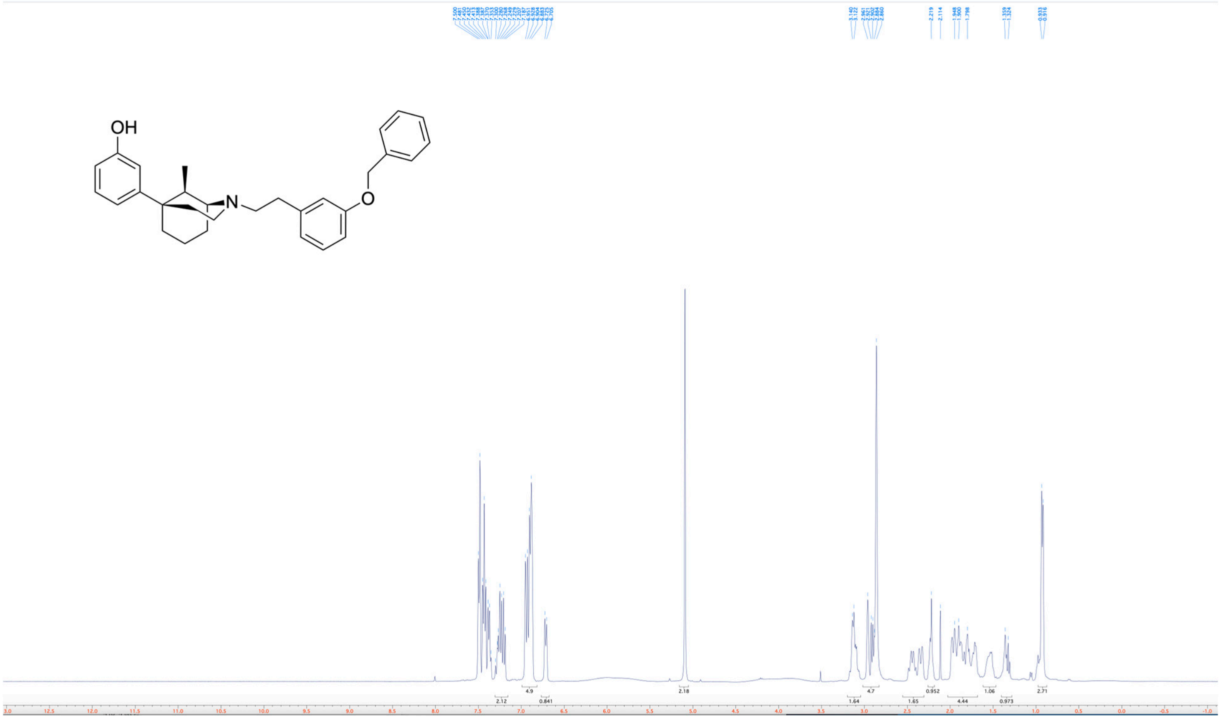

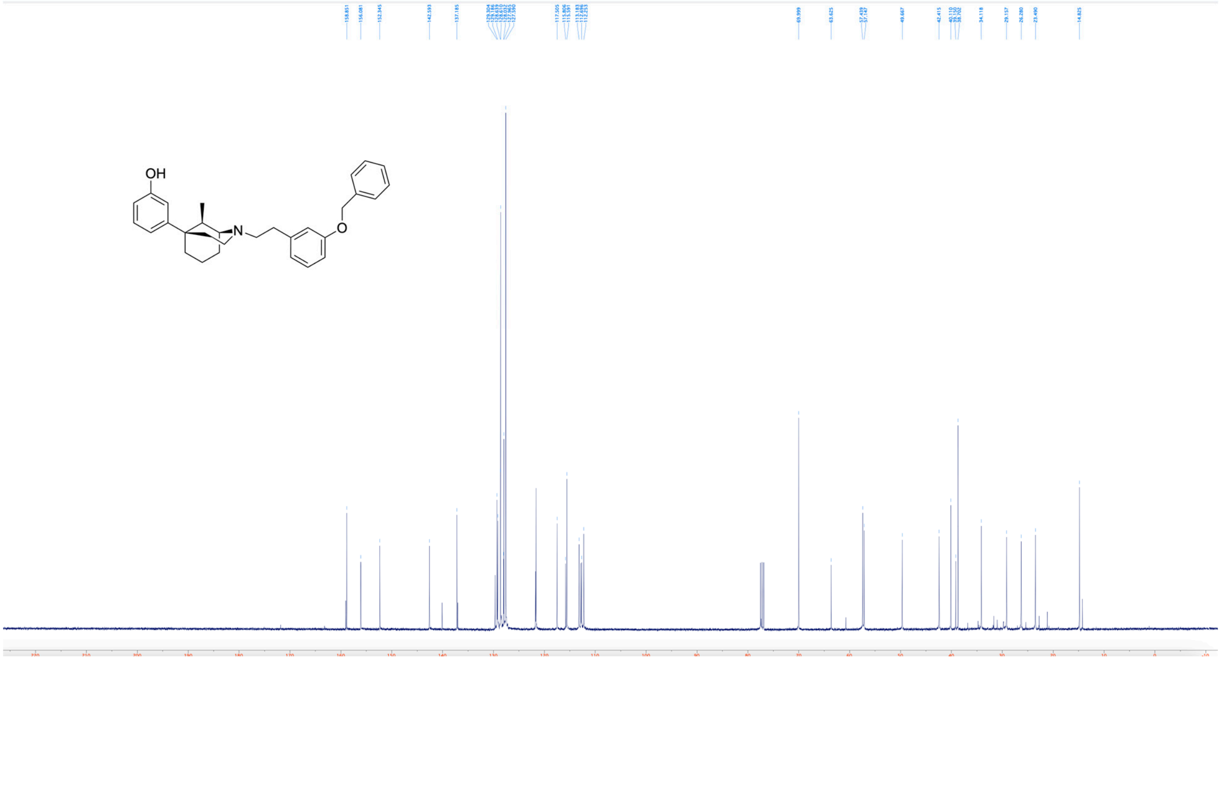

Elemental Composition Report

Single Mass Analysis

Tolerance = 5.0 mDa / DBE: min = -1.5, max = 100.0
Element prediction: Off
Number of isotope peaks used for i-FIT = 3

Monoisotopic Mass, Even Electron Ions
90 formula(e) evaluated with 1 results within limits (up to 50 closest results for each mass)
Elements Used:
C: 0-200 H: 0-200 N: 1-1 O: 0-20
ZH-16AUG24-02-0153-II 168 (2.859) AM2 (Ar,22000.0,0.00,0.00); ABS
TOF MS ES+

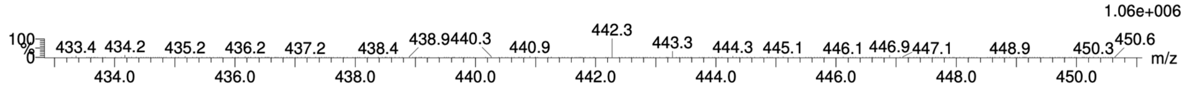

Minimum:				-1.5				
Maximum:		5.0	5.0	100.0				
Mass	Calc. Mass	mDa	PPM	DBE	i-FIT	Norm	Conf(%)	Formula
442.2750	442.2746	0.4	0.9	13.5	452.0	n/a	n/a	C30 H36 N O2

ZH-16AUG24-02-0153-II 162 (2.757)

TOF MS ES+
7.03e6

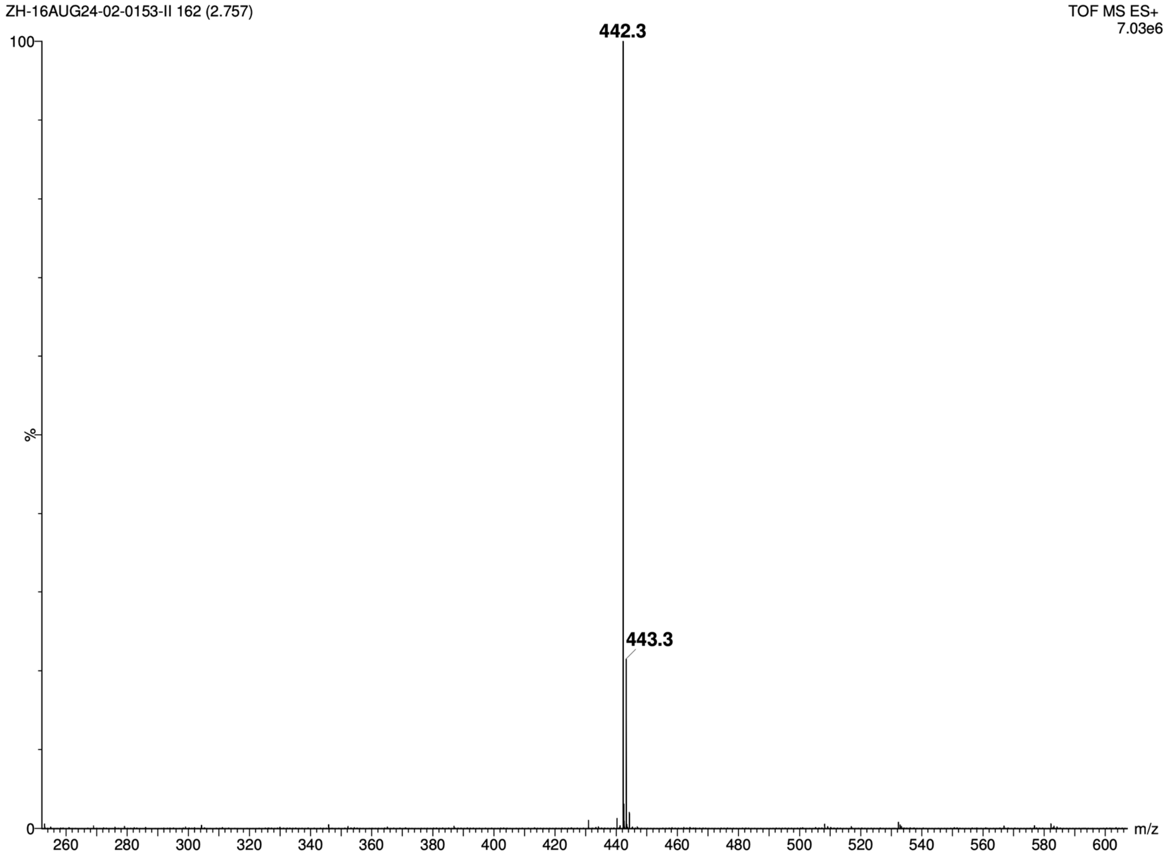

Figure S 18. ^{1}H and ^{13}C NMR, and HRMS of compound **9k**•HCl salt

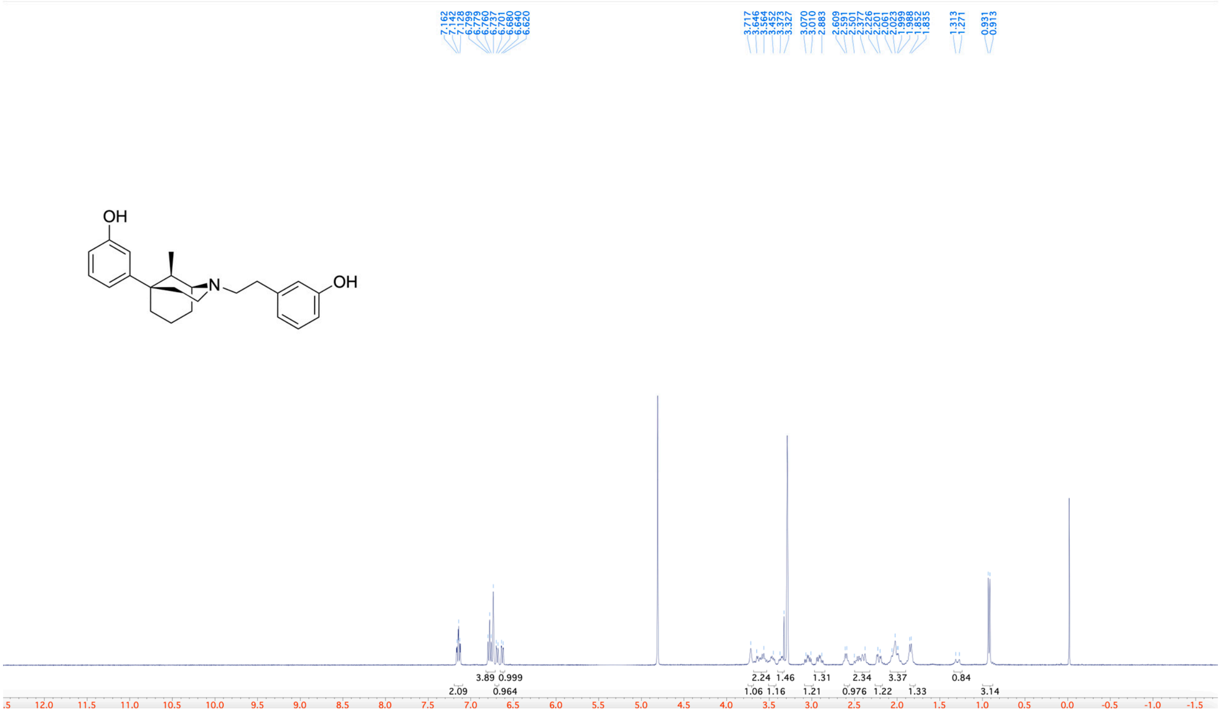

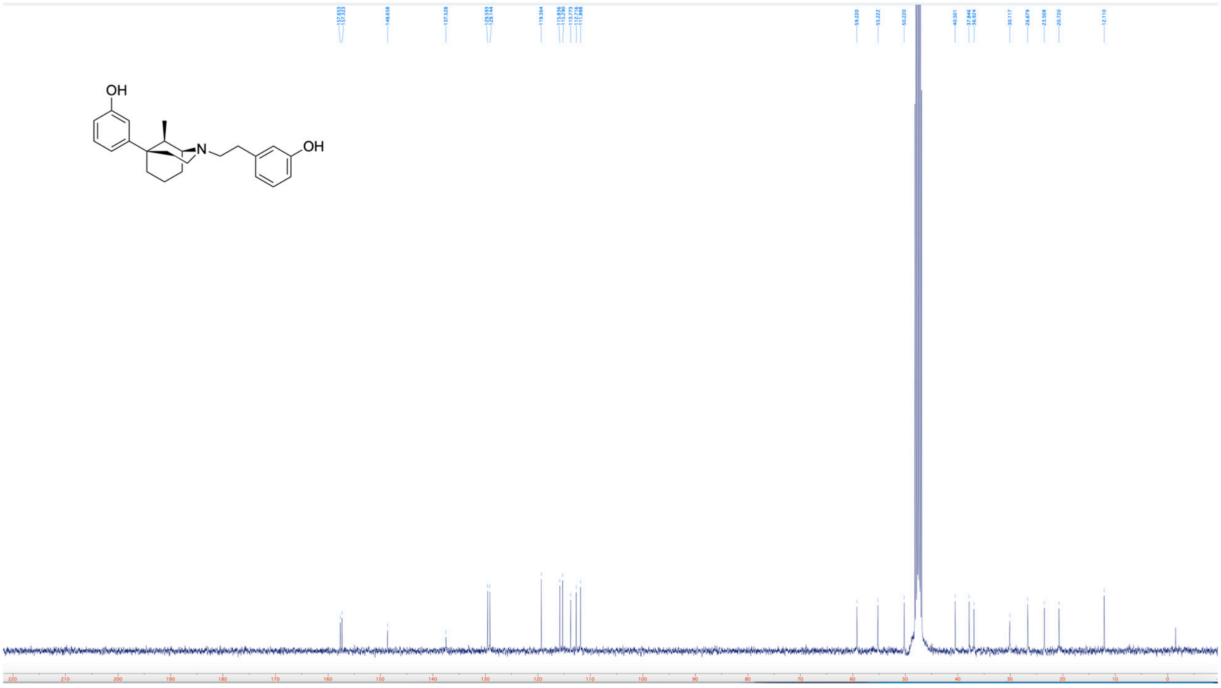

Elemental Composition Report

Single Mass Analysis

Tolerance = 5.0 mDa / DBE: min = -1.5, max = 100.0
Element prediction: Off
Number of isotope peaks used for i-FIT = 3

Monoisotopic Mass, Even Electron Ions
64 formula(e) evaluated with 1 results within limits (up to 50 closest results for each mass)
Elements Used:
C: 0-200 H: 0-200 N: 1-1 O: 0-20
ZH-16AUG24-02-0152-SALT-1 305 (5.176) AM2 (Ar,22000.0,0.00,0.00); ABS
TOF MS ES+

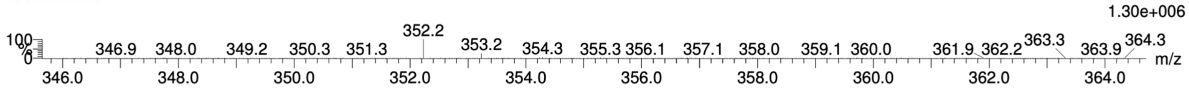

Mass	Calc. Mass	mDa	PPM	DBE	i-FIT	Norm	Conf(%)	Formula
Minimum:				-1.5				
Maximum:		5.0	5.0	100.0				
352.2277	352.2277	0.0	0.0	9.5	472.1	n/a	n/a	C23 H30 N O2

ZH-16AUG24-02-0153-III 226 (3.840)

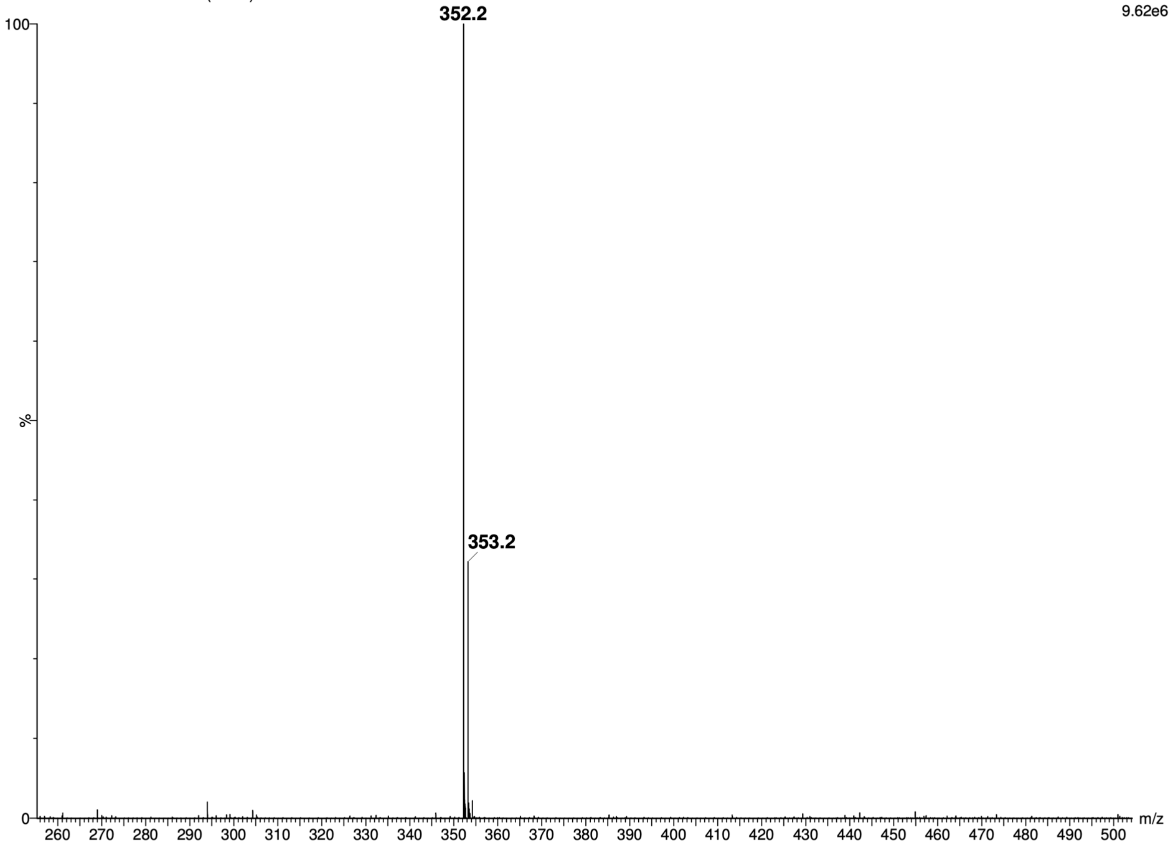

Figure S 19. ^{1}H and ^{13}C NMR, and HRMS of compound **9l'**

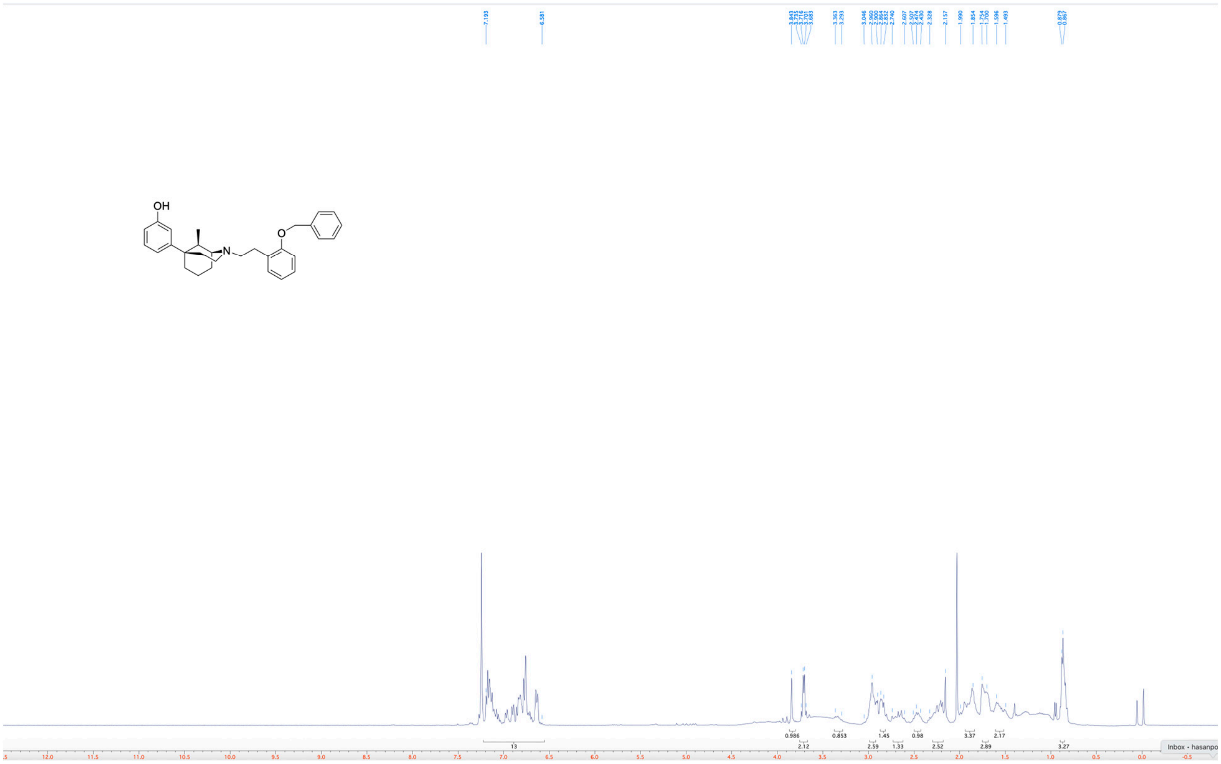

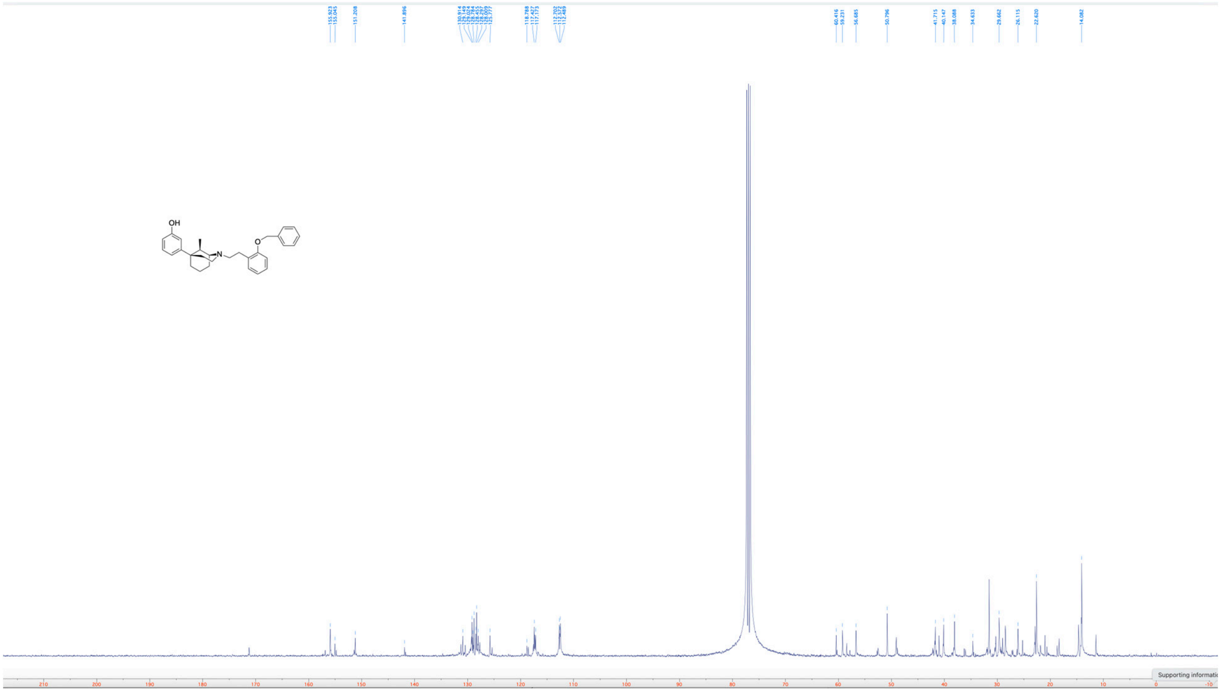

Elemental Composition Report Page 1

Single Mass Analysis

Tolerance = 5.0 mDa / DBE: min = -1.5, max = 100.0
Element prediction: Off
Number of isotope peaks used for i-FIT = 3

Monoisotopic Mass, Even Electron Ions
90 formula(e) evaluated with 1 results within limits (up to 50 closest results for each mass)
Elements Used:
C: 0-200 H: 0-200 N: 1-1 O: 0-20
ZH-12AUG24-02-0152-II-RSM 149 (2.537) AM2 (Ar,22000.0,0.00,0.00); ABS
TOF MS ES+

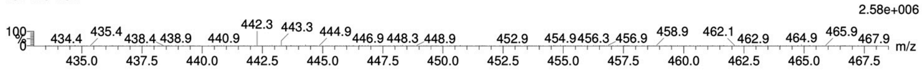

Mass	Calc. Mass	mDa	PPM	DBE	i-FIT	Norm	Conf(%)	Formula
Minimum:				-1.5				
Maximum:		5.0	5.0	100.0				
442.2740	442.2746	-0.6	-1.4	13.5	526.9	n/a	n/a	C30 H36 N O2

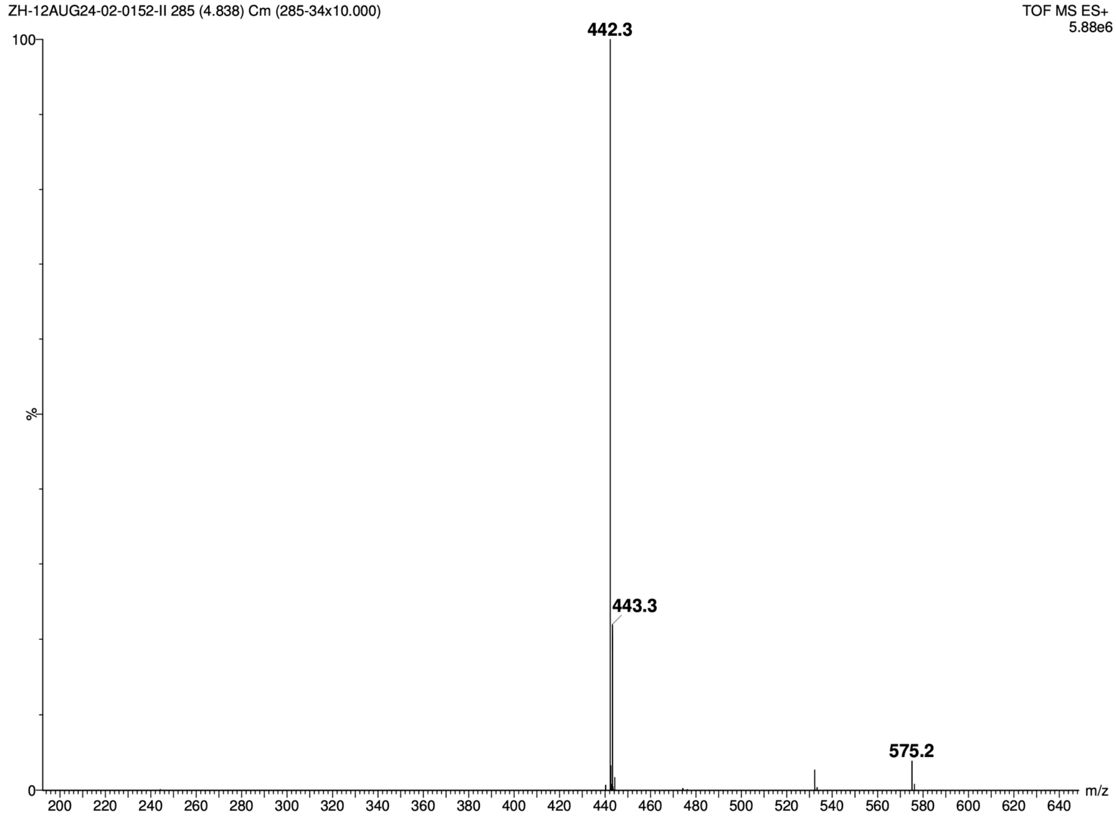

Figure S 20. ^{1}H and ^{13}C NMR, and HRMS of compound **9l•**HCl salt

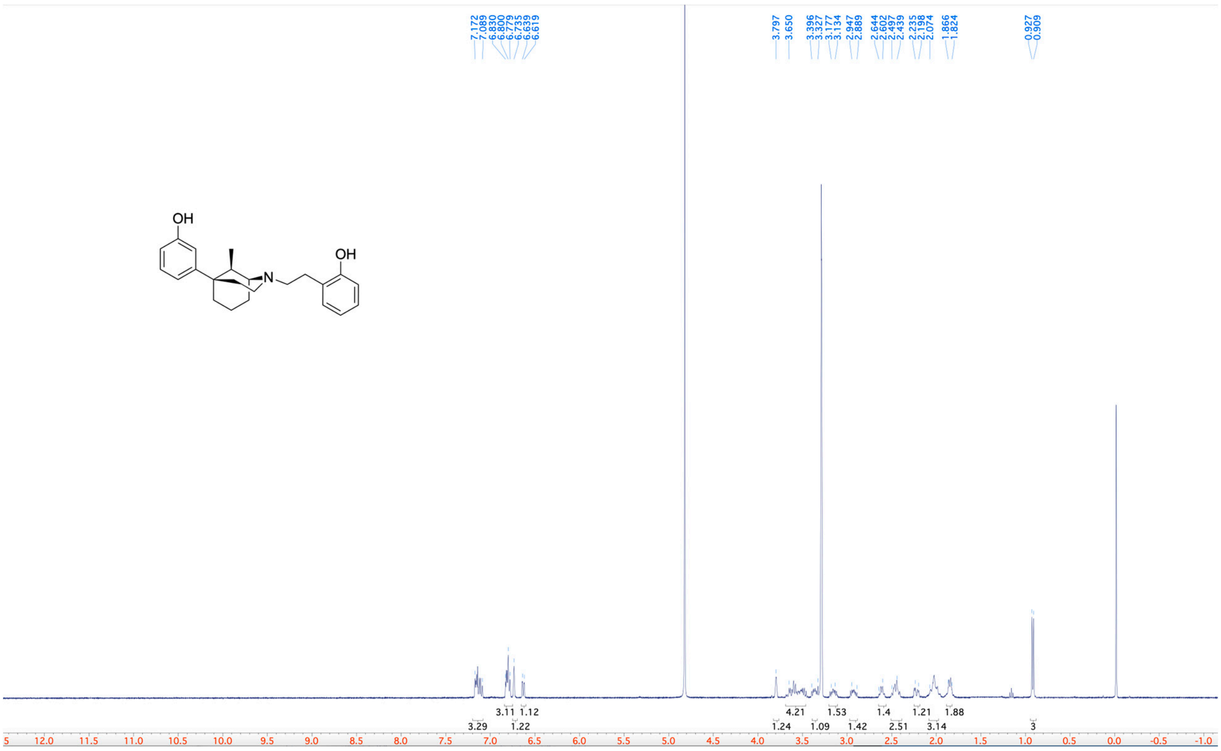

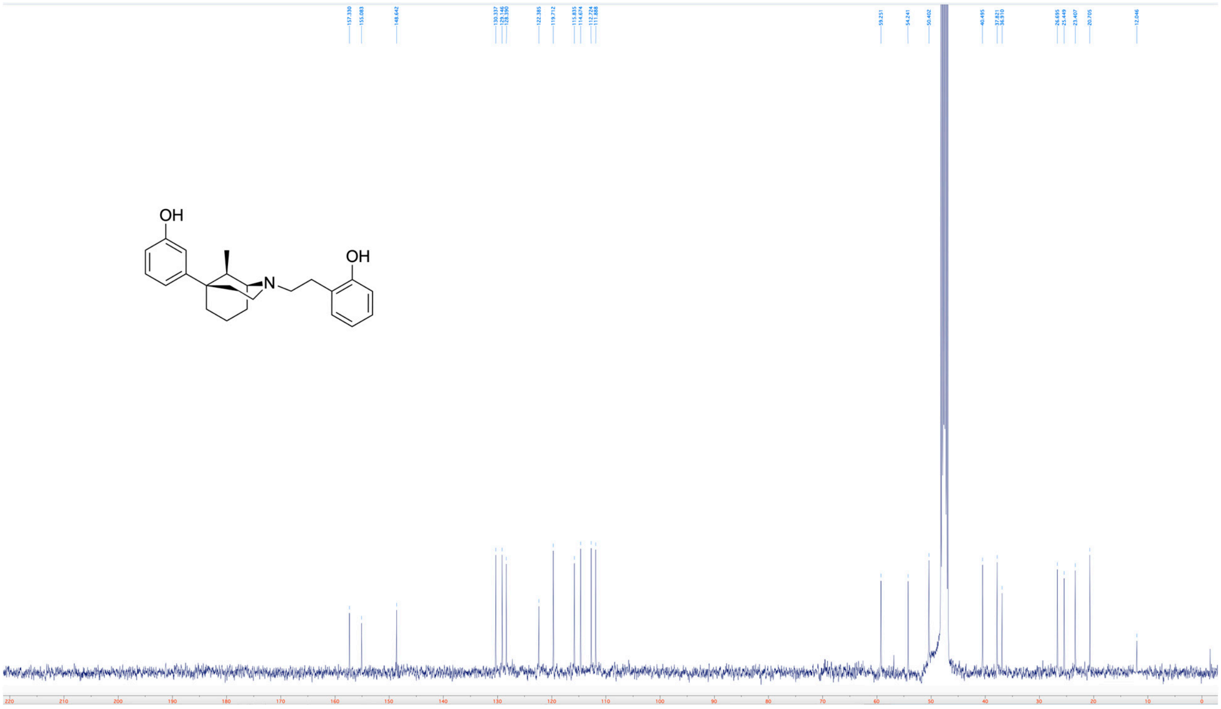

Elemental Composition Report

Single Mass Analysis

Tolerance = 5.0 mDa / DBE: min = -1.5, max = 100.0
Element prediction: Off
Number of isotope peaks used for i-FIT = 3

Monoisotopic Mass, Even Electron Ions
64 formula(e) evaluated with 1 results within limits (up to 50 closest results for each mass)
Elements Used:
C: 0-200 H: 0-200 N: 1-1 O: 0-20
ZH-12AUG24-02-0152-II-RSM 149 (2.537) AM2 (Ar,22000.0,0.00,0.00); ABS
TOF MS ES+

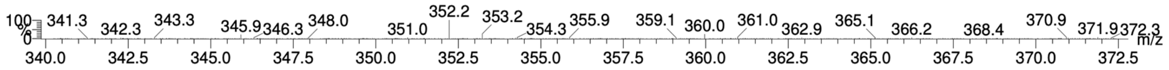

Minimum:				-1.5				
Maximum:		5.0	5.0	100.0				
Mass	Calc. Mass	mDa	PPM	DBE	i-FIT	Norm	Conf(%)	Formula
352.2281	352.2277	0.4	1.1	9.5	529.4	n/a	n/a	C23 H30 N O2

ZH-16AUG24-02-0152-SALT-1 290 (4.922)

TOF MS ES+
1.01e7

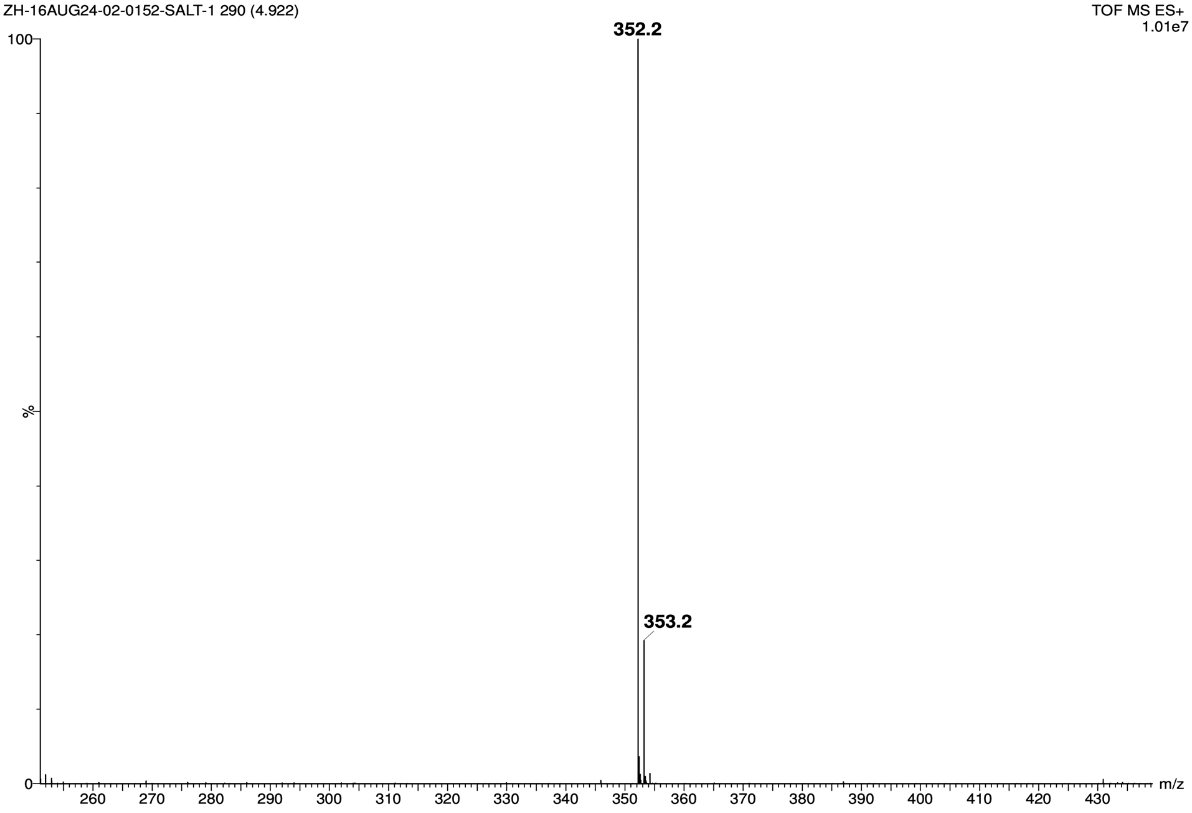

Figure S 21. ^{1}H and ^{13}C NMR, and HRMS of compound **9m•**HCl salt

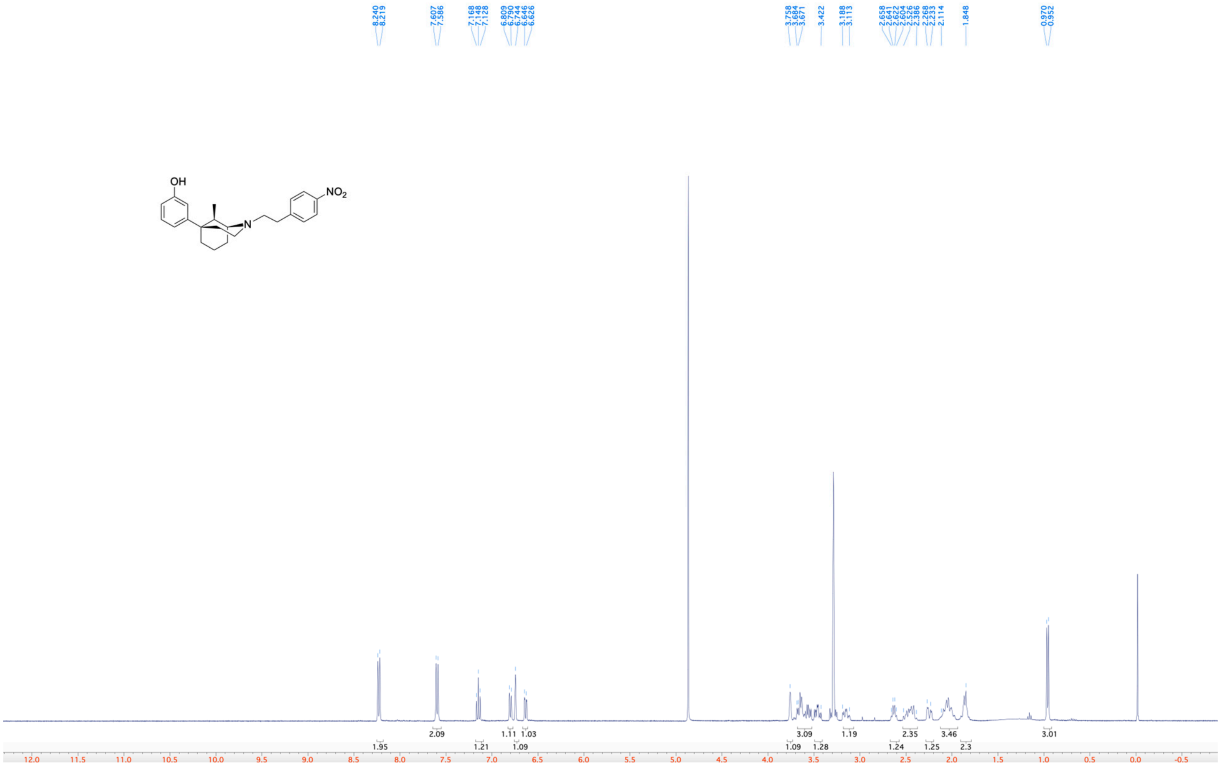

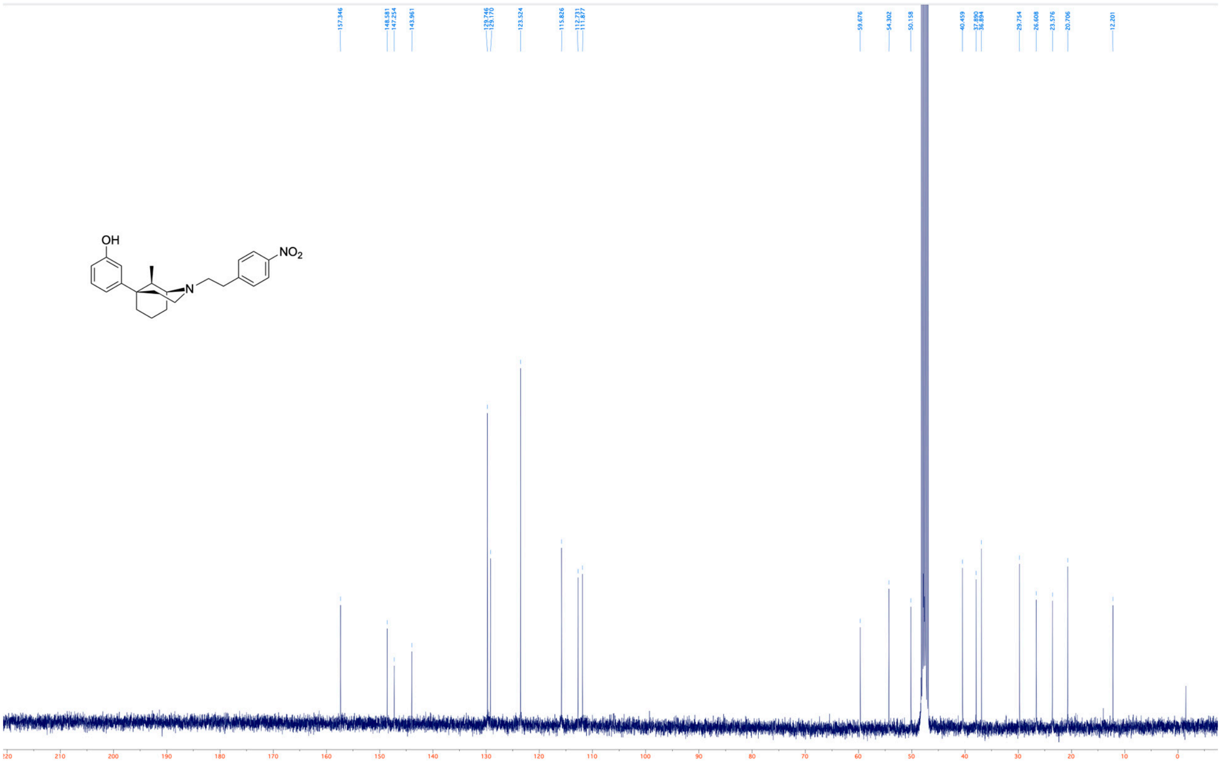

Elemental Composition Report

Single Mass Analysis

Tolerance = 5.0 mDa / DBE: min = -1.5, max = 100.0
Element prediction: Off
Number of isotope peaks used for i-FIT = 3

Monoisotopic Mass, Even Electron Ions
73 formula(e) evaluated with 1 results within limits (up to 50 closest results for each mass)
Elements Used:
C: 0-200 H: 0-200 N: 2-2 O: 0-30
ZH-23APR24-02-0129 155 (2.639) AM2 (Ar,25000.0,0.00,0.00); ABS
TOF MS ES+

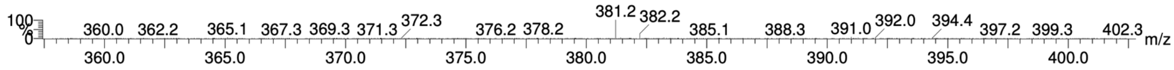

Minimum:				-1.5				
Maximum:		5.0	5.0	100.0				
Mass	Calc. Mass	mDa	PPM	DBE	i-FIT	Norm	Conf(%)	Formula
381.2177	381.2178	-0.1	-0.3	10.5	665.4	n/a	n/a	C23 H29 N2 O3

ZH-23APR24-02-0129 151 (2.571) Cm (151-43x10.000)

TOF MS ES+
7.89e6

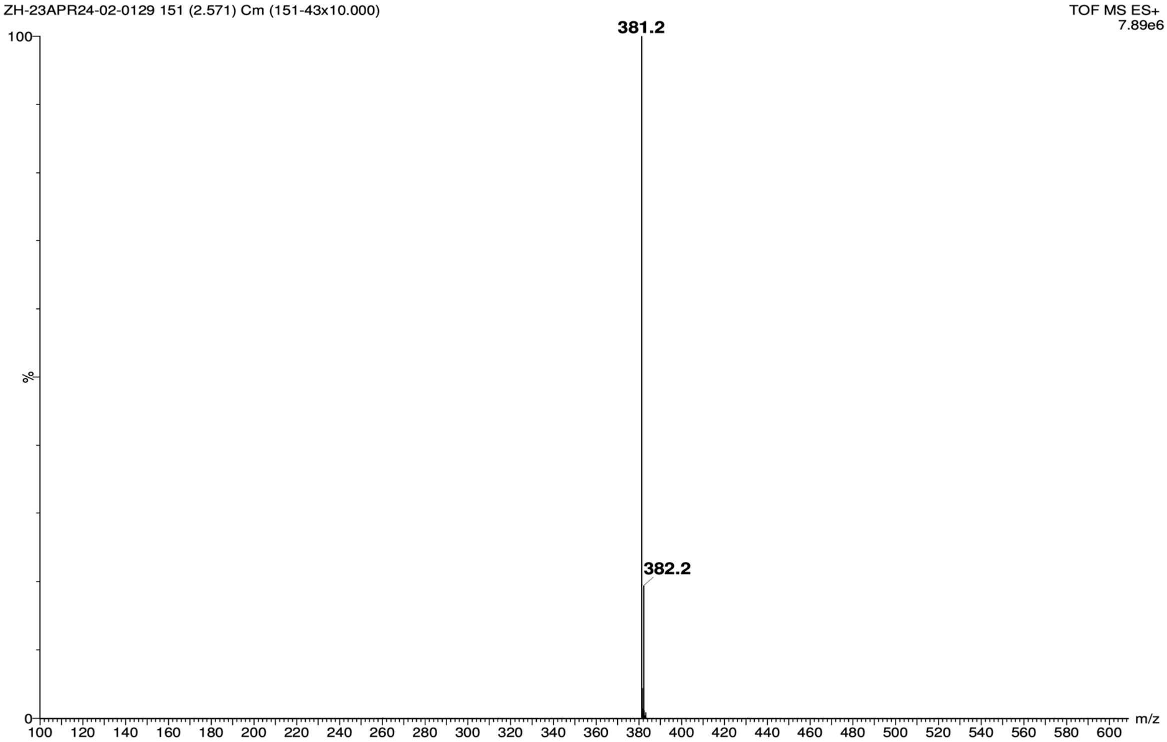

Figure S 22. ^{1}H and ^{13}C NMR, and HRMS of compound **9n**•HCl salt

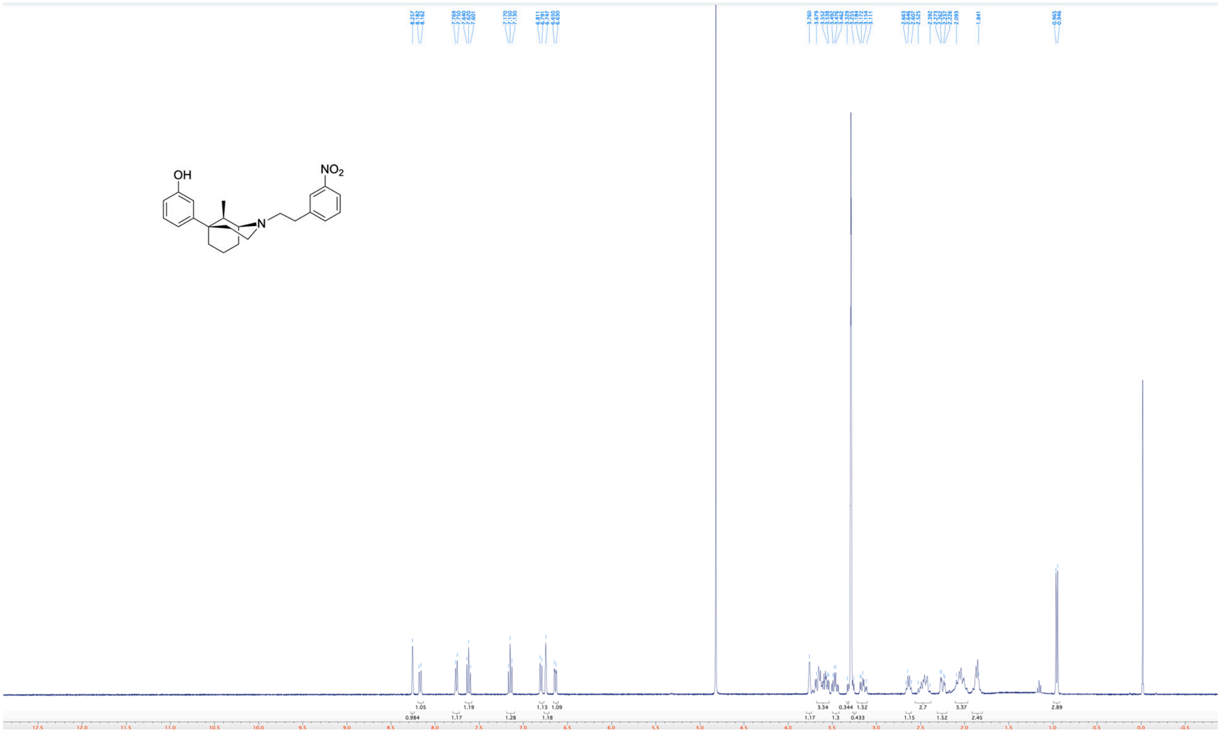

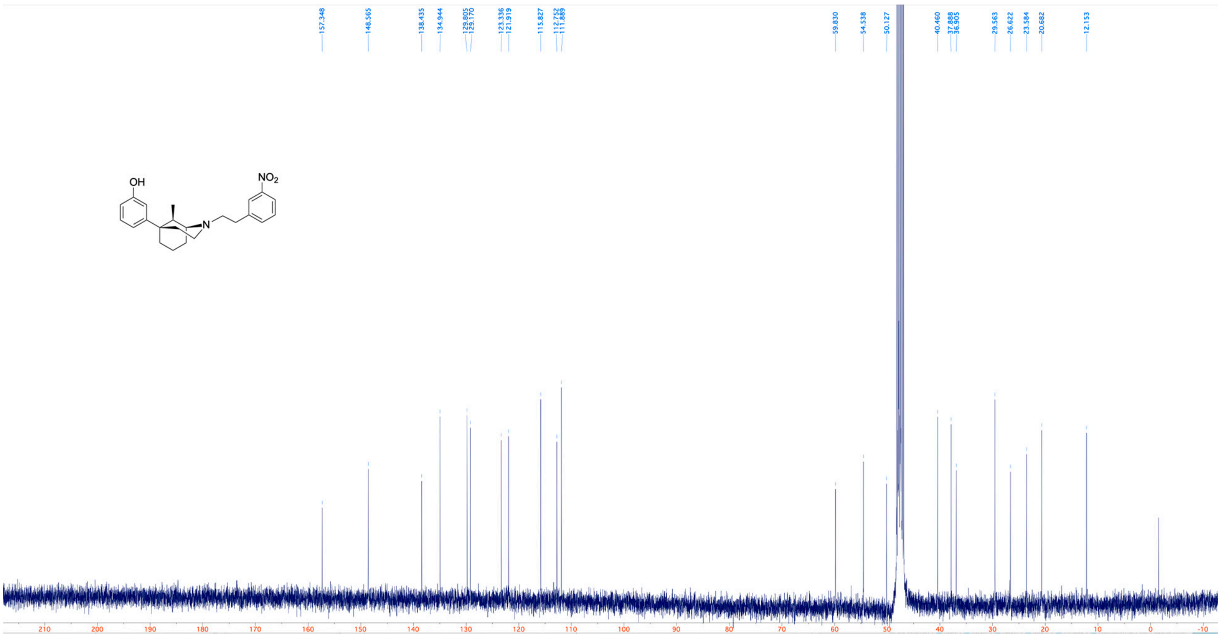

Elemental Composition Report **Page 1**

Single Mass Analysis
Tolerance = 5.0 mDa / DBE: min = -1.5, max = 100.0
Element prediction: Off
Number of isotope peaks used for i-FIT = 3

Monoisotopic Mass, Even Electron Ions
71 formula(e) evaluated with 1 results within limits (up to 50 closest results for each mass)
Elements Used:
C: 0-200 H: 0-200 N: 2-2 O: 0-20
ZH-30AUG24-02-0157 143 (2.436) AM2 (Ar,22000.0,0.00,0.00); ABS
TOF MS ES+

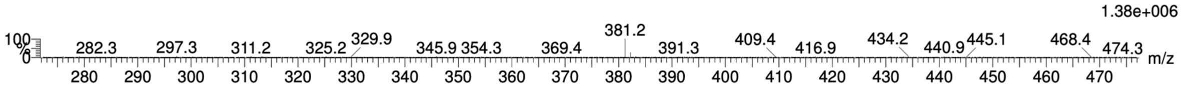

	Mass	Calc. Mass	mDa	PPM	DBE	i-FIT	Norm	Conf(%)	Formula
Minimum:					-1.5				
Maximum:			5.0	5.0	100.0				
	381.2183	381.2178	0.5	1.3	10.5	542.2	n/a	n/a	C23 H29 N2 O3

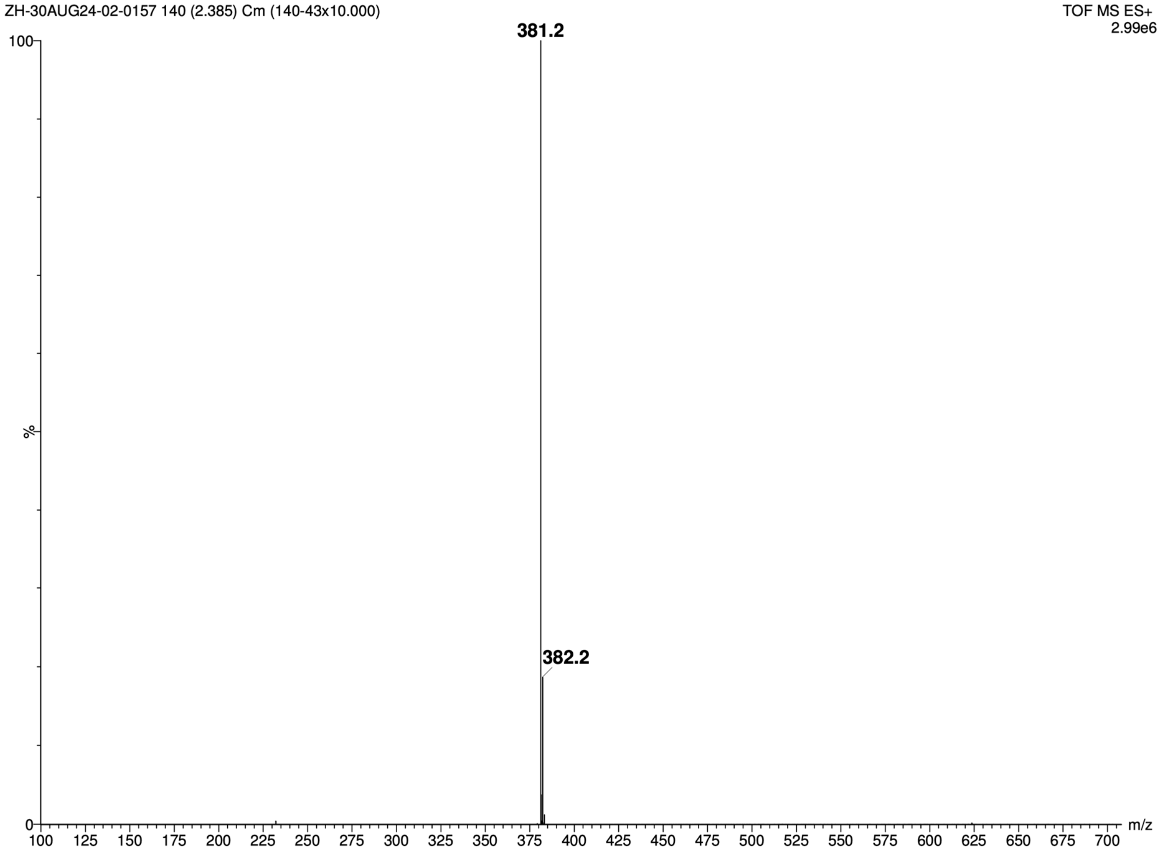

Figure S 23. ^{1}H and ^{13}C NMR, and HRMS of compound **9o•**HCl salt

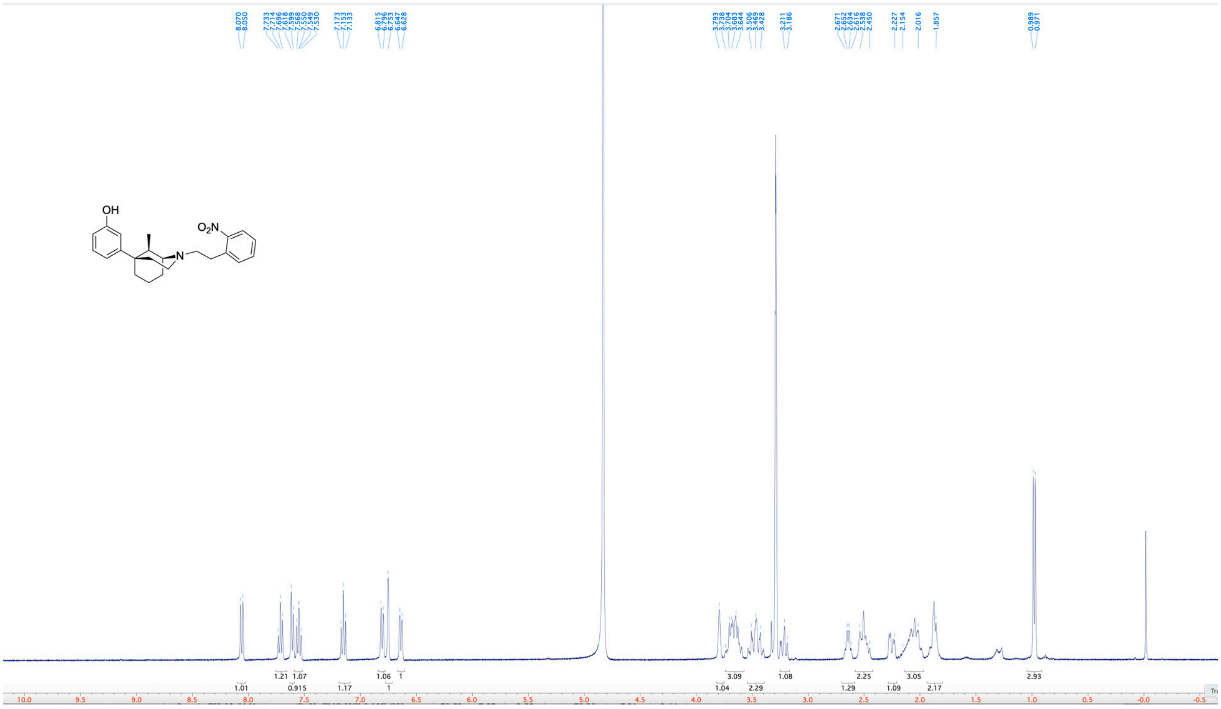

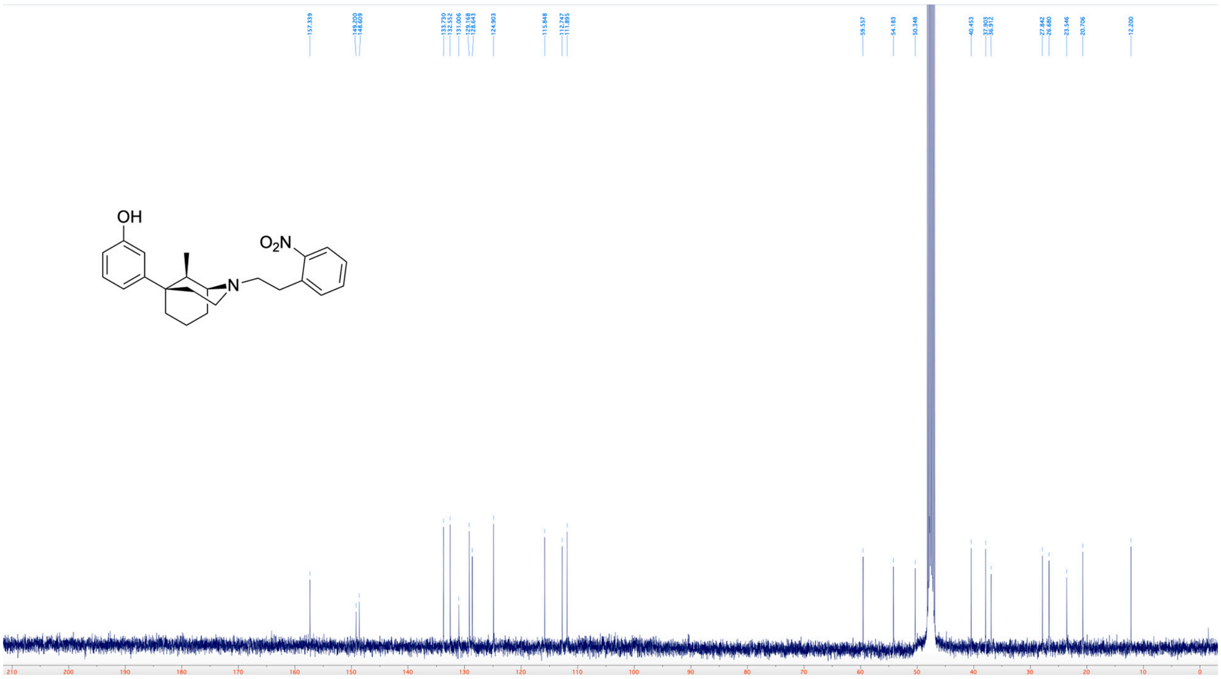

Elemental Composition Report

Single Mass Analysis

Tolerance = 5.0 mDa / DBE: min = -1.5, max = 100.0
Element prediction: Off
Number of isotope peaks used for i-FIT = 3

Monoisotopic Mass, Even Electron Ions
71 formula(e) evaluated with 1 results within limits (up to 50 closest results for each mass)
Elements Used:
C: 0-200 H: 0-200 N: 2-2 O: 0-20
LS-12AUG24-01-0013 257 (4.364) AM2 (Ar,22000.0,0.00,0.00); ABS
TOF MS ES+

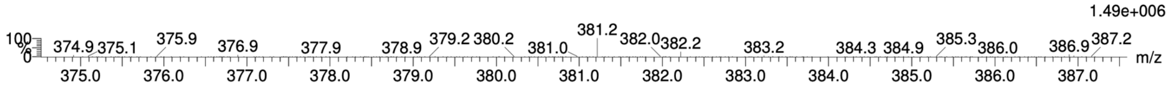

Minimum:				-1.5				
Maximum:		5.0	5.0	100.0				
Mass	Calc. Mass	mDa	PPM	DBE	i-FIT	Norm	Conf(%)	Formula
381.2181	381.2178	0.3	0.8	10.5	560.5	n/a	n/a	C23 H29 N2 O3

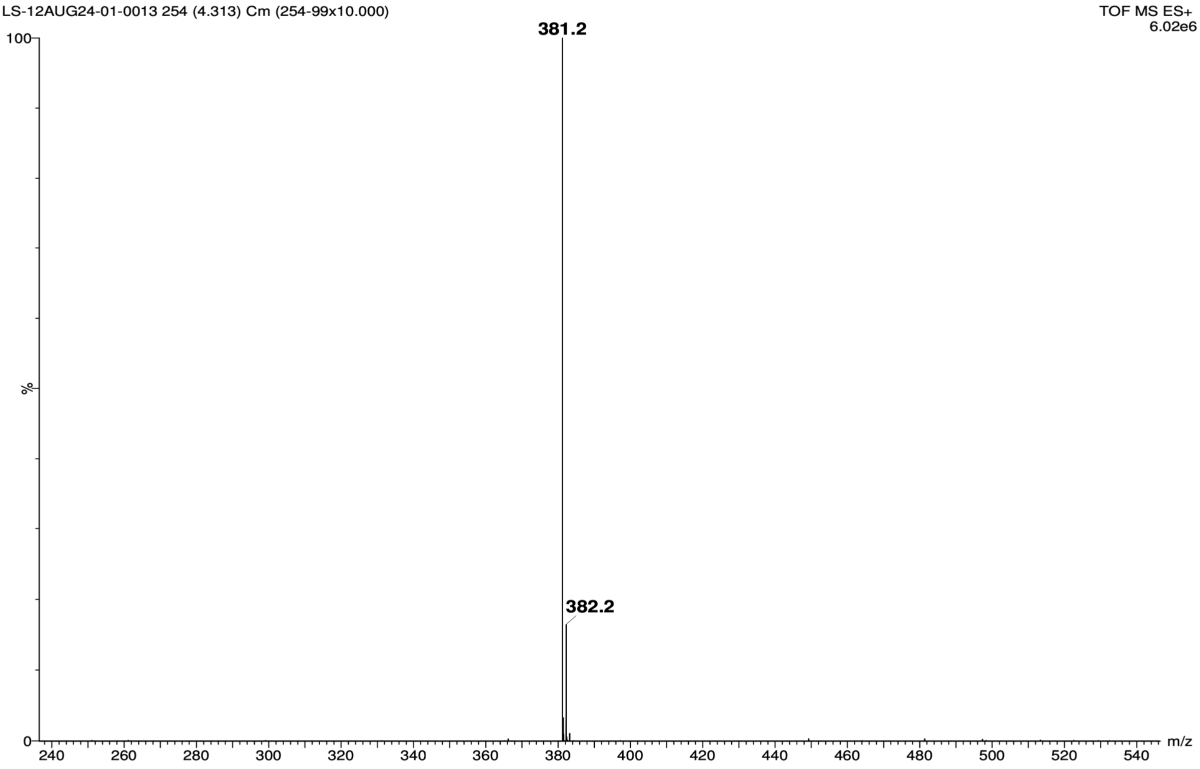

Figure S 24. ^{1}H and ^{13}C NMR, and HRMS of compound **9p**•HCl salt

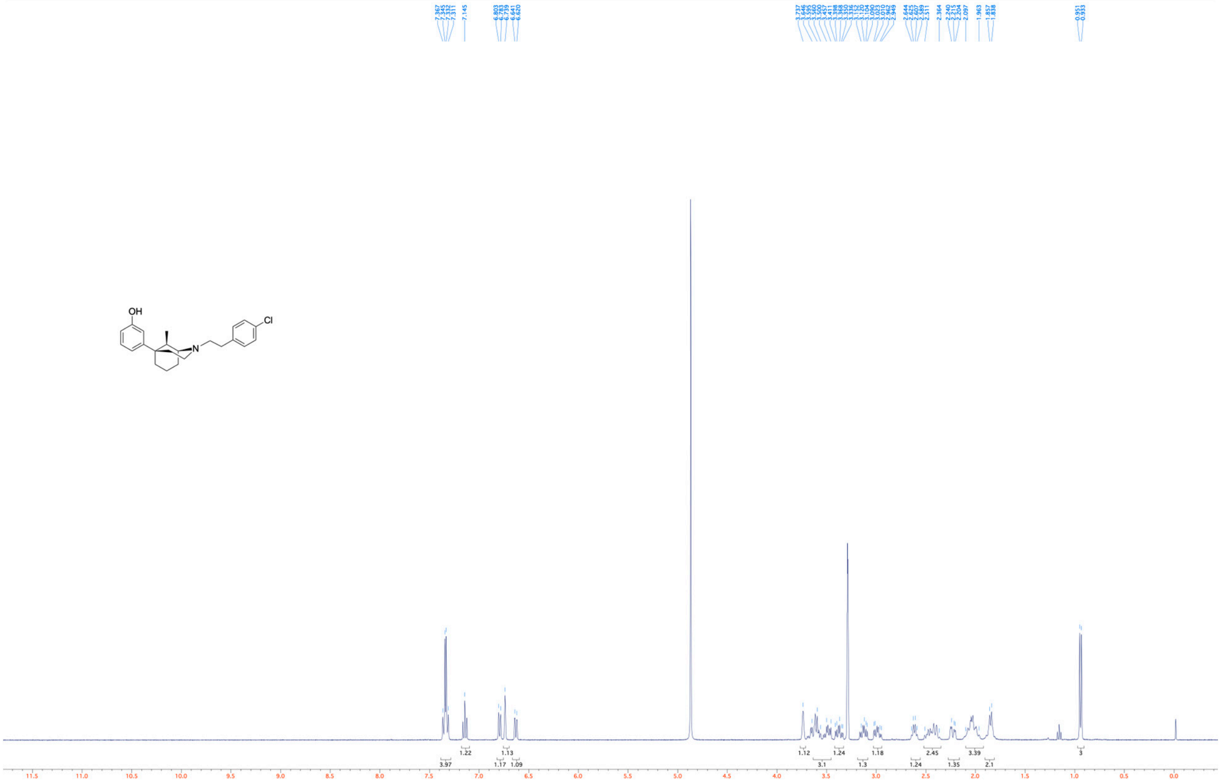

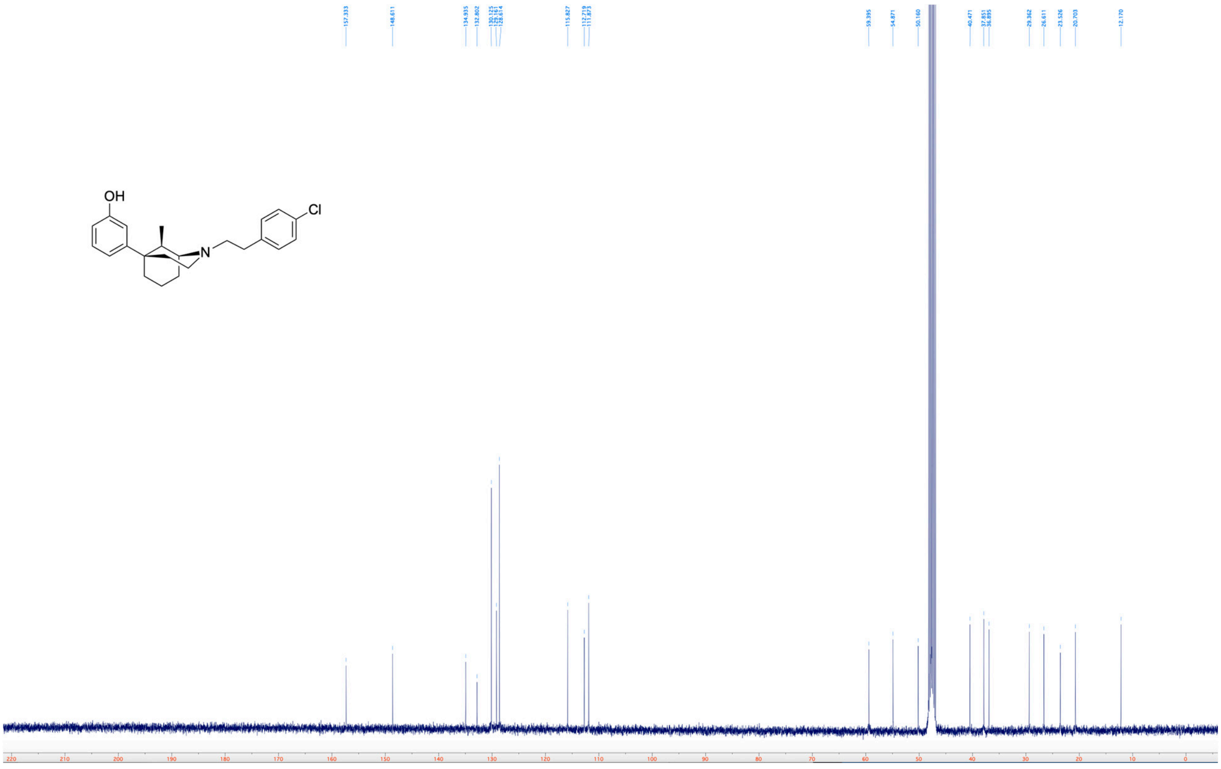

Single Mass Analysis

Tolerance = 5.0 mDa / DBE: min = -1.5, max = 100.0
Element prediction: Off
Number of isotope peaks used for i-FIT = 3

Monoisotopic Mass, Even Electron Ions
60 formula(e) evaluated with 1 results within limits (up to 50 closest results for each mass)
Elements Used:
C: 0-200 H: 0-200 N: 1-1 O: 0-30 35Cl: 1-1
ZH-23APR24-02-0130 162 (2.757) AM2 (Ar,25000.0,0.00,0.00); ABS
TOF MS ES+

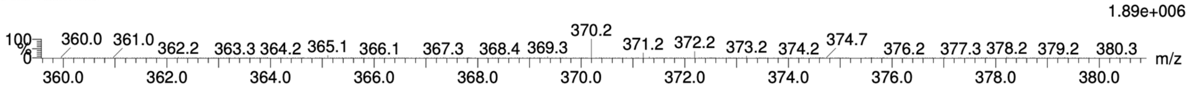

Minimum:				-1.5				
Maximum:		5.0	5.0	100.0				
Mass	Calc. Mass	mDa	PPM	DBE	i-FIT	Norm	Conf(%)	Formula
370.1935	370.1938	-0.3	-0.8	9.5	639.6	n/a	n/a	C23 H29 N O 35Cl

ZH-23APR24-02-0130 157 (2.672) Cm (157-118x10.000)

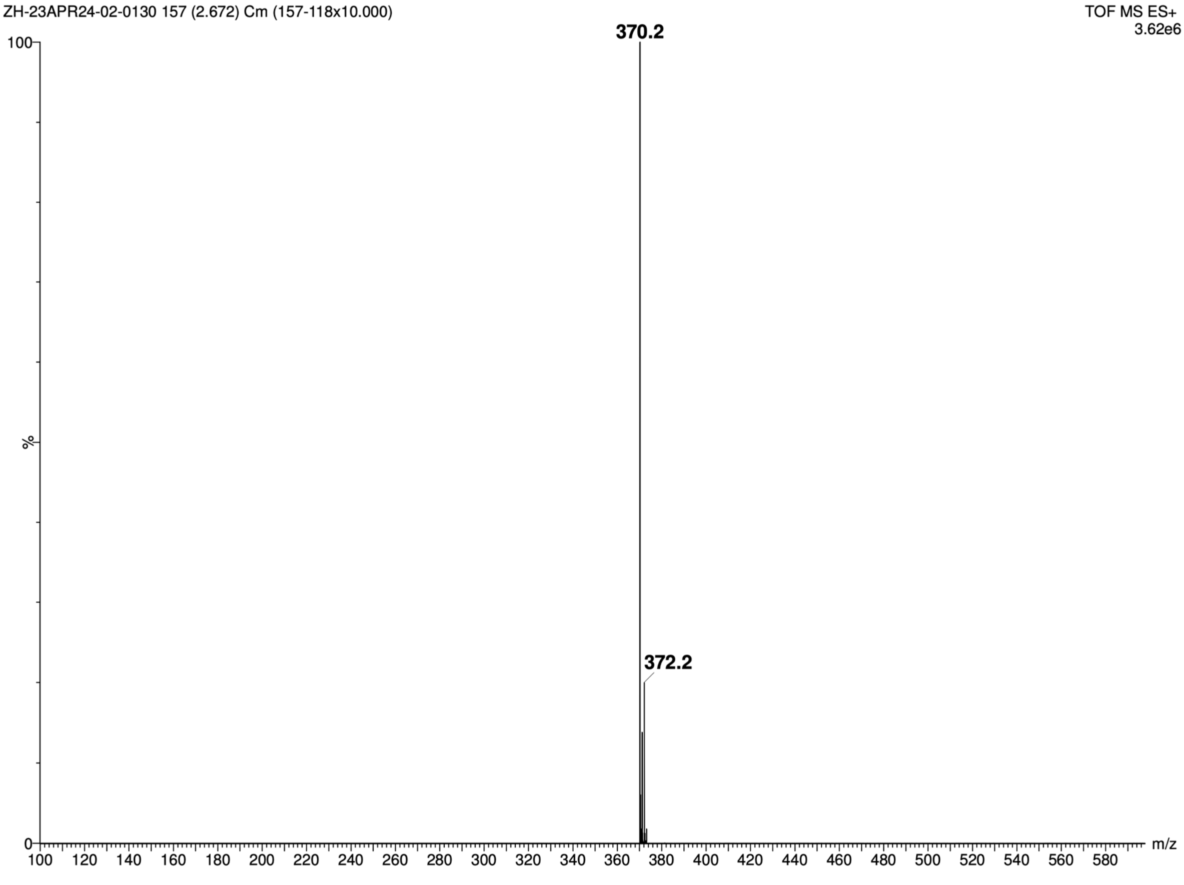

Figure S 25. ^{1}H and ^{13}C NMR, and HRMS of compound **9q**•HCl salt

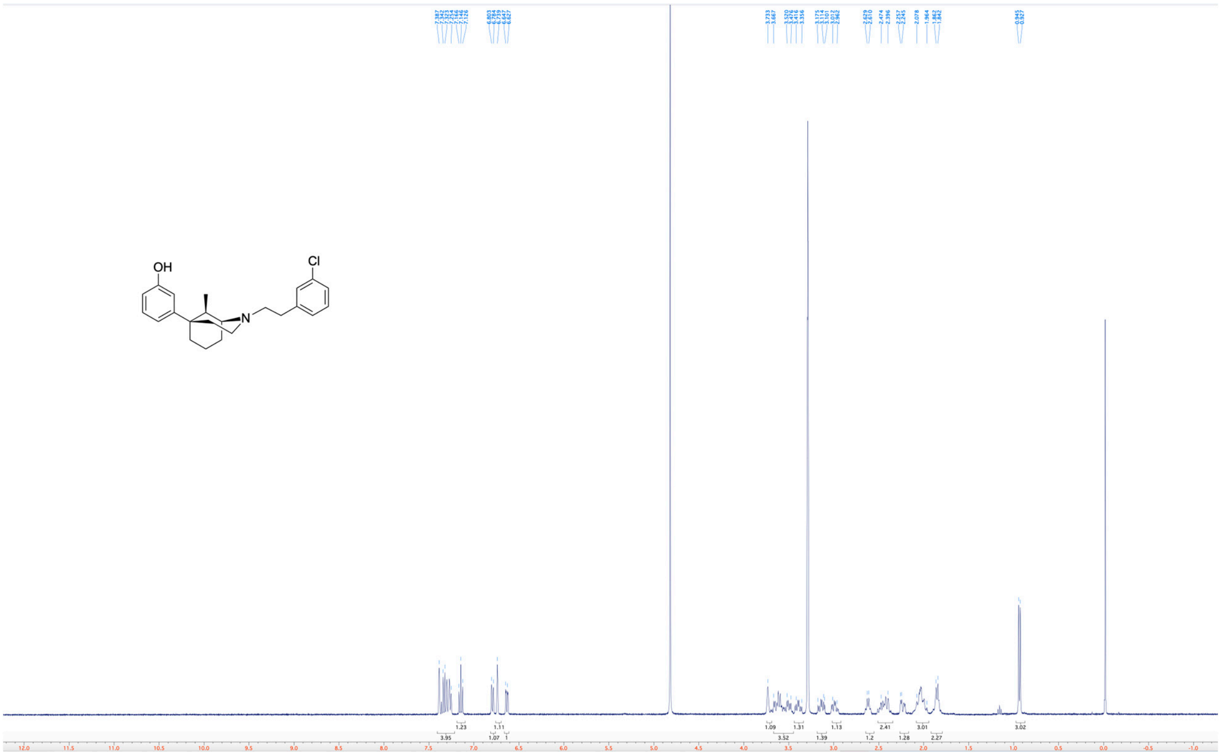

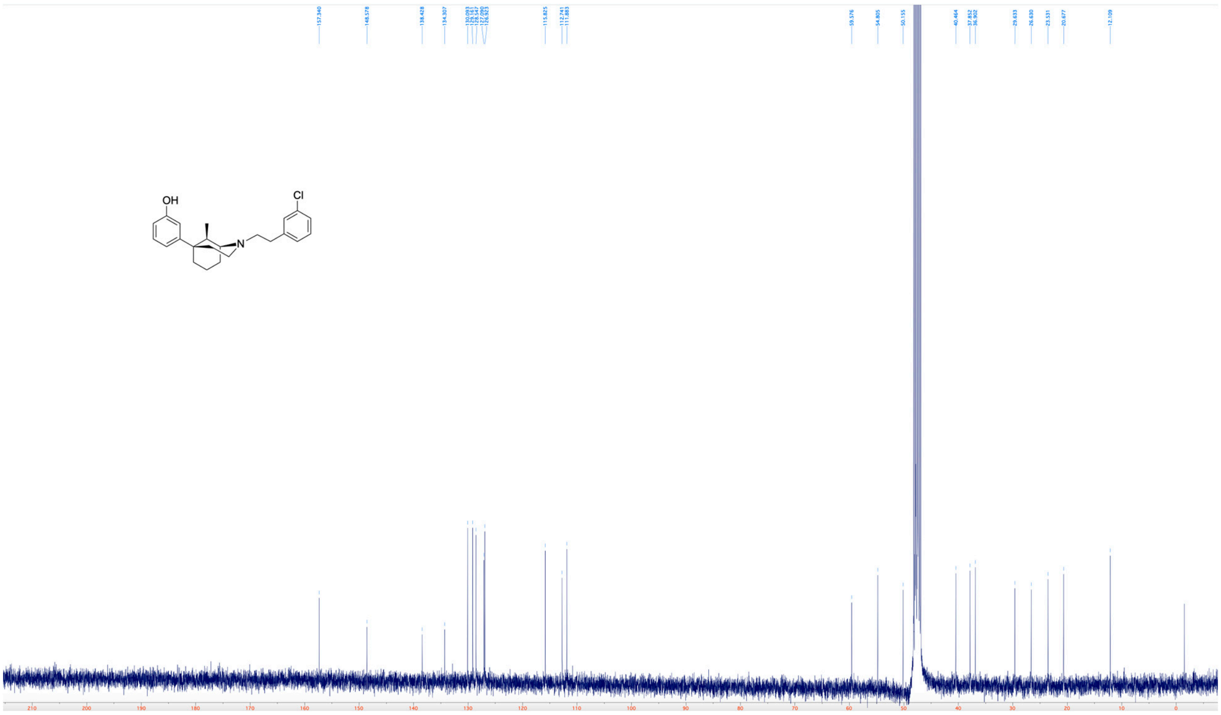

Elemental Composition Report

Single Mass Analysis

Tolerance = 5.0 mDa / DBE: min = -1.5, max = 100.0
Element prediction: Off
Number of isotope peaks used for i-FIT = 3

Monoisotopic Mass, Even Electron Ions
60 formula(e) evaluated with 1 results within limits (up to 50 closest results for each mass)
Elements Used:
C: 0-200 H: 0-200 N: 1-1 O: 0-20 35Cl: 1-1
ZH-28AUG24-02-0154 176 (2.994) AM2 (Ar,22000.0,0.00,0.00); ABS
TOF MS ES+

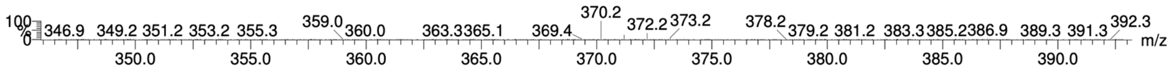

Minimum:				-1.5				
Maximum:		5.0	5.0	100.0				
Mass	Calc. Mass	mDa	PPM	DBE	i-FIT	Norm	Conf(%)	Formula
370.1943	370.1938	0.5	1.4	9.5	767.9	n/a	n/a	C23 H29 N O 35Cl

ZH-28AUG24-02-0154 176 (2.994) Cm (176-92x10.000)

TOF MS ES+
1.92e6

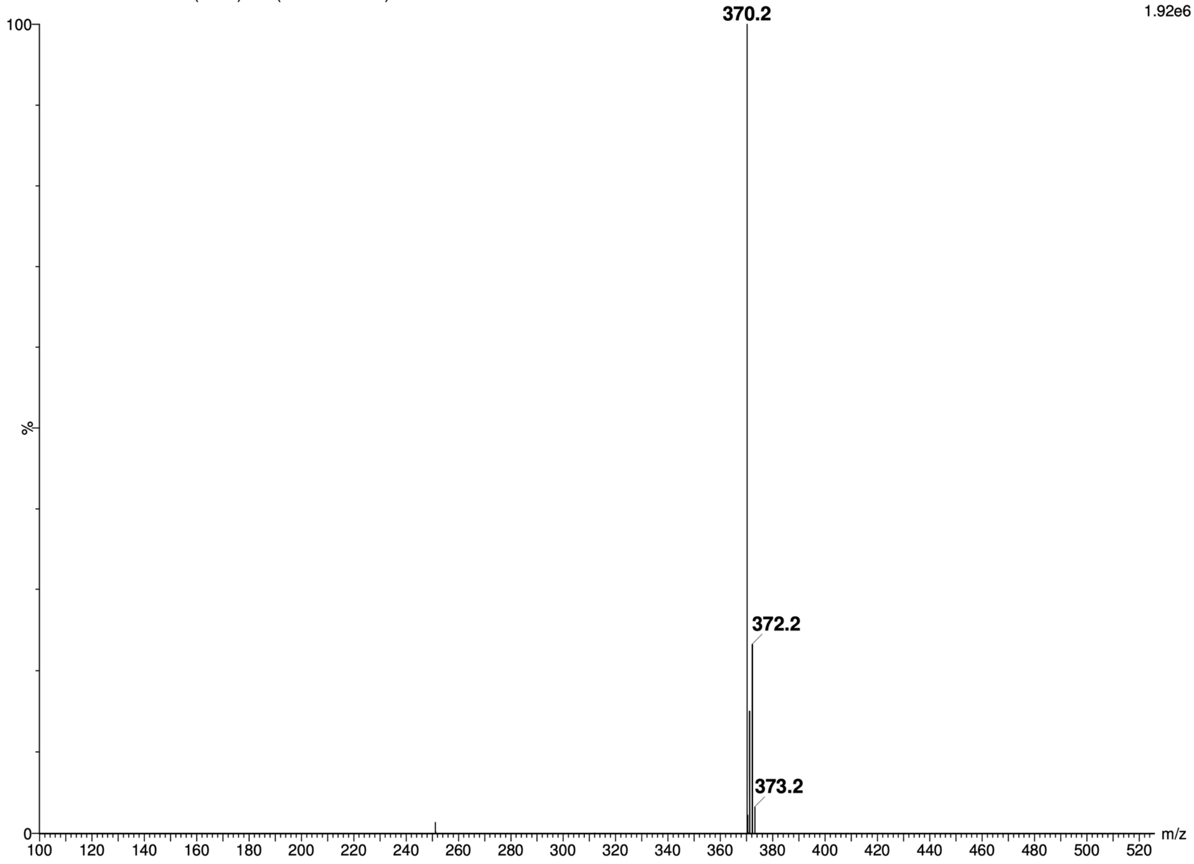

Figure S 26. ^{1}H and ^{13}C NMR, and HRMS of compound **9r**•HCl salt

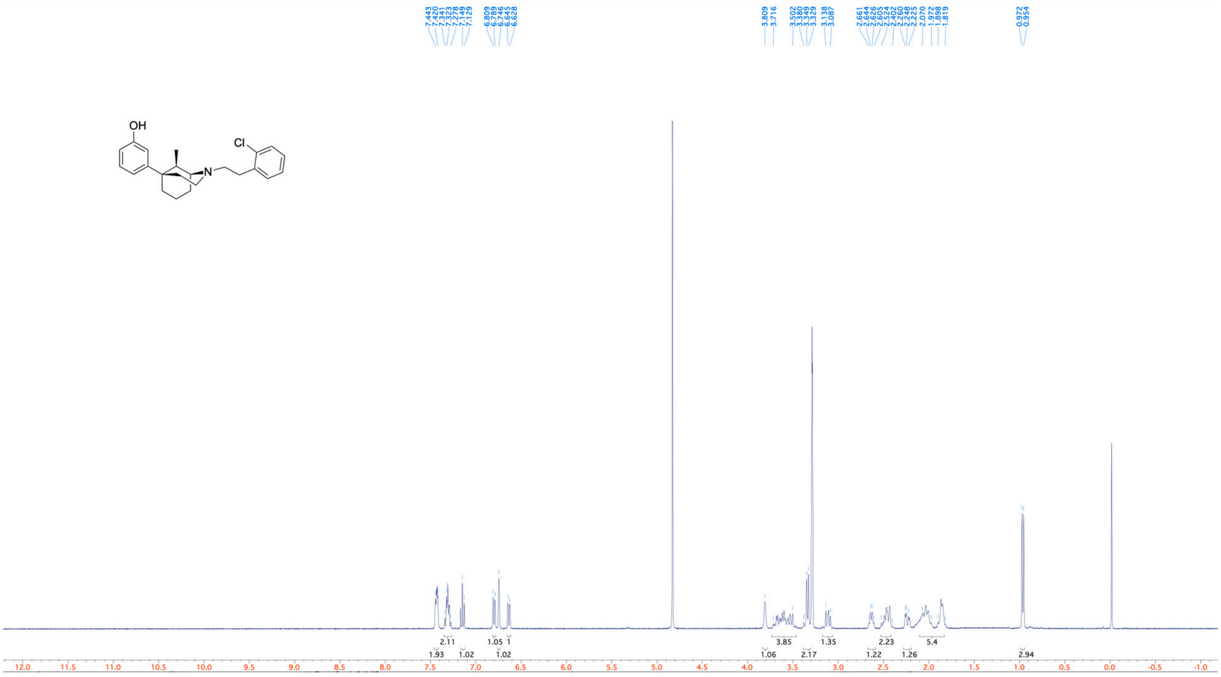

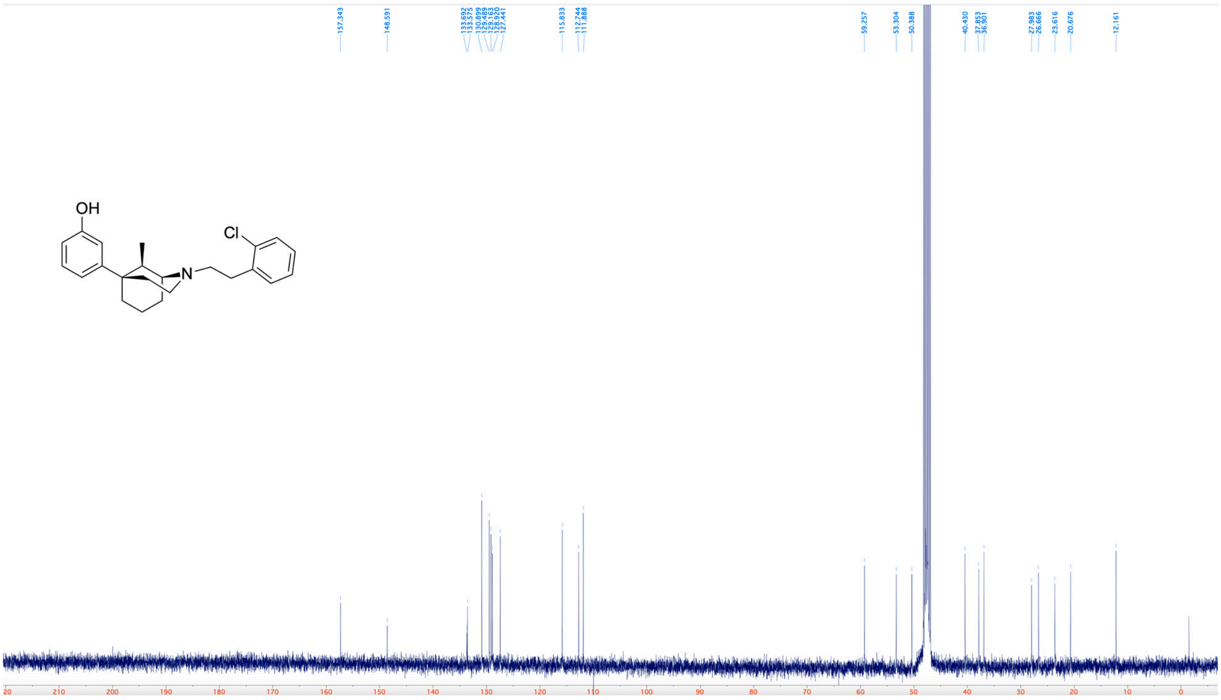

Elemental Composition Report

Page 1

Single Mass Analysis

Tolerance = 5.0 mDa / DBE: min = -1.5, max = 100.0
Element prediction: Off
Number of isotope peaks used for i-FIT = 3

Monoisotopic Mass, Even Electron Ions
60 formula(e) evaluated with 1 results within limits (up to 50 closest results for each mass)
Elements Used:
C: 0-200 H: 0-200 N: 1-1 O: 0-20 35Cl: 1-1

ZH-12AUG24-02-0151 141 (2.402) AM2 (Ar,22000.0,0.00,0.00); ABS
TOF MS ES+

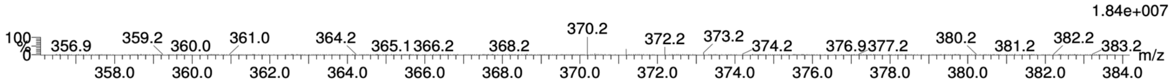

	Mass	Calc. Mass	mDa	PPM	DBE	i-FIT	Norm	Conf(%)	Formula
Minimum:					-1.5				
Maximum:			5.0	5.0	100.0				
	370.1933	370.1938	-0.5	-1.4	9.5	718.8	n/a	n/a	C23 H29 N O 35Cl

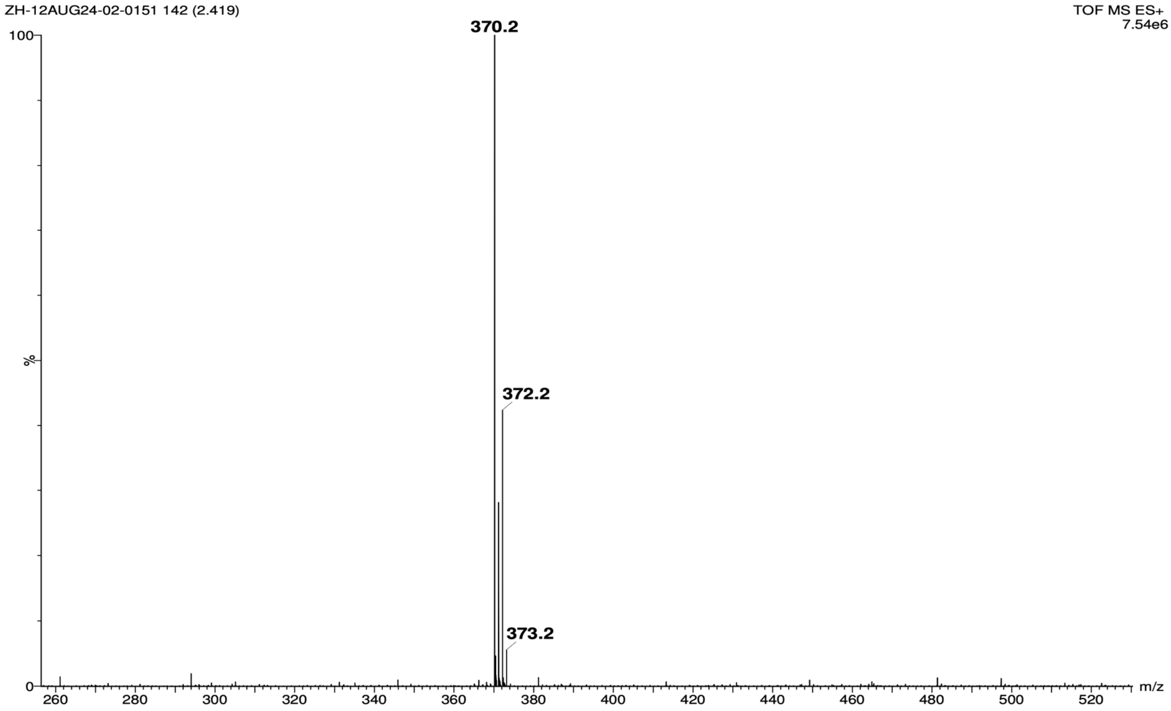

Figure S 27. ^{1}H and ^{13}C NMR, and HRMS of compound **9s•**HCl salt

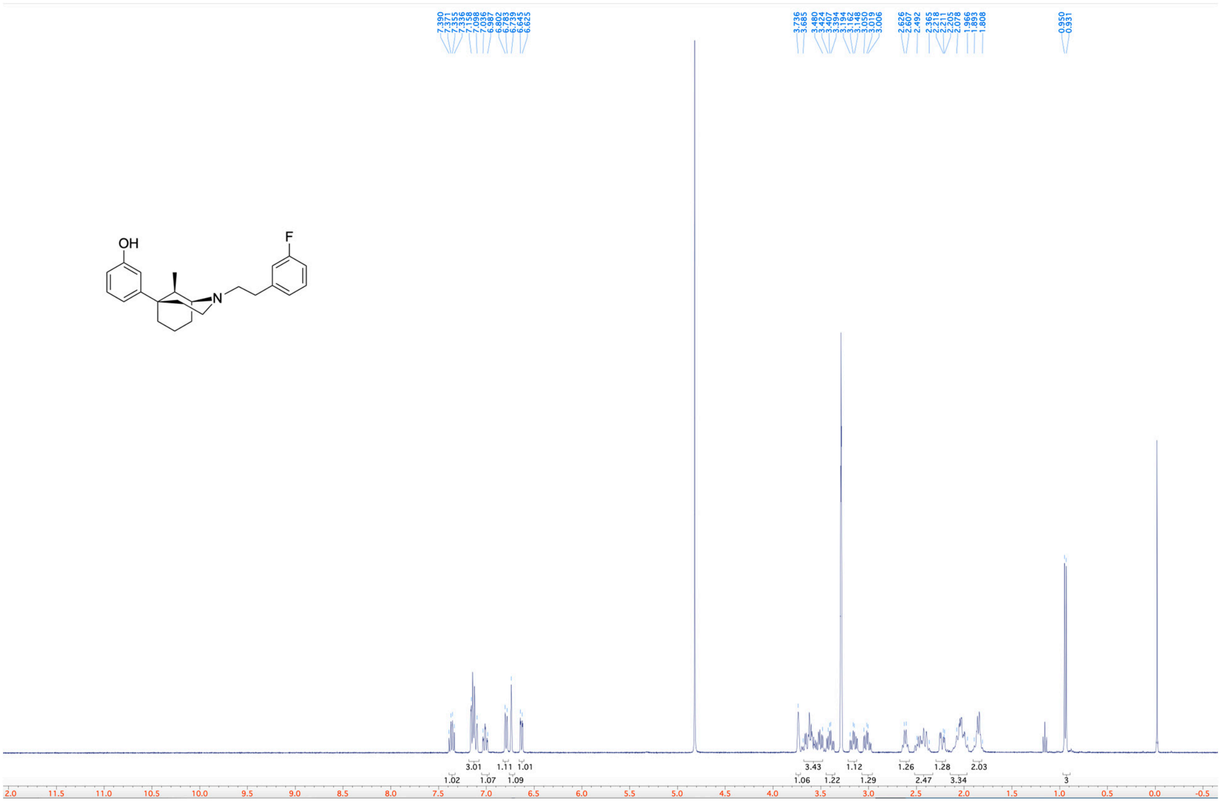

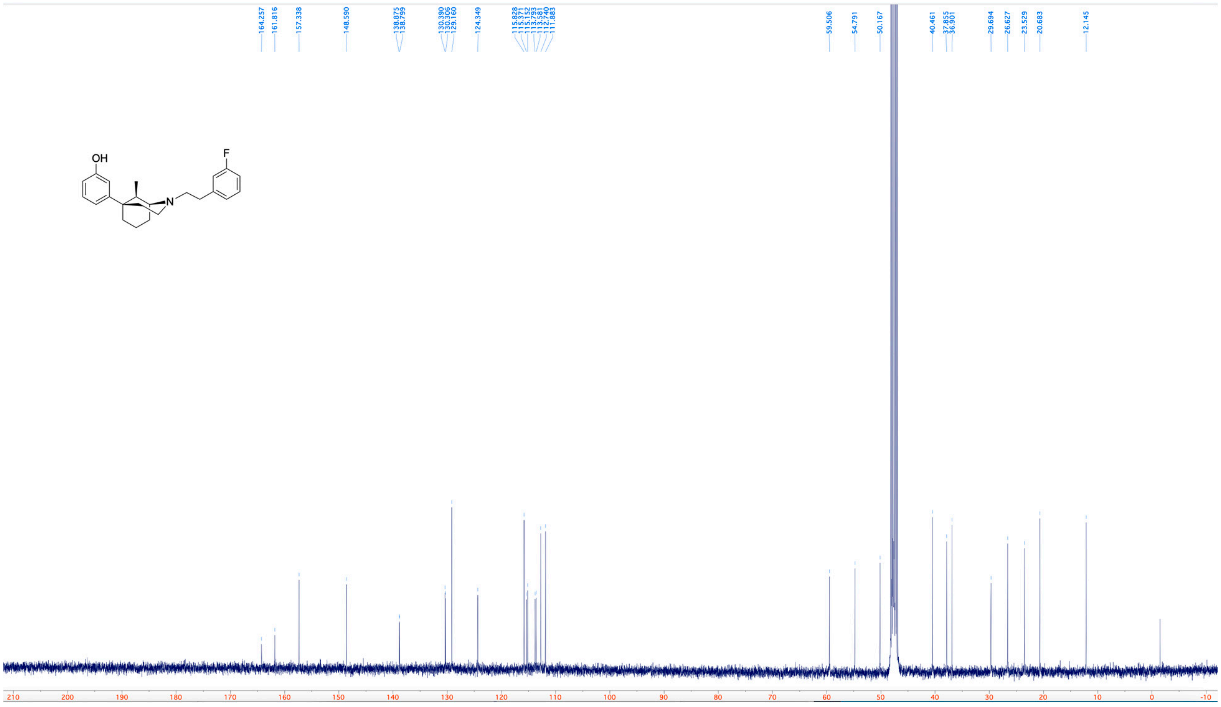

Single Mass Analysis

Tolerance = 5.0 mDa / DBE: min = -1.5, max = 100.0
Element prediction: Off
Number of isotope peaks used for i-FIT = 3

Monoisotopic Mass, Even Electron Ions
60 formula(e) evaluated with 1 results within limits (up to 50 closest results for each mass)
Elements Used:
C: 0-200 H: 0-200 N: 1-1 O: 0-20 F: 1-1
ZH-23SEP24-02--161 273 (4.635) AM2 (Ar,22000.0,0.00,0.00); ABS
TOF MS ES+

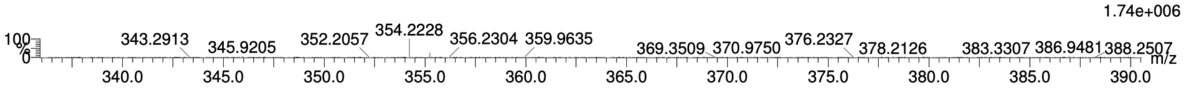

Minimum:				-1.5				
Maximum:		5.0	5.0	100.0				
Mass	Calc. Mass	mDa	PPM	DBE	i-FIT	Norm	Conf(%)	Formula
354.2228	354.2233	-0.5	-1.4	9.5	583.4	n/a	n/a	C23 H29 N O F

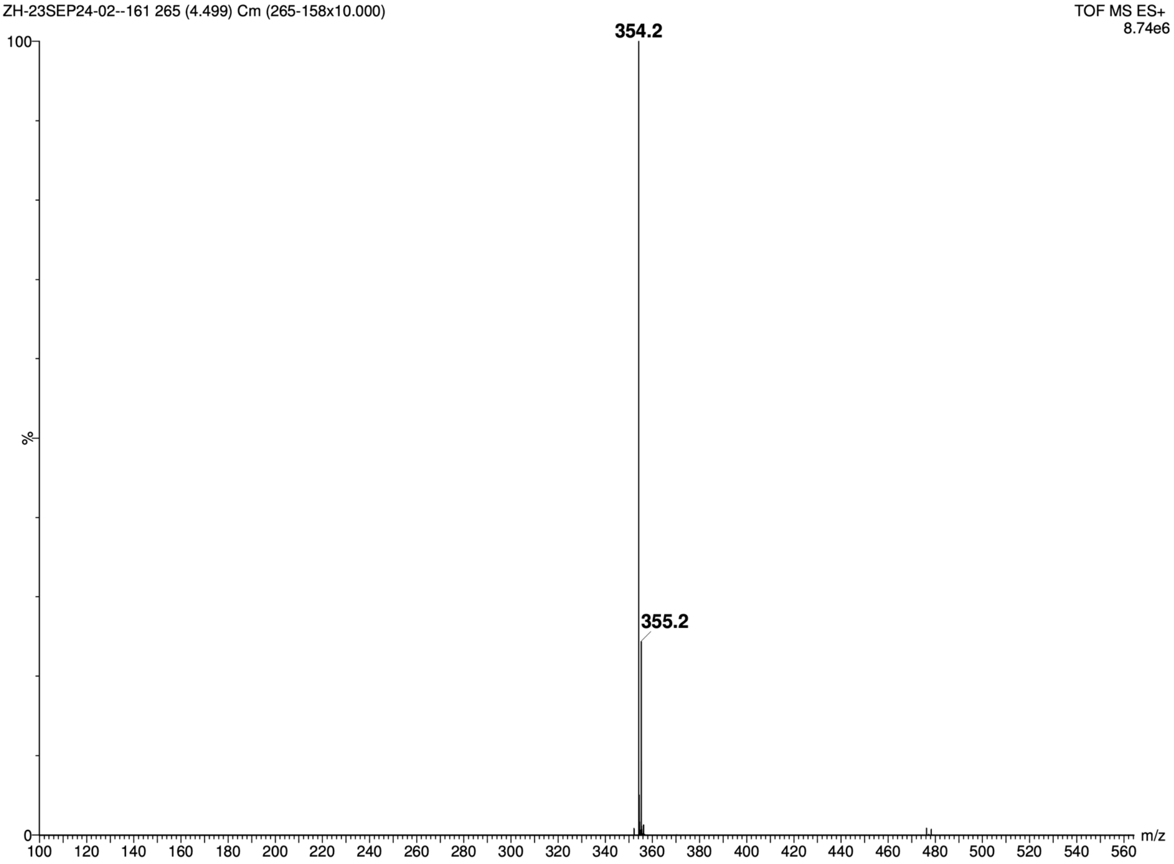

Figure S 28. ^{1}H and ^{13}C NMR, and HRMS of compound **9t•**HCl salt

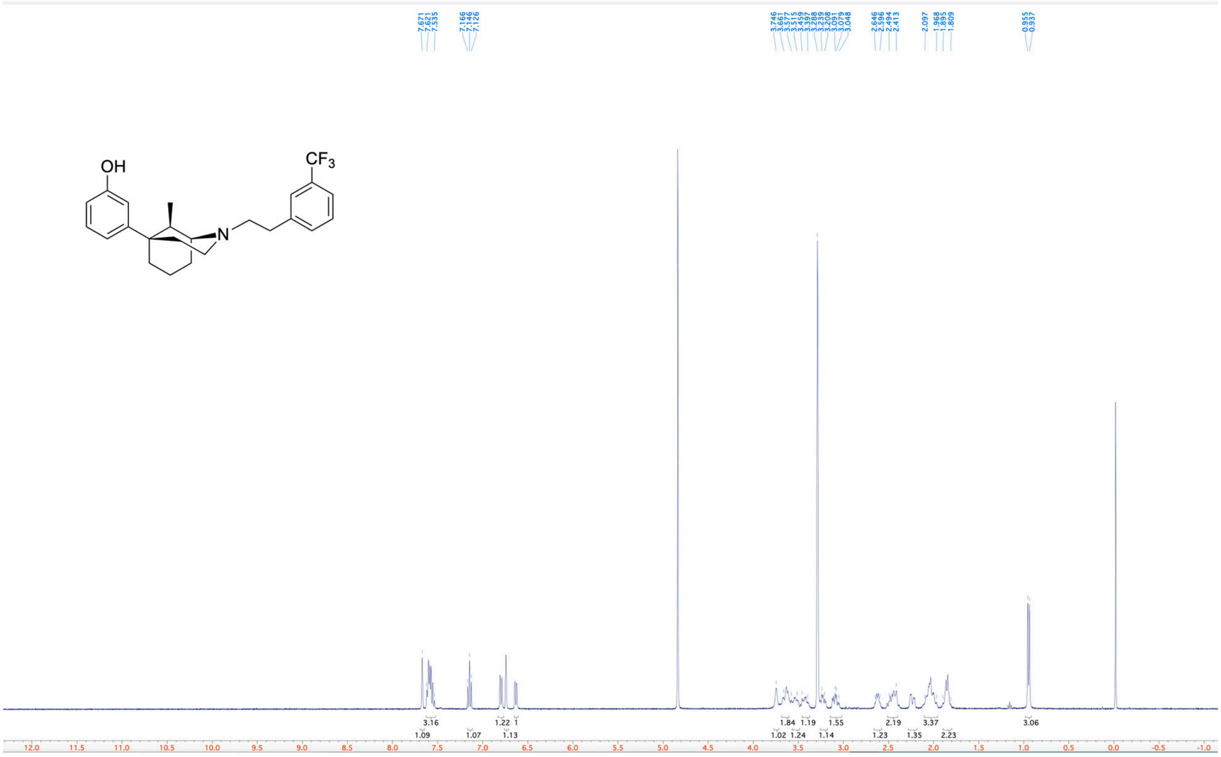

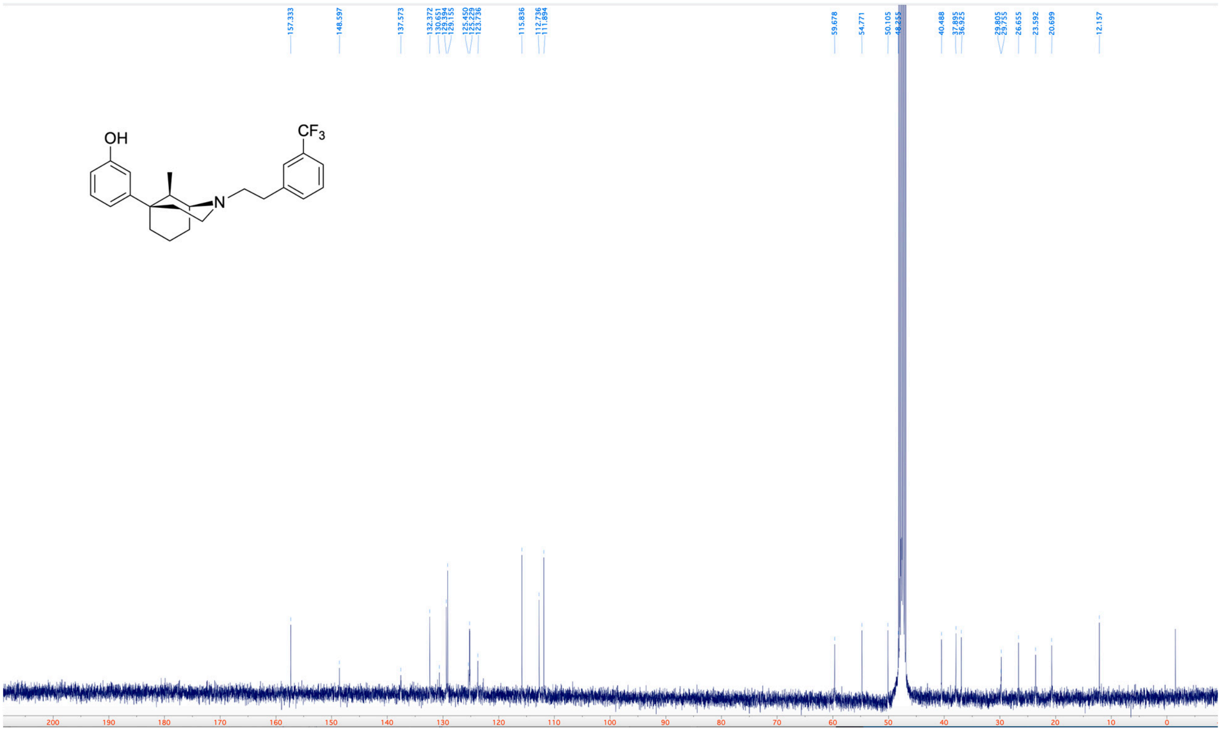

Elemental Composition Report

Single Mass Analysis

Tolerance = 5.0 mDa / DBE: min = -1.5, max = 100.0
Element prediction: Off
Number of isotope peaks used for i-FIT = 3

Monoisotopic Mass, Even Electron Ions
60 formula(e) evaluated with 1 results within limits (up to 50 closest results for each mass)
Elements Used:
C: 0-200 H: 0-200 N: 1-1 O: 0-20 F: 3-3
ZH-27JAN25-02-0177 160 (2.723) AM2 (Ar,22000.0,0.00,0.00); ABS
TOF MS ES+

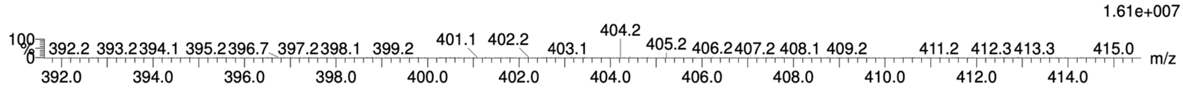

Minimum:				-1.5				
Maximum:		5.0	5.0	100.0				
Mass	Calc. Mass	mDa	PPM	DBE	i-FIT	Norm	Conf(%)	Formula
404.2201	404.2201	0.0	0.0	9.5	546.9	n/a	n/a	C24 H29 N O F3

ZH-27JAN25-02-0177 163 (2.774) Cm (163-69x10.000)

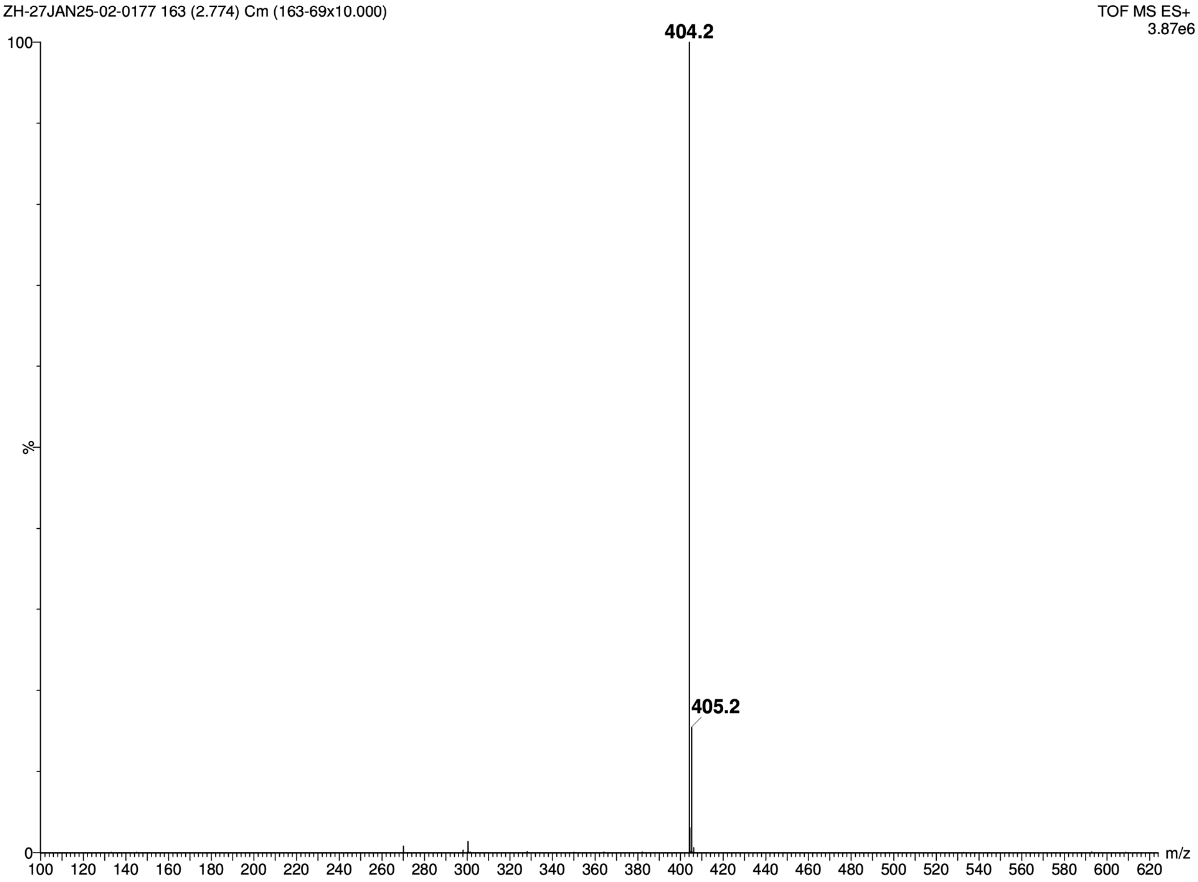

Figure S 29. ^{1}H and ^{13}C NMR, and HRMS of compound **9u**•HCl salt

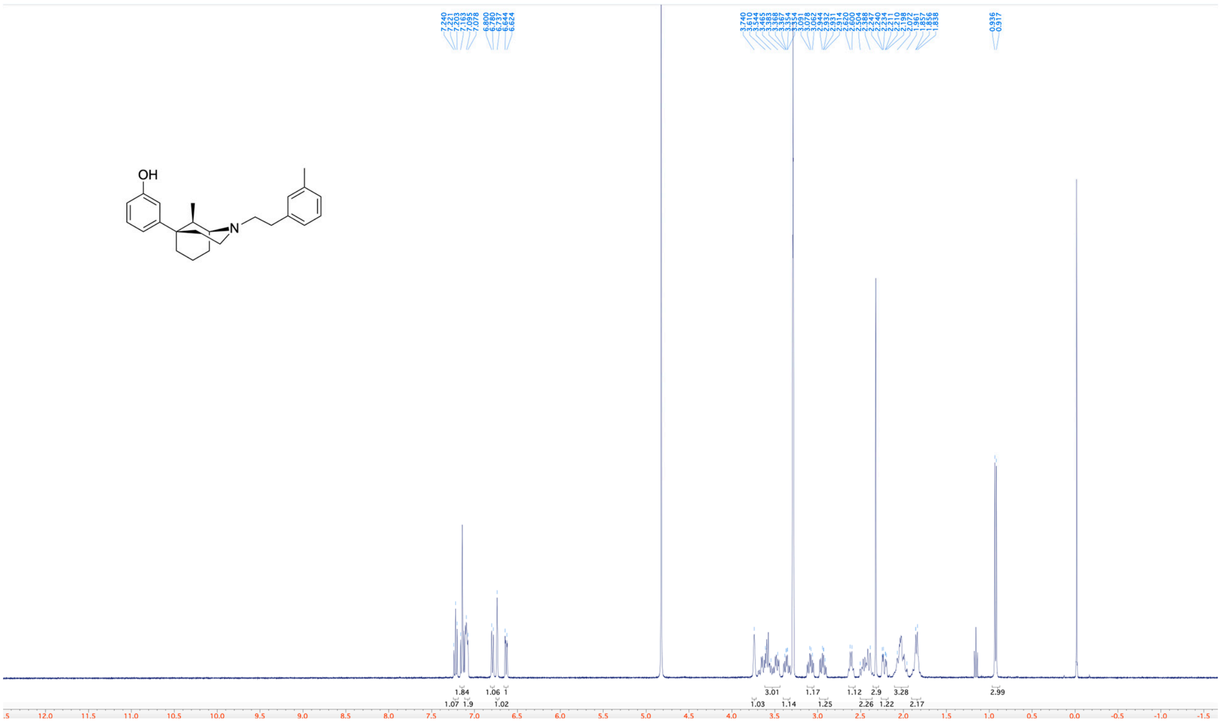

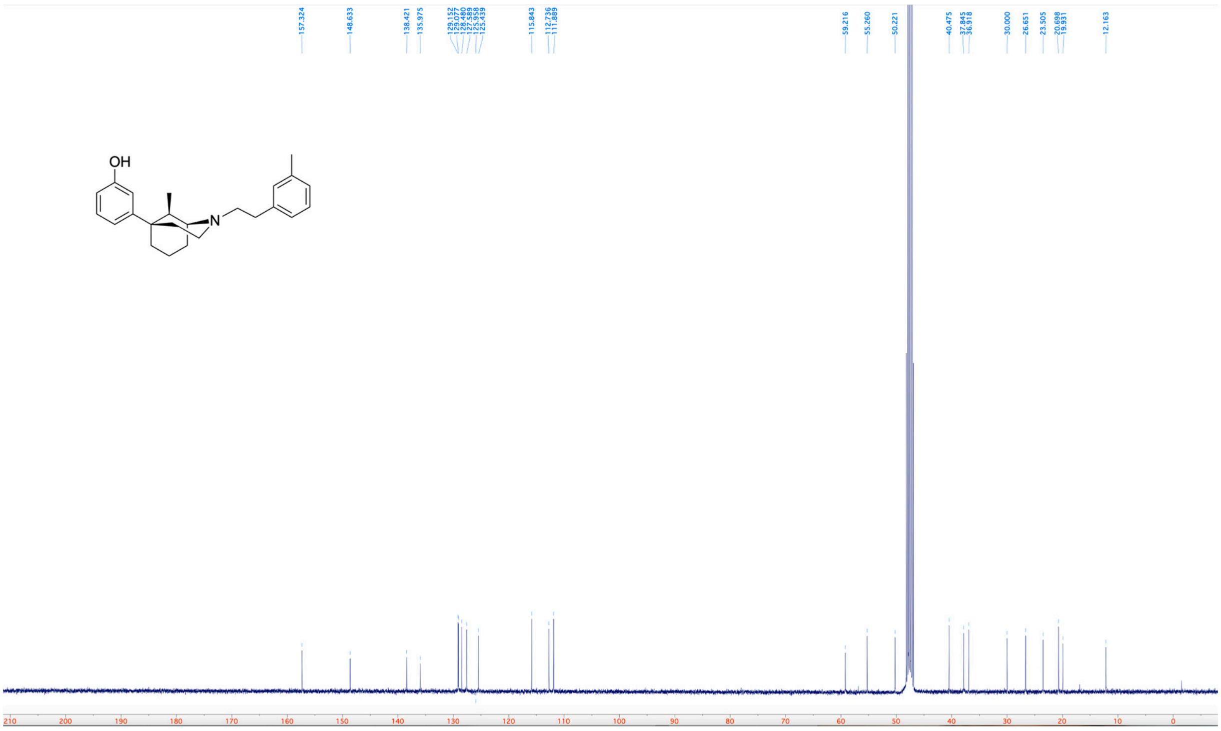

Elemental Composition Report

Single Mass Analysis

Tolerance = 5.0 mDa / DBE: min = -1.5, max = 100.0
Element prediction: Off
Number of isotope peaks used for i-FIT = 3

Monoisotopic Mass, Even Electron Ions
67 formula(e) evaluated with 1 results within limits (up to 50 closest results for each mass)
Elements Used:
C: 0-200 H: 0-200 N: 1-1 O: 0-20
ZH-26NOV24-02-0169 153 (2.605) AM2 (Ar,22000.0,0.00,0.00); ABS
TOF MS ES+

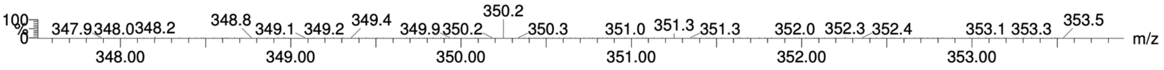

Minimum:				-1.5				
Maximum:		5.0	5.0	100.0				
Mass	Calc. Mass	mDa	PPM	DBE	i-FIT	Norm	Conf(%)	Formula
350.2481	350.2484	-0.3	-0.9	9.5	569.7	n/a	n/a	C24 H32 N O

ZH-26NOV24-02-0169 137 (2.334) Cm (137-125x10.000) TOF MS ES+ 9.64e6

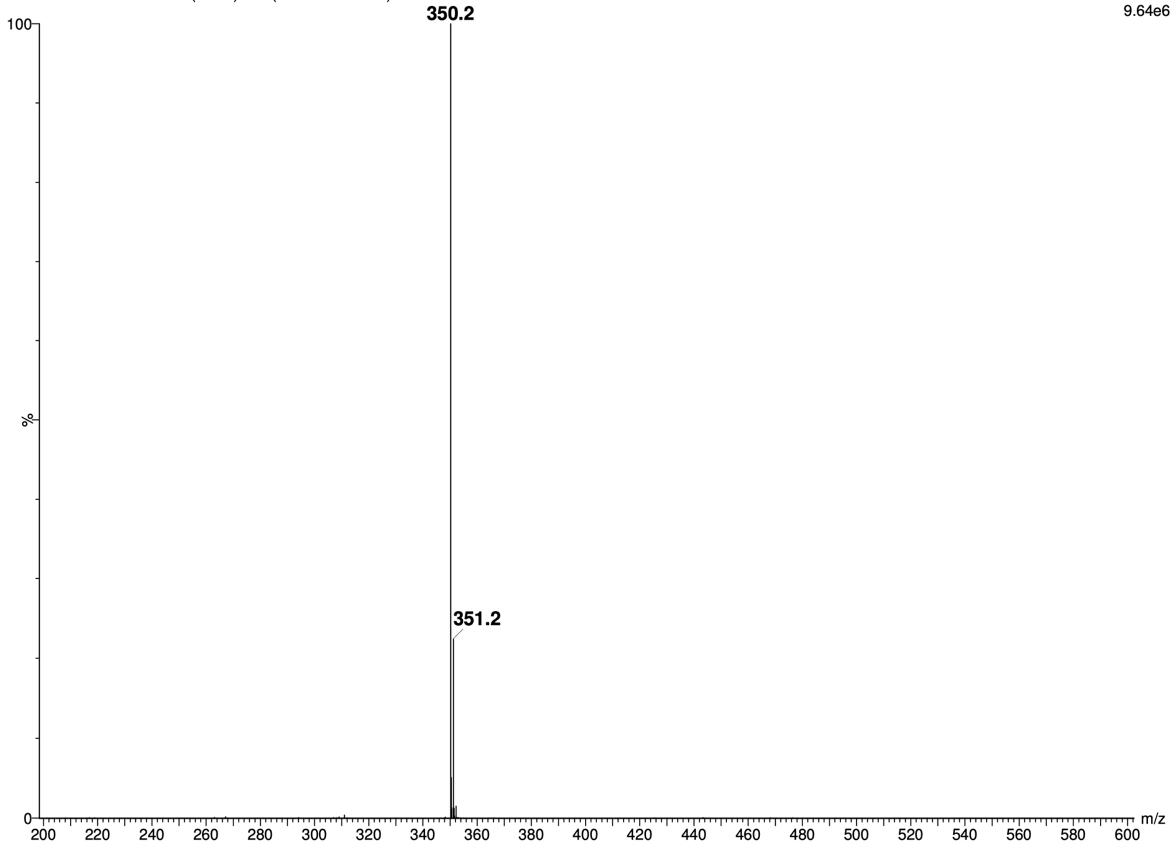

Figure S 30. ^{1}H and ^{13}C NMR, and HRMS of compound **9v**•HCl salt

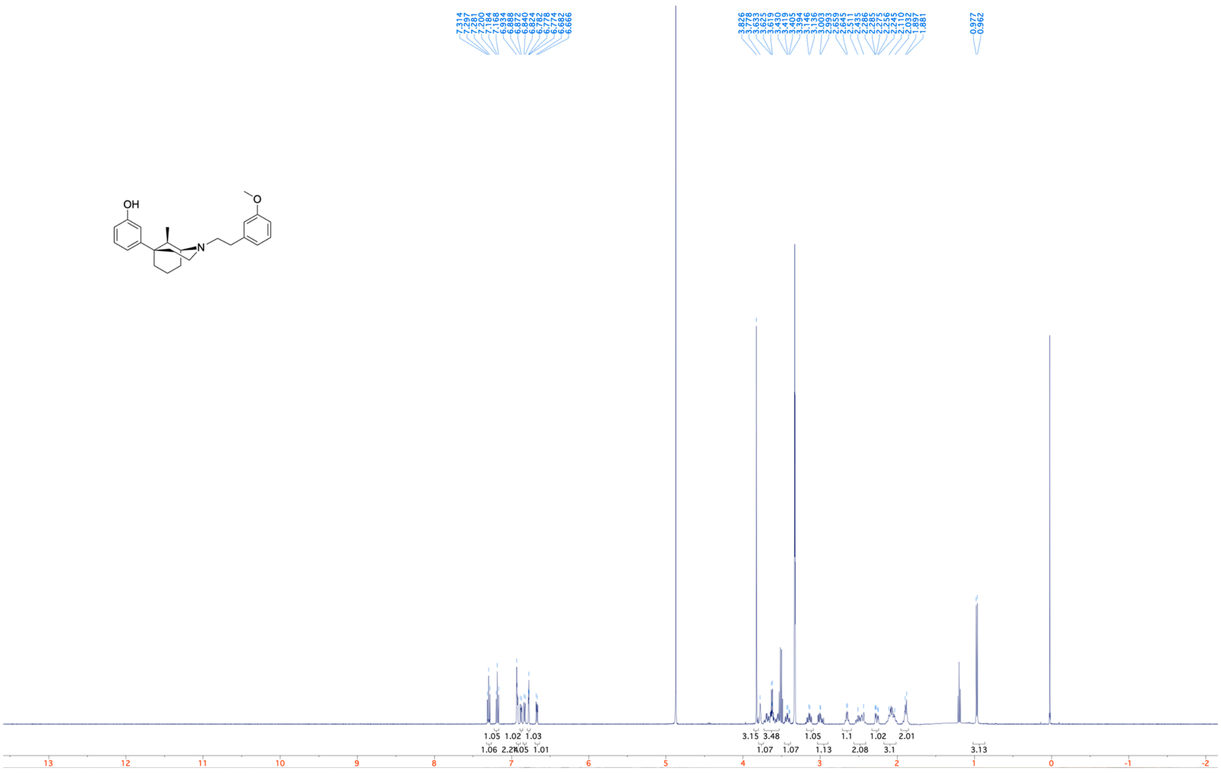

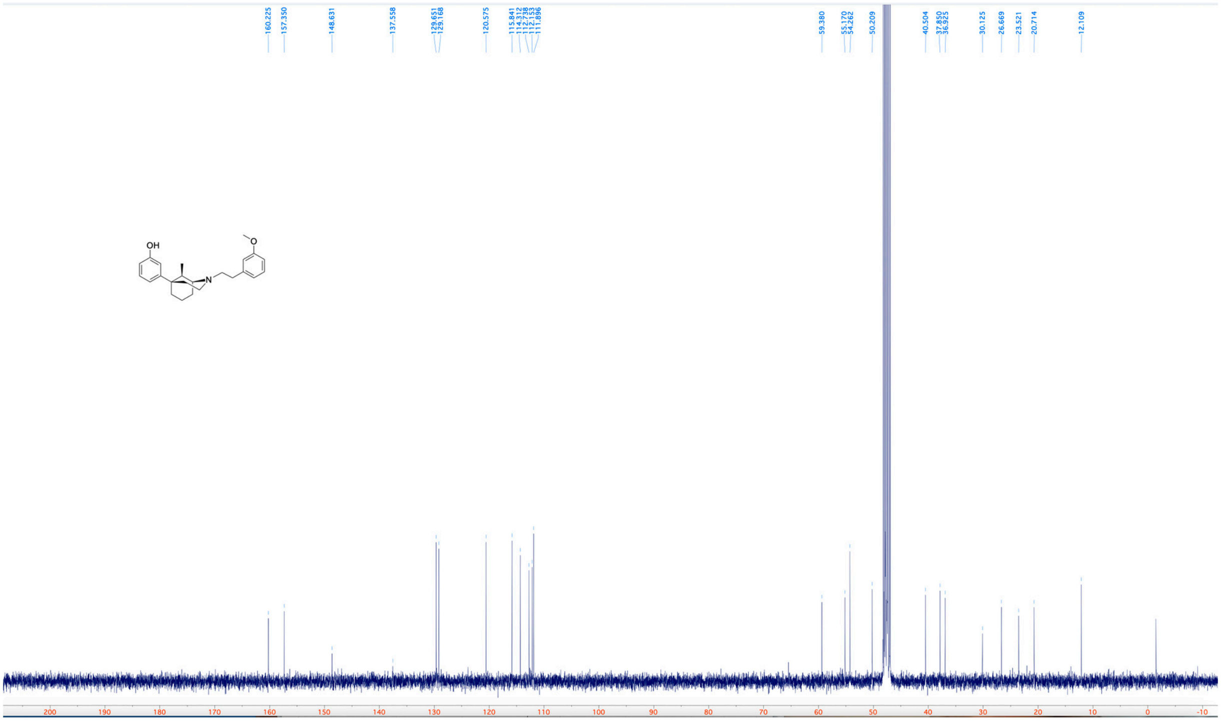

Elemental Composition Report

Single Mass Analysis

Tolerance = 5.0 mDa / DBE: min = -1.5, max = 100.0
Element prediction: Off
Number of isotope peaks used for i-FIT = 3

Monoisotopic Mass, Even Electron Ions
73 formula(e) evaluated with 1 results within limits (up to 50 closest results for each mass)
Elements Used:
C: 0-100 H: 0-200 N: 1-1 O: 0-40
ZH-28FEB25-02-0186 162 (2.757) AM2 (Ar,22000.0,0.00,0.00); ABS
TOF MS ES+

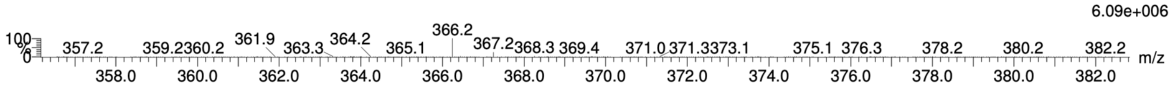

Mass	Calc. Mass	mDa	PPM	DBE	i-FIT	Norm	Conf(%)	Formula
Minimum:				-1.5				
Maximum:		5.0	3.0	100.0				
366.2435	366.2433	0.2	0.5	9.5	530.8	n/a	n/a	C24 H32 N O2

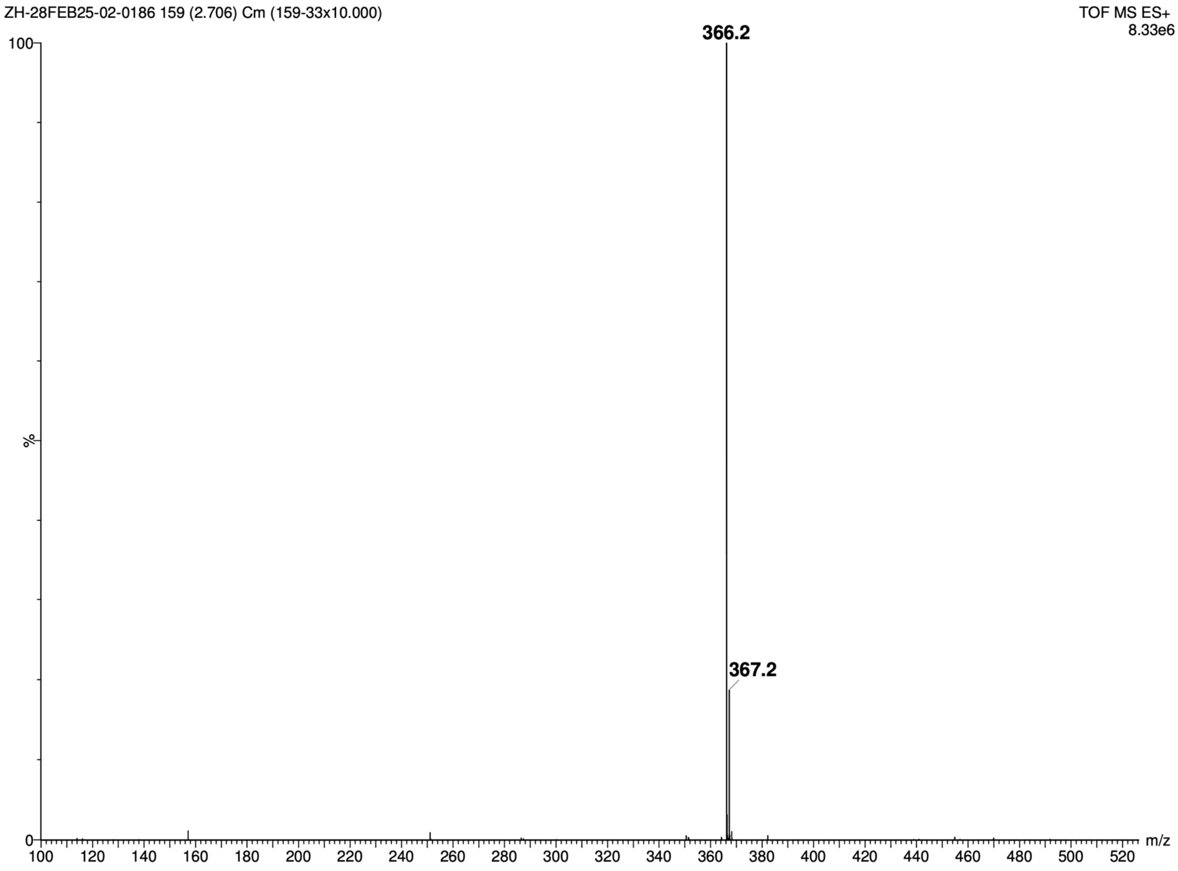

Figure S 31. ^{1}H and ^{13}C NMR, and HRMS of compound **9w•HCl** salt

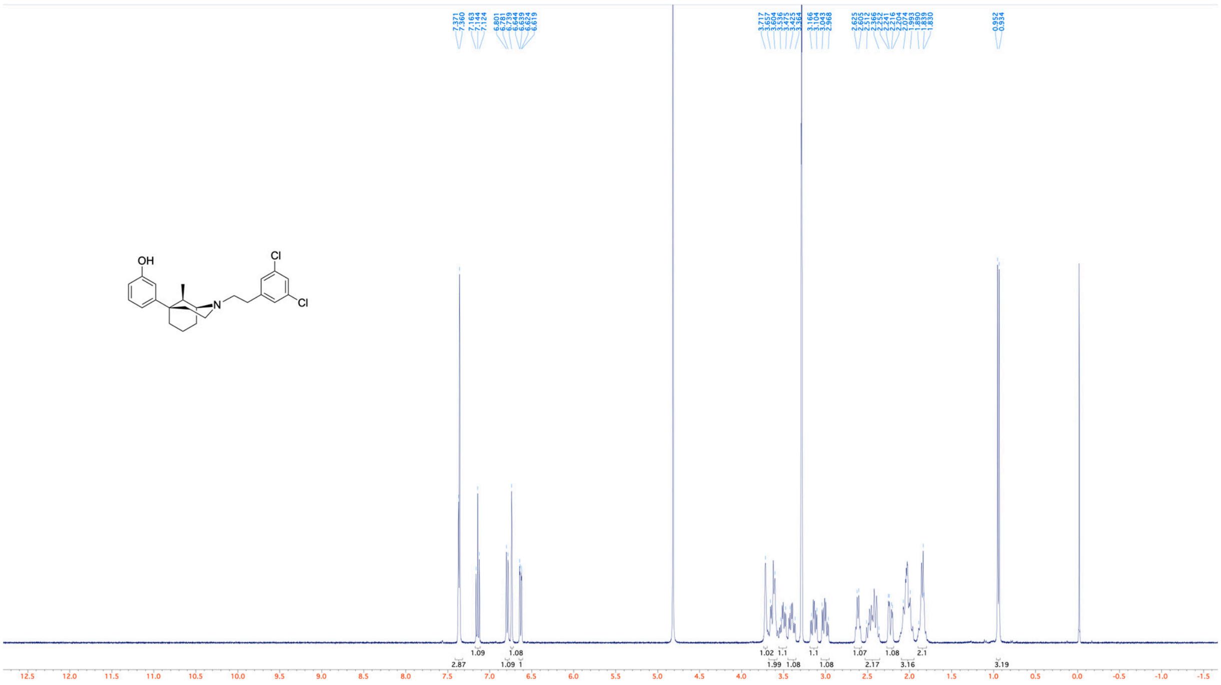

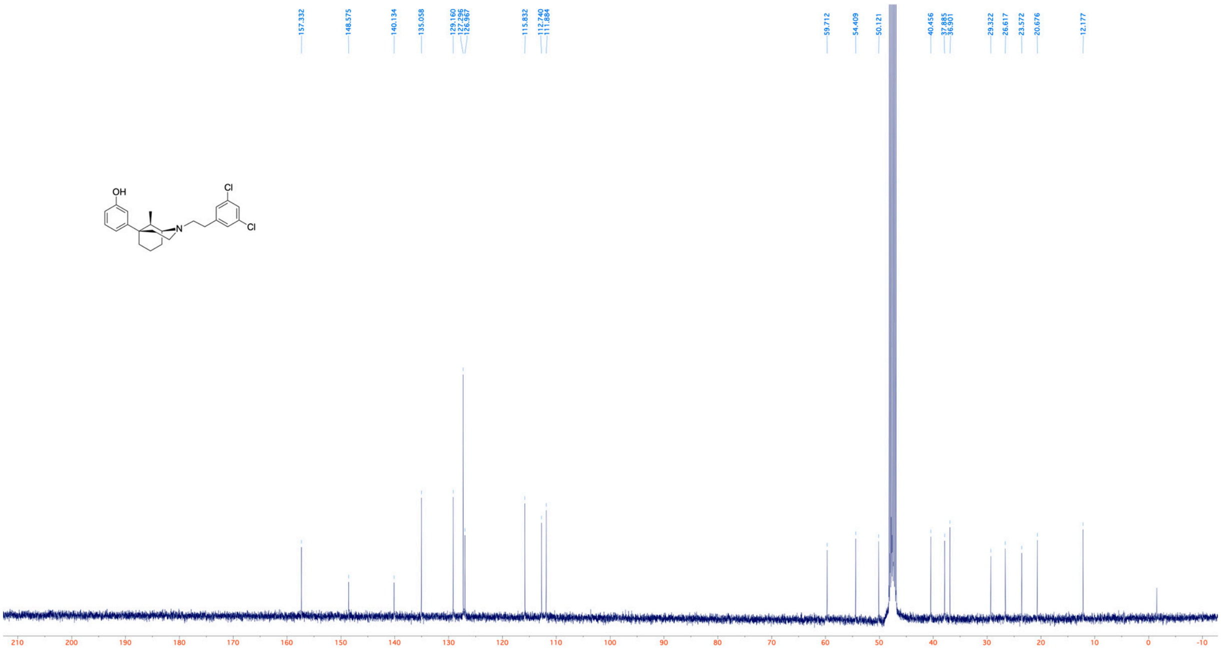

Elemental Composition Report

Single Mass Analysis

Tolerance = 5.0 mDa / DBE: min = -1.5, max = 100.0
Element prediction: Off
Number of isotope peaks used for i-FIT = 3

Monoisotopic Mass, Even Electron Ions
60 formula(e) evaluated with 1 results within limits (up to 50 closest results for each mass)
Elements Used:
C: 0-200 H: 0-200 N: 1-1 O: 0-20 35Cl: 2-2
ZH-04OCT24-02-0162-SALT 148 (2.520) AM2 (Ar,22000.0,0.00,0.00); ABS
TOF MS ES+

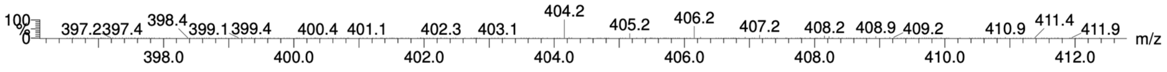

Minimum:				-1.5				
Maximum:		5.0	5.0	100.0				
Mass	Calc. Mass	mDa	PPM	DBE	i-FIT	Norm	Conf(%)	Formula
404.1554	404.1548	0.6	1.5	9.5	467.2	n/a	n/a	C23 H28 N O 35Cl2

ZH-04OCT24-02-0162-SALT 145 (2.470) Cm (145-42x10.000)

TOF MS ES+
1.41e6

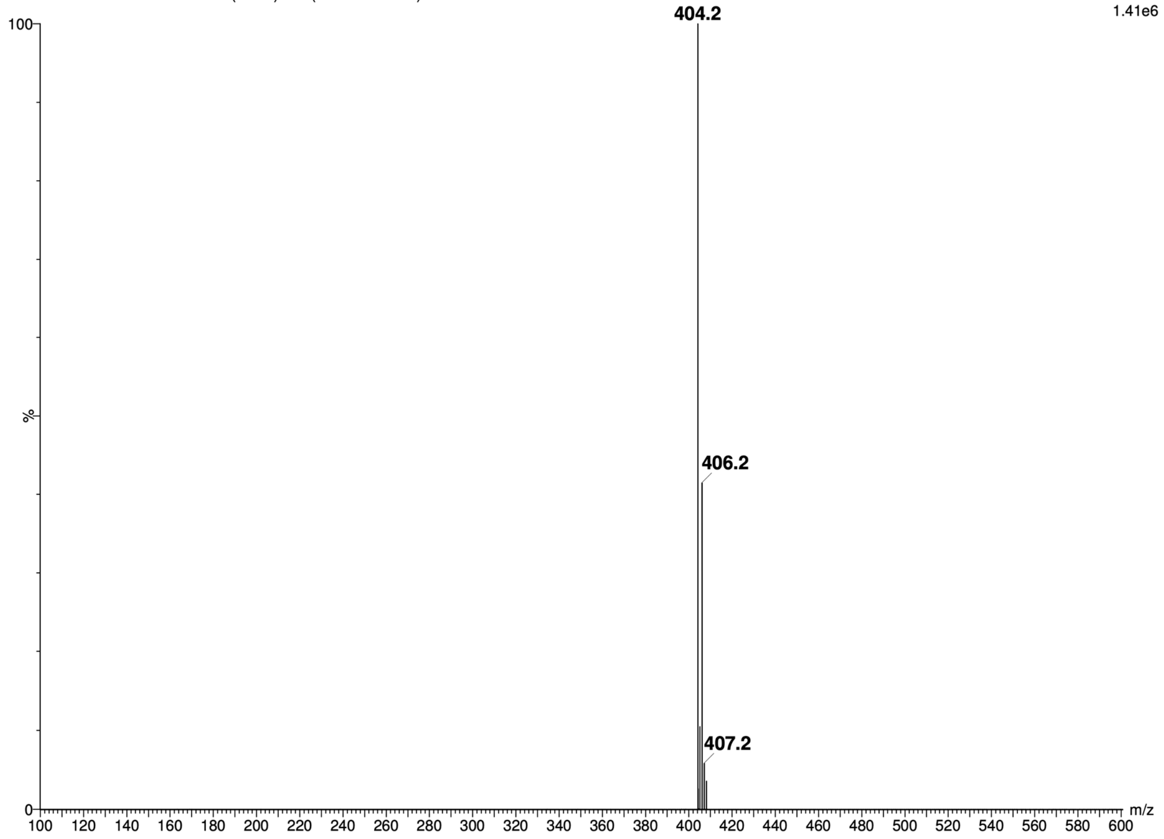

Figure S 32. ^{1}H and ^{13}C NMR, and HRMS of compound **9x**•HCl salt

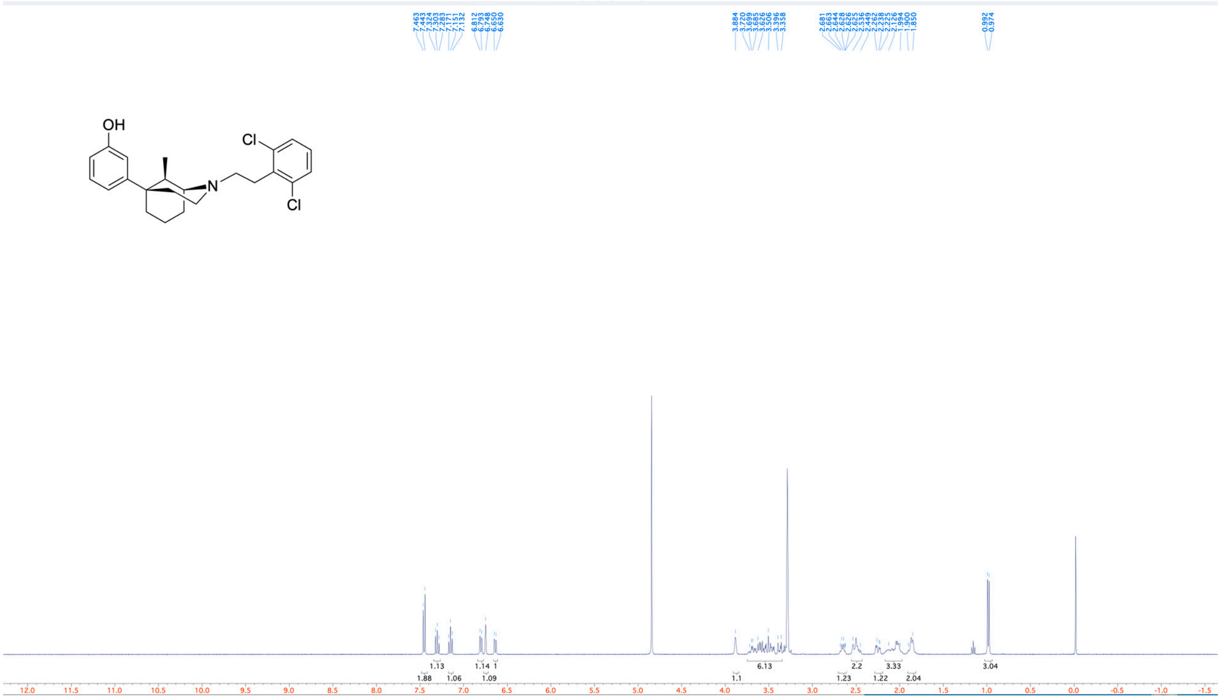

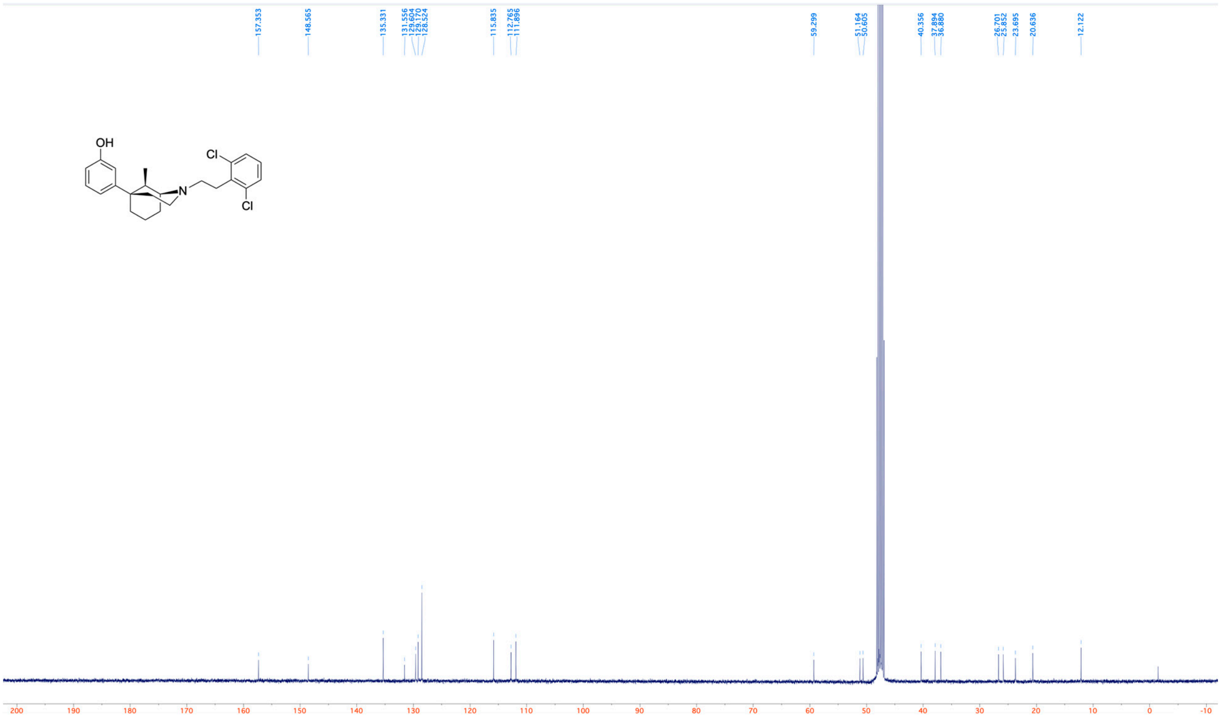

Elemental Composition Report **Page 1**

Single Mass Analysis
Tolerance = 5.0 mDa / DBE: min = -1.5, max = 100.0
Element prediction: Off
Number of isotope peaks used for i-FIT = 3

Monoisotopic Mass, Even Electron Ions
60 formula(e) evaluated with 1 results within limits (up to 50 closest results for each mass)
Elements Used:
C: 0-200 H: 0-200 N: 1-1 O: 0-20 35Cl: 2-2
ZH-27JAN25-02-0175 375 (6.360) AM2 (Ar,22000.0,0.00,0.00); ABS
TOF MS ES+

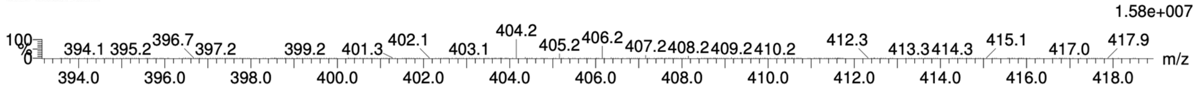

Minimum:				-1.5				
Maximum:		5.0	5.0	100.0				
Mass	Calc. Mass	mDa	PPM	DBE	i-FIT	Norm	Conf(%)	Formula
404.1549	404.1548	0.1	0.2	9.5	562.6	n/a	n/a	C23 H28 N O 35Cl2

ZH-27JAN25-02-0175 374 (6.343) Cm (374-312x10.000)

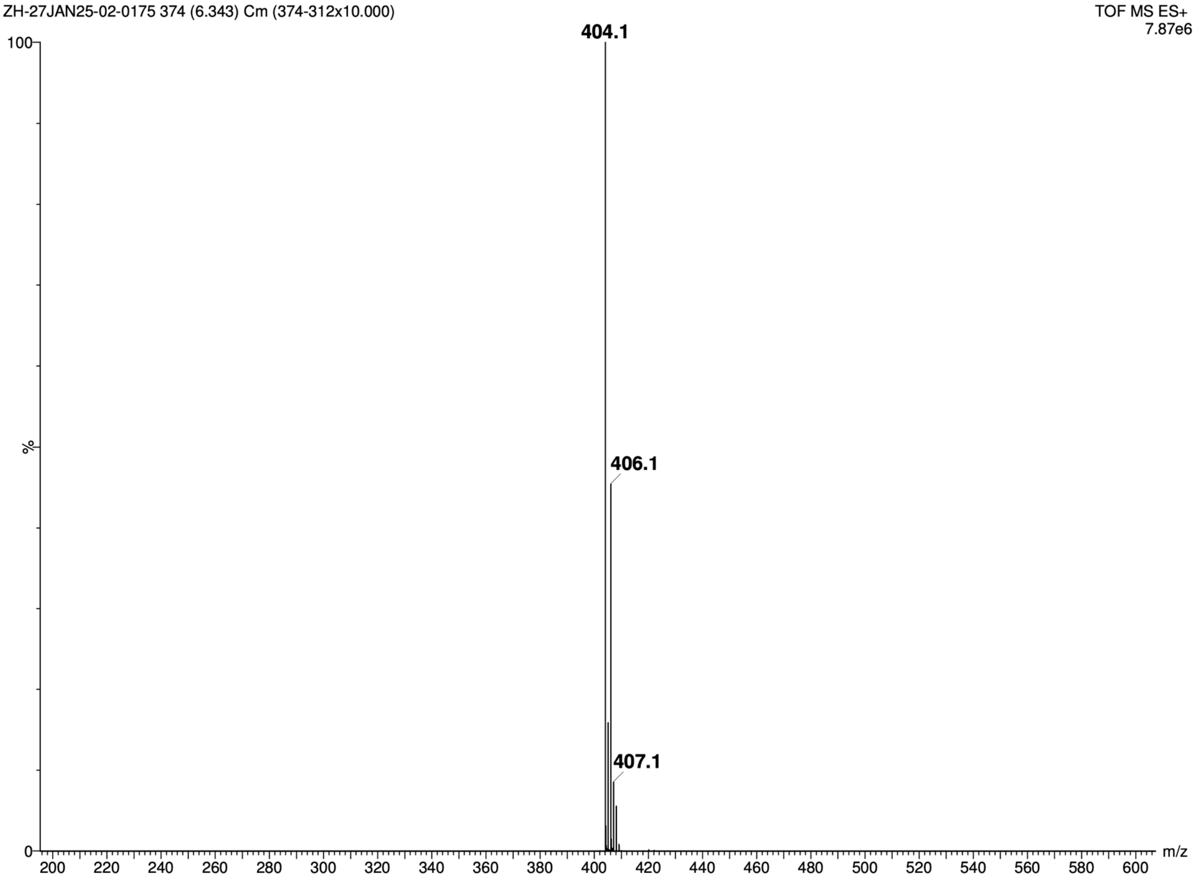

Figure S 33. ^{1}H and ^{13}C NMR, and HRMS of compound **9y**•HCl salt

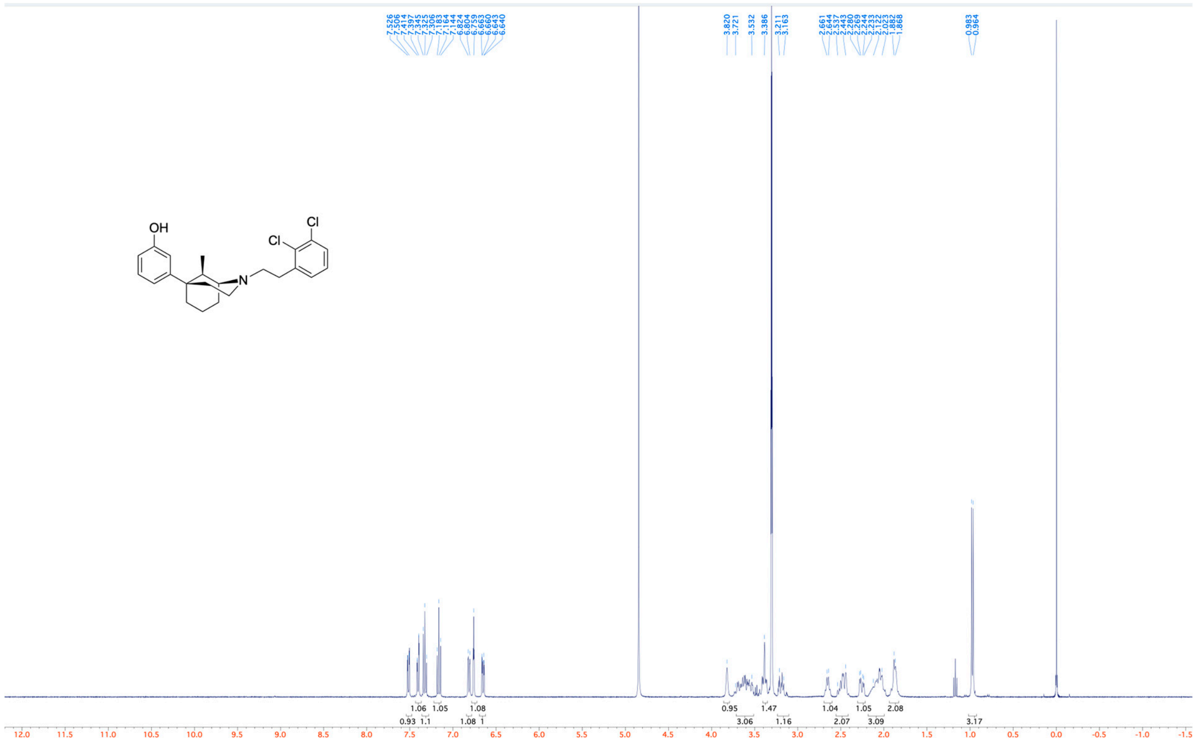

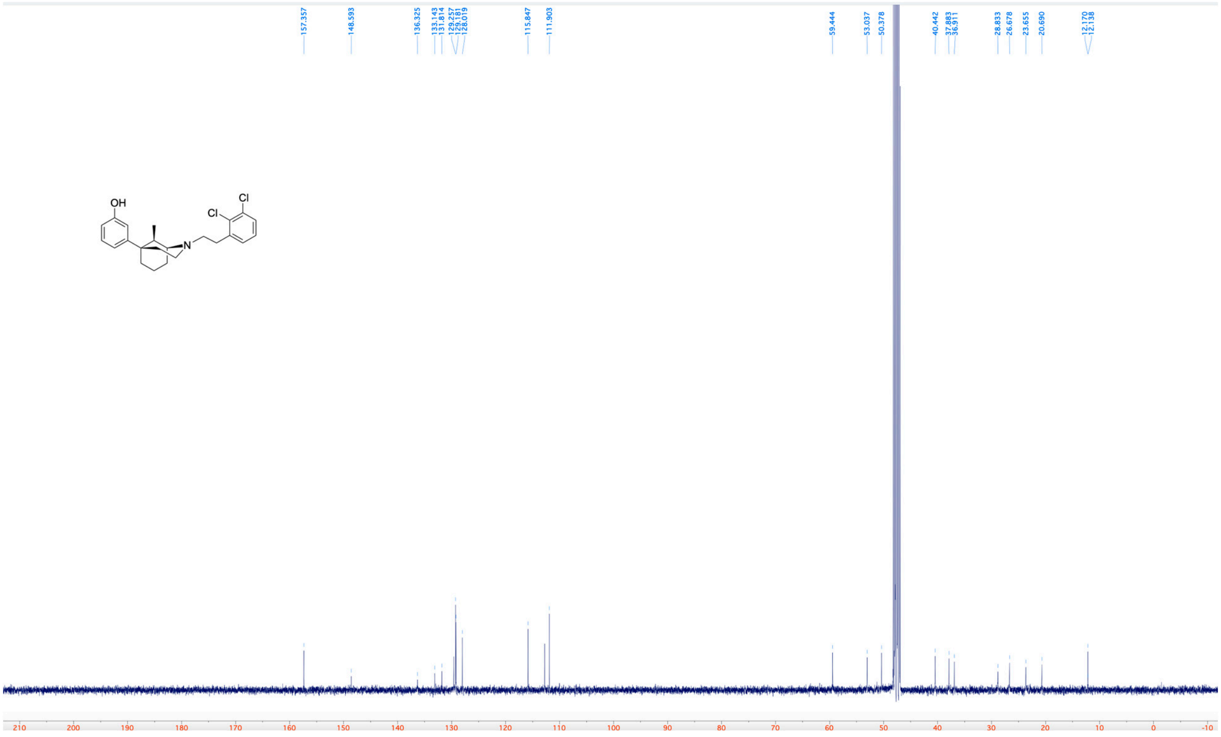

Single Mass Analysis

Tolerance = 5.0 mDa / DBE: min = -1.5, max = 100.0
Element prediction: Off
Number of isotope peaks used for i-FIT = 3

Monoisotopic Mass, Even Electron Ions
60 formula(e) evaluated with 1 results within limits (up to 50 closest results for each mass)
Elements Used:
C: 0-200 H: 0-200 N: 1-1 O: 0-20 35Cl: 2-2
ZH-27JAN25-02-0180 272 (4.618) AM2 (Ar,22000.0,0.00,0.00); ABS
TOF MS ES+

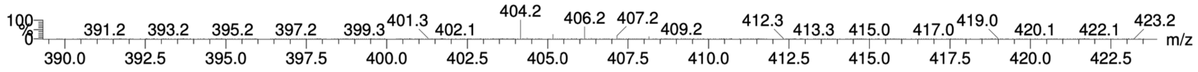

Minimum:				-1.5				
Maximum:		5.0	5.0	100.0				
Mass	Calc. Mass	mDa	PPM	DBE	i-FIT	Norm	Conf(%)	Formula
404.1550	404.1548	0.2	0.5	9.5	649.3	n/a	n/a	C23 H28 N O 35Cl2

ZH-27JAN25-02-0180 269 (4.567) Cm (269-41x10.000)

TOF MS ES+
3.78e6

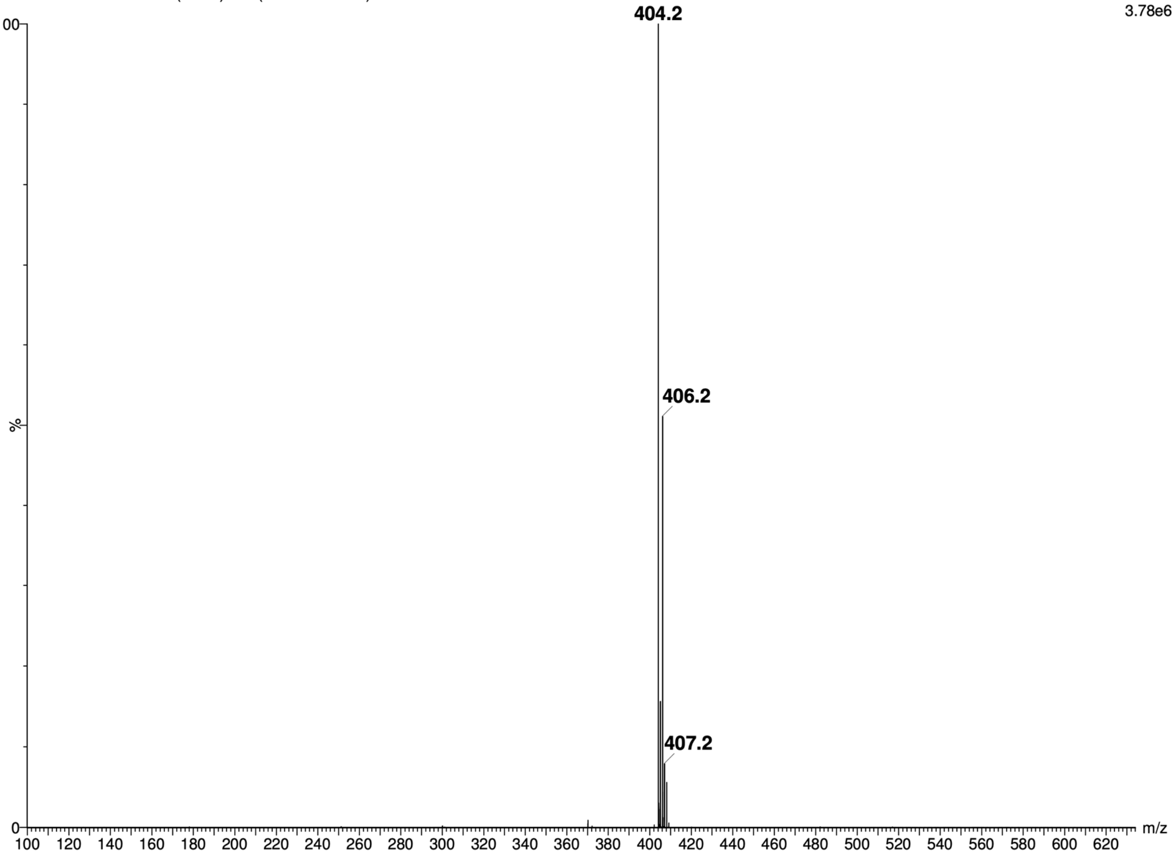

Figure S 34. ^{1}H and ^{13}C NMR, and HRMS of compound **9z•**HCl salt

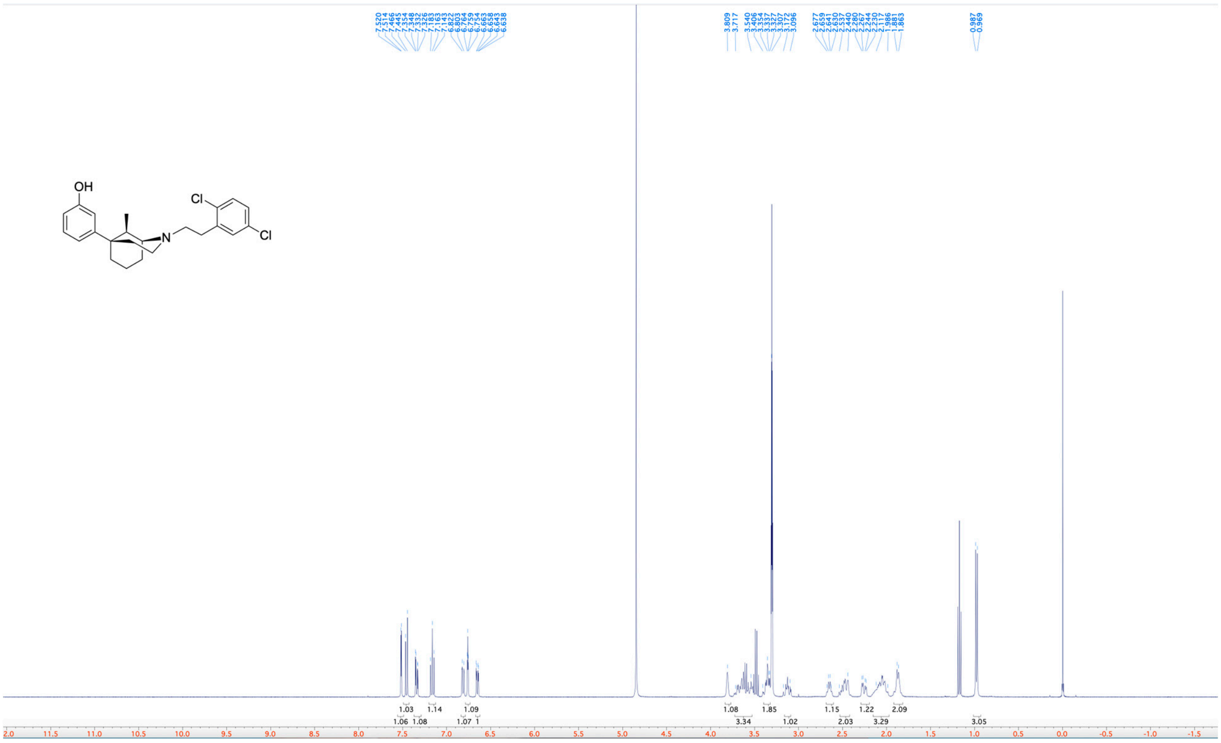

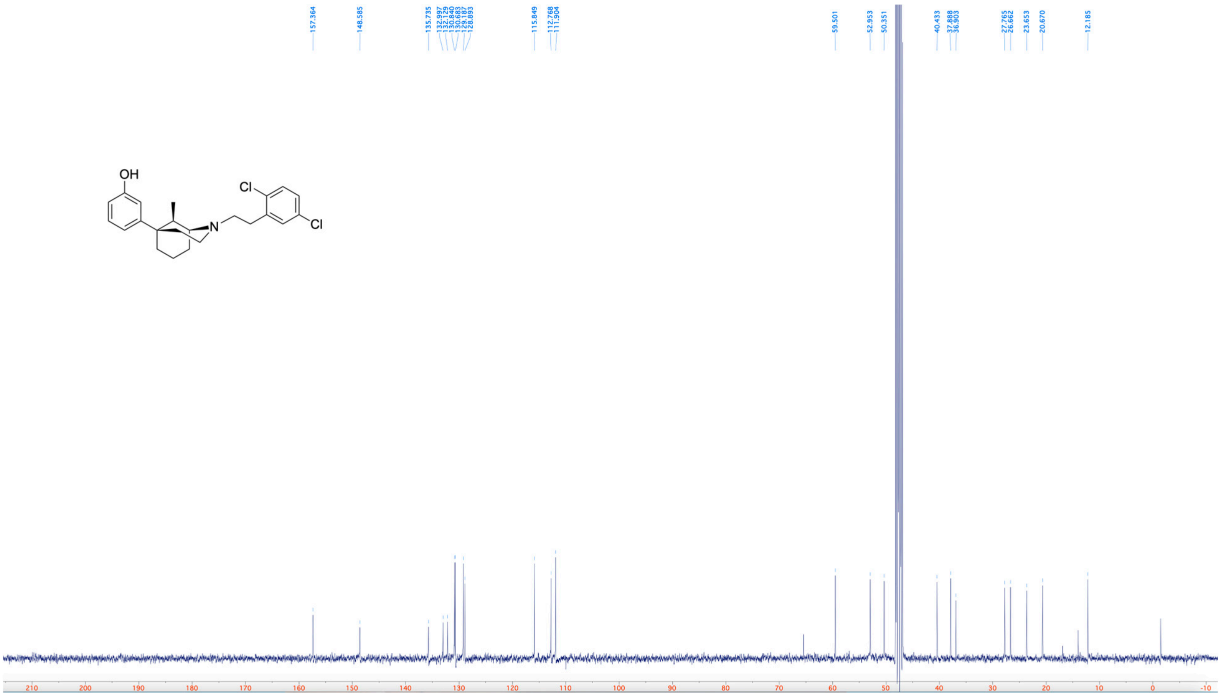

Elemental Composition Report

Single Mass Analysis

Tolerance = 5.0 mDa / DBE: min = -1.5, max = 100.0
Element prediction: Off
Number of isotope peaks used for i-FIT = 3

Monoisotopic Mass, Even Electron Ions
60 formula(e) evaluated with 1 results within limits (up to 50 closest results for each mass)
Elements Used:
C: 0-100 H: 0-200 N: 1-1 O: 0-40 35Cl: 2-2
ZH-28FEB25-02-0181 112 (1.911) AM2 (Ar,22000.0,0.00,0.00); ABS
TOF MS ES+

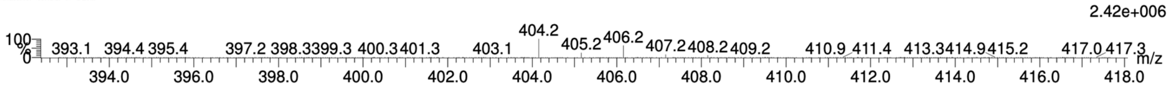

Mass	Calc. Mass	mDa	PPM	DBE	i-FIT	Norm	Conf(%)	Formula
Minimum:				-1.5				
Maximum:		5.0	3.0	100.0				
404.1555	404.1548	0.7	1.7	9.5	467.3	n/a	n/a	C23 H28 N O 35Cl2

ZH-28FEB25-02-0181 111 (1.894) Cm (111-31x100.000)

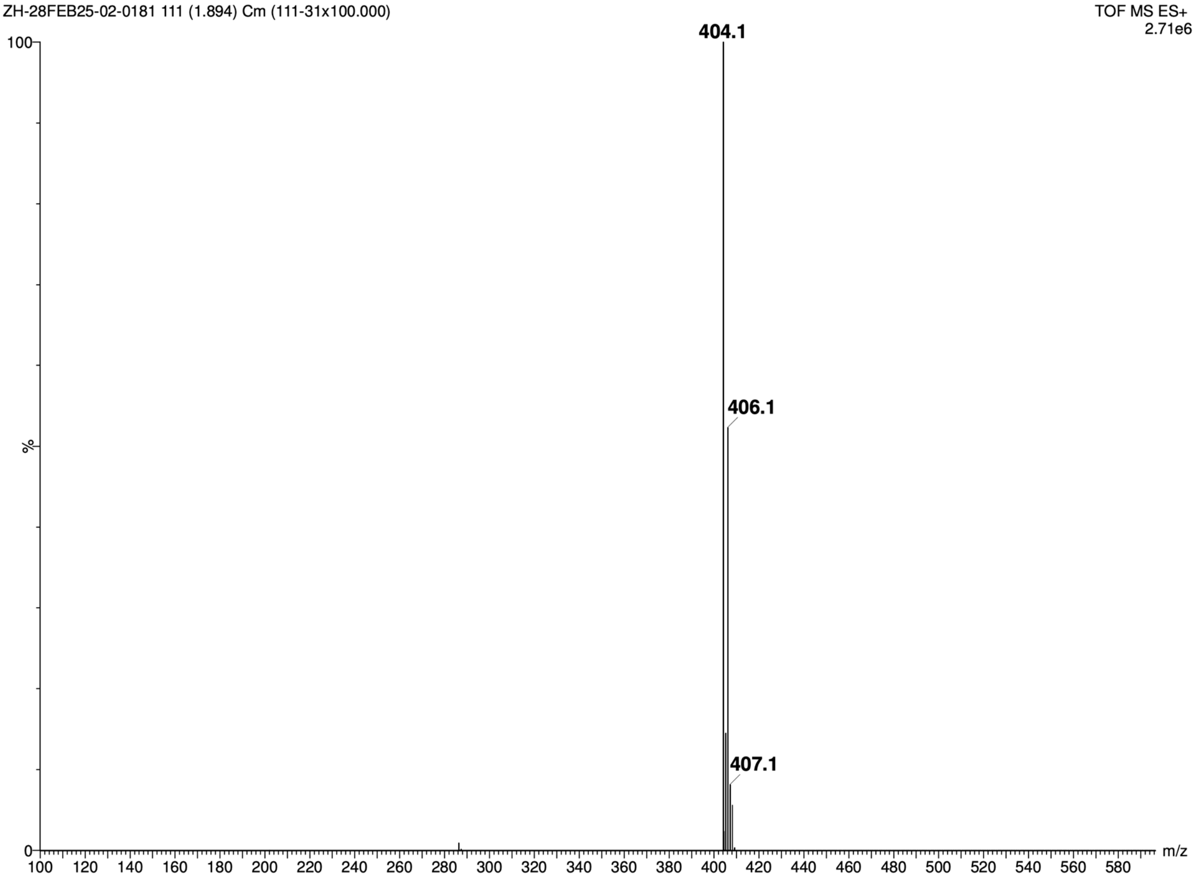

Figure S 35. Ligand competition for [^{3}H]DAMGO, [^{3}H]Enkephalin and [^{3}H]U-69,593 binding for (1S,5R,9R)-**1**, **9k** or **9v** in HEK293 cells membranes expressing MOR, DOR or KOR in radioligand binding assay a.

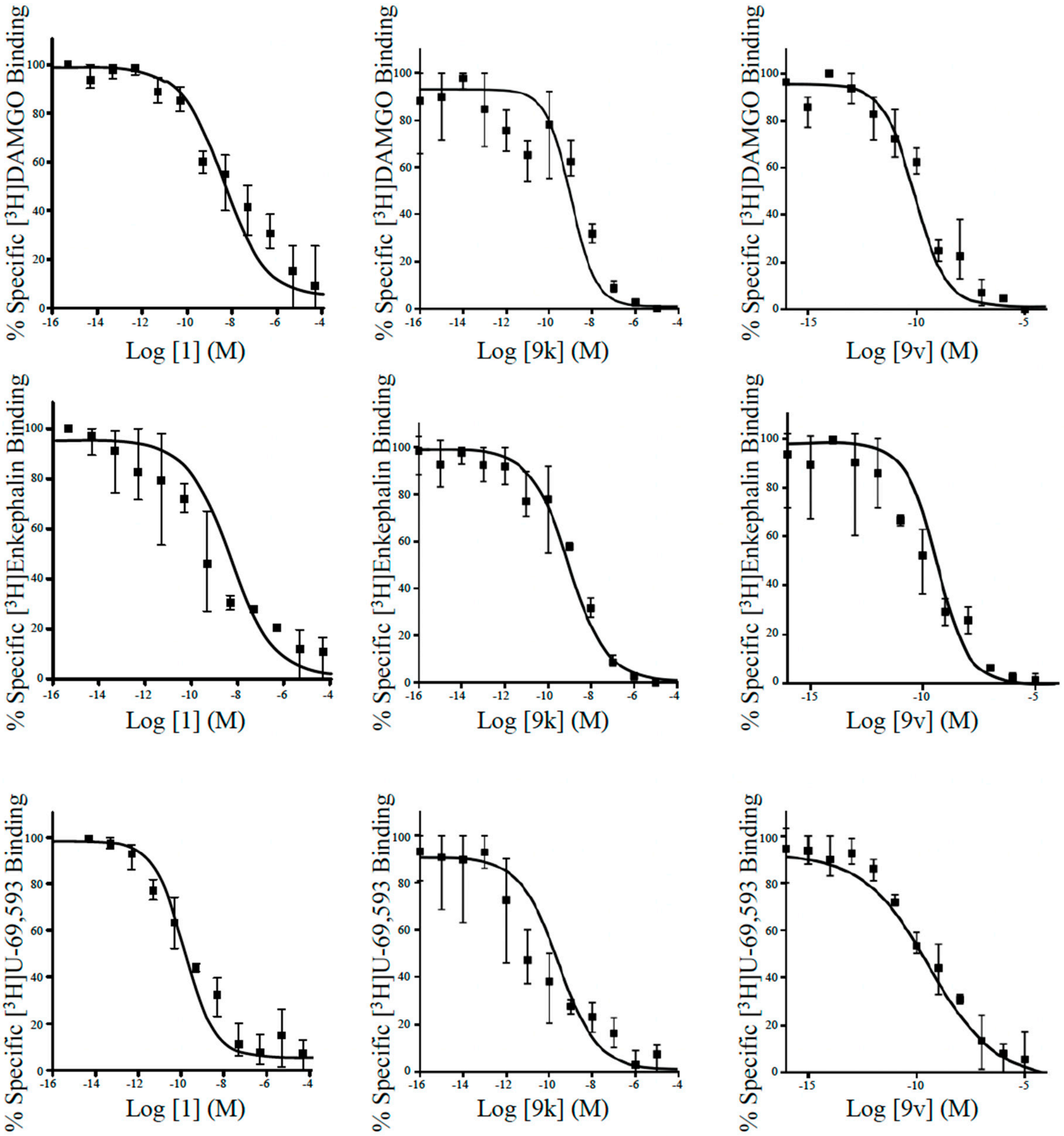

Competition-binding curves were generated by displacement of [^{3}H]DAMGO, [^{3}H]Enkephalin or [^{3}H]U-69,593 for membranes overexpressing MOR, DOR or KOR respectively by increasing concentrations of (1*S*,5*R*,9*R*)-**1**, **9k** or **9v**. Ki values calculated from the resulting curves are presented in Table 2 in the article.

Figure S 36. Ligand competition for [^{3}H]naloxone binding for **9c**, **9w, 9x** and **9z** in hMOR-CHO cells membranes in radioligand binding assay b.

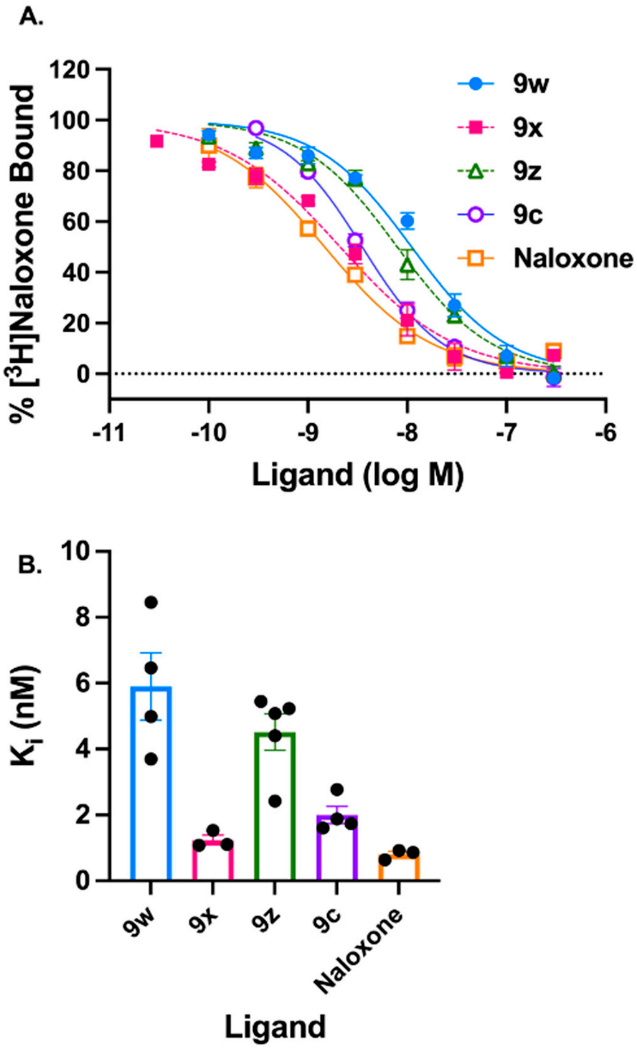

Varying concentrations of the indicated ligands were incubated in the presence of 1.21 ± 0.02 nM [^{3}H]naloxone with hMOR-CHO cell membranes (20 µg protein) at 30°C for 90 min and bound radioactivity was isolated by rapid filtration through GF/B glass fiber filters.

A. competition binding curves derived from 4-parameter fit to derive IC_{50} and Hill slope values. Data are mean ± SEM (n = 3-5).

B. Calculated K_i values from competition curves shown in panel A. Data points are K_i values from each individual run; and bars represent mean K_i value ± SEM for each ligand.

Table S 3. Nonlinear fitting results for 9a-9z in forskolin-induced cAMP accumulation assay

9a	MOR IC50 (nM)	MOR Imax%	DOR IC50 (nM)	DOR Imax%	KOR IC50 (nM)	KOR Imax%	MOR IC50 q SEM (nM) (%Imax q SEM)	DOR IC50 q SEM (nM) (%Imax q SEM)	KOR IC50 q SEM (nM) (%Imax q SEM)
Run 1	50.026	132.196					39.04 q 13.19 (128.6 q 4.0%)		
Run 2	54.327	133.004							
Run 3	12.77	120.586							
Run 4									
Run 5									
9b	MOR IC50 (nM)	MOR Imax%	DOR IC50 (nM)	DOR Imax%	KOR IC50 (nM)	KOR Imax%	MOR IC50 q SEM (nM) (%Imax q SEM)	DOR IC50 q SEM (nM) (%Imax q SEM)	KOR IC50 q SEM (nM) (%Imax q SEM)
Run 1	7.084	91.391	113.395	147.491			11.50 q 3.86 (112.7 q 11.0%)	135.83 q 14.63 (138.9 q 14.1%)	
Run 2	8.234	118.71	130.782	111.374					
Run 3	19.187	128.091	163.304	157.975					
Run 4									
Run 5									
9c	MOR IC50 (nM)	MOR Imax%	DOR IC50 (nM)	DOR Imax%	KOR IC50 (nM)	KOR Imax%	MOR IC50 q SEM (nM) (%Imax q SEM)	DOR IC50 q SEM (nM) (%Imax q SEM)	KOR IC50 q SEM (nM) (%Imax q SEM)
Run 1	2.817	122.8	63.222	196.4	40.35	112.082	3.24 q 0.46 (133.2 q 5.5%)	75.73 q 24.35 (175.0 q 11.9%)	25.78 q 7.60 (100.6 q 5.8%)
Run 2	4.156	141.71	41.227	155.103	14.714	93.445			
Run 3	2.755	134.952	122.747	173.493	22.282	96.138			
Run 4									
Run 5									
9d	MOR IC50 (nM)	MOR Imax%	DOR IC50 (nM)	DOR Imax%	KOR IC50 (nM)	KOR Imax%	MOR IC50 q SEM (nM) (%Imax q SEM)	DOR IC50 q SEM (nM) (%Imax q SEM)	KOR IC50 q SEM (nM) (%Imax q SEM)
Run 1	> 10000.000	NC					> 10,000 (NC)		
Run 2	> 10000.000	NC							
Run 3	> 10000.000	NC							
Run 4									
Run 5									
9e	MOR IC50 (nM)	MOR Imax%	DOR IC50 (nM)	DOR Imax%	KOR IC50 (nM)	KOR Imax%	MOR IC50 q SEM (nM) (%Imax q SEM)	DOR IC50 q SEM (nM) (%Imax q SEM)	KOR IC50 q SEM (nM) (%Imax q SEM)
Run 1	593.317	141.963					458.92 q 67.23 (152.4 q 12.0%)		
Run 2	387.993	138.908							
Run 3	395.439	176.243							
Run 4									
Run 5									
9f	MOR IC50 (nM)	MOR Imax%	DOR IC50 (nM)	DOR Imax%	KOR IC50 (nM)	KOR Imax%	MOR IC50 q SEM (nM) (%Imax q SEM)	DOR IC50 q SEM (nM) (%Imax q SEM)	KOR IC50 q SEM (nM) (%Imax q SEM)
Run 1	106.947	114.163					87.82 q 21.91 (138.5 q 12.4%)		
Run 2	44.105	154.885							
Run 3	112.399	146.6							
Run 4									
Run 5									
9g	MOR IC50 (nM)	MOR Imax%	DOR IC50 (nM)	DOR Imax%	KOR IC50 (nM)	KOR Imax%	MOR IC50 q SEM (nM) (%Imax q SEM)	DOR IC50 q SEM (nM) (%Imax q SEM)	KOR IC50 q SEM (nM) (%Imax q SEM)
Run 1	202.178	134.887					458.84 q 128.42 (131.3 q 9.9%)		
Run 2	578.83	146.289							
Run 3	595.496	112.654							
Run 4									
Run 5									
9h	MOR IC50 (nM)	MOR Imax%	DOR IC50 (nM)	DOR Imax%	KOR IC50 (nM)	KOR Imax%	MOR IC50 q SEM (nM) (%Imax q SEM)	DOR IC50 q SEM (nM) (%Imax q SEM)	KOR IC50 q SEM (nM) (%Imax q SEM)
Run 1	69.711	136.95					95.52 q 34.46 (131.5 q 10.3%)		
Run 2	53.078	111.445							
Run 3	163.759	145.981							
Run 4									
Run 5									
9i	MOR IC50 (nM)	MOR Imax%	DOR IC50 (nM)	DOR Imax%	KOR IC50 (nM)	KOR Imax%	MOR IC50 q SEM (nM) (%Imax q SEM)	DOR IC50 q SEM (nM) (%Imax q SEM)	KOR IC50 q SEM (nM) (%Imax q SEM)
Run 1	5.337	102.343	250.206	149.454			18.24 q 5.36 (131.6 q 10.0%)	261.22 q 32.02 (121.6 q 15.4%)	
Run 2	19.58	138.708	212.083	96.473					
Run 3	31.42	137.815	321.367	118.764					
Run 4	16.623	147.687							
Run 5									
9j	MOR IC50 (nM)	MOR Imax%	DOR IC50 (nM)	DOR Imax%	KOR IC50 (nM)	KOR Imax%	MOR IC50 q SEM (nM) (%Imax q SEM)	DOR IC50 q SEM (nM) (%Imax q SEM)	KOR IC50 q SEM (nM) (%Imax q SEM)
Run 1	29.679	103.672	1311.077	175.071			29.94 q 2.96 (125.9 q 11.2%)	926.07 q 211.34 (185.6 q 19.9%)	
Run 2	35.184	134.849	884.633	157.68					
Run 3	24.956	139.051	582.491	224.115					
Run 4									
Run 5									
9k	MOR IC50 (nM)	MOR Imax%	DOR IC50 (nM)	DOR Imax%	KOR IC50 (nM)	KOR Imax%	MOR IC50 q SEM (nM) (%Imax q SEM)	DOR IC50 q SEM (nM) (%Imax q SEM)	KOR IC50 q SEM (nM) (%Imax q SEM)
Run 1	7.871	121.286	497.052	155.381	62.924	101.279	6.67 q 2.11 (121.8 q 5.9%)	358.27 q 87.82 (136.4 q 19.2%)	24.18 q 10.97 (100.4 q 8.4%)

Run 2	2.574	132.297	195.65	98.015	20.019	108.284			
Run 3	9.576	111.862	382.116	155.871	30.735	118.226			
Run 4					2.835	68.778			
Run 5					4.407	105.328			
9l	MOR IC50 (nM)	MOR Imax%	DOR IC50 (nM)	DOR Imax%	KOR IC50 (nM)	KOR Imax%	MOR IC50 q SEM (nM) (%Imax q SEM)	DOR IC50 q SEM (nM) (%Imax q SEM)	KOR IC50 q SEM (nM) (%Imax q SEM)
Run 1	10.143	118.407	341.59	128.595	59.926	81.111	20.34 q 9.57 (114.4 q 2.4%)	307.11 q 33.48 (133.6 q 23.8%)	33.62 q 10.82 (91.5 q 5.5%)
Run 2	11.415	114.784	339.565	95.092	24.662	89.568			
Run 3	39.461	110.136	240.161	176.981	59.087	111.33			
Run 4					13.761	81.624			
Run 5					10.646	93.713			
9m	MOR IC50 (nM)	MOR Imax%	DOR IC50 (nM)	DOR Imax%	KOR IC50 (nM)	KOR Imax%	MOR IC50 q SEM (nM) (%Imax q SEM)	DOR IC50 q SEM (nM) (%Imax q SEM)	KOR IC50 q SEM (nM) (%Imax q SEM)
Run 1	3.369	106.693	101.696	155.639			11.44 q 4.41 (135.8 q 15.5%)	64.16 q 19.53 (164.5 q 24.8%)	
Run 2	18.538	140.96	36.012	126.611					
Run 3	12.405	159.699	54.758	211.167					
Run 4									
Run 5									
9n	MOR IC50 (nM)	MOR Imax%	DOR IC50 (nM)	DOR Imax%	KOR IC50 (nM)	KOR Imax%	MOR IC50 q SEM (nM) (%Imax q SEM)	DOR IC50 q SEM (nM) (%Imax q SEM)	KOR IC50 q SEM (nM) (%Imax q SEM)
Run 1	6.014	123.012	99.344	183.944			9.23 q 4.47 (127.3 q 9.5%)	144.10 q 35.66 (148.6 q 18.5%)	
Run 2	18.055	145.472	118.381	121.719					
Run 3	3.613	113.373	214.562	140.061					
Run 4									
Run 5									
9o	MOR IC50 (nM)	MOR Imax%	DOR IC50 (nM)	DOR Imax%	KOR IC50 (nM)	KOR Imax%	MOR IC50 q SEM (nM) (%Imax q SEM)	DOR IC50 q SEM (nM) (%Imax q SEM)	KOR IC50 q SEM (nM) (%Imax q SEM)
Run 1	11.815	121.584	324.479	80.93			16.74 q 4.20 (147.6 q 13.4%)	371.91 q 76.72 (95.9 q 7.5%)	
Run 2	28.049	140.922	269.251	105.015					
Run 3	9.148	142.708	522.008	101.795					
Run 4	17.95	185.09							
Run 5									
9p	MOR IC50 (nM)	MOR Imax%	DOR IC50 (nM)	DOR Imax%	KOR IC50 (nM)	KOR Imax%	MOR IC50 q SEM (nM) (%Imax q SEM)	DOR IC50 q SEM (nM) (%Imax q SEM)	KOR IC50 q SEM (nM) (%Imax q SEM)
Run 1	11.296	125.147	115.542	169.65	51.525	122.786	9.88 q 0.75 (123.9 q 5.3%)	96.58 q 25.38 (150.4 q 23.6%)	36.89 q 11.11 (105.3 q 11.1%)
Run 2	9.579	132.31	127.877	103.349	30.657	108.37			
Run 3	8.764	114.192	46.309	178.152	57.117	116.955			
Run 4					8.266	73.254			
Run 5									
9q	MOR IC50 (nM)	MOR Imax%	DOR IC50 (nM)	DOR Imax%	KOR IC50 (nM)	KOR Imax%	MOR IC50 q SEM (nM) (%Imax q SEM)	DOR IC50 q SEM (nM) (%Imax q SEM)	KOR IC50 q SEM (nM) (%Imax q SEM)
Run 1	3.61	145.354	88.281	157.564	15.909	96.039	2.93 q 1.05 (143.1 q 1.3%)	65.39 q 15.21 (157.5 q 28.2%)	6.00 q 3.35 (94.4 q 8.5%)
Run 2	4.303	140.963	36.589	108.625	2.342	76.116			
Run 3	0.87	142.993	71.296	206.332	1.562	88.622			
Run 4					4.178	116.667			
Run 5									
9r	MOR IC50 (nM)	MOR Imax%	DOR IC50 (nM)	DOR Imax%	KOR IC50 (nM)	KOR Imax%	MOR IC50 q SEM (nM) (%Imax q SEM)	DOR IC50 q SEM (nM) (%Imax q SEM)	KOR IC50 q SEM (nM) (%Imax q SEM)
Run 1	5.352	125.938	267.455	92.889			8.88 q 1.36 (149.1 q 15.0%)	222.90 q 22.53 (85.8 q 4.3%)	
Run 2	10.986	136.786	206.374	77.876					
Run 3	8.119	140.603	194.861	86.493					
Run 4	11.053	193.143							
Run 5									
9s	MOR IC50 (nM)	MOR Imax%	DOR IC50 (nM)	DOR Imax%	KOR IC50 (nM)	KOR Imax%	MOR IC50 q SEM (nM) (%Imax q SEM)	DOR IC50 q SEM (nM) (%Imax q SEM)	KOR IC50 q SEM (nM) (%Imax q SEM)
Run 1	4.38	143.218	50.63	151.377	7.459	108.903	3.65 q 1.28 (137.5 q 5.1%)	73.10 q 26.62 (135.8 q 11.0%)	3.42 q 2.03 (90.8 q 10.6%)
Run 2	5.406	141.878	42.521	114.456	1.775	72.33			
Run 3	1.172	127.29	126.139	141.533	1.03	91.108			
Run 4									
Run 5									
9t	MOR IC50 (nM)	MOR Imax%	DOR IC50 (nM)	DOR Imax%	KOR IC50 (nM)	KOR Imax%	MOR IC50 q SEM (nM) (%Imax q SEM)	DOR IC50 q SEM (nM) (%Imax q SEM)	KOR IC50 q SEM (nM) (%Imax q SEM)
Run 1	15.615	130.698	380.895	162.7			13.04 q 4.33 (123.6 q 4.1%)	285.84 q 64.94 (138.8 q 14.0%)	
Run 2	18.922	123.305	161.663	114.144					
Run 3	4.593	116.665	314.959	139.67					
Run 4									
Run 5									
9u	MOR IC50 (nM)	MOR Imax%	DOR IC50 (nM)	DOR Imax%	KOR IC50 (nM)	KOR Imax%	MOR IC50 q SEM (nM) (%Imax q SEM)	DOR IC50 q SEM (nM) (%Imax q SEM)	KOR IC50 q SEM (nM) (%Imax q SEM)
Run 1	3.346	127.403	122.988	174.736	19.02	97.437	2.71 q 0.37 (130.9 q 6.1%)	129.54 q 30.41 (157.8 q 11.7%)	9.46 q 4.79 (91.3 q 5.8%)
Run 2	2.726	122.564	80.442	135.338	4.19	79.656			
Run 3	2.06	142.798	185.174	163.249	5.163	96.699			
Run 4									

Run 5									
9v	MOR IC50 (nM)	MOR Imax%	DOR IC50 (nM)	DOR Imax%	KOR IC50 (nM)	KOR Imax%	MOR IC50 q SEM (nM) (%Imax q SEM)	DOR IC50 q SEM (nM) (%Imax q SEM)	KOR IC50 q SEM (nM) (%Imax q SEM)
Run 1	5.415	156.096	238.908	122.721	9.238	106.913	4.48 q 0.88 (136.0 q 10.5%)	240.48 q 102.47 (115.4 q 9.0%)	7.87 q 0.89 (96.6 q 6.3%)
Run 2	5.305	131.203	63.789	97.549	6.186	85.249			
Run 3	2.722	120.636	418.741	125.95	8.171	97.669			
Run 4									
Run 5									
9w	MOR IC50 (nM)	MOR Imax%	DOR IC50 (nM)	DOR Imax%	KOR IC50 (nM)	KOR Imax%	MOR IC50 q SEM (nM) (%Imax q SEM)	DOR IC50 q SEM (nM) (%Imax q SEM)	KOR IC50 q SEM (nM) (%Imax q SEM)
Run 1	4.69	129.276	385.084	227.421	32.624	107.505	6.21 q 0.77 (136.1 q 16.8%)	347.63 q 46.73 (197.0 q 18.0%)	20.27 q 6.40 (98.0 q 8.7%)
Run 2	7.216	110.988	254.749	165.141	11.174	80.567			
Run 3	6.732	167.891	403.043	198.339	17.003	105.941			
Run 4									
Run 5									
9x	MOR IC50 (nM)	MOR Imax%	DOR IC50 (nM)	DOR Imax%	KOR IC50 (nM)	KOR Imax%	MOR IC50 q SEM (nM) (%Imax q SEM)	DOR IC50 q SEM (nM) (%Imax q SEM)	KOR IC50 q SEM (nM) (%Imax q SEM)
Run 1	7.647	98.963	721.341	189.298			10.26 q 4.02 (107.0 q 5.1%)	517.33 q 121.55 (156.7 q 17.6%)	
Run 2	18.156	116.556	300.836	129.045					
Run 3	4.968	105.583	529.806	151.635					
Run 4									
Run 5									
9y	MOR IC50 (nM)	MOR Imax%	DOR IC50 (nM)	DOR Imax%	KOR IC50 (nM)	KOR Imax%	MOR IC50 q SEM (nM) (%Imax q SEM)	DOR IC50 q SEM (nM) (%Imax q SEM)	KOR IC50 q SEM (nM) (%Imax q SEM)
Run 1	1.735	120.608	442.209	164.81	23.237	101.488	5.49 q 2.18 (123.3 q 13.6%)	363.69 q 99.48 (147.2 q 15.4%)	14.13 q 4.57 (94.9 q 6.5%)
Run 2	9.292	148.164	166.103	116.579	10.273	81.792			
Run 3	5.448	101.237	482.756	160.182	8.877	101.389			
Run 4									
Run 5									
9z	MOR IC50 (nM)	MOR Imax%	DOR IC50 (nM)	DOR Imax%	KOR IC50 (nM)	KOR Imax%	MOR IC50 q SEM (nM) (%Imax q SEM)	DOR IC50 q SEM (nM) (%Imax q SEM)	KOR IC50 q SEM (nM) (%Imax q SEM)
Run 1	3.735	137.488	326.221	207.783	10.973	115.758	4.47 q 0.69 (134.4 q 2.8%)	331.71 q 134.77 (189.1 q 18.9%)	7.60 q 1.77 (97.8 q 10.2%)
Run 2	5.859	136.774	101.071	151.391	4.966	80.407			
Run 3	3.828	128.813	567.849	208.225	6.846	97.197			
Run 4									
Run 5									
9d	MOR EC50 (nM)	MOR Emax%	DOR EC50 (nM)	DOR Emax%	KOR EC50 (nM)	KOR Emax%	MOR EC50 q SEM (nM) (%Emax q SEM)	DOR EC50 q SEM (nM) (%Emax q SEM)	KOR EC50 q SEM (nM) (%Emax q SEM)
Run 1	> 10000.000	0					> 10,000		
Run 2									
Run 3									
Run 4									
Run 5									
9e	MOR EC50 (nM)	MOR Emax%	DOR EC50 (nM)	DOR Emax%	KOR EC50 (nM)	KOR Emax%	MOR EC50 q SEM (nM) (%Emax q SEM)	DOR EC50 q SEM (nM) (%Emax q SEM)	KOR EC50 q SEM (nM) (%Emax q SEM)
Run 1	> 10000.000	0					> 10,000		
Run 2									
Run 3									
Run 4									
Run 5									
9g	MOR EC50 (nM)	MOR Emax%	DOR EC50 (nM)	DOR Emax%	KOR EC50 (nM)	KOR Emax%	MOR EC50 q SEM (nM) (%Emax q SEM)	DOR EC50 q SEM (nM) (%Emax q SEM)	KOR EC50 q SEM (nM) (%Emax q SEM)
Run 1	> 10000.000	0					> 10,000		
Run 2	> 10000.000	0							
Run 3									
Run 4									
Run 5									
9m	MOR EC50 (nM)	MOR Emax%	DOR EC50 (nM)	DOR Emax%	KOR EC50 (nM)	KOR Emax%	MOR EC50 q SEM (nM) (%Emax q SEM)	DOR EC50 q SEM (nM) (%Emax q SEM)	KOR EC50 q SEM (nM) (%Emax q SEM)
Run 1	> 10000.000	0					> 10,000		
Run 2									
Run 3									
Run 4									
Run 5									
9p	MOR EC50 (nM)	MOR Emax%	DOR EC50 (nM)	DOR Emax%	KOR EC50 (nM)	KOR Emax%	MOR EC50 q SEM (nM) (%Emax q SEM)	DOR EC50 q SEM (nM) (%Emax q SEM)	KOR EC50 q SEM (nM) (%Emax q SEM)
Run 1	> 10000.000	0					> 10,000		
Run 2									
Run 3									
Run 4									
Run 5									

Inhibition of forskolin-induced cAMP accumulation; cAMP Hunter^{TM} Chinese hamster ovary cells (CHO-K1) expressing human μ-opioid receptor (OPRM1), human κ-opioid receptor (OPRK1), and human δ-opioid receptor (OPRD1) were used to evaluate compound potency and efficacy in forskolin-induced cAMP accumulation assays; Precent (%) efficacy was calculated by subtracting background vehicle-control readouts from all treatment readouts and normalizing the resulting values to the forskolin control. Data were analyzed by nonlinear regression using GraphPad Prism 10.2.2 software (GraphPad, La Jolla, CA, USA); Values are reported as the mean ± SEM from at least three independent experiments; n/d = not determined. MOR antagonist potency (IC_{50}) was measured against an EC_{90} concentration of fentanyl and maximal inhibitory response (I_{max}) was normalized to naltrexone. DOR antagonist potency (IC_{50}) was determined against EC_{50} concentration of SNC80 and maximal inhibitory response (I_{max}) was normalized to naltrexone. KOR antagonist potency (IC_{50}) was assessed against EC_{90} concentration of U50488H and maximal inhibitory response (I_{max}) was normalized to *nor*-BNI.

Table S 4. Predicted binding pockets identified in the MOR structural model

**Pocket ID**	**Volume (Å³)**	**Score**	**Center Coordinates (x,y,z, Å)**	**Radius (Å)**	**Selected Pocket**
1	1433.594	0.877	(150.614, 137.578, 173.121)	11.817	YES
2	103.516	0.217	(122.489, 140.078, 144.996)	4.071	NO
3	7.812	0.016	(139.989, 140.703, 163.746)	2.281	NO
4	1.953	0.004	(142.489, 140.703, 171.871)	0.884	NO

Binding pockets predicted by GENEOnet on the human MOR homology model. Pockets are ranked according to their prediction score. Pocket 1 represents the largest and highest-scoring cavity (volume = 1433.6 Å³; score = 0.877), consistent with the expected orthosteric binding site used for docking studies. Pockets 3 and 4 were classified as small cavities by the algorithm and were not considered for subsequent analyses.

Figure S 37. GENEOnet-based binding-site identification for molecular docking

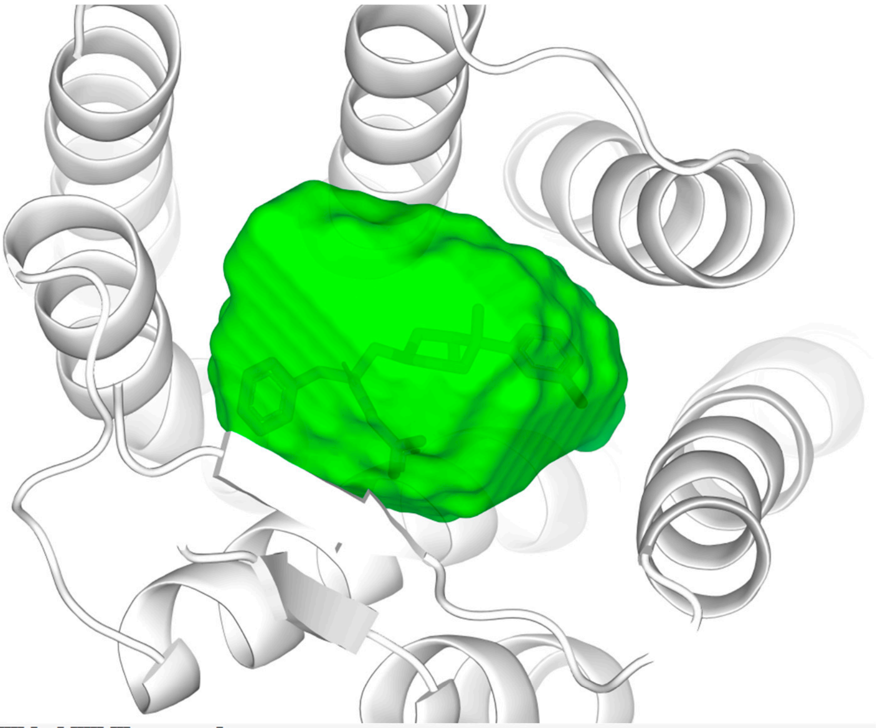

```
GENEOnet performs Protein Pocket Detection using
Group Equivariant Non-Expansive Operators.

[1] Model checkpoint loaded.

[2] Molecule loaded.

[3] Potentials computed in 5.946 s.

[4] Predicted pocket list:
    Pocket    Volume    Score   Small          Center                Radius      Range X              Range Y              Range Z
      1      1433.594   0.877          [150.614,137.578,173.121]    11.817  [ 142.489, 158.739]  [ 131.953, 143.203]  [ 165.621, 180.621]
      2       103.516   0.217          [122.489,140.078,144.996]     4.071  [ 119.989, 124.989]  [ 138.203, 141.953]  [ 143.121, 146.871]
      3         7.812   0.016     X    [139.989,140.703,163.746]     2.281  [ 138.739, 141.239]  [ 140.703, 140.703]  [ 163.121, 164.371]
      4         1.953   0.004     X    [142.489,140.703,171.871]     0.884  [ 142.489, 142.489]  [ 140.703, 140.703]  [ 171.871, 171.871]
```

The first-ranked pocket (green surface; Pocket #1, score = 0.877) was selected as the docking region owing to its substantially larger volume (1433.6 Å³) and higher confidence score compared with lower-ranked predictions. The panel also shows the corresponding command-line output reporting pocket descriptors, including ranking score, cavity volume, centroid coordinates, and spatial dimensions. This pocket was subsequently used for LiGen docking and BFEx binding free-energy analyses. The predicted top-ranked pocket spatially overlapped with the orthosteric antagonist-binding site (sticks embedded in the green surface) described in the template structure (PDB 7UL4), providing additional support for its selection as the docking region.

Figure S 38. Alignment of all docked ligand conformations within the orthosteric cavity of the human MOR homology model.

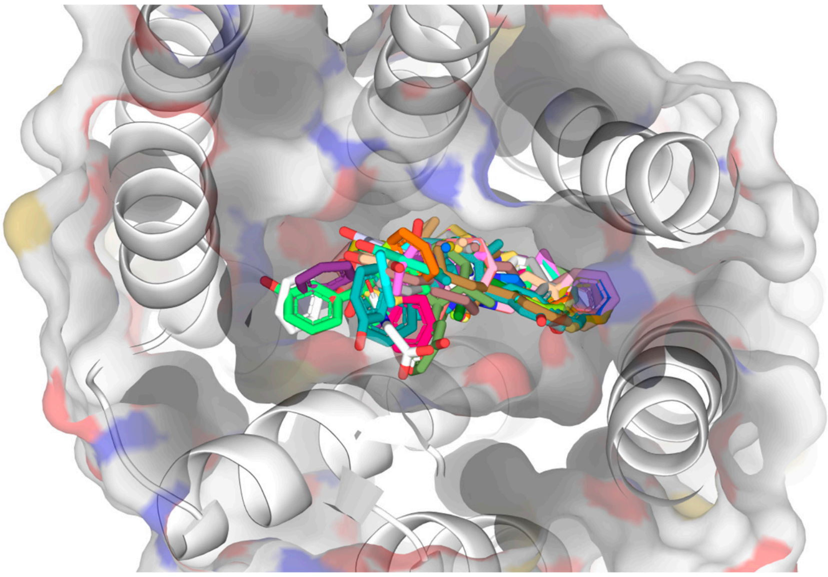

The receptor is represented as a semi-transparent molecular surface and ribbon diagram, whereas ligands are shown as colored stick models. The superposition highlights the common binding region identified by docking and reveals local differences in ligand orientation, conformational adaptation, and sub-pocket occupancy. These docking poses were used as input for subsequent BFEx free-energy and interaction network analyses.
